# The calmodulin fused kinase novel gene family is the major system in plants converting Ca^2+^ signals to protein phosphorylation responses

**DOI:** 10.1038/s41598-017-03367-8

**Published:** 2017-06-23

**Authors:** Fei Chen, Liangsheng Zhang, Zong-Ming (Max) Cheng

**Affiliations:** 1 College of Horticulture, Nanjing Agricultural University, Nanjing, 210095 China; 2 Department of Plant Sciences, University of Tennessee, Knoxville, TN 37996 USA; 3 Center for Genomics and Biotechnology, HIST, Fujian Agriculture and Forestry University, Fuzhou, 350002 China

## Abstract

Eukaryotes utilize Ca^2+^ as a universal second messenger to convert and multiply environmental and developmental signals to downstream protein phosphorylation responses. However, the phylogenetic relationships of the genes that convert Ca^2+^ signal (CS) to protein phosphorylation responses (PPRs) remain highly controversial, and their origin and evolutionary trajectory are unclear, which greatly hinders functional studies. Here we examined the deep phylogeny of eukaryotic CS converter gene families and identified a phylogenetically and structurally distinctive monophyly in Archaeplastida. This monophyly can be divided into four subfamilies, and each can be traced to ancestral members that contain a kinase domain and a calmodulin-like domain. This strongly indicates that the ancestor of this monophyly originated by a *de novo* fusion of a kinase gene and a calmodulin gene. This gene family, with a proposed new name, Calmodulin Fused Kinase (CFK), had expanded and diverged significantly both in sizes and in structures for efficient and accurate Ca^2+^ signalling, and was shown to play pivotal roles in all the six major plant adaptation events in evolution. Our findings elucidated the common origin of all CS-PPR converter genes except CBL-CIPK converter genes, and revealed that CFKs act as the main CS conversion system in plants.

## Introduction

Unlike prokaryotes that rely on the two-component (histidine kinase and response regulator protein) system for intracellular signalling^1, 2^, animals and plants use Ca^2+^ as the intracellular signalling carrier^3, 4^. Ca^2+^ signal (CS) or Ca^2+^ signature is the intracellular universal^5^ signal shaped by different Ca^2+^ channels when Ca^2+^ influx into the plant/animal cells^6^ in response to almost every aspect of information procesing, including enviromental stresses, developmental processes, and inter-organismal communications^7^. All CSs are converted^8^ and then relayed into the downstream universal protein phosphorylation responses (PPRs)^9^ to multiply the messages into the nucleus.

Classical biochemical and phylogenetic studies have identified seven gene families in plants and animals that convert CSs to PPRs, including (i) Ca^2+^-dependent protein kinase (CDPK), (ii & iii) calcineurin B-like protein (CBL) & CBL-interacting protein kinase (CIPK), (iv) Ca^2+^/calmodulin-dependent protein kinase (CCaMK)^10^, and (v, vi, vii) Ca^2+^/calmodulin-dependent kinase I, II, IV (CaMKI, II, IV)^11^. These seven families work by two different mechanisms^12^: one group of CDPK, binds Ca^2+^ by a calmodulin-like domain (CaM-LD) and converts CSs into PPRs via a kinase domain (KD) in the same protein; the rest group of genes require an independent calmodulin protein to bind Ca^2+^ first and then bind another kinase to convert CSs to PPRs^10, 11^. These drastic different biochemical mechanisms along with their diverse structures in these two groups of proteins raise an intriguing and fundamental question whether or not these two groups of genes originated dependently/independently. Current classifications of the gene families that contain both types of proteins remain highly ambiguous and controversy.

Hrabak and colleagues^13^ first established the CDPK-SnRK (sucrose non-fermenting1-related kinases) superfamily based on alignment of KDs that contain families CDPKs, CDPK-related kinases (CRKs), SnRKs, CCaMKs, CaMKIs, phospho*enol*pyruvate carboxylase kinases (PPCKs), and PPCK-related kinases (PEPRKs) from *Arabidopsis* and several other eukaryotic species. Interestingly, CaMKIs are absent in plant kinomes and only found in animals and fungi^14^, CDPKs are only found in plants and several protists (ciliates and apicomplexans)^15^, whereas CRKs, PPCKs, PEPRKs, and SnRK2s and SnRK3s (also termed as CIPKs) have been confirmed only in plants^13^. CCaMKs harbour a visin-like domain that is most likely an Animalia derived gene^16^. Furthermore, the superfamily members CRKs, PPCKs, and PEPRKs do not require Ca^2+^ for activation^17^, suggesting inconsistency between biochemical properties and these nomenclature. The highly heterogeneous protein structures in the CDPK-SnRK superfamily, Ca^2+^ dependence, signal relaying, and distribution in eukaryotes, strongly indicate that they may have different origins and evolutionary trajectories.

In another classification, Ca^2+^/calmodulin-dependent protein kinase (CAMK), a group name adopted from animal orthologs based on Ca^2+^/calmodulin binding property^8, 14^, covers most families in the CDPK-SnRK superfamily, strangely excludes the Ca^2+^-dependent mitogen-activated protein kinases (MAPK) and includes the Ca^2+^/calmodulin independent PPCK family^18^. This discrepancy also points to the strong likelihood that two types of CS-PPR converters included in either CDPK-SnRK superfamily or CAMK group may have independent origins and evolved with different trajectories.

Among all CS-PPR converter families, another basic question is that which family plays the leading role in each eukaryotic supergroup. In animals, CaMKI&II&IVs act as the main CS-PPR converter among all kinases^19, 20^. However, the main gene family of CS-PPR converter in plants remains unclear because of ambiguity of classification^21^. In addition, Ca^2+^ signal conversion mechanisms/genes in eukaryotes other than animals and plants remain poorly understood because of the lack of comparative analyses.

Here, we re-examined the phylogeny of the gene families that convert CSs into PPRs, specifically by including the deep phylogenetic clades of Glaucophyta, Rhodophyta, Charophyta, Pteridophyta, and gymnosperms, and other less-researched eukaryotic supergroups organisms. We identified three monophylies in all eukaryotic supergroups, and particularly, an Archaeplastida monophylic gene group independent from closely related CaMKI/II/IVs. We further traced the origin of this Archaeplastida gene monophyly to the fusion of a kinase gene and a calmodulin gene near the root of tree of plant life. We proposed to name this family as the *calmodulin fused kinase* (*CFK*) gene family. We further analysed evolutionary trajectory of *CFK* genes along with the tree of plant life in terms of numerical expansion, structural innovations and divergence, in relation to the six major evolutionary events in plant evolution from algae to flowering plants.

## Results

### A phylogenomic screen identified three eukaryotic gene monophylies of CS-PPR converters

**Figure 1:**
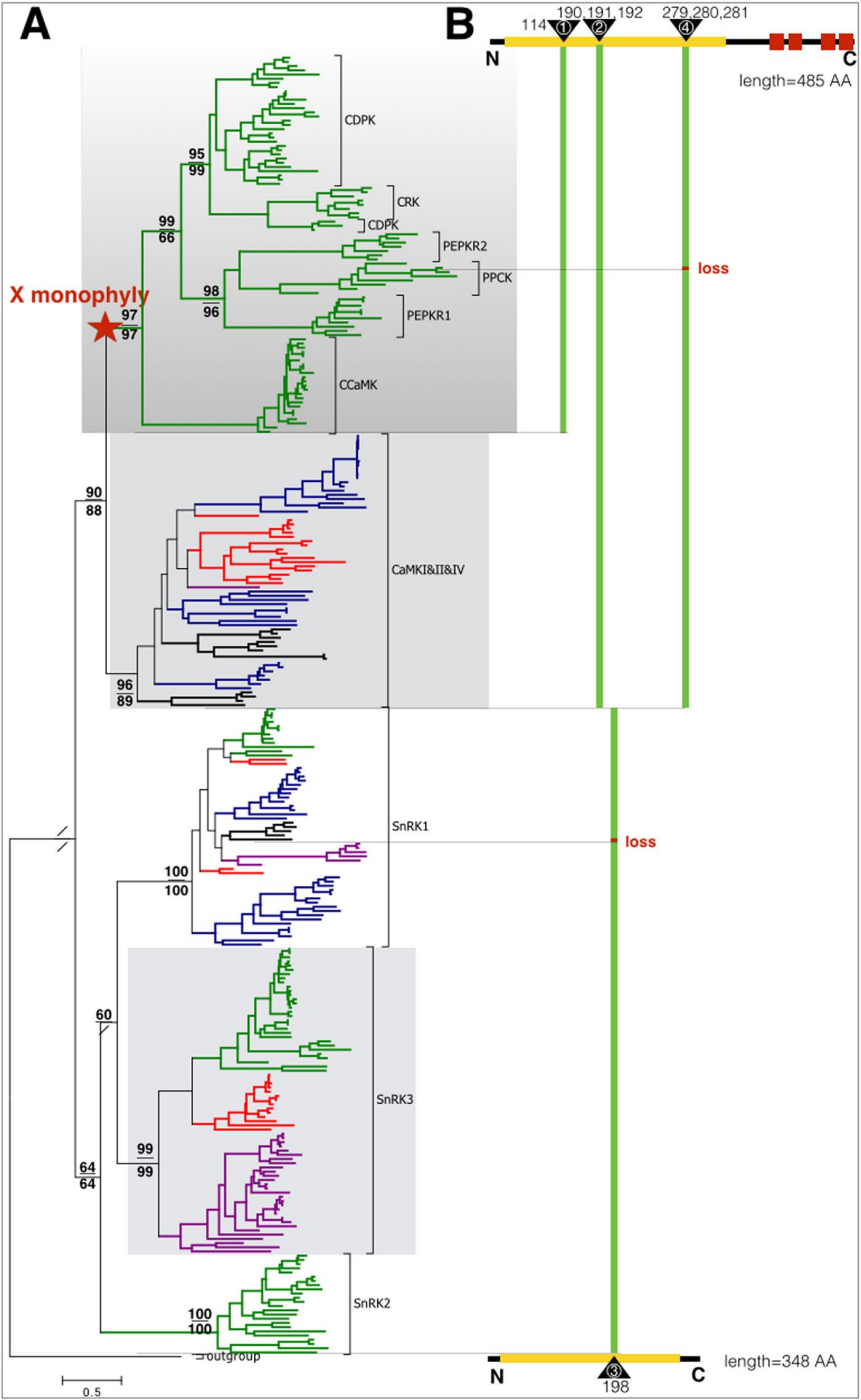
Three monophylic clusters identified among eukaryotic Ca^2+^ activated protein kinases. (**A**) The rooted tree displaying three monophylies of Ca^2+^ activated protein kinases among the eukaryotes. Mitogen-activated protein kinase (MAPK) was the outgroup. Major nodes were shown with two supporting values: maximum-likelihood by FastTree (upper number), randomized axelerated maximum likelihood methods (lower number). Branches are colored to represent different origins, Green: Archaeplastida, blue: Opisthokonta, red: SAR, black: Amoebozoa, purple: Excavata. (**B**) Schematic representation of monophylic cluster specific structural features with the schematic structures from the first and the last branch. Yellow block: kinase domain, red block: EF hand, green bar: monophyly specific inserted amino acid (s).

To explore the phylogeny of CS-PPR converter families, we performed phylogenetic inference using sequences of all Ca^2+^ activated kinases, covering the CDPK-SnRK superfamily, CAMK group, and MAPK (the complete tree is shown in Fig. S1). MAPK was the outgroup (Fig. 1A), consistent to the phylogenetic relationship in the kinome tree^22^. CDPKs, CRKs, CCaMKs, PPCKs, and PPCK-related kinases (PEPRKs) formed a well-supported monophyly with the high local supporting value 97 from FastTree’s near maximum likelihood with 1000 sampling, and bootstrap support value 97 from randomized axelerated maximum likelihood (RAxML) 1000 sampling, respectively (Fig. 1A). This monophyly had no existing name and we temporarily designated it as the X monophyly. CaMKI, II, IV families from various eukaryotes constituted the second monophyly with supporting values 96 and 89, and were grouped as the CaMKI &II &IV monophyly (Fig. 1A). SnRK3 (also known as CIPK) was a subfamily of SnRK group that had been experimentally validated as CS-PPR converters^23^. SnRK3s were found in eukaryotic supergroups Archaeplastida, SAR, and Excavata, and formed the third monophylic group with both high supporting values 99 (Fig. 1A). All SnRKs formed the outgroup of X monophyly and CaMKI&II&IV monophyly, since the X monophyly and CaMKI&II&IV monophyly were sister monophylies with supporting values 90 and 88 (Fig. 1A).

To determine whether or not three monophylies were reliable and whether they had independent or shared origins, we compared each monophyly members at the sequence level. Firstly, the X monophyly had an insertion signature (insertion 1) with one amino acid (AA) residue in the KD (Fig. 1B, the conserved feature of insertions in sequence logo was shown in Fig. S2). Secondly, except one PPCK from *Brassica napus*, members from the X monophyly and CaMKI&II&IVs lost the entire C-terminal domain, and had insertion signatures 2 and 4, each with three AAs located in the very conserved KD. Thirdly, SnRKs shared a conserved signature insertion 3 with one AA in the KD. The phylogenetic relations and the structural analyses clearly showed that the X monophyly was an independent monophyly, and had a common ancestor with the monophyly CaMKI&II&IV, whereas the SnRK3 was an independent monophyly.

### Suggested calmodulin fused kinase (CFK) gene family

Because the X monophyly covered several independent gene families with various domain structures, and distributed in different organisms, we then revealed whether or not the gene families covered in the X monophyly would share the same structured ancestor gene by analysing more samples, especially from those previously unavailable taxa, Glaucophyta, Rhodophyta, Charophyta, Pteridophyta, and gymnosperm of Archaeplastida, and the often-neglected protists (Table S1). We found that the X monophylic members not only distributed in plants, ciliates, and apicomplexans as previously reported^13^, but were also present in other eukaryotic supergroups, including Excavata, SAR Clade, Amoebozoa, and Opisthokonta (Fig. S3). However, X monophylic members in other eukaryotes nested within the plant X monophylic members and share high similarities with plant sequences. These suggested that the X monophylic members had first evolved in ancestral plants and these non-plant eukaryotes very likely obtained these X monophylic sequences via independent horizontal gene transfers (HGTs) in their ancestors (Fig. S4). We then focused on the origin and evo-devo analysis of the X monophyly in plants.

**Figure 2:**
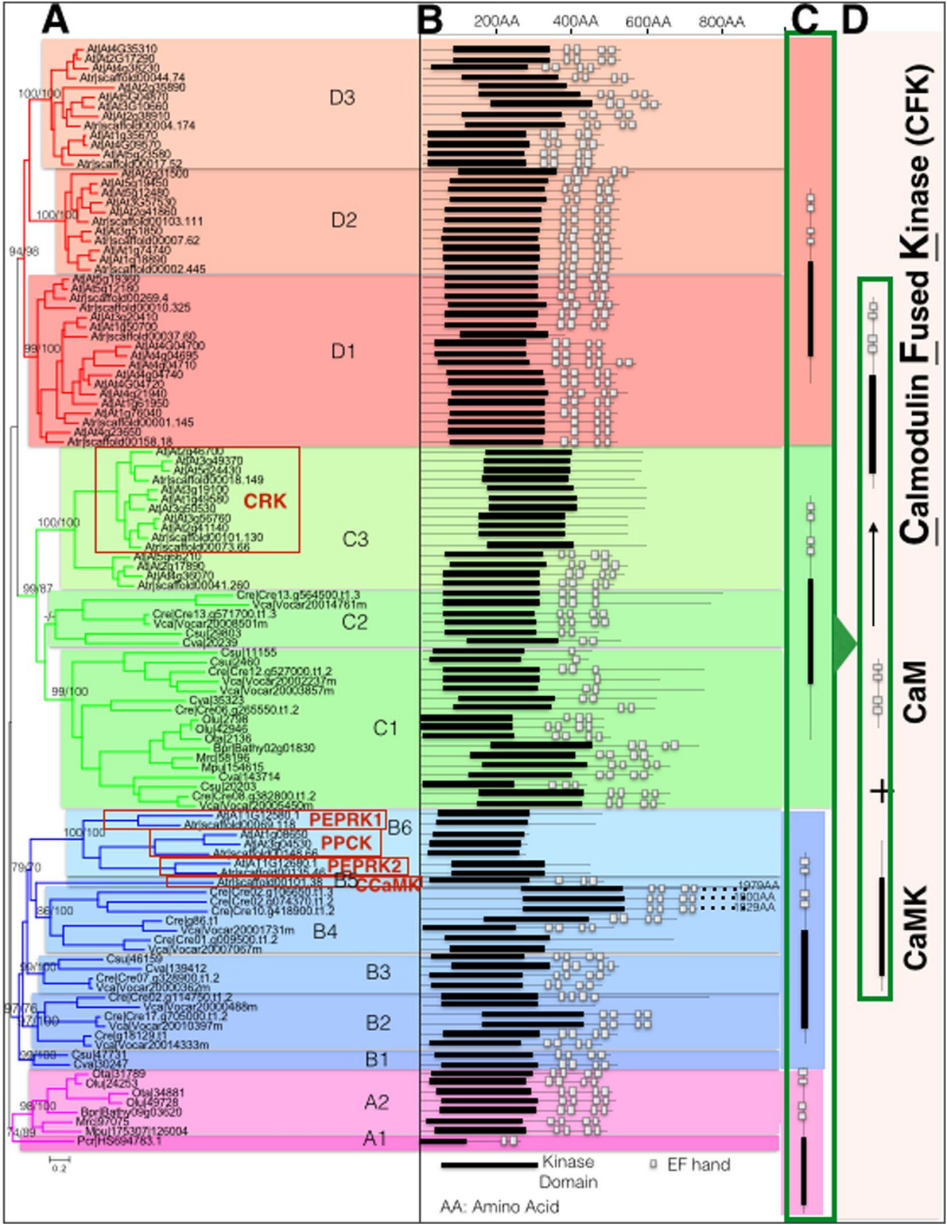
The X monophyly members originated from a single ancestor. (**A**) The X monophyly is divided into four subfamilies (**A–D**) and further subdivided into 14 groups according to the rooted tree using the subfamily A as the root group. (**B**) Protein structures of X monophylic members with kinase domain (solid black bar) and calmodulin-like domain (each small open square represents an EF hand). (**C**) Summary of diagrammatic structures of early branches in each subfamily, (**D**) the entire X monophyly originated from a presumed fusion of a kinase and a calmodulin.

After various trials, we constructed a phylogenetic tree using sequences representing all subfamilies and sub-subfamilies (or groups) from all algae, *Amborella trichopoda* and *Arabidopsis thaliana*. The final synthetic tree based on maximum-likelihood and Bayesian inference both yielded a topologically well-supported tree that was subdivided into four subfamilies (A, B, C, D), and further divided into 14 groups according to the tree topology (Fig. 2A). The CRK, a gene family previously recognized as a sister group to the CDPK in CDPK-SnRK superfamily^13^, now occupied only a proportion of C3 group (Fig. 2A). Structurally, all basal members of four subfamilies had both the KD and CaM-LD (Fig. 2B), only the crown members of the subfamily B6 group, which consisted of the PPCK and PEPRKs, lost the auto-inhibitory domain (AID) (the domain following the KD) and CaM-LD (Fig. 2B). The sub-group of the C3 group, designated as CRK before, lost the CaM-LD (Fig. 2B). Therefore, the ancestors of all four subfamilies in the X monophyly shared the same structured ancestor: a gene fused by a kinase gene and a calmodulin gene, the same as the CDPK^24^ (Fig. 2C). Since prokaryotes do not use Ca^2+^ as a signal, and no gene had been found in prokaryotes that contain both of these domains, the possible ancestral gene of all four subfamilies in the X monophyly was most likely originated through a *de novo* fusion of a kinase gene and a calmodulin gene. Based on this conserved common structure and the phylogenetic relationship (Fig. 1), we proposed to name this X monophyly the **C**almodulin **F**used **K**inase (CFK) gene family.

### Origin and expansion of CFK subfamilies in plants

From aquatic single-celled algae to terrestrial flowering plants, cell signaling networks evolved to become more and more complex and robust. Here we show that the CFKs have evolved from a single primitive ancestor gene into a large gene family in flowering plants. We categorized CFKs from the sampled plants representing all plant clades according to the four subfamilies and 14 groups (Fig. S5). We found glaucophytes contained no CFK and only one of the four sequenced red algae *Porphyridium cruentum* harbored one CFK gene. All of the Chlorophyta and Charophyta algae and land plant genomes contained CFKs, indicating that CFKs at least emerged about 1500 million year ago (MYA)^25^ in red algae.

**Figure 3:**
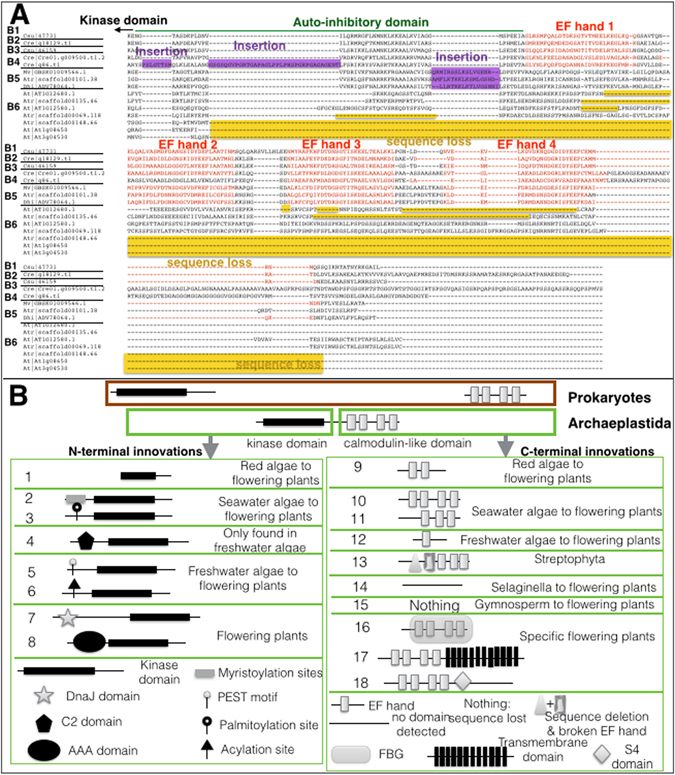
Structural innovations at the N-terminal and the C-terminal of calmodulin fused kinase proteins. (**A**) Domain changes in the C-terminal of subfamily B caused by sequence insertion (purple) and deletion (yellow). (**B**) The 18 representative structural innovations and modifications in both N- and C-terminal of CFKs.

In general, three major stages of gene expansion and loss occurred in plants (Table 1). The marine algae stage I had only two subfamilies, A (A1 and A2, these two groups existed only in stage I) and C (C1); the freshwater algae stage II had subfamilies B (B1, 2, 3, 4, these groups existed only in freshwater algae) and C2; and the streptophyta stage III had subfamilies B5, B6, C3, and D. Among all groups, subfamily A had maintained only 1–2 copies; B1, 2, 3, 4 each had kept 1–5 copies; B5 had kept 1 or 2 copies in plant genomes, but were lost in banana, *Aquilegia coerulea*, strawberry, and species in the Brassicales. A recent report stated that B5 CFKs (CCaMKs) originated in *Physcomitrella patens* ^26^, however, we found B5 CFKs emerged in *Mesostigma viride* (Fig. 3), the earliest branch of charophyta that arose about 725 mya^27^. The B6 subgroup retained its medium size, with an average 6.35 genes, and the C3 and D groups expanded dramatically during plant evolution (Table 1). Among the land plant lineage, the Brassicaceae had evolved the richest CFK family (Fig. S6A), more than the basal Brassicales plant papaya (*Carica papaya*) and grapevine (*Vitis vinifera*) (Fig. S6B). The expansions in the subfamilies B6, C, and D, resulted mostly through various forms of recent gene duplications (Fig. S6B)^28^.

**Table 1:** Numbers of CFKs in different species and subfamilies.

Species	Archaeplastida Lineage	A1	A2	B1	B2	B3	B4	B5	B6	C1	C2	C3	D1	D2	D3	Total	
37034 Glaucophyta nucleotide & EST sequences	Glaucophyta	0	0	0	0	0	0	0	0	0	0	0	0	0	0	0	
Cyanophora paradoxa	Glaucophyta	0	0	0	0	0	0	0	0	0	0	0	0	0	0	0	stage I
Cyanoptyche gloeocystis	Glaucophyta	0	0	0	0	0	0	0	0	0	0	0	0	0	0	0	
Gloeochaete wittrockiana	Glaucophyta	0	0	0	0	0	0	0	0	0	0	0	0	0	0	0	
Cyanidioschyzon merolae	Rhydophyta/Cyanidiophyceae	0	0	0	0	0	0	0	0	0	0	0	0	0	0	0	
*Calliarthron tuberculosum	Rhydophyta/Rhodophyceae	0	0	0	0	0	0	0	0	0	0	0	0	0	0	0	
Porphyra yezoensis	Rhydophyta/Rhodophyceae	0	0	0	0	0	0	0	0	0	0	0	0	0	0	0	
*Porphyridium cruentum	Rhydophyta/Porphyridiophyceae	1	0	0	0	0	0	0	0	0	0	0	0	0	0	1	
Micromonas pusilla CCMP1545	Chlorophyta/Prasinophyceae	0	1	0	0	0	0	0	0	1	0	0	0	0	0	2	
Micromonas pusilla RCC299	Chlorophyta/Prasinophyceae	0	1	0	0	0	0	0	0	1	0	0	0	0	0	2	
Ostreococcus lucimarinus	Chlorophyta/Prasinophyceae	0	2	0	0	0	0	0	0	2	0	0	0	0	0	4	
Ostreococcus tauri	Chlorophyta/Prasinophyceae	0	2	0	0	0	0	0	0	1	0	0	0	0	0	3	
Bathycoccus prasinos	Chlorophyta/Prasinophyceae	0	1	0	0	0	0	0	0	1	0	0	0	0	0	2	
Chlorella variabilis NC64A	Chlorophyta/Trebouxiophyceae	0	0	1	0	1	0	0	0	2	1	0	0	0	0	5	stage II
Volvox carteri	Chlorophyta/Chlorophyceae	0	0	0	3	1	2	0	0	3	2	0	0	0	0	11	
Chlamydomonas reinhardtii	Chlorophyta/Chlorophyceae	0	0	0	3	1	5	0	0	3	2	0	0	0	0	14	
Coccomyxa subellipsoidea	Chlorophyta/Chlorophyceae	0	0	1	0	1	0	0	0	3	1	0	0	0	0	6	
*Mesostigma viride	Charophyta/Mesostigmatophyceae	0	0	0	0	0	0	1	0	4	0	0	0	1	0	6	stage III
Klebsormidium flaccidum	Charophyta/Klebsormidiophyceae	0	0	0	0	0	0	0	0	0	0	9	1	0	2	12	
**Nothoceros aenigmaticus	Bryobiotina/Anthocerotophyta (hornwort)	NA	NA	NA	NA	NA	NA	1	NA	NA	NA	NA	NA	NA	NA	1	
**Phaeoceros laevis	Bryobiotina/Anthocerotophyta (hornwort)	NA	NA	NA	NA	NA	NA	1	NA	NA	NA	NA	NA	NA	NA	1	
**Marchantia polymorpha	Bryobiotina/Marchantiophyta(Liverwort)	NA	NA	NA	NA	NA	NA	NA	NA	NA	NA	NA	1	NA	NA	1	
**Dumortiera hirsuta	Bryobiotina/Marchantiophyta(Liverwort)	NA	NA	NA	NA	NA	NA	1	NA	NA	NA	NA	NA	NA	NA	1	
**Haplomitrium gibbsiae	Bryobiotina/Marchantiophyta(Liverwort)	NA	NA	NA	NA	NA	NA	1	NA	NA	NA	NA	NA	NA	NA	1	
**Pellia epiphylla	Bryobiotina/Marchantiophyta(Liverwort)	NA	NA	NA	NA	NA	NA	1	NA	NA	NA	NA	NA	NA	NA	1	
Physcomitrella patens	Bryobiotina/Bryophyta/(moss)/Bryopsida	0	0	0	0	0	0	2	0	0	0	8	7	6	10	33	
Selaginella moellendorffii	Lycopodiophyta	0	0	0	0	0	0	1	18	0	1	5	4	2	2	33	
**Ceratopteris richardii	Pteridophyta/Pteridopsida	NA	NA	NA	NA	NA	NA	1	NA	NA	NA	NA	NA	NA	NA	1	
*Anemia tomentosa	Pteridophyta/Polypodiopsida	0	0	0	0	0	0	0	0	0	0	8	2	4	3	18	
*Angiopteris evecta	Pteridophyta/Marattiopsida	0	0	0	0	0	0	1	4	0	0	4	2	2	1	14	
*Ginkgo biloba	gymnosperm/Ginkgophyta	0	0	0	0	0	0	1	5	0	0	5	4	1	3	19	
Picea abies	gymnosperm/Pinophyta	0	0	0	0	0	0	1	7	0	0	0	4	1	3	16	
Amborella trichopoda	Angiospermae/Amborella	0	0	0	0	0	0	1	3	0	0	4	5	3	3	19	
Phoenix dactylifera	Angiospermae/monocot/Arecales	0	0	0	0	0	0	1	3	0	0	7	6	8	6	31	
Musa acuminata	Angiospermae/monocot/Zingiberales	0	0	0	0	0	0	0	10	0	0	16	13	13	14	66	
Oryza sativa japonica	Angiospermae/monocot/Poales	0	0	0	0	0	0	1	4	0	0	5	8	8	11	37	
Aquilegia coerulea	Angiospermae/Magnoliophyta	0	0	0	0	0	0	0	4	0	0	5	4	4	6	23	
Vitis vinifera	Angiospermae/Vitales	0	0	0	0	0	0	1	5	0	0	7	5	5	6	29	
Arabidopsis lyrata	Angiospermae/Brassicales	0	0	0	0	0	0	0	4	0	0	11	14	8	11	48	
Arabidopsis thaliana	Angiospermae/Brassicales	0	0	0	0	0	0	0	4	0	0	11	13	8	10	46	
Brassica rapa	Angiospermae/Brassicales	0	0	0	0	0	0	0	8	0	0	19	16	16	19	78	
Capsella rubella	Angiospermae/Brassicales	0	0	0	0	0	0	0	4	0	0	14	14	8	10	50	
Carica papaya	Angiospermae/Brassicales	0	0	0	0	0	0	0	4	0	0	6	5	5	5	25	
Thellungiella halophila	Angiospermae/Brassicales	0	0	0	0	0	0	0	4	0	0	11	11	10	13	49	
Glycine max	Angiospermae/Fabales	0	0	0	0	0	0	2	9	0	0	15	17	12	17	72	
Fragaria vesca	Angiospermae/Rosales	0	0	0	0	0	0	0	6	0	0	5	5	4	6	26	
Cucumis sativus	Angiospermae/Cucurbitales	0	0	0	0	0	0	1	4	0	0	8	5	6	6	30	
Populus trichocarpa	Angiospermae/Malpighiales	0	0	0	0	0	0	2	10	0	0	11	8	9	11	51	
Mimulus guttatus	Angiospermae/Asterids/Lamiales	0	0	0	0	0	0	1	7	0	0	8	7	9	10	42	

“NA” stands for unknown number due to lacking of the genomes/transcriptomes. *Indicates partial genome or RNA-seq sequences available. **Indicates only limited sequences and without transcriptome sequences nor genomes.

### Structural innovations of the CFKs

In addition to variable sizes in CFK subfamilies, CFKs also evolved with dramatic structural diversification. Subfamily B members had the most versatile C-terminals (Fig. 2B). Basal branches (B1, 2, 3) had retained the complete set of KD, AID, and CaM-LD (Fig. 3A). Middle branches B4 and B5 had insertions in the AID (Fig. 3A). B5 lost the first EF hand although the sequence was still present, suggesting that the secondary or tertiary structure were destroyed due to sequence mutations (Fig. 3A). In the B6 group, the whole/partial loss of C-terminal had led to the loss of AID and CaM-LD, thereby PPCK and PEPRK were no longer activated by CSs (Fig. 3A).

Other domains, motifs, and modifications were found in the CFK family (Fig. 3B). The myristoylation and the palmitoylation modified AAs were first found in the basal seawater Chlorophyta algae and had been kept in the crown flowering plants (Fig. 3B). The PEST motif and acetylation AAs first appeared in CFK genes in the freshwater Chlorophyta algae (Fig. 3B). These motifs and modified AAs acted as the signal peptide for protein-protein, protein-lipid interactions, and membrane associations^29^, and for controlling the protein lifespan^30^. The presence of these modified AAs and motifs in the CFK genes strongly indicated that CFKs are potential key signal messengers in algae. Two CFK proteins from *Chlamydomonas reinhardtii* and *Volvox carteri* contained a C2 domain in the N-terminal, which was not believed to exist in CDPKs^31^. Some CFKs had a fused transmembrane domain-containing sequence (Fig. 3B, File S1), which might help to target the membrane for accurate and faster reception of CSs. Other new domains like FBG and S4 domains (Fig. 3B) were less researched in the Ca^2+^ signalling and future research is needed.

### Functional diversification evolution of CFKs for plant adaptation evolution

**Figure 4:**
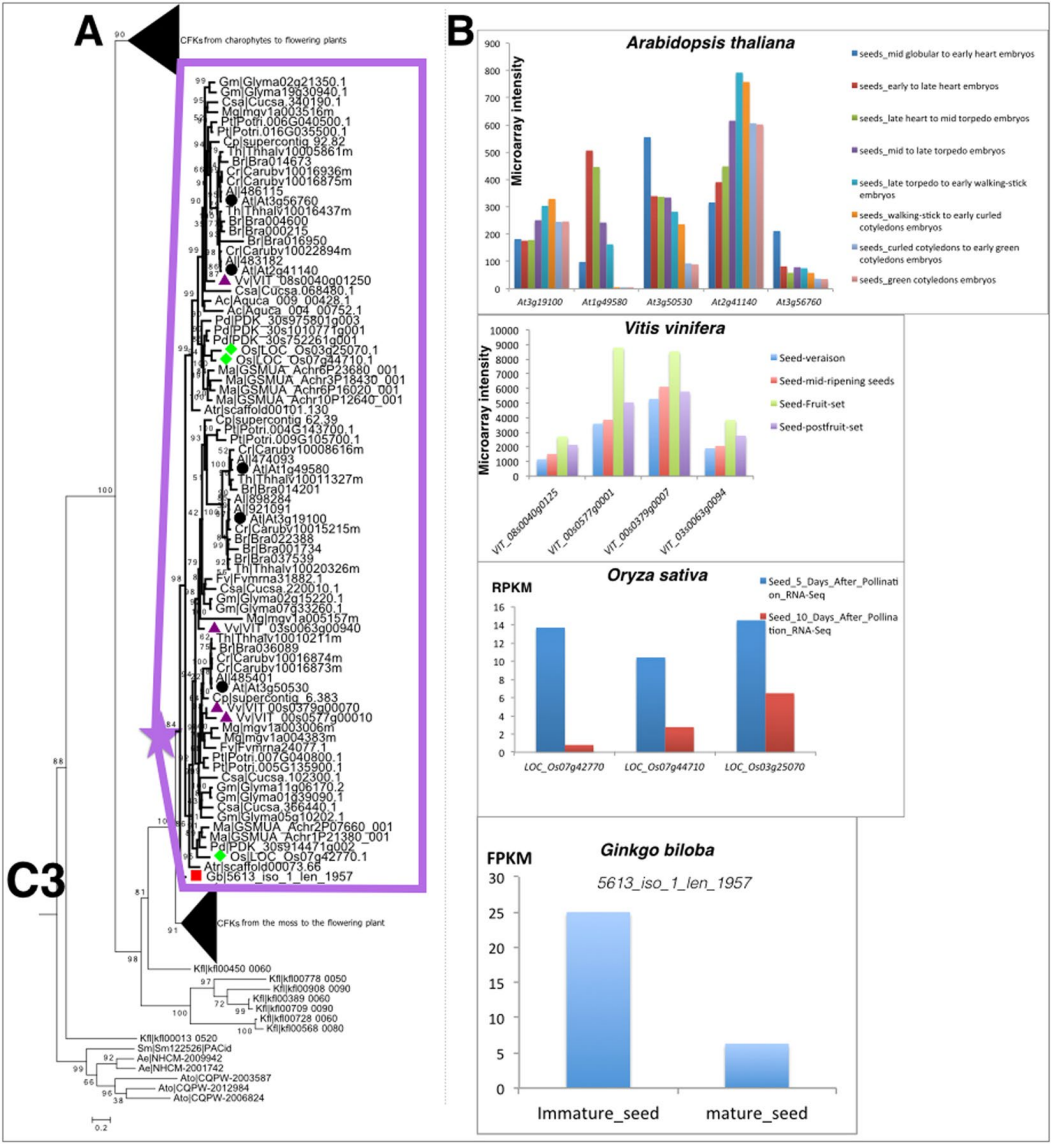
A cluster (marked with an asterisk) of seed plant specific C3 CFKs involved in seed maturation process. (**A**) The phylogenetic tree of a seed plant specific cluster from C3 group. (**B**) Expression profile of CFKs from seed plants *Arabidopsis thaliana*, *Vitis vinifera*, *Oryza sativa*, *Ginkgo biloba* are shown.

**Figure 5:**
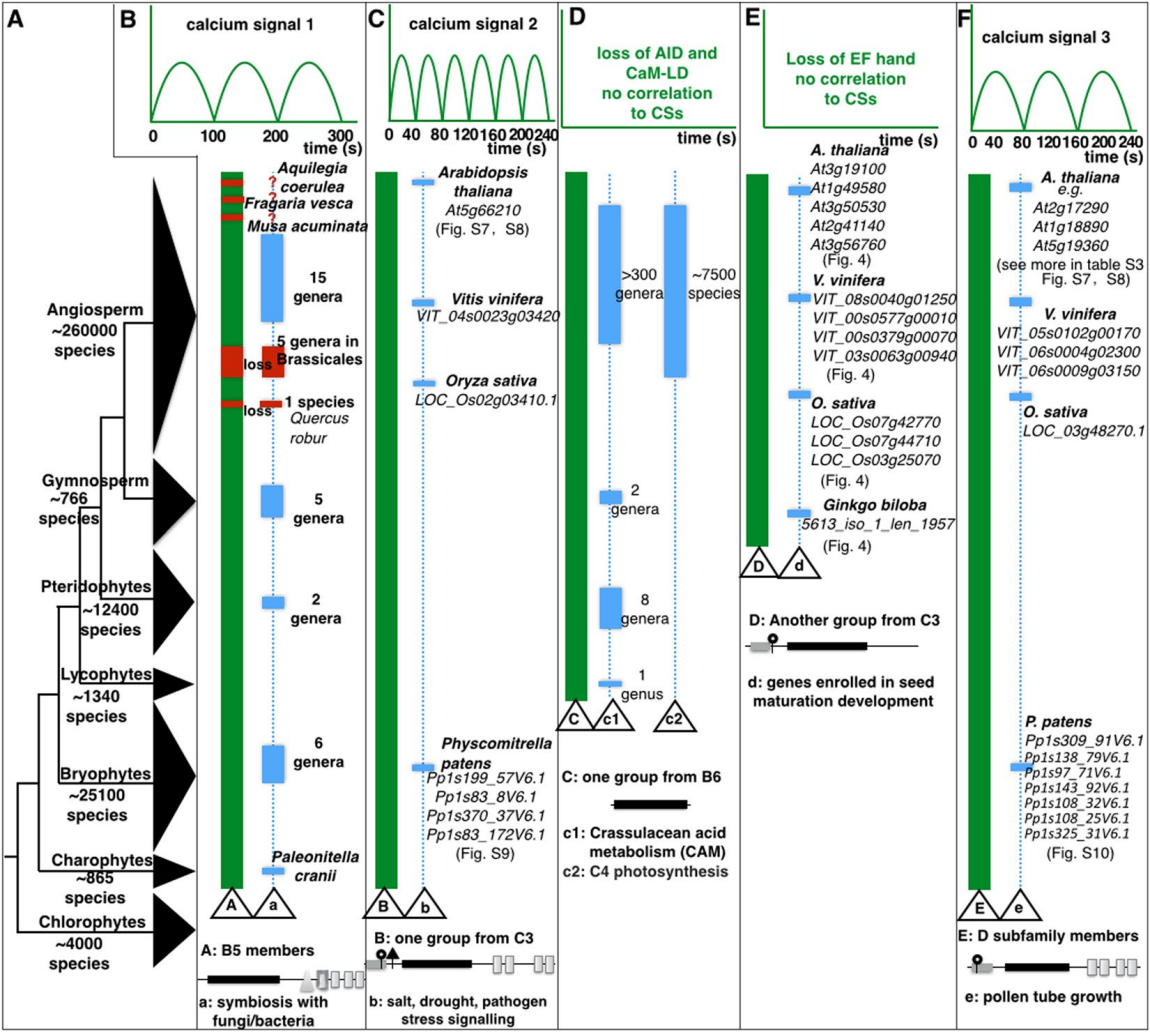
CFKs contribute to plant adaptation evolution. (**A**) Phylogeny and number of species among the lineages of green plants adapted from^54^. (**B**) The B5 CFKs receive low frequency CS and originated in charophytes, with functions dates back to charophyte alga-fungi symbiosis reported by^55^. (**C**) The high frequency CS that some B5 CFKs receive^36^. This group of genes shows myristoylation, palmitoylation, and acylation at the N-terminal and originated in charophyte, and act in the salt, drought, and pathogen stress signalling pathways. (**D**) The loss of auto-inhibitory domain (AID) and calmodulin-like domain (CaM-LD) of B6 genes caused these genes not activated by calcium. Both genes and crassulacean acid metabolism (CAM) originated in lycophytes whereas C4 photosynthesis was only found in angiosperms^56^. (**E**) This C3 subgroup of CFKs originated in the gymnosperm genomes. The loss of the EF hands also caused these genes not activated by calcium. Their roles in seed maturation development were found in seed plants. (**F**) The moderate frequency CS that some B5 CFKs receive. These genes have N-terminal myristoylation and palmitoylation and originated in charophytes. Their roles in male gamete maturation were confirmed in grapevine^38^, rice^57^, *A. thaliana* and *P. patens*. Capital letter in the triangle: gene group and its typical gene structure, small letter in the triangle: characterized gene functions. Solid vertical green bar: phylogenetic clade where genes are present; dotted vertical line indicates possible functions in these clades, and the solid blue bar in the dotted vertical line indicates species where gene functions have been validated.

To explore the contribution of CFKs to plant evolution, we analysed the evolution of CFKs in relation to their functions along the tree of plant life (Figs 4 and 5A). We focused on the subfamily B, C, D because there were no reports on subfamily A members due to its limited gene distribution in marine algae, nor any report on CFKs in Rhodophyta or seawater Chlorophyta algae.

In freshwater alga *C. reinhardtii*, subfamily B3 CFK *Cre07.g328900* and subfamily B4 CFK *Cre02.g074370* (Fig. 2A) function in the biogenesis of flagella and nutrition uptake^32^, respectively, indicating that CFKs played a role in plant freshwater adaptation. When plants transitioned from aquatic habitat to land environment, plants had to make substantial changes to adapt to the new environment. From *Mesostigma viride*, the earliest branch of Charophyta algae, streptophytes (covering charophytes and land plants) had evolved the 1^st^ EF hand-less B5 members (Fig. 3) that had the ability to decode the low frequency signal (100 s/cycle) (Fig. 5B) and played an indispensable role in arbuscular mycorrhizal (AM) fungi and rhizobia symbiosis^33^. Loss of B5 members in Brassicales and in *Quercus robur* disarmed plant-microbe rhizobia or plant-fungi associations^34^. Although putative functions of the orthologs of other B5 CFKs require future confirmation in plants, orthologs are generally assumed to retain equivalent functions in different organisms and to share other key properties^35^.

Terrestrial environment differs with aquatic environment mainly in dramatic changes in temperature, water, light, air, and soil environments. A group of C3 CFKs (Fig. 5C) evolved the N-terminal with myristoylation, palmitoylation, and acylation AAs, allowing these CFKs to associate with the plasma membrane for decoding the high frequency CSs (40 s/cycle)^36^. These genes were key members in plant drought, salt, and pathogen stress signalling as found in *A. thaliana* (Figs S7 and S8), *O. sativa* ^37^, *V. vinifera* ^38^, and *P. patens* (Fig. S9) by comparative transcription profiling (Fig. 5C).

One of the most interesting CFK groups is the B6 group that completely lost the CaM-LD. The earliest ortholog of PPCKs that enrolled in crassulacean acid metabolism (CAM)^39^ and C4 photosynthesis came from spikemoss *Selaginella* (Fig. 5D). They have lost the entire CaM-LD and are no longer activated by the CS probably when they were recruited to phosphorylate the PEPC, a signature enzyme of primary CO_2_ fixation in CAM^18^ and in C4 photosynthesis^40^.

CFKs had also contributed to the evolution of seed plants. Another cluster of C3 group genes had the myristoylation and palmitoylation sites, but lost all the typical EF hands in the C-terminal (Fig. 5E). This group CFKs originated in gymnosperms as this structured CFKs dated back to *Ginkgo biloba*. This seed plant lineage specific group displayed conserved expression pattern (rise first and then fall in the development of embryo) from primitive seed plant ginkgo to crown seed plant *A. thaliana* (Fig. 4), therefore, playing roles in seed maturation transition (Fig. 5E).

We found abundant CFKs, e g. 22 AtCFKs (Table S2) in the subfamily D act as key players in the male gamete development (Fig. 5F). These CFKs receive the moderate frequency CSs for pollen tube growth^36^. The orthologs of the D group CFKs originated in charophytes (Fig. 5F). These CFKs shared a conserved feature with both myristoylation and palmitoylation sites at the N-terminal. All these CFKs were experimentally validated or predicted to localize to the plasma membrane, These CFKs were found to be enrolled in pollen development in *A. thaliana*, *V. vinifera*, *O. sativa* (Figs S7 and S8), and gamete development in *P. patens* (Fig. S10).

## Discussions

### Eukaryotic cells evolved with three systems in converting CSs to PPRs and each eukaryotic group uses more than one system

Since previously designated superfamily CDPK-SnRK is clustered based on sequence similarity^13^ and CAMK group is clustered based on biochemical properties of Ca^2+^ binding^14^, each classification includes several structurally heterogeneous kinase gene families. These classifications are inconsistent and controversial with known discoveries as discussed in the introduction section, hence we questioned that these gene families might evolve independently. Since the CDPK genes exist in plants and in some protists, and all eukaryotic groups utilize the CS-PPR system, it is unknown whether or not the CS-PPR genes share a common eukaryotic origin. In this study, we characterized all CS-PPR converter families in all eukaryotic supergroups and classified them into three monophylic groups, the CaMKI&II&IV family, the SnRK3 subfamily, and the newly proposed CFK family, based on the evolutionary common origin.

Eukarya is now divided into five supergroups including Archaeplastida, Excavata, SAR clade, Amoebozoa, and Opisthokonta^41^. The Excavata were found to contain CFK system and SnRK3 system, and Excavata CFKs possibly were originated from plants CFKs through horizontal gene transfer. According to the gene family size, SnRK3s seem to be the major CS-PPR conversion system in Excavata. The SAR clade contains all three systems, however, the SnRK3s have not been reported with any functions. The Amoebozoa and Opisthokonta both had a few members of CFKs and the main CS-PPR conversion system would be CaMKI&II&IVs. Archaeplastida contain CFK system and SnRK3 system, each has multiple functional reports^26^. Overall, all the five eukaryotic supergroups have more than one CS-PPR conversion system, although they prefer to rely on one specific system.

### Plant evolved CFKs and utilized them as the major CS-PPR converter

There are three major ambiguous and controversial questions regarding plant CS-PPR decoding gene families: (1) what are the major gene families that decode CS to PPR, (2) since they work in two different biochemical mechanisms, whether these gene families originated dependently or independently, (3) what are their relationships with those in other eukaryotic supergroups and whether they may share a broader common origin. In this report, we clarified the previously controversial classifications of the CS-PPR converting gene families and we united them into a CFK gene family. Unlike former nomenclature based on biochemical properties like Ca^2+^ binding, we named these gene families based on their origins. The CFK gene family was most likely originated from a single ancestor gene, presumably by a *de novo* fusion of a CaMK kinase and a calmodulin in the deep root of Rhodophytes.

The CFK family, now also including CCaMKs and CRKs, competes with SnRK3 subfamily for the leading CS-PPR converter family in plants. Several lines of evidence, however, suggest that CFKs are the major CS-PPR converter in plants. First, CFKs expanded into more family members in flowering plants than SnRK3s did (e.g., 37 OsCFKs and 46 AtCFKs compared to 30 OsSnRK3s and 25 AtSnRK3s^23^). Second, CFKs have more comprehensive functions than SnRK3s have, including in plant basic metabolism (such as C4 and CAM photosynthesis), development, stress signalling, and plant-microbe interactions. SnRK3s are mostly involved in stress-related signalling as revealed in genome-wide expressional analyses both in eudicot Arabidopsis and monocot rice^12, 23^. Third, CFKs presumably are more efficient than SnRK3s in converting CSs to PPRs because CFK requires a single protein to sense CS and activate the downstream transcription factors, whereas SnRKs need two transcription factors to activate two genes, a kinase and a calmodulin, and two transcriptional machines to transcribe two genes, therefore, requires more ATPs and more time for molecular docking of two genes; Fourth, CFKs have versatile N-terminal and C-terminal domains and modifications for multi-subcellular localizations, whereas SnRK3s do not contain recognizable localization signals, and thereby most SnRK3s localized to the cytoplasm and nucleoplasm^23^.

### CFKs play pivotal roles in evolution of plant clade adaptation

Plant evolution includes the following six watershed events: colonization of land, plant-microbe co-evolution, development of life cycles, changes in morphology, advancement of photosynthesis, and the secondary metabolism^42–44^. CFKs have contributed to success of each of these major evolutionary events. In conquering terrestrial environment, one group of C3 CFKs, originated from charophytes, plays key roles in abiotic and biotic stress signalling. In plant-microbe interactions, B5 CFKs are irreplaceable in plant-fungi and plant-rhizobia interactions. In the aspect of life cycles, we have shown that a C3 group CFKs with both myristoylation and palmitoylation contributed to the seed maturation program. In the evolution of morphology, the expanded subfamily D CFKs are also key regulators in pollen tube growth and male gamete development in early land plants, so it is highly possible that this group had roles in developing anisogamy in charophytes since the two stages gametophyte and sporophyte are externally indistinguishable in chlorophytes (en.wikipedia.org/wiki/Gametophyte). Furthermore, B6 members that lost the C-terminal, thereby no longer respond to CSs, were recruited to C4 and CAM photosynthesis. The subfamily D members have also been reported to be involved in secondary metabolism^45^. In summary, origin and evolution of diverse CFK subfamilies have played key roles in all the major evolutionary processes during the plant adaptation evolution from algae to extant higher plants.

## Methods

### Sampling and dataset

Sampled species covered five supergroups of eukaryotes, bacteria and archaea. Sequences were retrieved from publically available sequenced genomes and RNA-seq transcriptomes as supplemented with details in Table S1. Plant samples covered all Archaeplastida clades, including Glaucophyta, Rhodophyta, Chlorophyta, Charophyta, Marchantiophyta, Bryophyta, Anthocerotophyta, Lycopodiophyta, Pteridophyta, gymnosperm and angiosperm. Transcriptome data of *A. thaliana*, *O. sativa*, *P. patens*, *G. biloba* were obtained with information listed in Table S1.

### Sequence alignment and phylogenetic tree construction

Multiple sequences alignment for Fig. 1 tree construction was performed with mafft program^46^ and for all other trees with muscle program^47^. The seed of each monophyly were generated using representative monophyly members as a robust seed used to search against protein databases with HMMER3 program^48^. All hits were examined with phylogenetic analysis and only sequences within the monophyly were used. Seeds were built using the curated results and were searched again to detect any sequences that were not found in the first round. A new round of search was performed using the new seed according to the last result until no new CFK kinase was found. Blast hits were also employed for curation of sequences^49^. Maximum likelihood method with wag protein substitution model were applied in FastTree^50^ and RAxML^51^, each with 1000 sampling, and the RAxML method was performed via online platform (www.phylo.org). Bayes tree was constructed by Mrbayes^52^ with 200000 samplings and samplefreq is 100.

### Structural analyses

For domain prediction, sequences were compared with both NCBI/CDD (www.ncbi.nlm.nih.gov/Structure/cdd/wrpsb.cgi) and smart (smart.embl-heidelberg.de) databases with relaxed e-value as 1. Sequence logo was drawn via web logo tool (weblogo.berkeley.edu/logo.cgi). N-terminal modification sites were predicted: myristoylation (mendel.imp.ac.at/myristate/SUPLpredictor.htm), palmitoylation (csspalm.biocuckoo.org/online.php), acylation (bdmpail.biocuckoo.org/prediction.php). PEST motif was predicted by epestfind (emboss.bioinformatics.nl/cgi-bin/emboss/epestfind). For the prediction of Myristoylation, the false positive prediction was strictly limited to be lower than e-0. For the rest predictions, the threshold was set as the default parameters.

### Gene expression analyses

Arabidopsis gene expressional data were analysed online (weigelworld.org). Rice gene expression data were retrieved from genome annotation project (rice.plantbiology.msu.edu/expression.shtml), and grapevine seed transcription data were normalized from microarray data according to previous report^53^. RPKM/FPKM (Reads/Fragments Per Kilobase of exon model Million mapped reads) were calculated for RNA-seq data. Protein subcellular localization was predicted by WoLF PSORT (www.genscript.com/wolf-psort.html). Student t-test for two-dataset comparison was based on SPSS Statistics (www.spss.co.in).

## Electronic supplementary material

Supplementary file
