# Supplementary material for: The calmodulin fused kinase novel gene family is the major system in plants converting Ca2+ signals to protein phosphorylation responses
Source: Sci Rep. 2017 Jun 23;7:4127. doi: 10.1038/s41598-017-03367-8 (PMC5482843; doi:10.1038/s41598-017-03367-8)
Supplement: Supplementary file 1 — Supplementary file [file 41598_2017_3367_MOESM1_ESM.pdf]

[Supplementary Information]

The calmodulin fused kinase novel gene family is the major system in plants converting Ca^{2+} signals to protein phosphorylation responses

Fei Chen[1,2,3], Liangsheng Zhang[3], Zong-Ming (Max) Cheng[1,2*]

[1]College of Horticulture, Nanjing Agricultural University, Nanjing, 210095, China

[2]Department of Plant Sciences, University of Tennessee, Knoxville, TN 37996, USA

[3]Center for Genomics and Biotechnology, HIST, Fujian Agriculture and Forestry University, Fuzhou 350002, China

*Correspondence: Zong-Ming (Max) Cheng, E-mail: zmc@njau.edu.cn ; zcheng@utk.edu.

Supporting Information

Table S1. Samples used in this study.

Species	Code	Common Name	No. of chromosomes/genome s	Lineage	Source	Version	Link	Note
1) Archaeplastida								
37034 Glaucophyta nucleotides				Glaucophyta	NCBI			
Cyanophora paradoxa	Cpa	NA	NA/70/NA	Glaucophyta	Cyanophora genome project	1	http://cyanophora.rutgers.edu/cyanophora/	Genome available
Porphyridium cruentum	Pcr	NA	NA/NA/NA	Rhodophyta/Porphyridiophyceae/Porphyridiales/Porphyridiaceae		1	http://dbdata.rutgers.edu/data/plantae/	Genome not available
Chondrus crispus	Ccr	Irish moss	NA/~105Mb/NA	Rhodophyta/Florideophyceae	EBI	1	http://phylomedb.org/phylome_96	Genome available
Calliarthron tuberculosum	Ctu	NA	NA/NA/NA	Rhodophyta/Rhodophyceae/Nemaliophycidae/Corallinales/Corallinaceae			http://dbdata.rutgers.edu/data/plantae/	partial genome
Pyropia yezoensis	Pye	Zicai or Open Sea Nori		Rhodophyta/Bangiophyceae	http://nrifs.fra.affrc.go.jp	1	http://nrifs.fra.affrc.go.jp/ResearchCenter/5_AG/genomes/nori/	Genome available
Cyanidioschyzon merolae	Cme	NA	20/16.5/NA	Rhodophyta/Cyanidiophyceae/Cyanidiales/Cyanidiaceae	Cyanidioschyzon merolae Genome Project	1	http://merolae.biol.s.u-tokyo.ac.jp/download/	Genome available
Micromonas pusilla CCMP1545	Mpu	NA	19/21.9/NA	Chlorophyta/Prasinophyceae	JGI	3	http://genome.jgi.doe.gov/pages/dynamicOrganismDownload.jsf?organism=MicpuC3	Genome available
Micromonas pusilla RCC299	Mrc	NA	17/20.9/NA	Chlorophyta/Prasinophyceae	JGI	3	http://genome.jgi-psf.org/MicpuN3/MicpuN3.download.ftp.html	Genome available
Ostreococcus lucimarinus	Olu	NA	21/13.2/NA	Chlorophyta/Prasinophyceae	JGI	3	http://genome.jgi-psf.org/Ost9901_3/Ost9901_3.download.ftp.html	Genome available
Ostreococcus tauri	Ota	NA	20/12.6/NA	Chlorophyta/Prasinophyceae	JGI	3	http://genome.jgi-psf.org/Ostta4/Ostta4.download.ftp.html	Genome available
Bathycoccus prasinos	Bpr	NA	19/15/NA	Chlorophyta/Prasinophyceae	ugent-PSB	1	https://bioinformatics.psb.ugent.be/gdb/bathycoccus/RELEASE_15jul2011/	Genome available
Chlorella variabilis NC64A	Cva	NA	12/46.2Mb/>97% genes	Chlorophyta/Trebouxiophyceae	JGI	1	http://genome.jgi-psf.org/ChlNC64A_1/ChlNC64A_1.download.ftp.html	Genome available
Volvox carteri	Vca	fierce roller	14/138/98% mRNAs	Chlorophyta/Chlorophyceae	JGI	1	http://genome.jgi.doe.gov/Volca1/Volca1.download.ftp.html	Genome available
Chlamydomonas reinhardtii	Cre	NA	17/121/78% genome aligned	Chlorophyta/Chlorophyceae	phytozome9.0	5.3	ftp://ftp.jgi-psf.org/pub/compgen/phytozome/v9.0/Creinhardtii/annotation/	Genome available
Coccomyxa subellipsoidea	Csu	NA	20/48.8/NA	Chlorophyta/Chlorophyceae	JGI	2	http://genome.jgi.doe.gov/Coc_C169_1/Coc_C169_1.download.ftp.html	Genome available
Mesostigma viride	Mv	NA	NA	Charophyta/Mesostigmatophyceae	NCBI	1	http://www.ncbi.nlm.nih.gov/nuccore?LinkName=bioproject_nuccore&from_uid=242253	Genome not available
Klebsormidium flaccidum	Kfl	NA	NA/~104Mb/NA	Charophyta/Klebsormidiophyceae	http://genome.microbedb.jp	1	http://genome.microbedb.jp/klebsormidium/nies-2285	Genome available
Nothoceros aenigmaticus	Nae	NA	NA	Bryobiotina/Anthocerotophyta (hornwort)	NCBI	1	http://www.ncbi.nlm.nih.gov/protein/ADV78071.1	Genome not available
Phaeoceros laevis	Pla	NA	NA	Bryobiotina/Anthocerotophyta (hornwort)	NCBI	1	http://www.ncbi.nlm.nih.gov/protein/ADV78070.1	Genome not available
Marchantia polymorpha	Mpo	NA	NA	Bryobiotina/Marchantiophyta	NCBI	1	http://www.ncbi.nlm.nih.gov/protein/BAA81749.1	Genome not available
Dumortiera hirsuta	Dhi	NA	NA	Bryobiotina/Marchantiophyta(Liverwort)	NCBI	1	http://www.ncbi.nlm.nih.gov/protein/ADV78064.1	Genome not available
Haplomitrium gibbsiae	Hgi	NA	NA	Bryobiotina/Marchantiophyta(Liverwort)	NCBI	1	http://www.ncbi.nlm.nih.gov/protein/ADV78062.1	Genome not available
Pellia epiphylla	Pep	NA	NA	Bryobiotina/Marchantiophyta(Liverwort)	NCBI	1	http://www.ncbi.nlm.nih.gov/protein/ADV78065.1	Genome not available
Physcomitrella patens	Ppa	NA	27/480/NA	Bryobiotina/Bryophyta/moss	phytozome9.0	1	ftp://ftp.jgi-psf.org/pub/compgen/phytozome/v9.0/Ppatens/annotation/	Genome available
Selaginella moellendorffii	Sm	NA	NA/110Mb/NA	Lycopodiophyta	phytozome9.0	1	ftp://ftp.jgi-psf.org/pub/compgen/phytozome/v9.0/Smoellendorffii/annotation/	Genome available
Ceratopteris richardii	Cri	NA	NA	Pteridophyta/Pteridopsida	NCBI	1	http://www.ncbi.nlm.nih.gov/protein/AAN11310.1	Genome not available
Anemia tomentosa	Ato	NA	NA	Pteridophyta/Polypodiopsida	onekp	1	https://www.bioinfodata.org/Blast4OneKP/	Genome not available
Angiopteris evecta	Ae	NA	NA	Pteridophyta/Marattiopsida	onekp	1	https://www.bioinfodata.org/Blast4OneKP/	Genome not available
Ginkgo biloba	Gb	ginkgo	NA	gymnosperm/Ginkgoopsida	MSU/MPGR	1	ftp://ftp.plantbiology.msu.edu/pub/data/MPGR/Ginkgo_biloba/	partial genome data
Picea abies	Pa	Norway spruce	24/19.6Gb/96%	gymnosperm/Pinophyta	Spruce Genome Project	1	http://congenie.org/start	Genome available
Amborella trichopoda	Atr	Amborella	13/870Mb/NA	agnoliophyta(Angiospermae)/Amborella	Amborella genome database	3	ftp://amborella-project.huck.psu.edu/Public/Amborella/annotation/EVM_27_third_release_2012.08.03/	
Phoenix dactylifera	Pd	Date Palm	NA/380Mb/NA	agnoliophyta(Angiospermae)/monocot/Arecales	Angiospermae/monocot/Arecales	3	http://qatar-weill.cornell.edu/research/datepalmGenome/download.html	Genome available
Musa acuminata	Ma	banana	NA/472.2Mb/NA	agnoliophyta(Angiospermae)/monocot/Zingiberales	banana genome database	1	http://banana-genome.cirad.fr/download.php	Genome available
Setaria italica	Si	Foxtail millet	NA/405.7Mb/-	agnoliophyta(Angiospermae)/monocot/Poales	phytozome9.0	9	ftp://ftp.jgi-psf.org/pub/compgen/phytozome/v9.0/Sitalica/annotation/	Genome available
Oryza sativa japonica	Osj	rice nipponbare	24/372Mb/NA	agnoliophyta(Angiospermae)/monocot/Poales	phytozome9.0	9	ftp://ftp.jgi-psf.org/pub/compgen/phytozome/v9.0/Osativa/annotation/	Genome available
Oryza sativa indica	Osi	rice indica variety	NA/466Mb/NA	agnoliophyta(Angiospermae)/monocot/Poales	BGI	NA	http://rice.genomics.org.cn/rice/link/download.jsp	Genome available
Aquilegia coerulea	Ac	Colorado blue columbine	NA/302Mb/NA	agnoliophyta(Angiospermae)/Magnoliophyta/Magnoliopsida	phytozome9.0	9	ftp://ftp.jgi-psf.org/pub/compgen/phytozome/v9.0/Acoerulea/annotation/	Genome available
Vitis vinifera	Vv	grapevine	NA/487Mb/NA	agnoliophyta(Angiospermae)/Vitales	CRIBI	1	http://genomes.cribi.unipd.it/DATA/V1/FASTA/	Genome available
Arabidopsis lyrata	Al	NA	NA/207/NA	agnoliophyta(Angiospermae)/Brassicales	phytozome9.0	9	ftp://ftp.jgi-psf.org/pub/compgen/phytozome/v9.0/Alyrata/annotation/	Genome available
Arabidopsis thaliana	At	Mouse-ear cress	5/140.1/NA	agnoliophyta(Angiospermae)/Brassicales	phytozome9.0	9	ftp://ftp.jgi-psf.org/pub/compgen/phytozome/v9.0/Athaliana/annotation/	Genome available
Brassica rapa	Br	Chinese Cabbage	NA/485 M/NA	agnoliophyta(Angiospermae)/Brassicales	phytozome9.0	9	ftp://ftp.jgi-psf.org/pub/compgen/phytozome/v9.0/Brapa/annotation/	Genome available
Capsella rubella	Cr	Capsella	NA/134.8Mb/NA	agnoliophyta(Angiospermae)/Brassicales	phytozome9.0	9	ftp://ftp.jgi-psf.org/pub/compgen/phytozome/v9.0/Crubella/annotation/	Genome available
Carica papaya	Cp	papaya	9/372/NA	agnoliophyta(Angiospermae)/Brassicales	phytozome9.0	9	ftp://ftp.jgi-psf.org/pub/compgen/phytozome/v9.0/Cpapaya/annotation/	Genome available
Thellungiella halophila	Th	NA	NA/243.1Mb/NA	agnoliophyta(Angiospermae)/Brassicales	phytozome9.0	9	ftp://ftp.jgi-psf.org/pub/compgen/phytozome/v9.0/Thalophila/annotation/	Genome available
Glycine max	Gm	soybean	NA/975Mb/NA	agnoliophyta(Angiospermae)/Fabales	phytozome9.0	9	ftp://ftp.jgi-psf.org/pub/compgen/phytozome/v9.0/Gmax/annotation/	Genome available
Phaseolus vulgaris	Pv	common bean	NA/521/1/NA	agnoliophyta(Angiospermae)/Eudicots/RosidsFabales	phytozome9.0	9	ftp://ftp.jgi-psf.org/pub/compgen/phytozome/v9.0/Pvulgaris/annotation/	Genome available
Lotus japonicus	Lj	wild legume	NA/315.1/NA	agnoliophyta(Angiospermae)/Eudicots/Rosids/Fabales	miyakogusa.jp	NA	http://www.kazusa.or.jp/lotus/	Genome available
Medicago truncatula	Mt	barrelclover	NA/500Mb/NA	agnoliophyta(Angiospermae)/Eudicots/RosidsFabales	phytozome9.0	9	ftp://ftp.jgi-psf.org/pub/compgen/phytozome/v9.0/Mtruncatula/annotation/	Genome available
Cajanus cajan	Cc	pigeon pea	NA/833Mb/NA	agnoliophyta(Angiospermae)/Eudicots/RosidsFabales	Gigadb	5	http://gigadb.org/dataset/100028	Genome available
Malus domestica	Md	apple	NA/881.3Mb/NA	agnoliophyta(Angiospermae)/Eudicots/RosidsRosales	phytozome9.0	9	ftp://ftp.jgi-psf.org/pub/compgen/phytozome/v9.0/Mdomestica/annotation/	Genome available
Fragaria vesca	Fv	strawberry	NA/240Mb/NA	agnoliophyta(Angiospermae)/Rosales	phytozome9.0	9	ftp://ftp.jgi-psf.org/pub/compgen/phytozome/v9.0/Fvesca/annotation/	Genome available
Cucumis sativus	Csa	Cucumber	NA/203Mb/NA	agnoliophyta(Angiospermae)/Cucurbitales	phytozome9.0	9	ftp://ftp.jgi-psf.org/pub/compgen/phytozome/v9.0/Csativus/annotation/	Genome available
Populus trichocarpa	Pt	poplar	NA/422.9Mb/NA	agnoliophyta(Angiospermae)/Malpighiales	phytozome9.0	9	ftp://ftp.jgi-psf.org/pub/compgen/phytozome/v9.0/Ptrichocarpa/annotation/	Genome available
Jatropha curcas Palawan	Jc	Barbados nut	NA/410Mb/NA	agnoliophyta(Angiospermae)/Eudicots/Rosids/Malpighiales/Euphorbiaceae	kazusa	4.5	http://www.kazusa.or.jp/jatropha/	Genome available
Ricinus communis	Rc	castorbean	NA/400Mb/NA	agnoliophyta(Angiospermae)/Eudicots/Rosids/Malpighiales	phytozome9.0	9	ftp://ftp.jgi-psf.org/pub/compgen/phytozome/v9.0/Rcommunis/annotation/	Genome available
Manihot esculenta	Me	Cassava	NA/533Mb/NA	agnoliophyta(Angiospermae)/Eudicots/Rosids/Malpighiales/Euphorbiaceae	phytozome9.0	9	ftp://ftp.jgi-psf.org/pub/compgen/phytozome/v9.0/Mesculenta/annotation/	Genome available
Mimulus guttatus	Mg	monkey flower	NA/321.7Mb/NA	agnoliophyta(Angiospermae)/Asterids/Lamiales	phytozome9.0	1.1	ftp://ftp.jgi-psf.org/pub/compgen/phytozome/v9.0/Mguttatus/annotation/	Genome available
Bigelowiella natans	Bn	NA	NA/94.7Mb/96.5%	Rhizaria	JGI	1	http://genome.jgi.doe.gov/Bigna1/Bigna1.home.html	Genome available
Prunus mume	Pm	mei	16/280M/NA	agnoliophyta(Angiospermae)/Rosales	Prunus mume Genome Project		http://prunusmumegenome.bjfu.edu.cn/	Genome available
Quercus robur	Qr	oak	24/1.5Gb/NA	agnoliophyta(Angiospermae)/Rosids/Fagales	URGI	1	https://urgi.versailles.inra.fr/blast/	Genome available
Gossypium raimondii	Gr	cotton	NA/761.4Mb/NA	agnoliophyta(Angiospermae)/Eudicots/Rosids/Malvales	phytozome9.0	9	ftp://ftp.jgi-psf.org/pub/compgen/phytozome/v9.0/Ptrichocarpa/annotation/	Genome available
Theobroma cacao	Tc	cacao tree	NA/346Mb/NA	agnoliophyta(Angiospermae)/Eudicots/Rosids/Malvales	phytozome9.0	9	ftp://ftp.jgi-psf.org/pub/compgen/phytozome/v9.0/Tcacao/annotation/	Genome available
Citrullus lanatus	Cl	watermelon	NA/425Mb/NA	agnoliophyta(Angiospermae)/Eudicots/Rosids/Cucurbitales	icugi	1	ftp://www.icugi.org/pub/genome/watermelon/v1/	Genome available
Eucalyptus grandis	Eg	flooded gum	NA/691Mb/NA	agnoliophyta(Angiospermae)/Eudicots/RosidsMyrtales	phytozome9.0	9	ftp://ftp.jgi-psf.org/pub/compgen/phytozome/v9.0/Egrandis/annotation/	Genome available
Solanum lycopersicum	Sl	tomato	NA/900Mb/NA	agnoliophyta(Angiospermae)/Eudicots/Asterids/Solanales	solgenomics	2.3	ftp://ftp.solgenomics.net/tomato_genome/annotation/ITAG2.3_release/	Genome available
Solanum tuberosum	St	potato	NA/800Mb/NA	agnoliophyta(Angiospermae)/Eudicots/Asterids/Solanales	solgenomics	3.4	http://solgenomics.net/organism/Solanum_tuberosum/genome	Genome available
2) Amoebozoa								
Dictyostelium discoideum	NA	NA	NA	Amoebozoa/Conosa	dictybase	1	http://www.dictybase.org/Downloads/	Genome available
Dictyostelium fasciculatum	NA	NA	NA	Amoebozoa/Conosa	dictybase	1	http://genomes.dictybase.org/fasciculatum	Genome available
Polysphondylium pallidum	NA	NA	NA	Amoebozoa/Mycetozoa	dictybase	1	http://genomes.dictybase.org/pallidum	Genome available
Amoebozoa nucleotides	NA	NA	NA	Amoebozoa	NABI	NA	http://www.ncbi.nlm.nih.gov/nuccore/?term=amoebozoa%5Borganism%5D	Found 727019 nucleotide
3) Opisthokonta								
Monosiga brevicollis	NA	NA	NA/41.6Mb/NA	Opisthokonts/choanozoa	JGI	current	http://genome.jgi-psf.org/pages/dynamicOrganismDownload.jsf?organism=choanozoa	Genome available
Candida albicans	NA	NA	NA/8.4Mb/NA	Fungi/Ascomycota	JGI	22	http://www.candidagenome.org/download/sequence/C_albicans_SC5314/	Genome available
Candida tropicalis	NA	NA	NA/4.2Mb/NA	Fungi/Ascomycota	JGI	current	http://www.candidagenome.org/download/sequence/C_tropicalis_MYA-3404/	Genome available
Debaryomyces hansenii	NA	NA	NA/3.6Mb/NA	Fungi/Ascomycota	JGI	current	http://www.candidagenome.org/download/sequence/D_hansenii_CBS767/	Genome available
Lodderomyces elongisporus	NA	NA	NA/4.5Mb/NA	Fungi/Ascomycota	JGI	current	http://www.candidagenome.org/download/sequence/L_elongisporus_NRLL_YB-4239/	Genome available
Ganoderma lucidum	NA	Lingzhi mushroom	NA/12.5Mb/NA	Fungi/Basidiomycota/Agaricomycetes/Polyporales/Ganodermataceae/Ganoderm	Ganoderma lucidum database		http://www.herbalgenomics.org/galu/	Genome available
Ashbya gossypii	NA	NA	NA	Fungi/Ascomycota	JGI	current	http://img.jgi.doe.gov/cgi-bin/w/main.cgi?section=TaxonDetail&page=taxonDetail&taxon_oid=2521172682	Genome available
Fungi nucleotides	NA	NA	NA	Fungi	NCBI	NA	http://www.ncbi.nlm.nih.gov/nuccore/?term=fungi%5Borganism%5D	Found 9515224 nucleotid
Metazoa nucleotides	NA	NA	NA	Metazoa	NCBI	NA	http://www.ncbi.nlm.nih.gov/nuccore/?term=metazoa%5Borganism%5D	Found 162599849 nucleot
choanozoa nucleotides	NA	NA	NA	choanozoa	NCBI	NA	http://www.ncbi.nlm.nih.gov/nuccore/?term=choanozoa%5Borganism%5D	Found 189064 nucleotide
4) Excavata								
Leishmania major	NA	NA	NA/32.8Mb/NA	Excavata/Euglenozoa/Kinetoplastida	sanger institute	NA	ftp://ftp.sanger.ac.uk/pub/pathogens/Leishmania/major/DATASETS/	Genome available
Trypanosoma brucei	NA	NA	NA/35Mb/NA	Excavata/Euglenozoa/Kinetoplastida	sanger institute	4	ftp://ftp.sanger.ac.uk/pub/pathogens/Trypanosoma/brucei/T.brucei_genome_v4/	Genome available
Giardia intestinalis	NA	NA	NA/11Mb/NA	Excavata/Metamonada	GiardiaDB	current	http://giardiadb.org/common/downloads/release-2.5/GintestinalisAssemblageB/fasta/	Genome available
5) Rhizaria								
Bigelowiella natans	NA	NA	NA/94.7Mb/NA	Rhizaria/chlorarachniophyte	JGI	1	http://genome.jgi.doe.gov/Bigna1/Bigna1.home.html	Genome available
Reticulomyxa filosa	NA	NA	NA/30.6Mb/NA	Rhizaria/Foraminifera	NCBI	1	http://www.ncbi.nlm.nih.gov/Traces/wgs/?val=ASPP01#	Genome available
6) Chromalveolata								
Babesia bovis	NA	NA	NA/7.9Mb/NA	Chromalveolata/Alveolata/Apicomplexa	piroplasmadb	1.1	http://piroplasmadb.org/common/downloads/release-1.1/BbovisT2Bo/fasta/	Genome available
Cryptosporidium hominis	NA	NA	NA/8.6Mb/NA	Chromalveolata/Alveolata/Apicomplexa	cryptodb	4.6	http://cryptodb.org/common/downloads/release-4.6/Chominis/fasta/	Genome available
Ectocarpus siliculosus	NA	NA	NA/14.9Mb/NA	Chromalveolata/Heterokontophyta or stramenopiles		2	https://bioinformatics.psb.ugent.be/gdb/ectocarpus/	Genome available
Phytophthora sojae	NA	NA	NA/95Mb/NA	Chromalveolata/Heterokontophyta or stramenopiles/Oomycota			http://vmd.vbi.vt.edu/download/index.php	Genome available
Bacteria	NA	NA	NA	Bacteria	NCBI	NA	http://www.ncbi.nlm.nih.gov/nuccore/?term=bacteria%5Borganism%5D	Found 22832432 nucleoti
Archaea	NA	NA	NA	Archaea	NCBI	NA	http://www.ncbi.nlm.nih.gov/nuccore/?term=archaea%5Borganism%5D	Found 494532 nucleotide
Transcriptome data	origin							
Arabidopsis thaliana	weigelworld.org							
Orysa sativa	rice.plantbiology.msu.edu/expression.shtml							
Vitis vinifera	http://www.ncbi.nlm.nih.gov/geo/query/acc.cgi?acc=GSE36128							
Physcomitrella patens	http://www.ncbi.nlm.nih.gov/sra?term=SRP050162							
	supplementary file in http://www.sciencedirect.com/science/article/pii/S1674205215004554							
Ginkgo biloba	ftp://ftp.plantbiology.msu.edu/pub/data/MPGR/Ginkgo_biloba/							
Medicago truncatula	http://mtgea.noble.org/v3/experiments.php							

Table S2. CFKs from *Arabidopsis thaliana* enrolled in pollen development from D subfamily.

pollen development	myristoylation	palmytoylation	group
At2g17290	y	y	d3
At2g38910	y	y	d3
At3G10660	y	y	d3
At2g35890	y	y	d3
At5G04870	y	y	d3
At4G35310	y	y	d3
At1g18890	y	y	d2
At1g74740	y	y	d2
At2g41860	y	y	d2
At3g57530	y	y	d2
At5g19450	y	y	d2
At5g12480	y	y	d2
At3g51850	y	y	d2
At2g31500	y	y	d2
At5g19360	y	y	d1
At5g12180	y	y	d1
At4g23650	y	y	d1
At3g20410	y	y	d1
At4g04740	y	y	d1
At4g21940	y	y	d1
At4g04710	y	y	d1
At4G04700	y	y	d1

Figure S1 The details of the phylogenetic tree in Figure 1. The maximum-likelihood local supporting value was produced by FastTree software. Branches are colored to represent the origins from the five eukaryotic supergroups. Green: Archaeplastida, Blue: Opisthokonta, Red: SAR, Black: Amoebozoa, Purple: Excavata.

Figure S2 The four characteristic insertions in the kinase domain are represented as sequence logo format.

Insertion 1 and 3 each has one amino acid, insertion 2 and 4 each has three amino acids.

Figure S3 Distribution of X-monophylic members in eukaryotic supergroups.

Figure S4 X-monophylic members in Eukaryotes other than plants are possibly derived from plant X-monophylic members via horizontal gene transfers.

The plant X-monophylic members are shown in green, and the non-plant X-monophylic members are shown in red. The arrow indicates the possible horizontal gene transfer events.

Figure S5 The whole maximum-likelihood phylogenetic tree of CFK genes generated with CFKs from all sampled plants.

Abbreviations of species are shown in Table S1.

Figure S6 Enrichment and expansion of CFKs in the order Brassicales.

(A) The genome density of CFKs amongst green plant representatives. (B) Expansion of CFKs via various forms, and rounds of duplication.

Figure S7 Expression profile of CFKs in *Arabidopsis thaliana* and a B5 member in *Medicago truncatula*.

Expression profile of AtCFKs includes developmental stages of 8 organs/parts, cold, osmotic stress, salt, drought, genotoxic, oxidative, UV-B, wounding, and heat treatment, pseudomonas, phytophthora, and elicitors treatments. Expression profile of *M. truncatula* CFK includes different organs and developmental stages.

Figure S8 Functions of CFKs in *Arabidopsis thaliana*.

Domains, motifs, and modifications along with corresponding subcellular localizations are shown. Functions on three aspects (development, abiotic and biotic Stress, Plant-microbe communication) are based on expressional analyses as in Fig. S7 and previous reports (Boudsocq & Sheen, 2013). * indicates that it was reported by Boudsocq and Sheen (Boudsocq & Sheen, 2013). #$ represents reports by Aldous et al. (Aldous, 2014) and Yang et al. (Yang, 2015), respectively.

Figure S9 Expression profile of four CFKs from P. patens under the treatments of salt and drought stresses, respectively.

Figure S10 The expressional profiles of subfamily D CFKs from Physcomitrella patens between the sporophyte stage (mean_spores) and the gametophyte stage (mean_gametophore).

Details of the samples and the experiment could be found by Khraiwesh et al. 2015 (Khraiwesh, 2015).

File S1. Representative sequences that have innovative gene structure compared to the typical structure composed of a KD and a CaM-LD.

References

Aldous S. **2014**. Evolution of the phosphoenolpyruvate carboxylase protein kinase family in C3 and C4 Flaveria spp. *Plant Physiol* **165**: 1076–1091.

Boudsocq M, Sheen J. **2013**. CDPKs in immune and stress signaling. *Trends Plant Sci* **18**: 30–40.

Khraiwesh B. **2015**. Genome-wide expression analysis offers new insights into the origin and evolution of Physcomitrella patens stress response. *Sci Rep* **5**: 17434.

Yang X. **2015**. A roadmap for research on crassulacean acid metabolism (CAM) to enhance sustainable food and bioenergy production in a hotter, drier world. *New Phytol* **207**: 491–504.

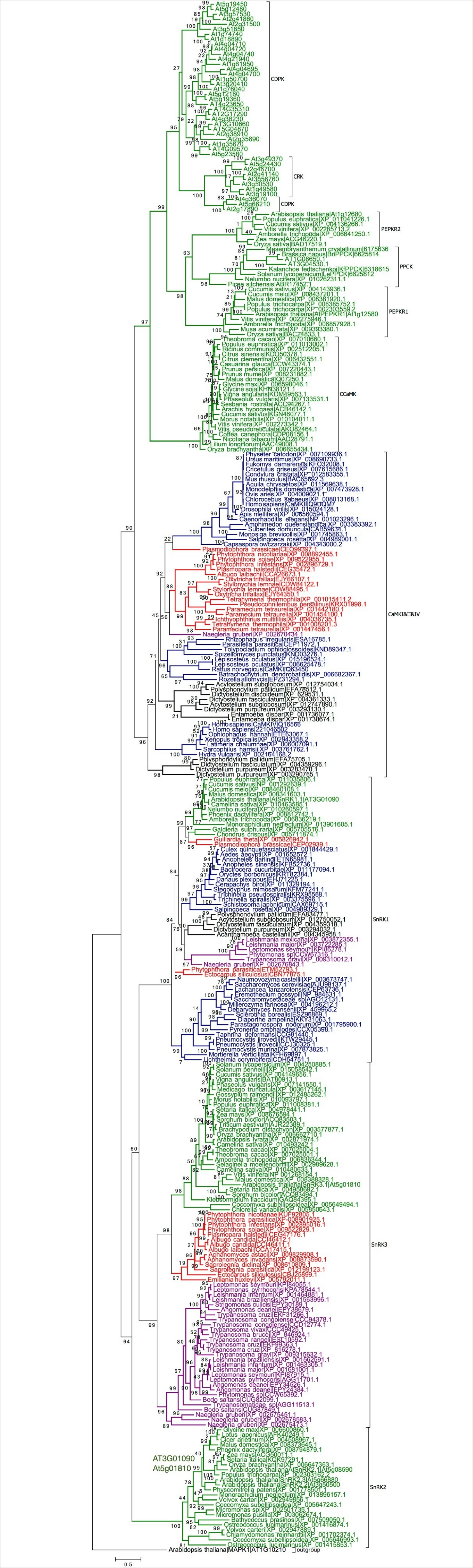
CDPK
CRK
CDPK
PEPKR2
PPCK
PEPKR1
CCaMK
CaMKI&II&IV
SnRK1
SnRK3
AT3G01090
At5g01810
SnRK2
Arabidopsis thaliana|MAPK1|AT1G10210
outgroup
0.5

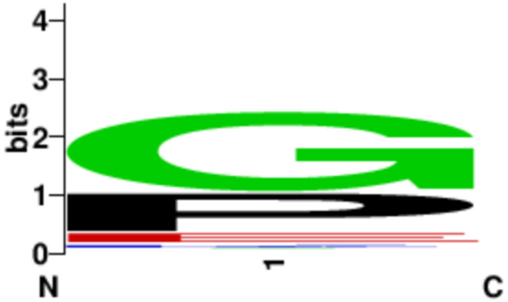

insertion 1

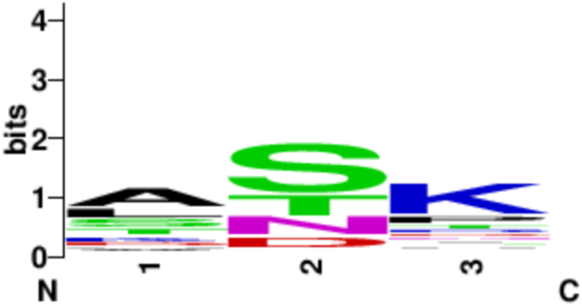

insertion 2

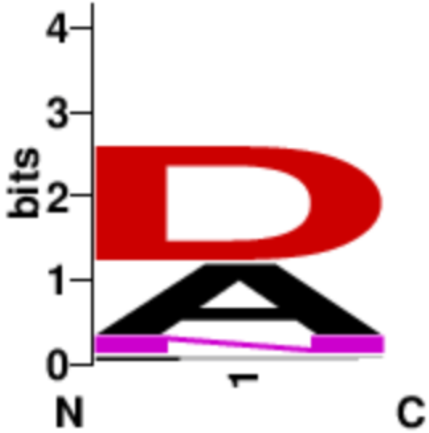

insertion 3

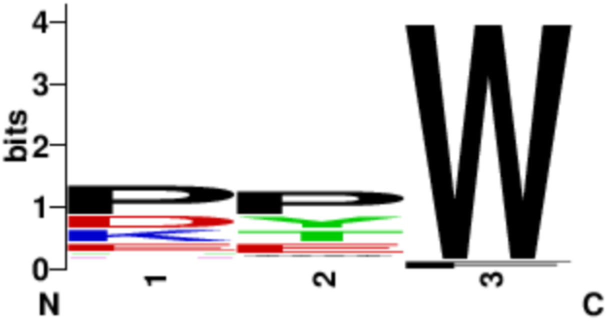

insertion 4

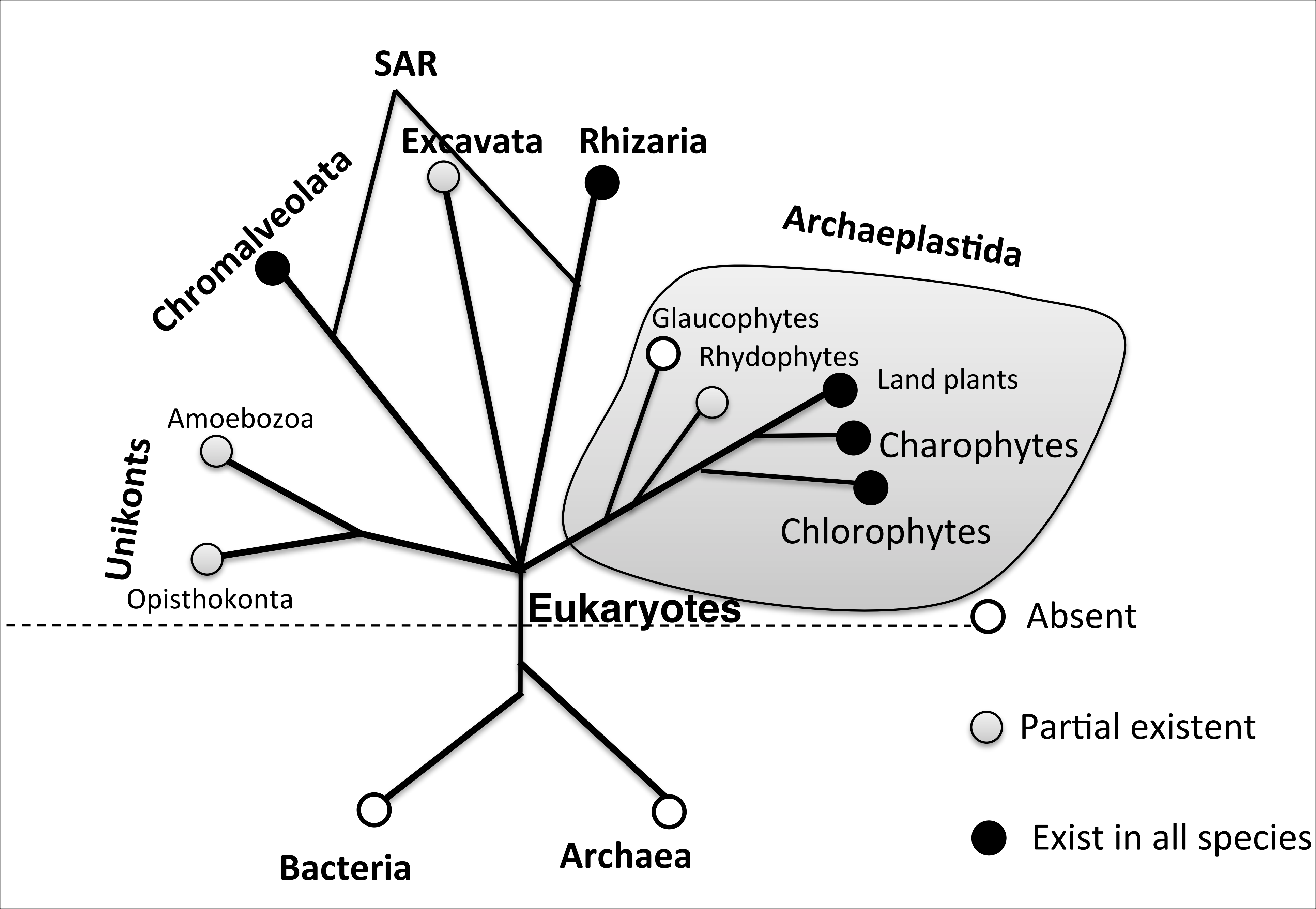

SAR
Excavata
Rhizaria
Chromalveolata
Archaeplastida
Glaucophytes
Rhydophytes
Land plants
Charophytes
Chlorophytes
Amoebozoa
Unikonts
Opisthokonta
Eukaryotes
Absent
Partial existent
Exist in all species
Bacteria
Archaea

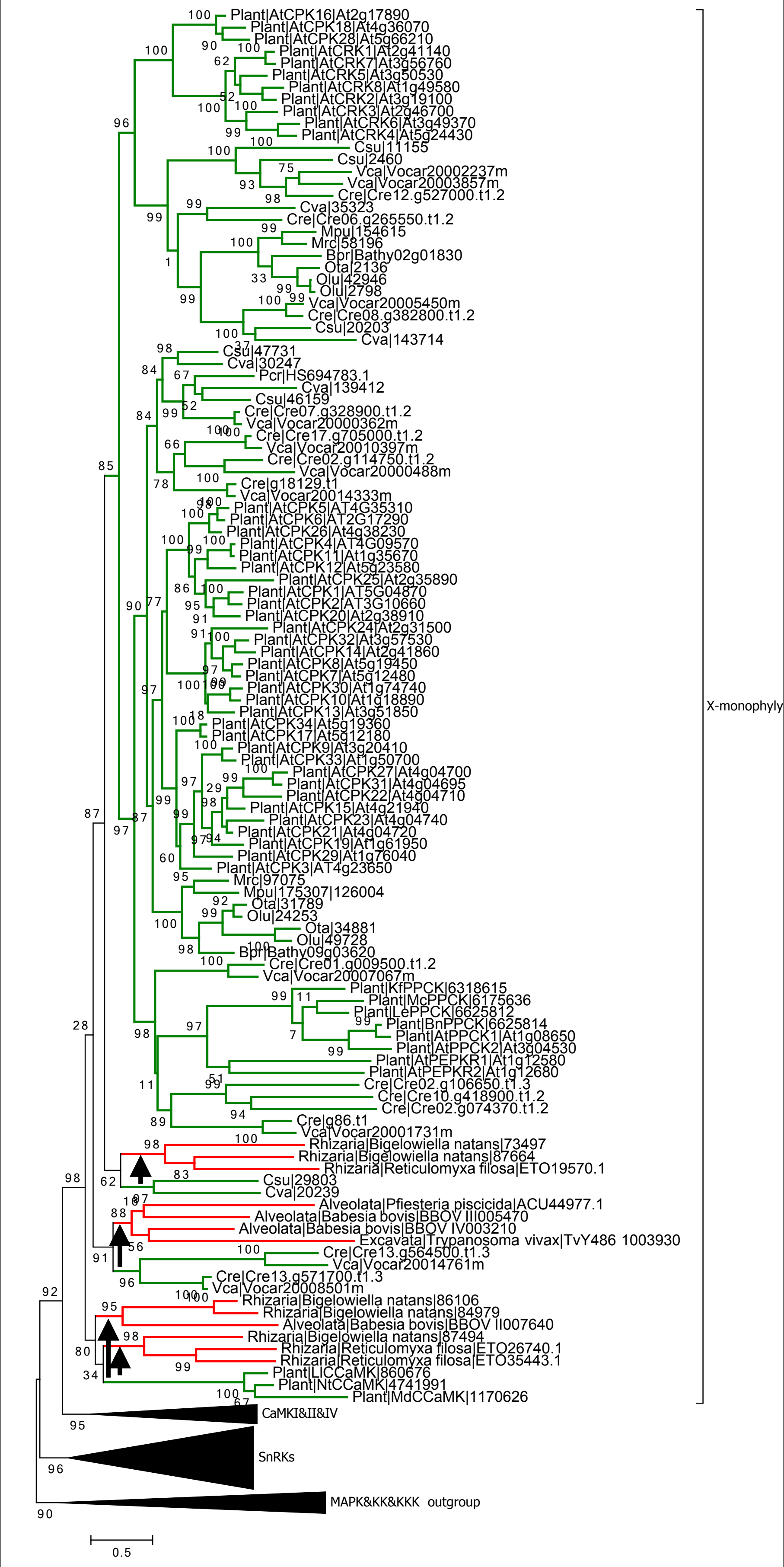
Plant|AtCPK16|At2g17890
Plant|AtCPK18|At4g36070
Plant|AtCPK28|At5g66210
Plant|AtCRK1|At2g41140
Plant|AtCRK7|At3g56760
Plant|AtCRK5|At3g50530
Plant|AtCRK8|At1g49580
Plant|AtCRK2|At3g19100
Plant|AtCRK3|At2g46700
Plant|AtCRK6|At3g49370
Plant|AtCRK4|At5g24430
Csu|11155
Csu|2460
Vca|Vocar20002237m
Vca|Vocar20003857m
Cre|Cre12.g527000.t1.2
Cva|35323
Cre|Cre06.g265550.t1.2
Mpu|154615
Mrc|58196
Bpr|Bathy02g01830
Ota|2136
Olu|42946
Olu|2798
Vca|Vocar20005450m
Cre|Cre08.g382800.t1.2
Csu|20203
Cva|143714
Csu|47731
Cva|30247
Pcr|HS694783.1
Cva|139412
Csu|46159
Cre|Cre07.g328900.t1.2
Vca|Vocar20000362m
Cre|Cre17.g705000.t1.2
Vca|Vocar20010397m
Cre|Cre02.g114750.t1.2
Vca|Vocar20000488m
Cre|g18129.t1
Vca|Vocar20014333m
Plant|AtCPK5|AT4G35310
Plant|AtCPK6|AT2G17290
Plant|AtCPK26|At4g38230
Plant|AtCPK4|AT4G09570
Plant|AtCPK11|At1g35670
Plant|AtCPK12|At5g23580
Plant|AtCPK25|At2g35890
Plant|AtCPK1|AT5G04870
Plant|AtCPK2|AT3G10660
Plant|AtCPK20|At2g38910
Plant|AtCPK24|At2g31500
Plant|AtCPK32|At3g57530
Plant|AtCPK14|At2g41860
Plant|AtCPK8|At5g19450
Plant|AtCPK7|At5g12480
Plant|AtCPK30|At1g74740
Plant|AtCPK10|At1g18890
Plant|AtCPK13|At3g51850
Plant|AtCPK34|At5g19360
Plant|AtCPK17|At5g12180
Plant|AtCPK9|At3g20410
Plant|AtCPK33|At1g50700
Plant|AtCPK27|At4g04700
Plant|AtCPK31|At4g04695
Plant|AtCPK22|At4g04710
Plant|AtCPK15|At4g21940
Plant|AtCPK23|At4g04740
Plant|AtCPK21|At4g04720
Plant|AtCPK19|At1g61950
Plant|AtCPK29|At1g76040
Plant|AtCPK3|AT4g23650
Mrc|97075
Mpu|175307|126004
Ota|31789
Olu|24253
Ota|34881
Olu|49728
Bpr|Bathy09g03620
Cre|Cre01.g009500.t1.2
Vca|Vocar20007067m
Plant|KfPPCK|6318615
Plant|McPPCK|6175636
Plant|LePPCK|6625812
Plant|BnPPCK|6625814
Plant|AtPPCK1|At1g08650
Plant|AtPPCK2|At3g04530
Plant|AtPEPKR1|At1g12580
Plant|AtPEPKR2|At1g12680
Cre|Cre02.g106650.t1.3
Cre|Cre10.g418900.t1.2
Cre|Cre02.g074370.t1.2
Cre|g86.t1
Vca|Vocar20001731m
Rhizaria|Bigelowiella natans|73497
Rhizaria|Bigelowiella natans|87664
Rhizaria|Reticulomyxa filosa|ETO19570.1
Csu|29803
Cva|20239
Alveolata|Pfiesteria piscicida|ACU44977.1
Alveolata|Babesia bovis|BBOV III005470
Alveolata|Babesia bovis|BBOV IV003210
Excavata|Trypanosoma vivax|TvY486 1003930
Cre|Cre13.g564500.t1.3
Vca|Vocar20014761m
Cre|Cre13.g571700.t1.3
Vca|Vocar20008501m
Rhizaria|Bigelowiella natans|86106
Rhizaria|Bigelowiella natans|84979
Alveolata|Babesia bovis|BBOV II007640
Rhizaria|Bigelowiella natans|87494
Rhizaria|Reticulomyxa filosa|ETO26740.1
Rhizaria|Reticulomyxa filosa|ETO35443.1
Plant|LlCCaMK|860676
Plant|NtCCaMK|4741991
Plant|MdCCaMK|1170626
X-monophyly
CaMKI&II&IV
SnRKs
MAPK&KK&KKK outgroup
0.5

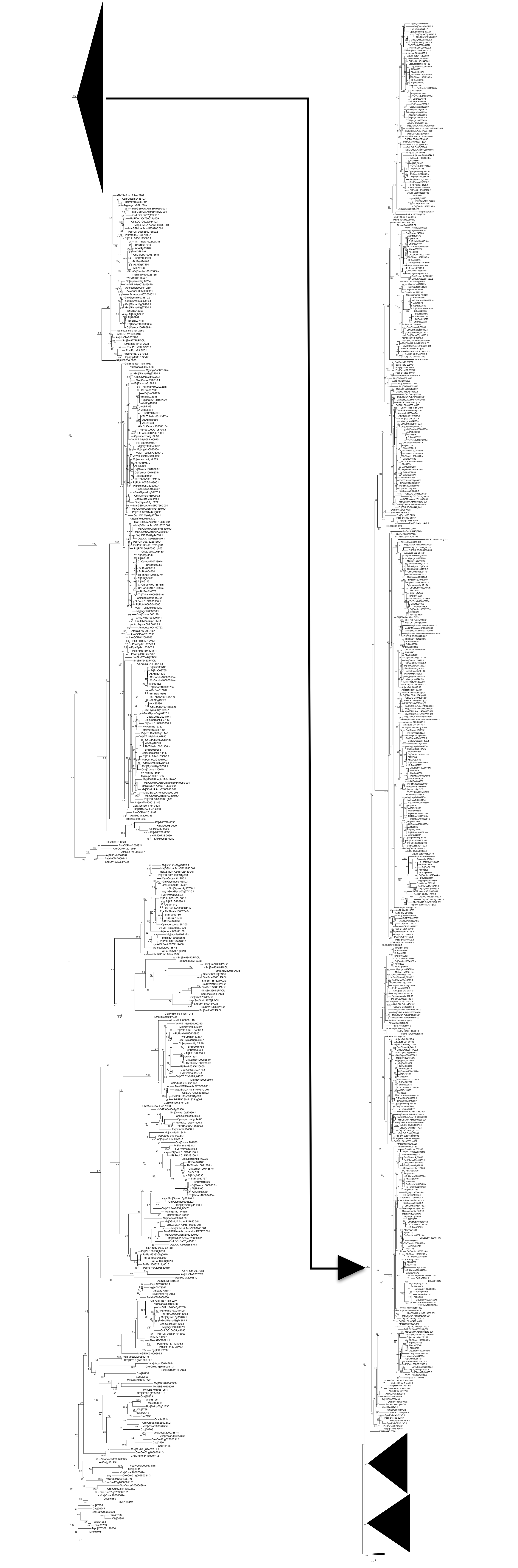

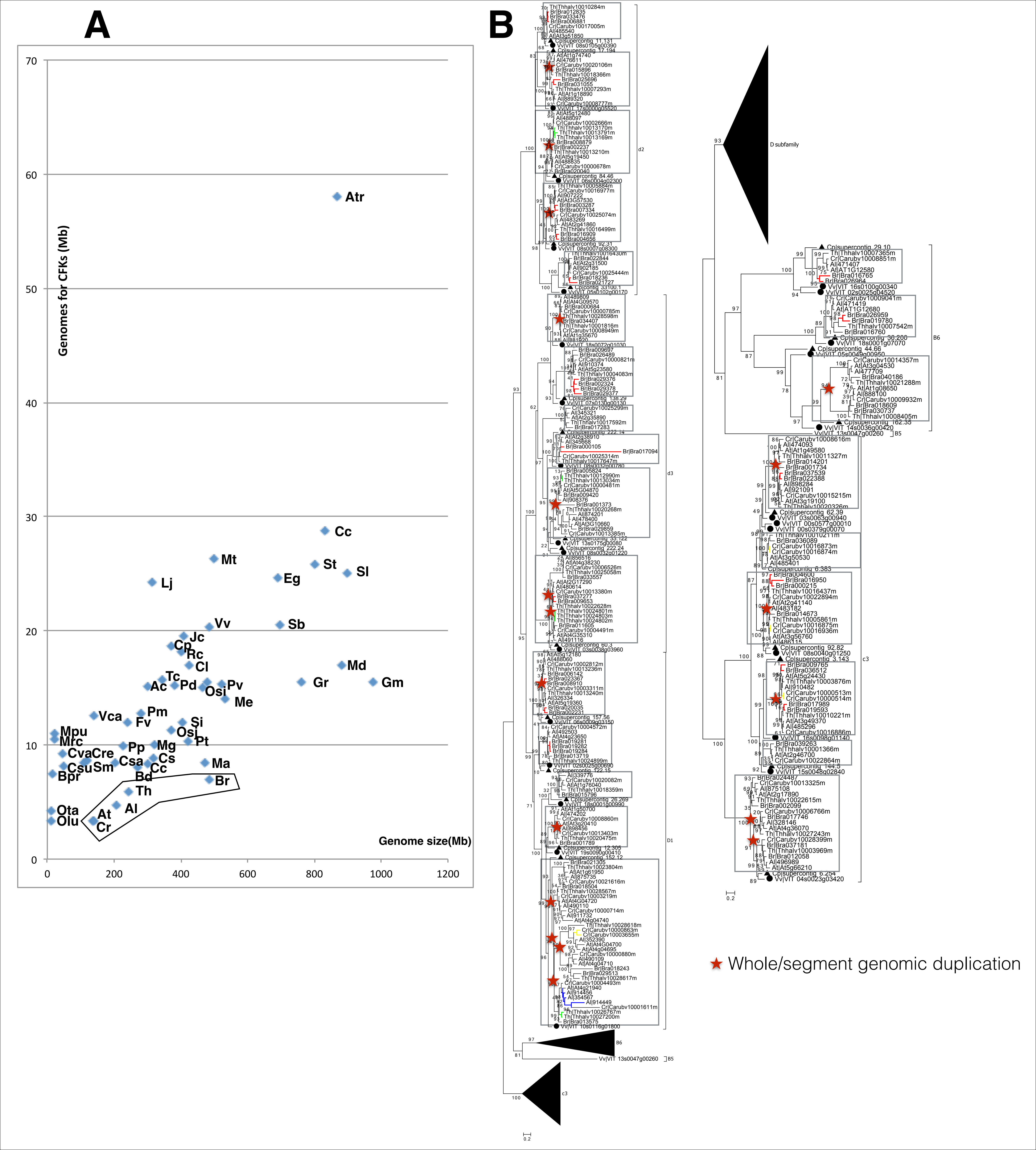

A
Genomes for CFKs (Mb)
Genome size(Mb)
0
10
20
30
40
50
60
70
0
200
400
600
800
1000
1200
Atr
Cc
Mt
St
Sl
Lj
Eg
Vv
Sb
Jc
Cp
Rc
Cl
Md
Tc
Ac
Pd
Osi
Pv
Gr
Gm
Me
Vca
Pm
Fv
Si
Mpu
Mrc
Osj
Pt
Pp
Mg
CvaCre
Cs
Csa
CsuSm
Cc
Ma
Bd
Bpr
Br
Th
Al
Ota
At
Olu
Cr
B
D subfamily
d2
d3
D1
B6
B5
c3
0.2
Whole/segment genomic duplication

1

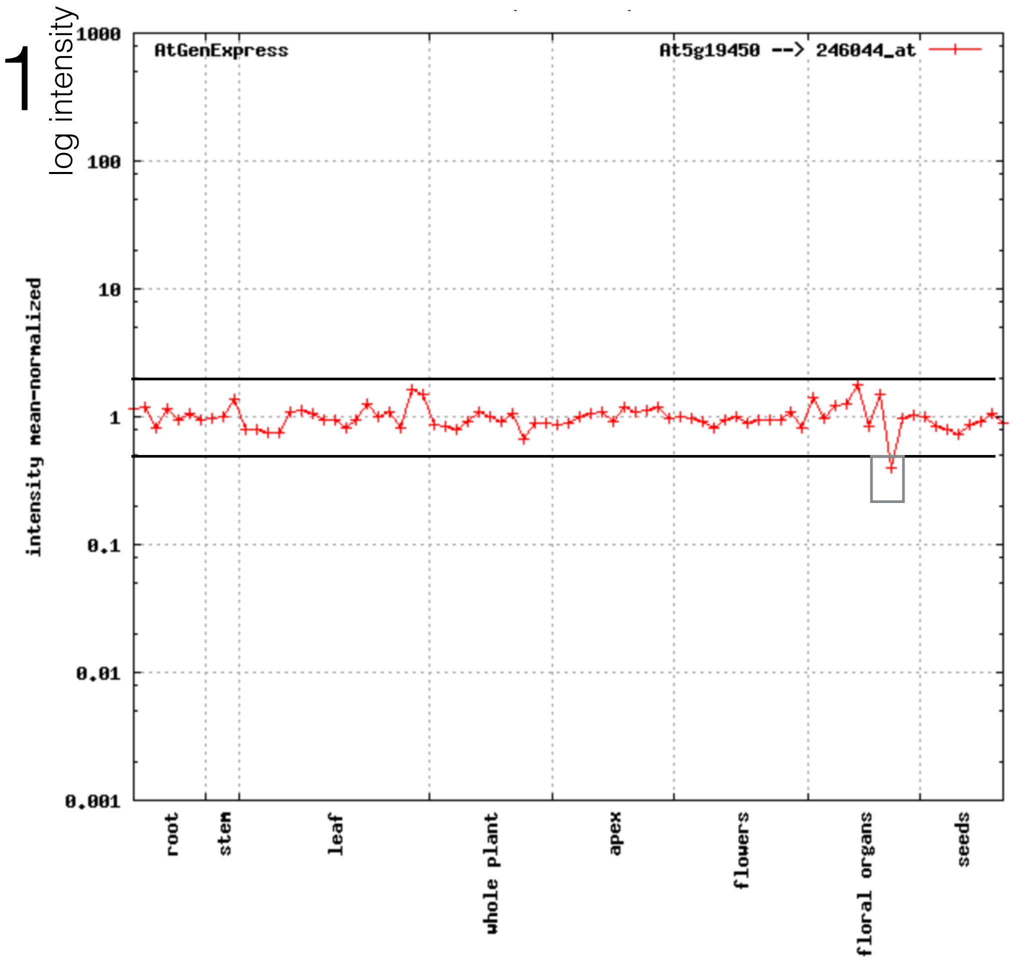
log intensity
AtGenExpress
At5g19450 --> 246044_at
intensity mean-normalized
1000
100
10
1
0.1
0.01
0.001
root
stem
leaf
whole plant
apex
flowers
floral organs
seeds

2

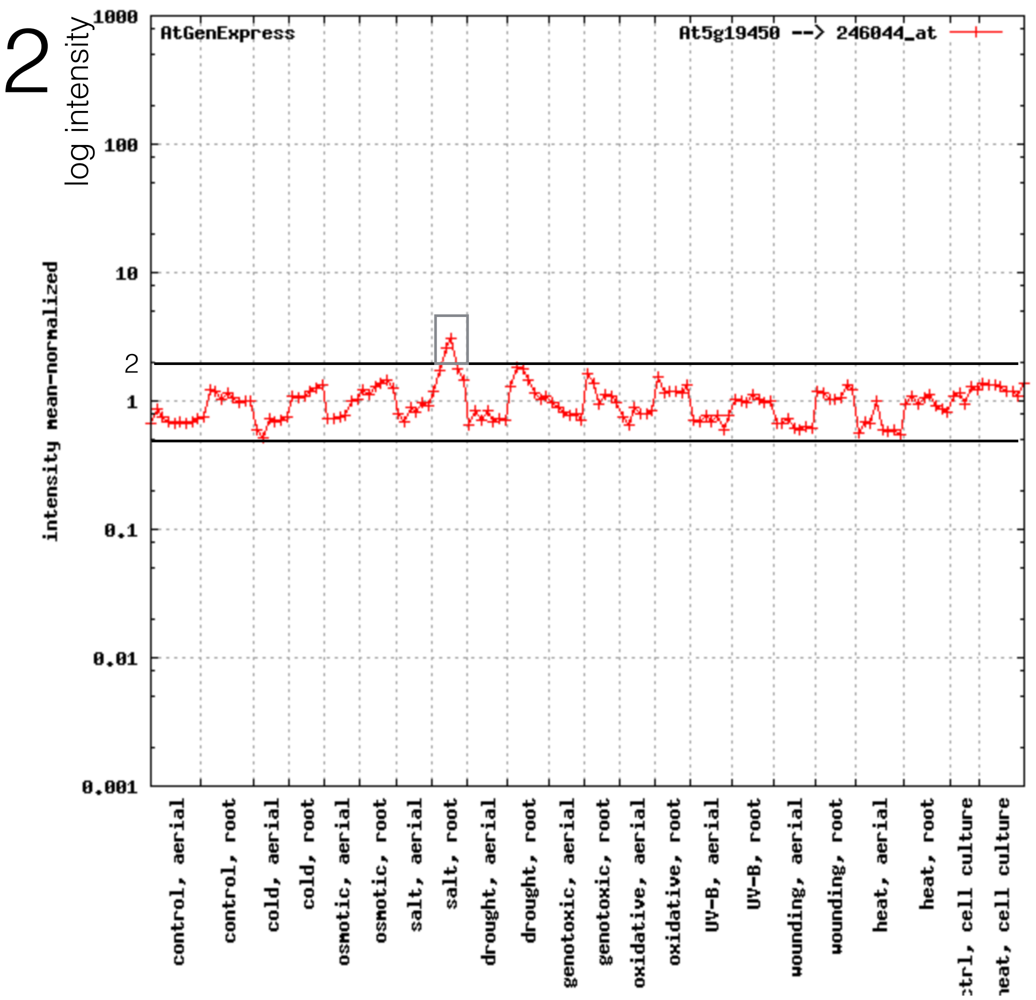
log intensity
AtGenExpress
At5g19450 --> 246044_at
intensity mean-normalized
1000
100
10
2
1
0.1
0.01
0.001
control, aerial
control, root
cold, aerial
cold, root
osmotic, aerial
osmotic, root
salt, aerial
salt, root
drought, aerial
drought, root
genotoxic, aerial
genotoxic, root
oxidative, aerial
oxidative, root
UV-B, aerial
UV-B, root
wounding, aerial
wounding, root
heat, aerial
heat, root

3

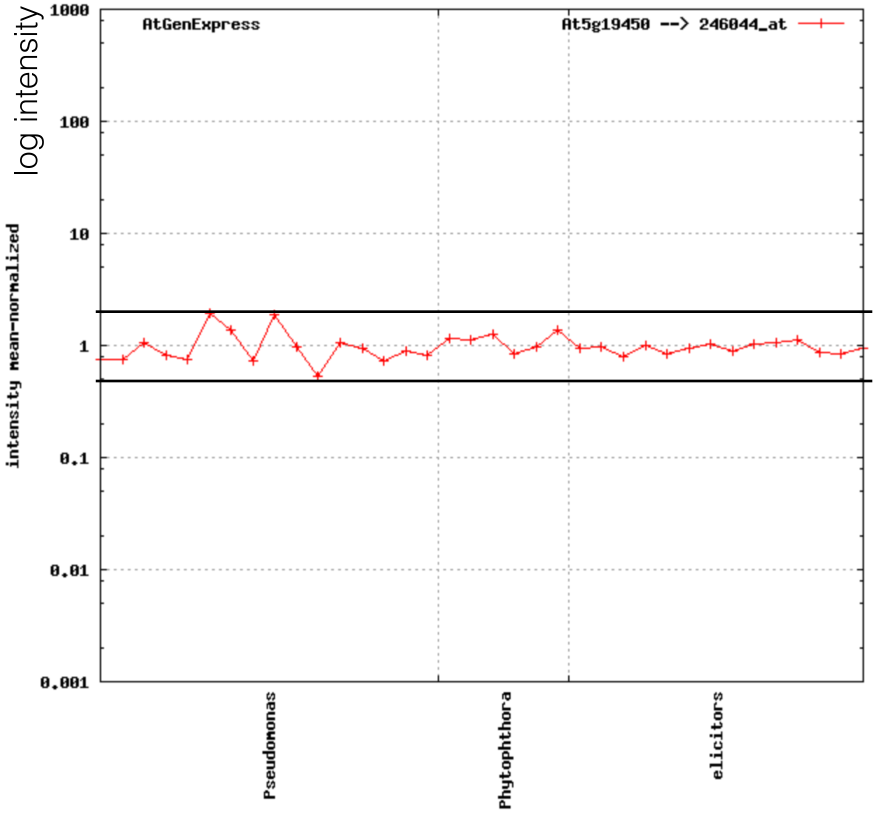
log intensity
AtGenExpress
At5g19450 --> 246044_at
intensity mean-normalized
1000
100
10
1
0.1
0.01
0.001
Pseudomonas
Phytophthora
elicitors

4

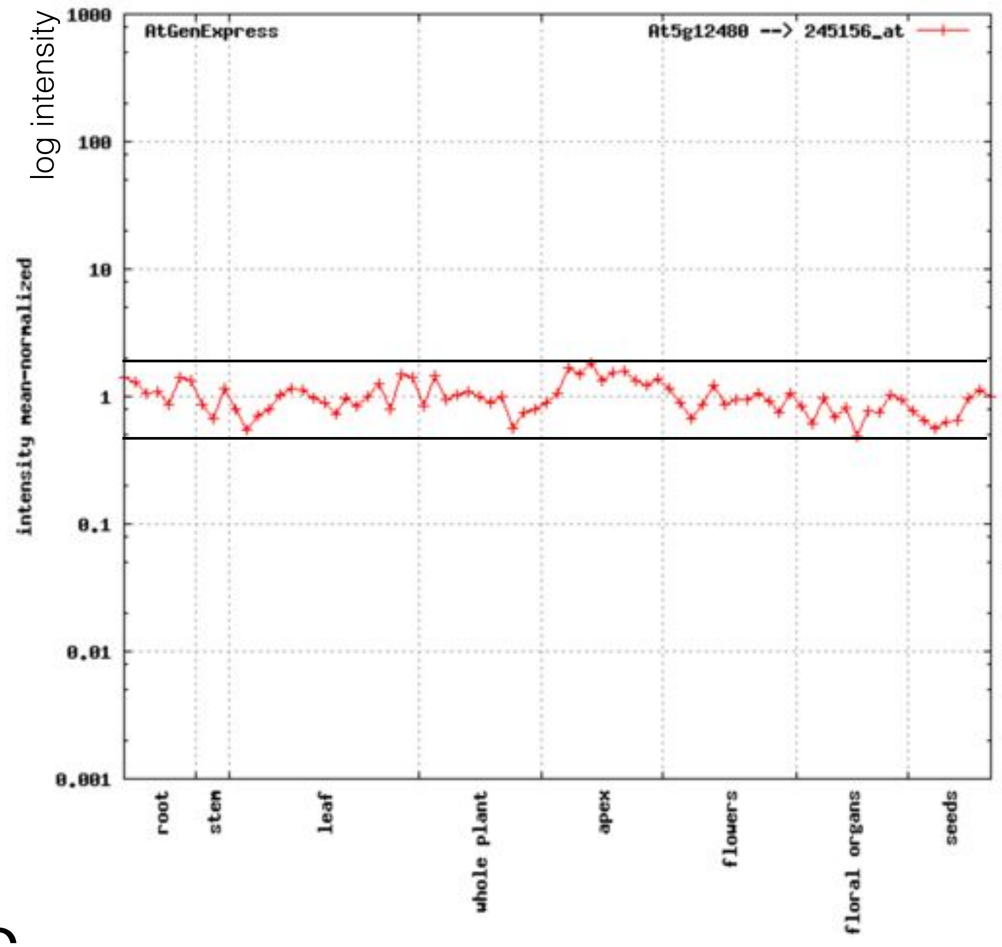

log intensity
intensity mean-normalized
AtGenExpress
At5g12480 --> 245156_at
1000
100
10
1
0.1
0.01
0.001
root
stem
leaf
whole plant
apex
flowers
floral organs
seeds

5

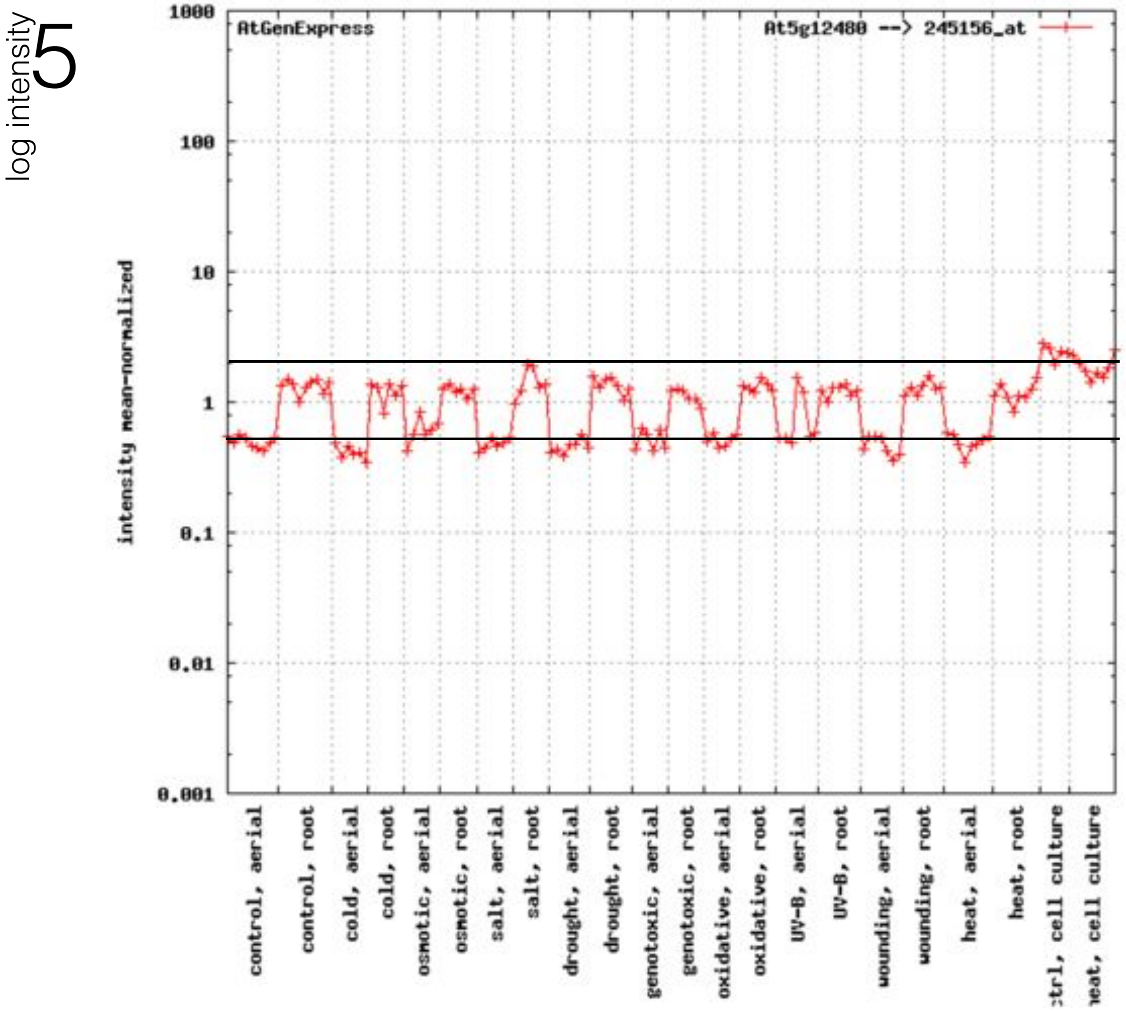

log intensity
intensity mean-normalized
AtGenExpress
At5g12480 --> 245156_at
1000
100
10
1
0.1
0.01
0.001
control, aerial
control, root
cold, aerial
cold, root
osmotic, aerial
osmotic, root
salt, aerial
salt, root
drought, aerial
drought, root
genotoxic, aerial
genotoxic, root
oxidative, aerial
oxidative, root
UV-B, aerial
UV-B, root
wounding, aerial
wounding, root
heat, aerial
heat, root
ctrl, cell culture
heat, cell culture

6

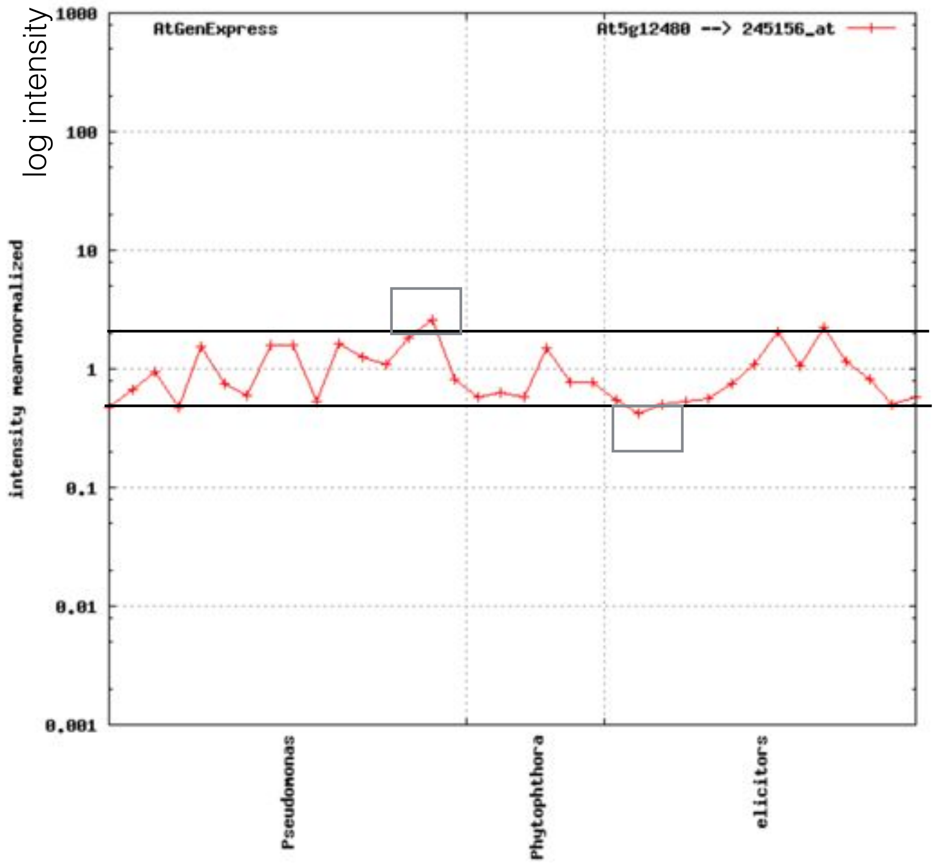

log intensity
intensity mean-normalized
AtGenExpress
At5g12480 --> 245156_at
1000
100
10
1
0.1
0.01
0.001
Pseudomonas
Phytophthora
elicitors

7

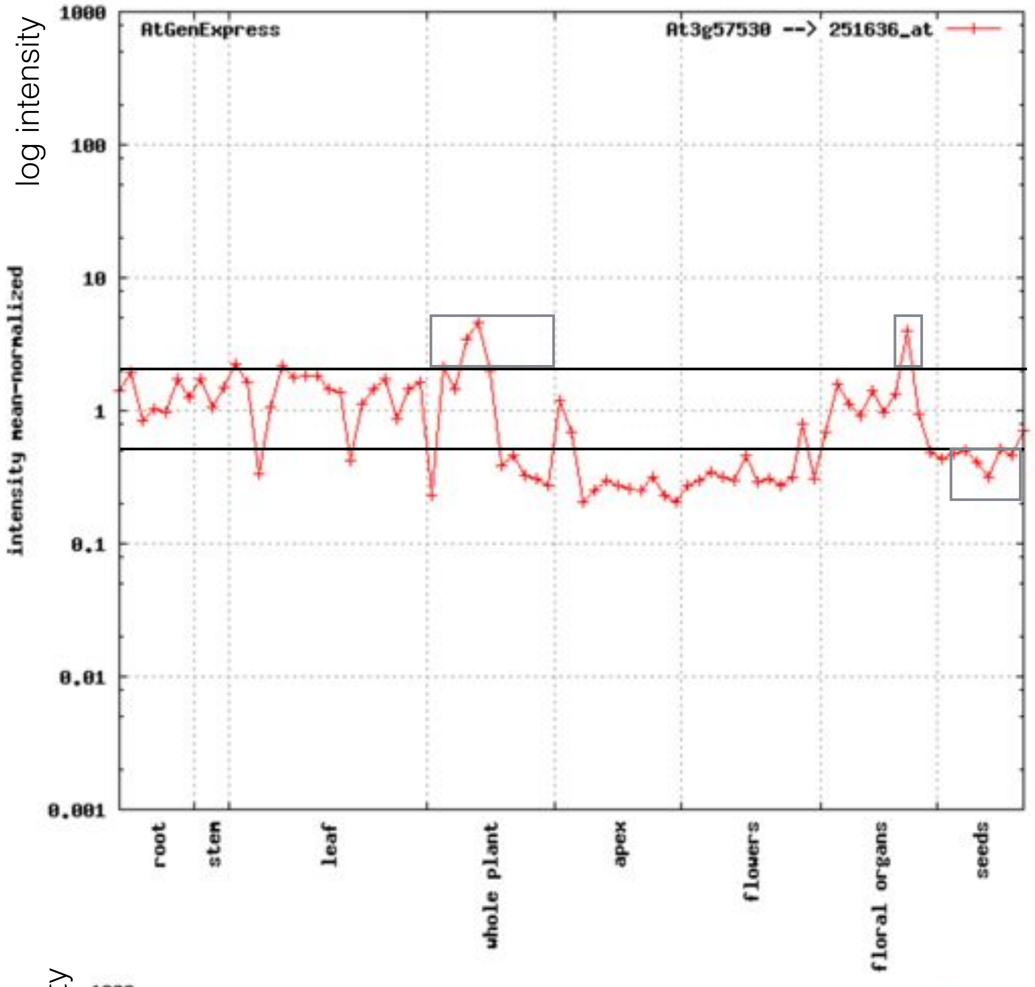
log intensity
AtGenExpress
At3g57530 --> 251636_at
1000
100
10
1
0.1
0.01
0.001
intensity mean-normalized
root
stem
leaf
whole plant
apex
flowers
floral organs
seeds

8

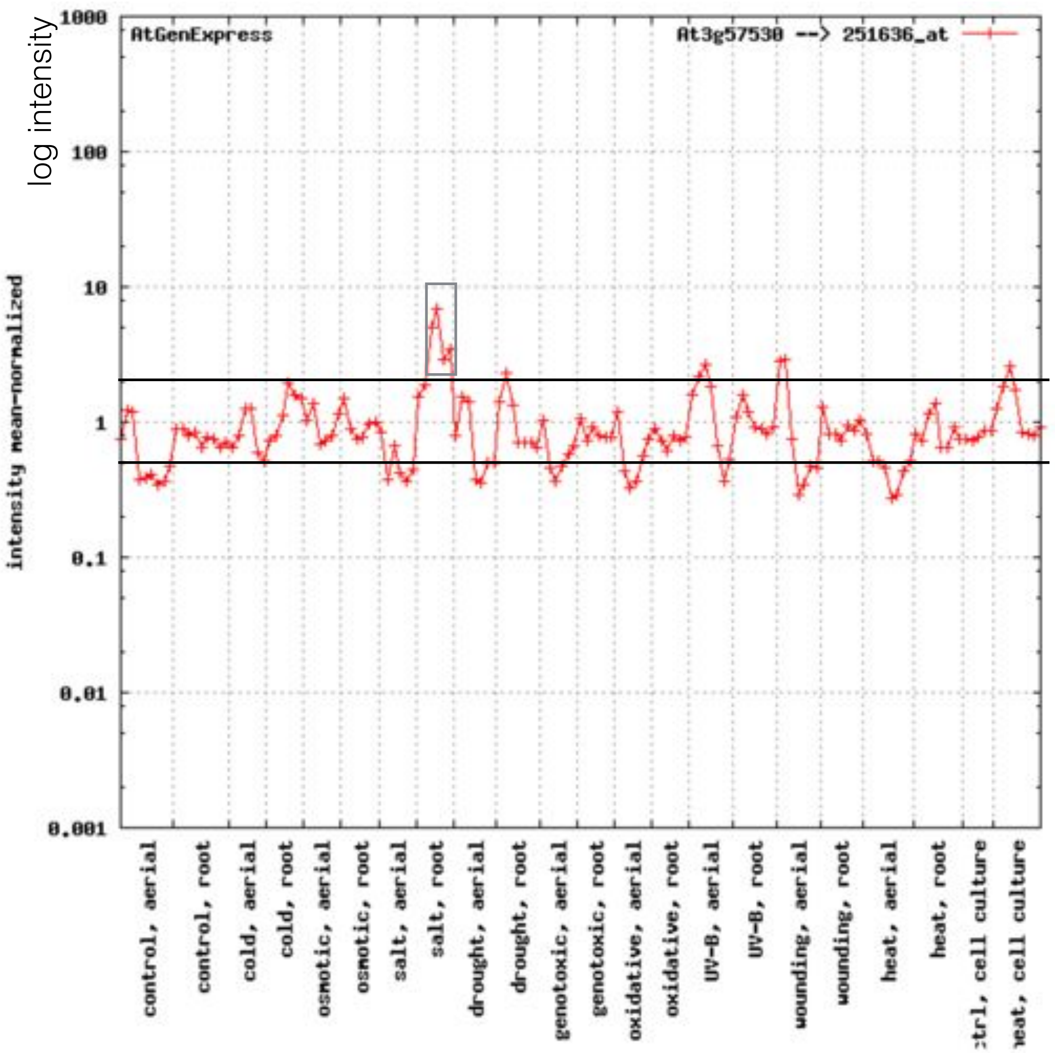
log intensity
AtGenExpress
At3g57530 --> 251636_at
1000
100
10
1
0.1
0.01
0.001
intensity mean-normalized
control, aerial
control, root
cold, aerial
cold, root
osmotic, aerial
osmotic, root
salt, aerial
salt, root
drought, aerial
drought, root
genotoxic, aerial
genotoxic, root
oxidative, aerial
oxidative, root
UV-B, aerial
UV-B, root
wounding, aerial
wounding, root
heat, aerial
heat, root
trl, cell culture
eat, cell culture

9

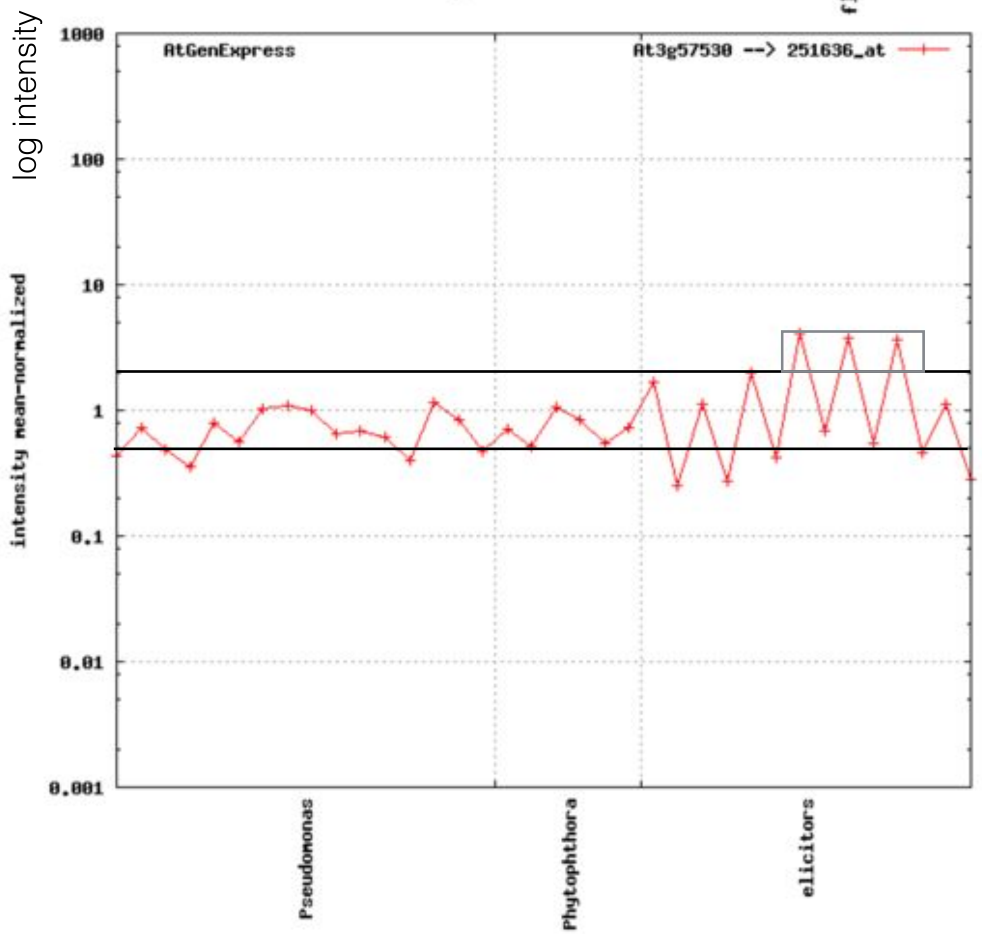
log intensity
AtGenExpress
At3g57530 --> 251636_at
1000
100
10
1
0.1
0.01
0.001
intensity mean-normalized
Pseudomonas
Phytophthora
elicitors

10

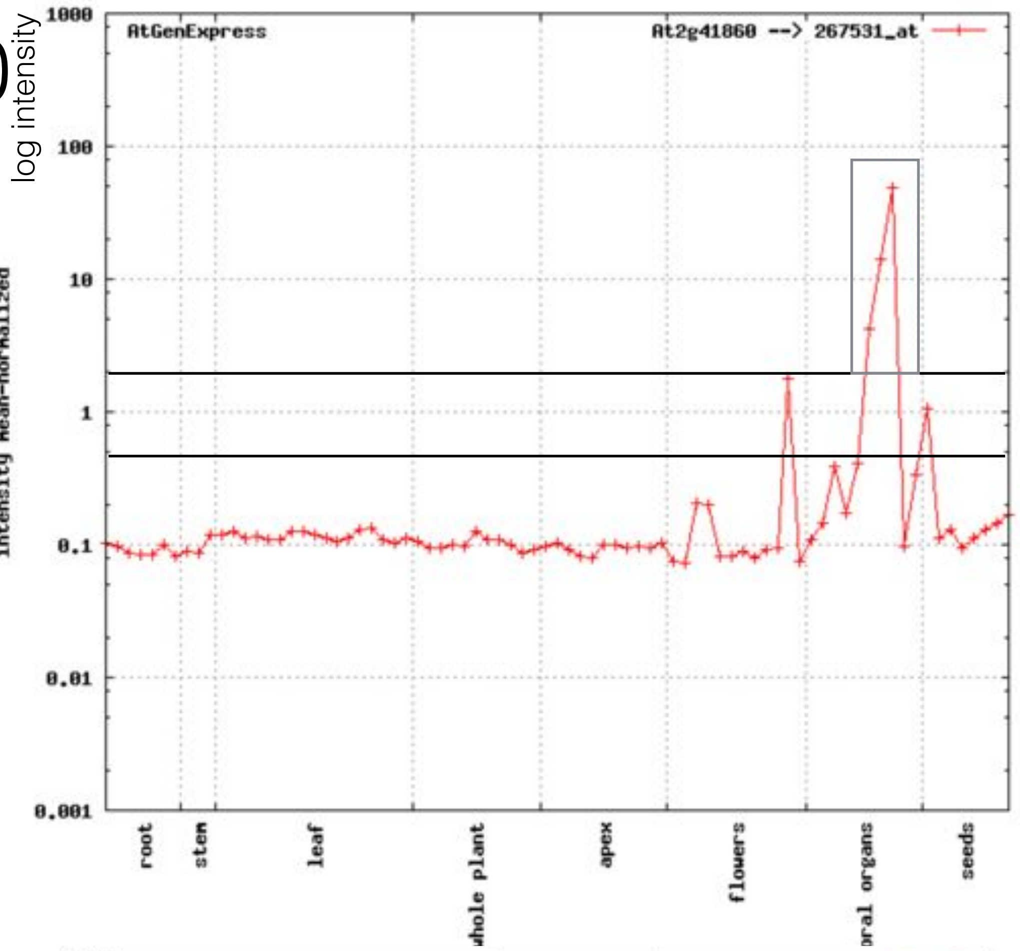
log intensity
intensity mean-normalized
AtGenExpress
At2g41860 --> 267531_at
1000
100
10
1
0.1
0.01
0.001
root
stem
leaf
whole plant
apex
flowers
oral organs
seeds

11

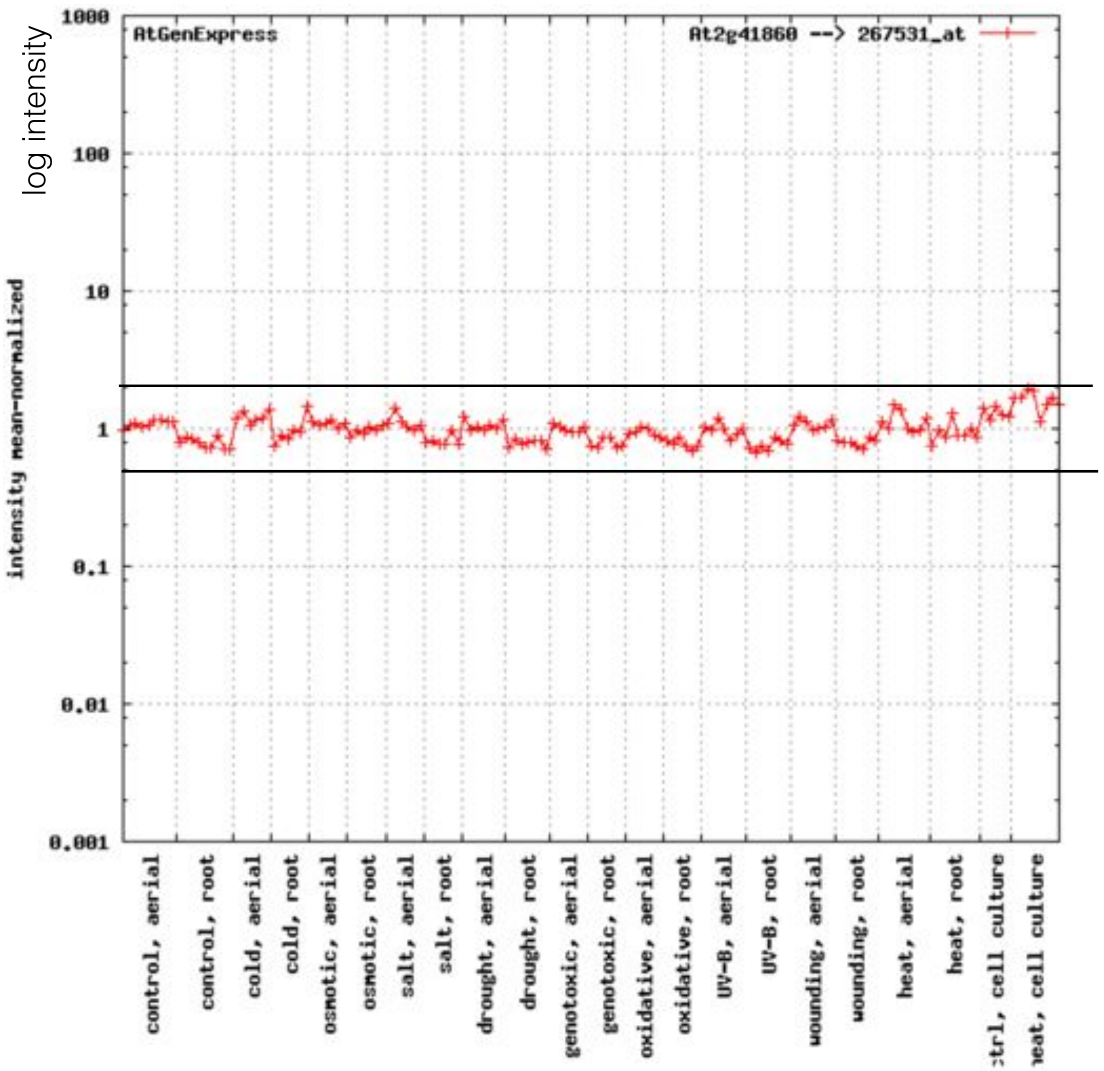
log intensity
intensity mean-normalized
AtGenExpress
At2g41860 --> 267531_at
1000
100
10
1
0.1
0.01
0.001
control, aerial
control, root
cold, aerial
cold, root
osmotic, aerial
osmotic, root
salt, aerial
salt, root
drought, aerial
drought, root
genotoxic, aerial
genotoxic, root
oxidative, aerial
oxidative, root
UV-B, aerial
UV-B, root
wounding, aerial
wounding, root
heat, aerial
heat, root
ctrl, cell culture
heat, cell culture

12

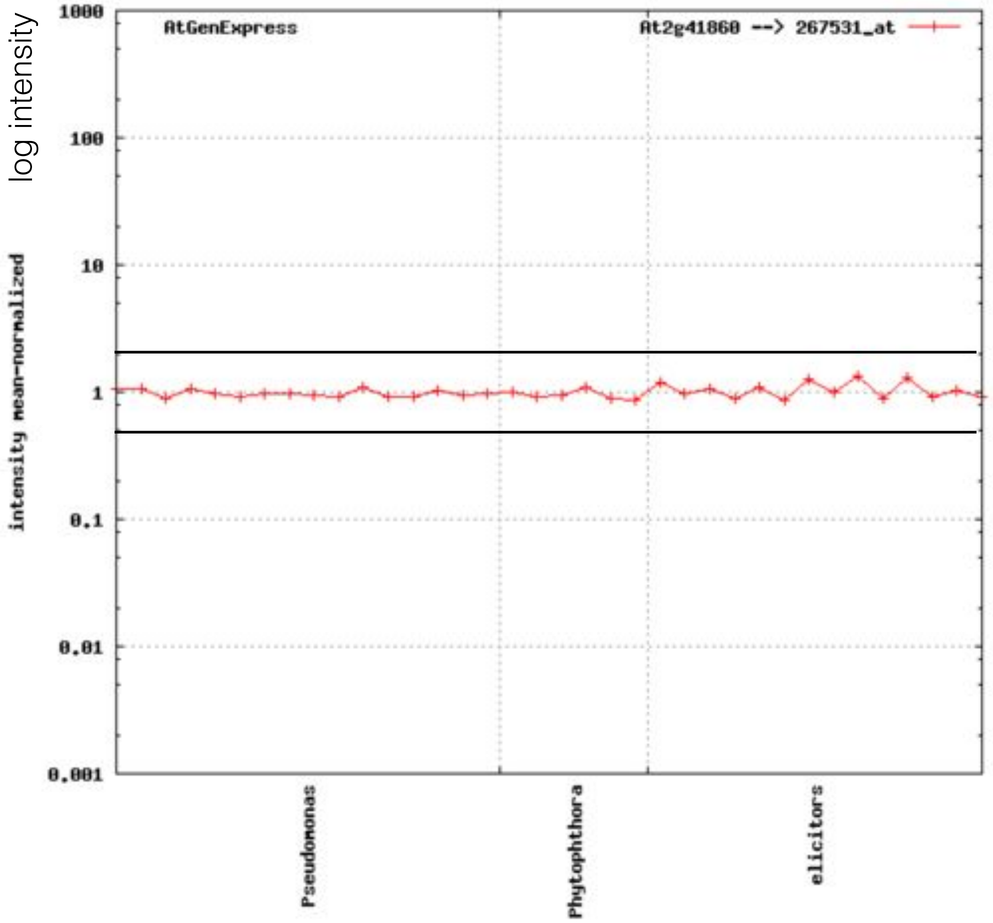
log intensity
intensity mean-normalized
AtGenExpress
At2g41860 --> 267531_at
1000
100
10
1
0.1
0.01
0.001
Pseudomonas
Phytophthora
elicitors

13

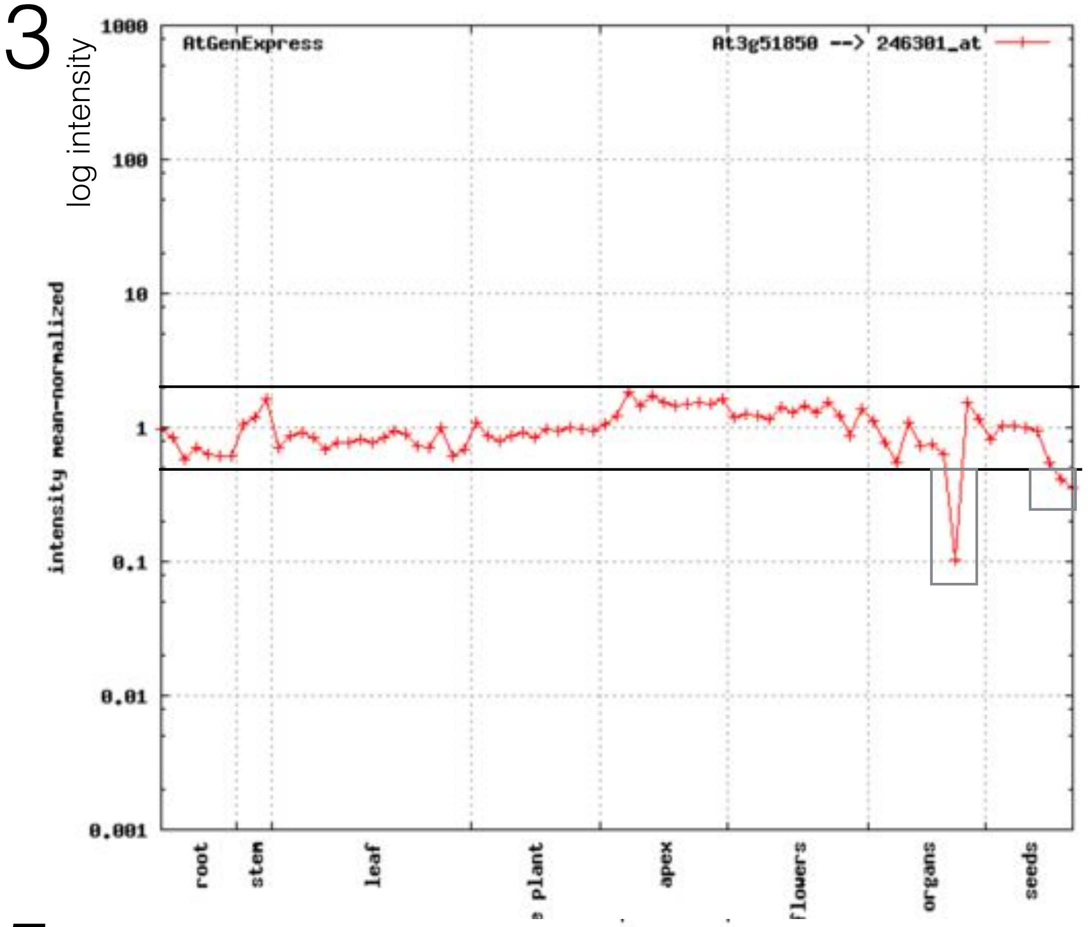
log intensity
AtGenExpress
At3g51850 --> 246301_at
intensity mean-normalized
1000
100
10
1
0.1
0.01
0.001
root
stem
leaf
plant
apex
organs
seeds

14

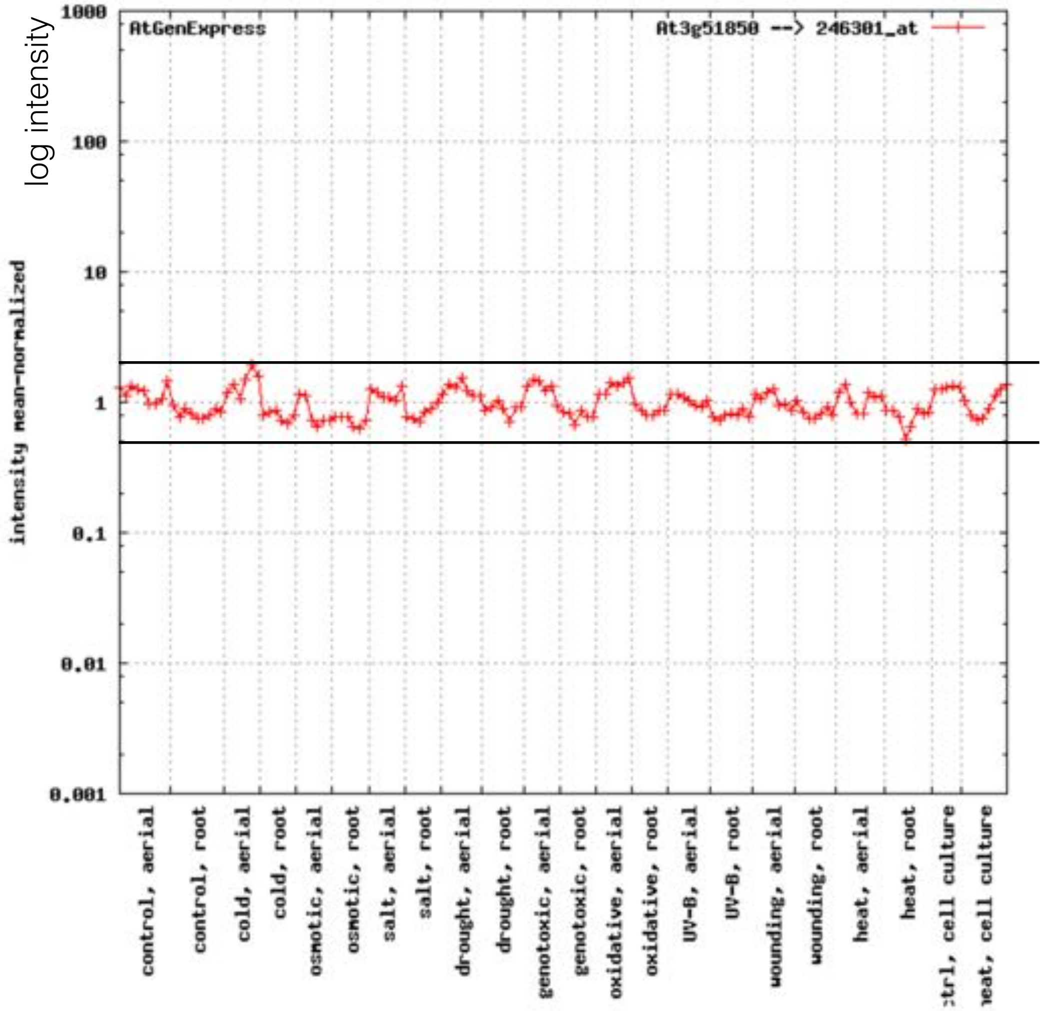
log intensity
AtGenExpress
At3g51850 --> 246301_at
intensity mean-normalized
1000
100
10
1
0.1
0.01
0.001
control, aerial
control, root
cold, aerial
cold, root
osmotic, aerial
osmotic, root
salt, aerial
salt, root
drought, aerial
drought, root
genotoxic, aerial
genotoxic, root
oxidative, aerial
oxidative, root
UV-B, aerial
UV-B, root
wounding, aerial
wounding, root
heat, aerial
heat, root

15

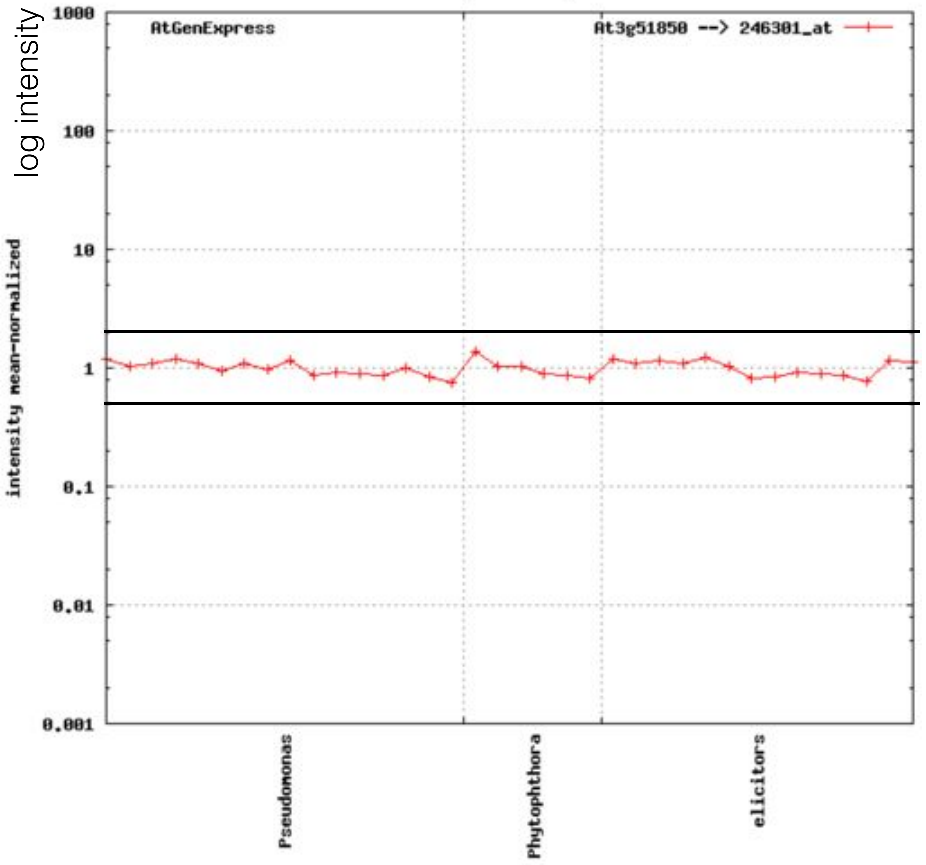
log intensity
AtGenExpress
At3g51850 --> 246301_at
intensity mean-normalized
1000
100
10
1
0.1
0.01
0.001
Pseudomonas
Phytophthora
elicitors

16

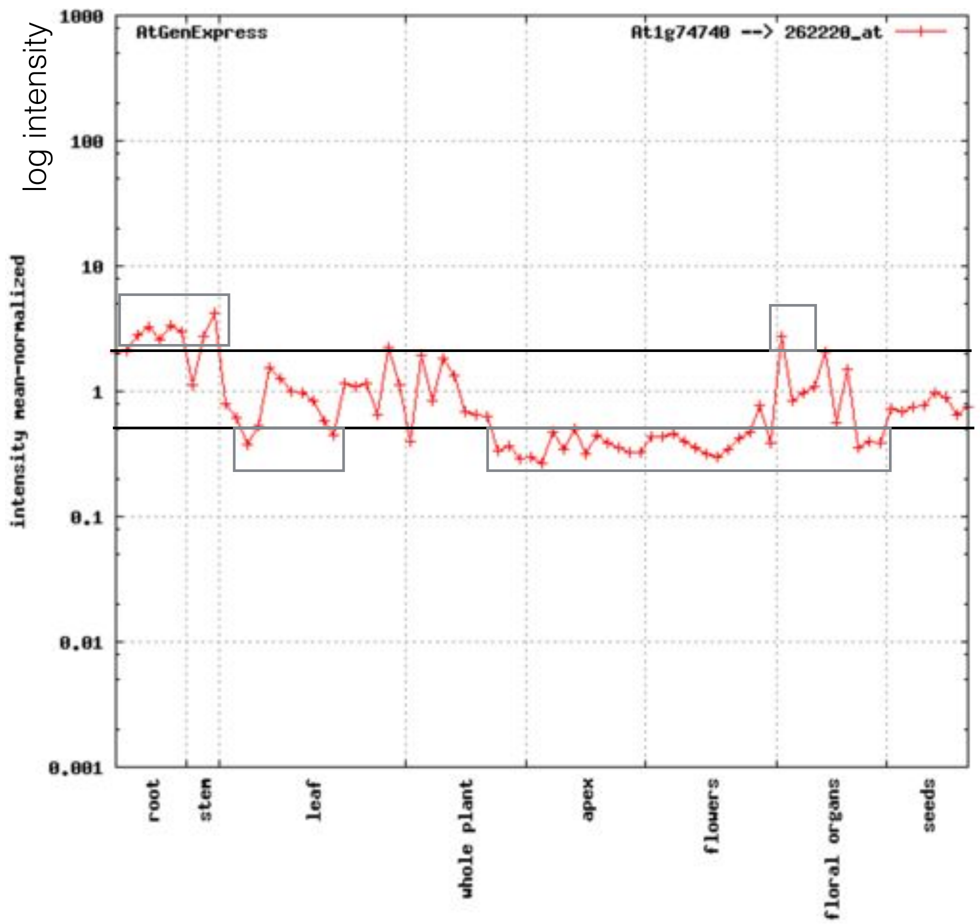
log intensity
intensity mean-normalized
AtGenExpress
At1g74740 --> 262220_at
1000
100
10
1
0.1
0.01
0.001
root
stem
leaf
whole plant
apex
flowers
floral organs
seeds

17

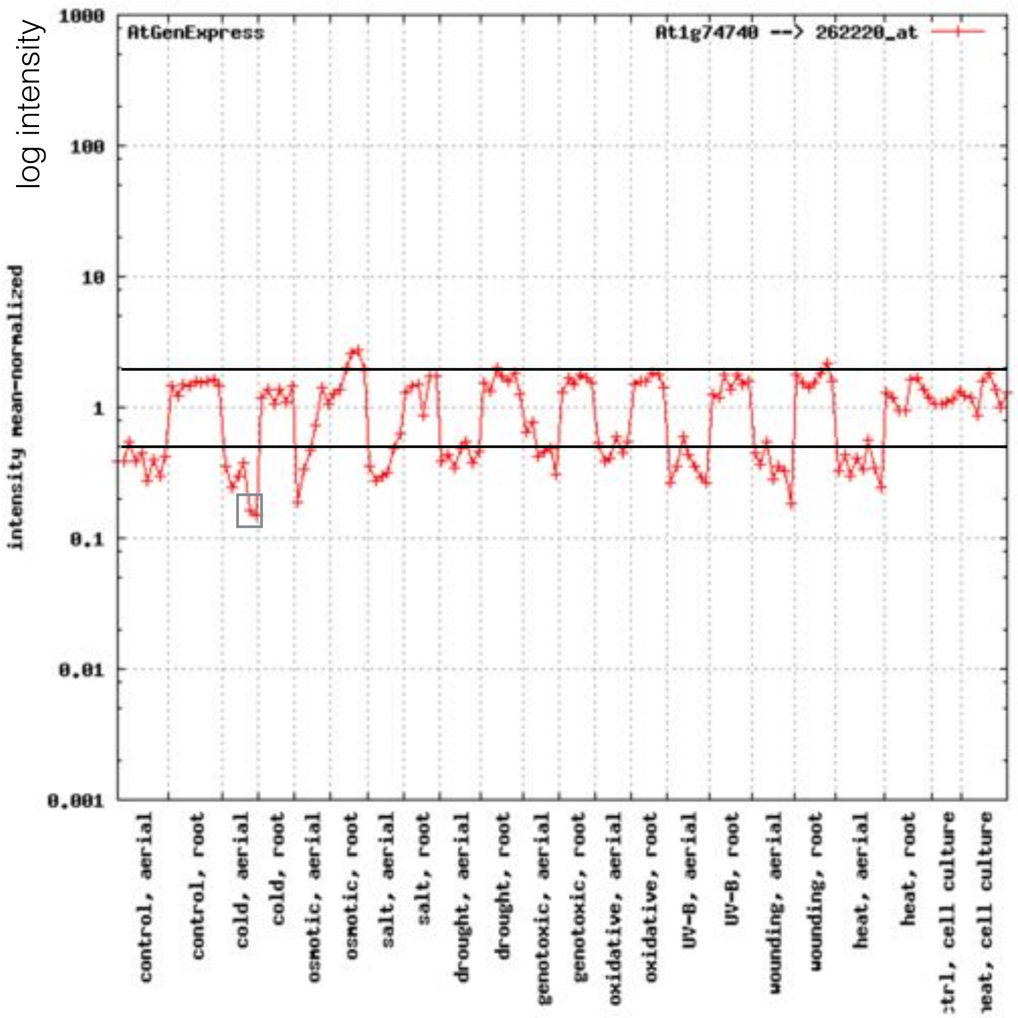
log intensity
intensity mean-normalized
AtGenExpress
At1g74740 --> 262220_at
1000
100
10
1
0.1
0.01
0.001
control, aerial
control, root
cold, aerial
cold, root
osmotic, aerial
osmotic, root
salt, aerial
salt, root
drought, aerial
drought, root
genotoxic, aerial
genotoxic, root
oxidative, aerial
oxidative, root
UV-B, aerial
UV-B, root
wounding, aerial
wounding, root
heat, aerial
heat, root

18

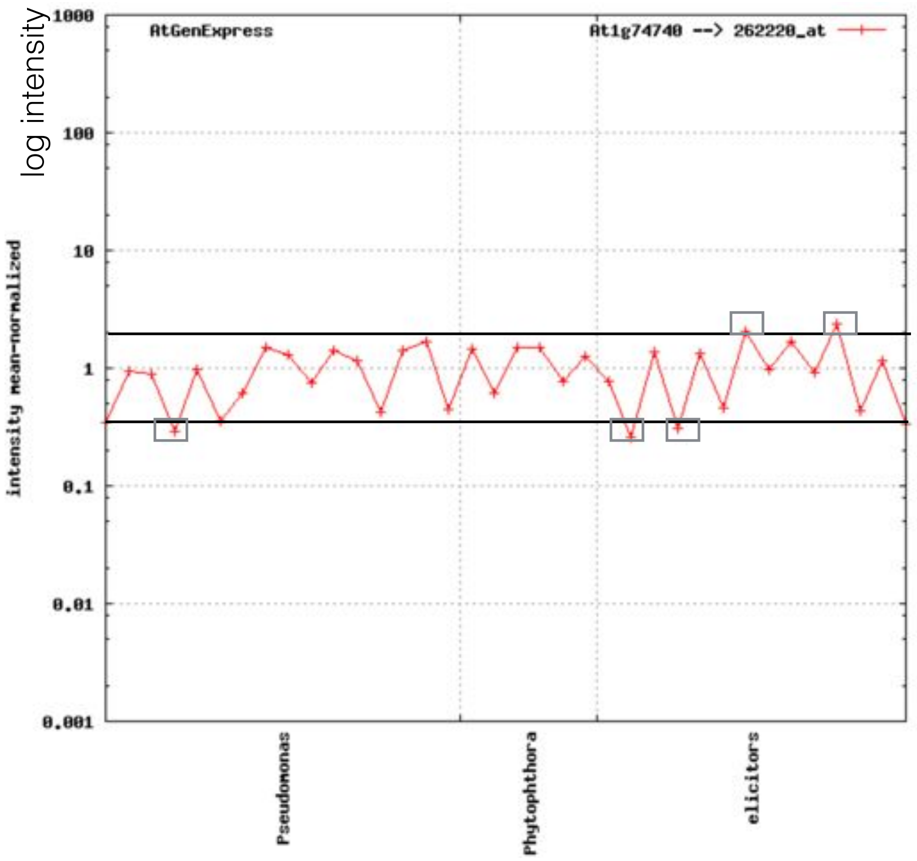
log intensity
intensity mean-normalized
AtGenExpress
At1g74740 --> 262220_at
1000
100
10
1
0.1
0.01
0.001
Pseudomonas
Phytophthora
elicitors

19

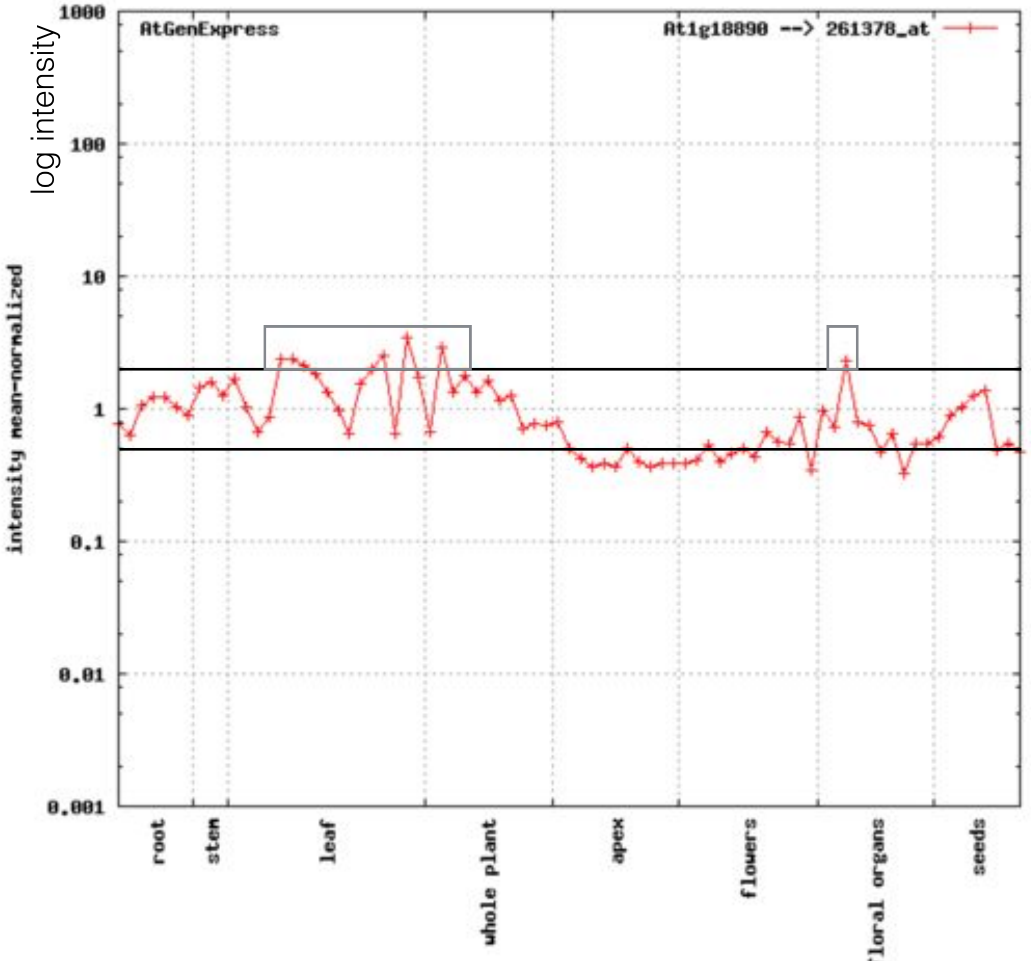
log intensity
intensity mean-normalized
AtGenExpress
At1g18890 --> 261378_at
1000
100
10
1
0.1
0.01
0.001
root
stem
leaf
whole plant
apex
flowers
floral organs
seeds

20

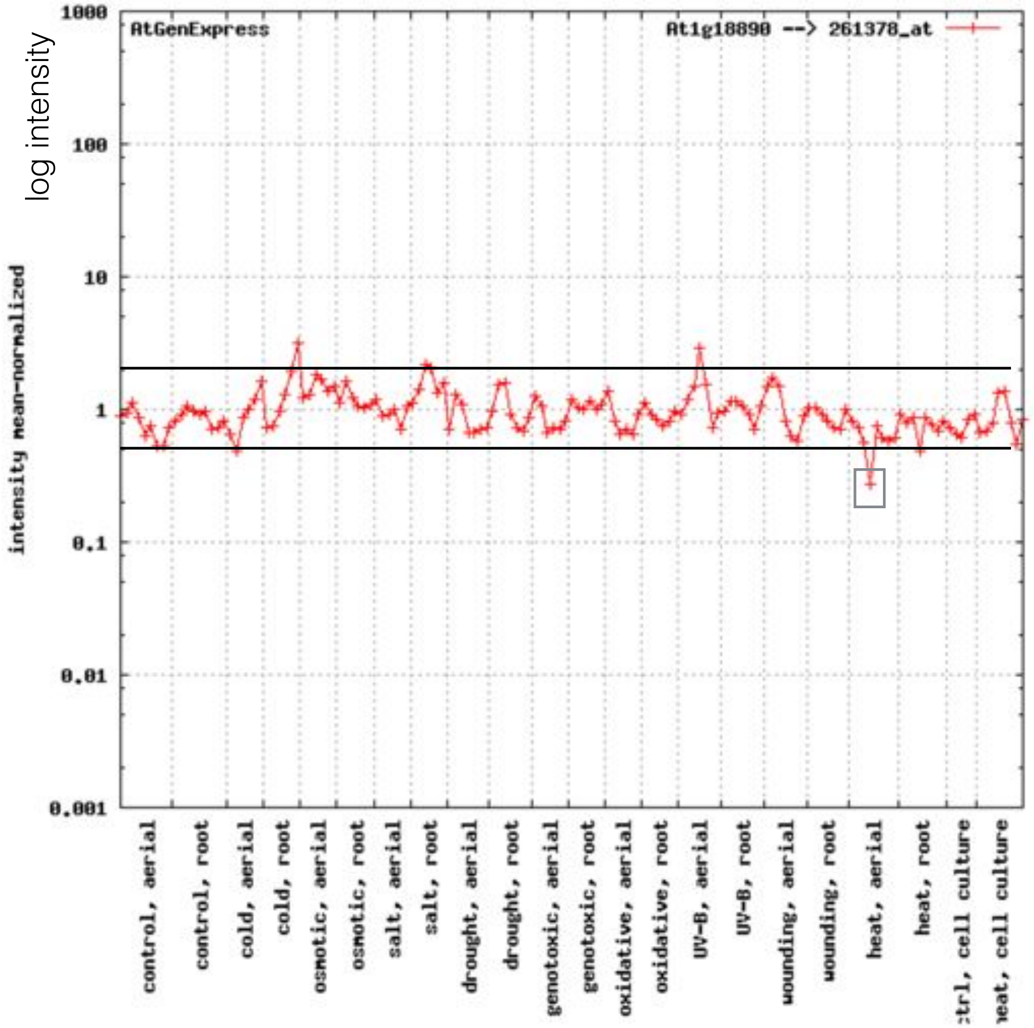
log intensity
intensity mean-normalized
AtGenExpress
At1g18890 --> 261378_at
1000
100
10
1
0.1
0.01
0.001
control, aerial
control, root
cold, aerial
cold, root
osmotic, aerial
osmotic, root
salt, aerial
salt, root
drought, aerial
drought, root
genotoxic, aerial
genotoxic, root
oxidative, aerial
oxidative, root
UV-B, aerial
UV-B, root
wounding, aerial
wounding, root
heat, aerial
heat, root

21

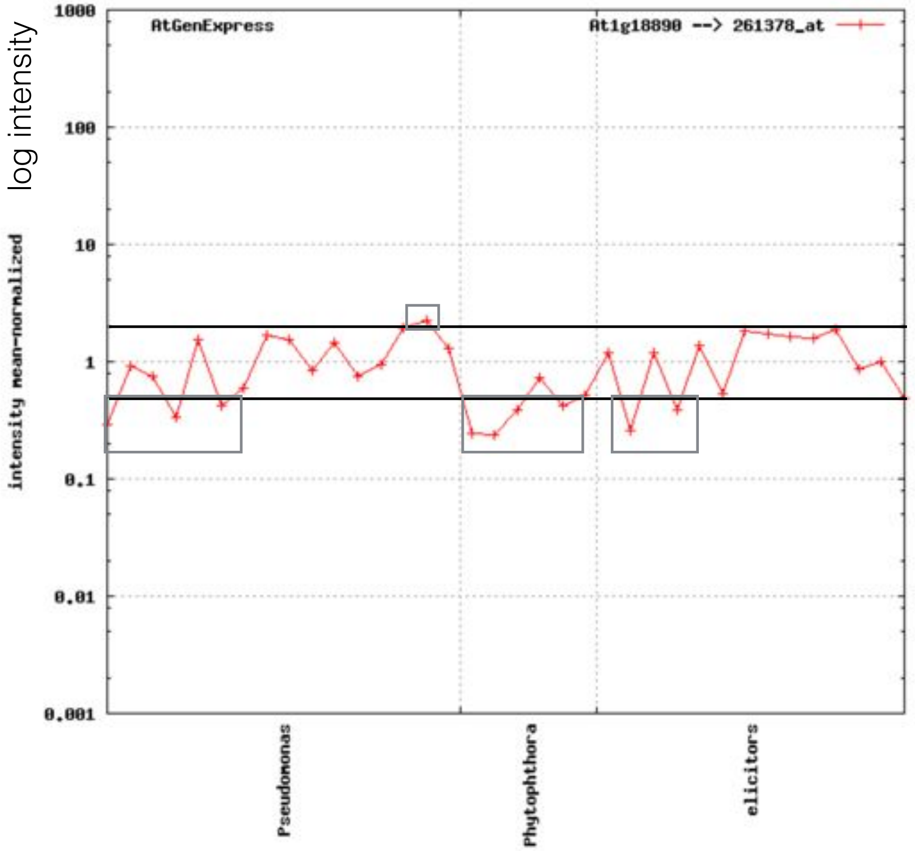
log intensity
intensity mean-normalized
AtGenExpress
At1g18890 --> 261378_at
1000
100
10
1
0.1
0.01
0.001
Pseudomonas
Phytophthora
elicitors

22

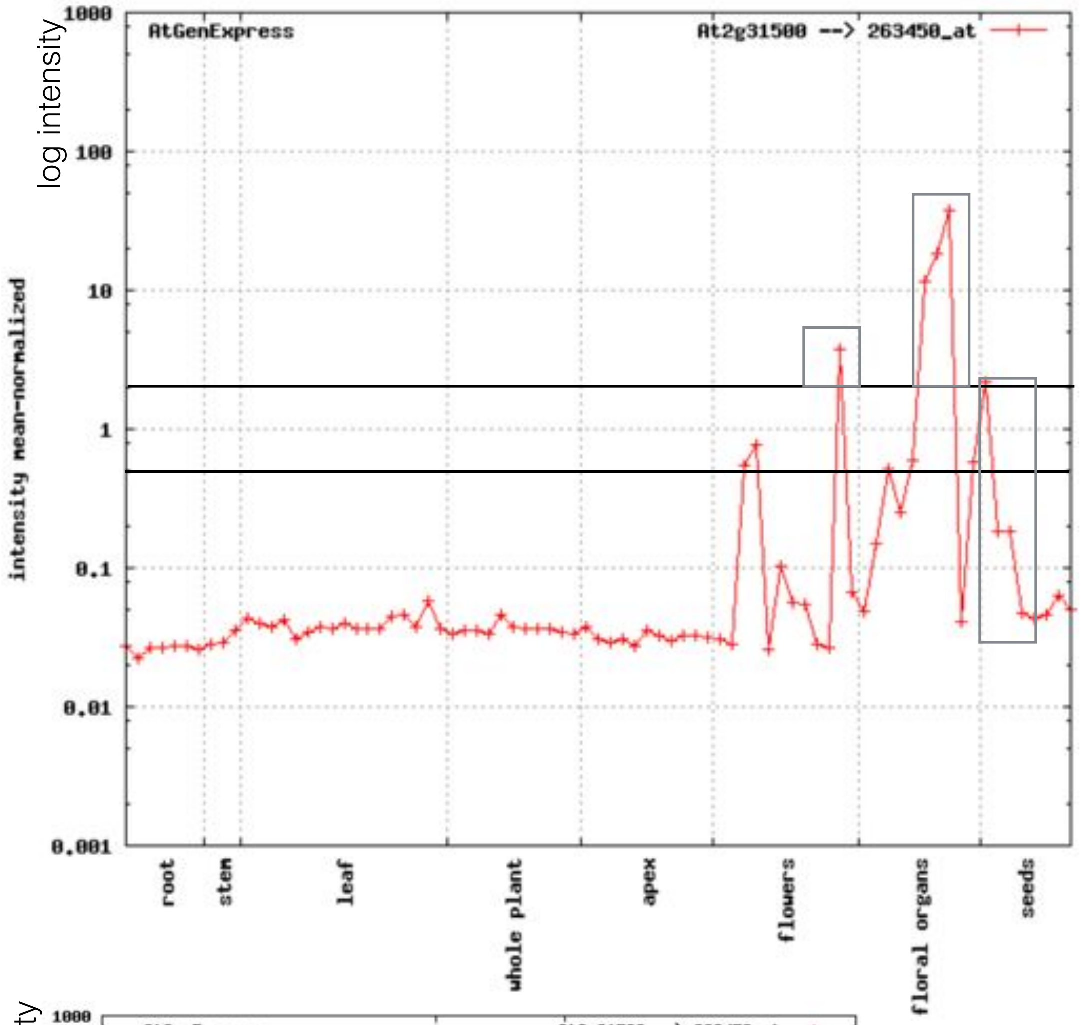
log intensity
intensity mean-normalized
AtGenExpress
At2g31500 --> 263450_at
1000
100
10
1
0.1
0.01
0.001
root
stem
leaf
whole plant
apex
flowers
floral organs
seeds

23

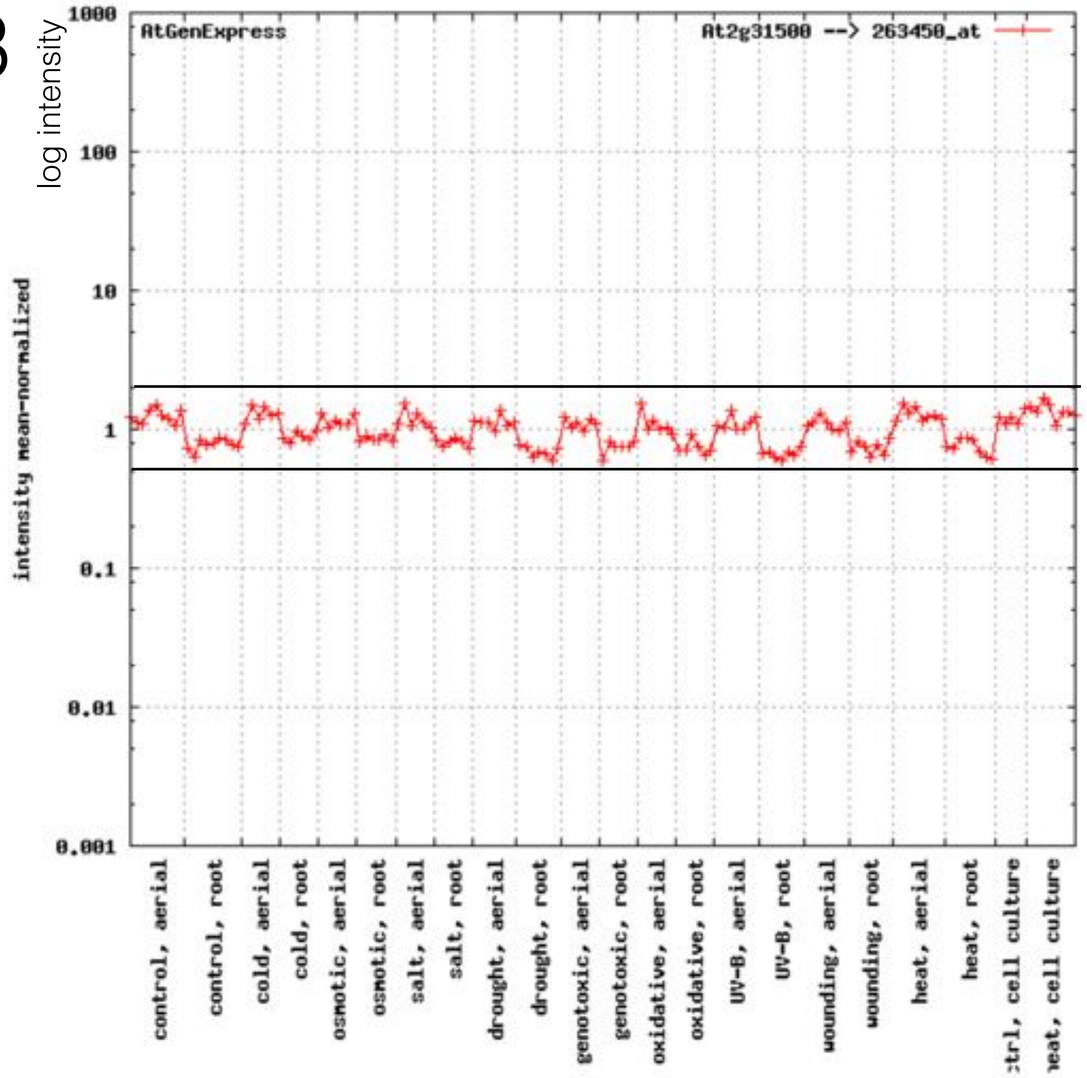
log intensity
intensity mean-normalized
AtGenExpress
At2g31500 --> 263450_at
1000
100
10
1
0.1
0.01
0.001
control, aerial
control, root
cold, aerial
cold, root
osmotic, aerial
osmotic, root
salt, aerial
salt, root
drought, aerial
drought, root
genotoxic, aerial
genotoxic, root
oxidative, aerial
oxidative, root
UV-B, aerial
UV-B, root
wounding, aerial
wounding, root
heat, aerial
heat, root
ctrl, cell culture
heat, cell culture

24

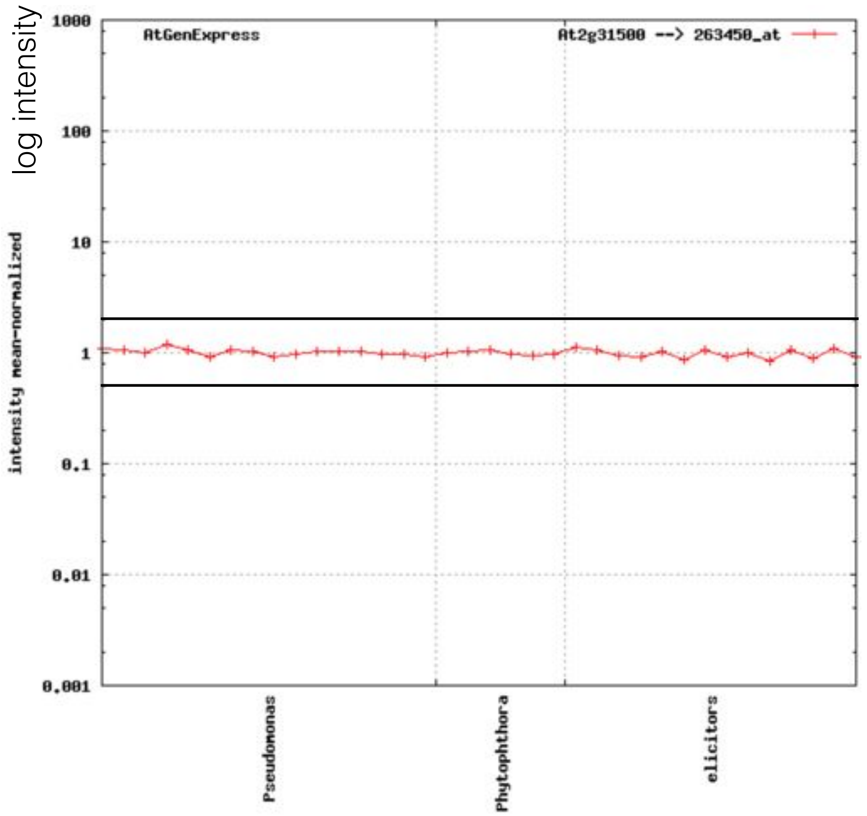
log intensity
intensity mean-normalized
AtGenExpress
At2g31500 --> 263450_at
1000
100
10
1
0.1
0.01
0.001
Pseudomonas
Phytophthora
elicitors

25

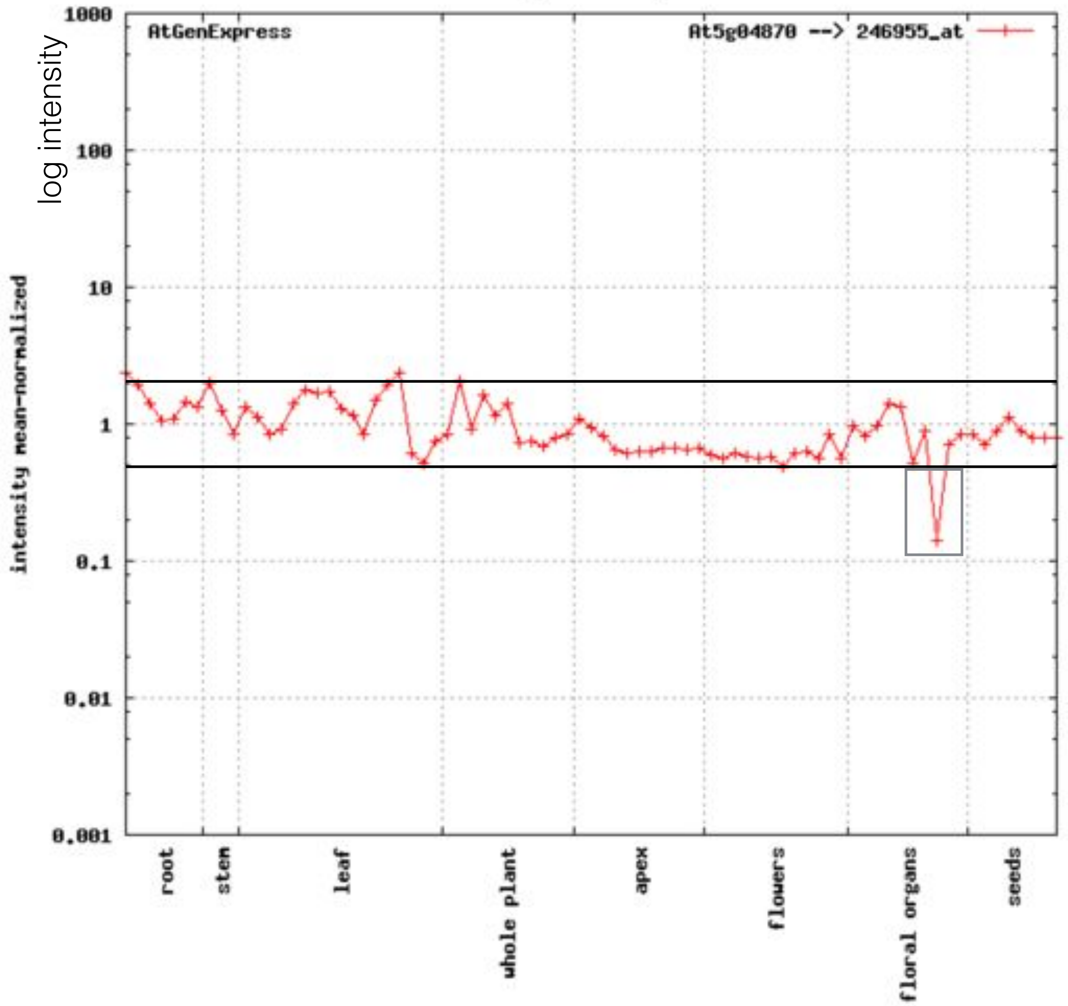

log intensity
intensity mean-normalized
AtGenExpress
At5g04870 --> 246955_at
1000
100
10
1
0.1
0.01
0.001
root
stem
leaf
whole plant
apex
flowers
floral organs
seeds

26

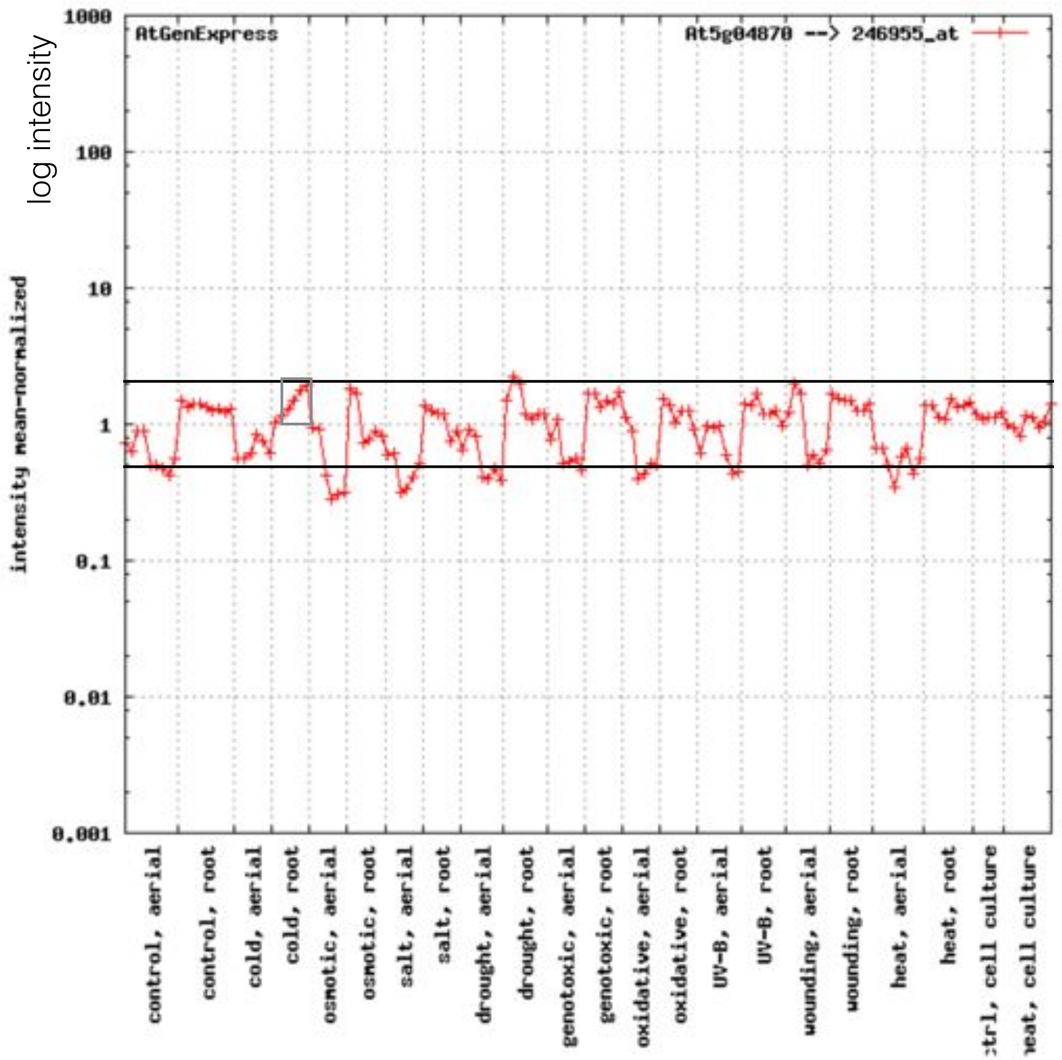

log intensity
intensity mean-normalized
AtGenExpress
At5g04870 --> 246955_at
1000
100
10
1
0.1
0.01
0.001
control, aerial
control, root
cold, aerial
cold, root
osmotic, aerial
osmotic, root
salt, aerial
salt, root
drought, aerial
drought, root
genotoxic, aerial
genotoxic, root
oxidative, aerial
oxidative, root
UV-B, aerial
UV-B, root
wounding, aerial
wounding, root
heat, aerial
heat, root

27

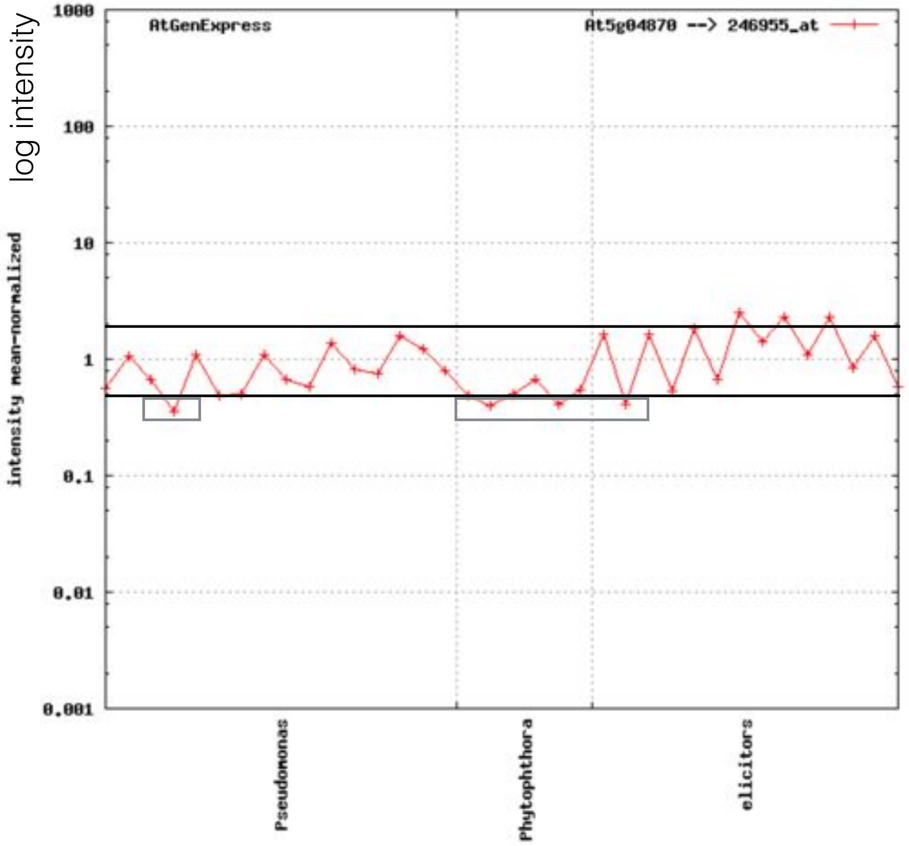

log intensity
intensity mean-normalized
AtGenExpress
At5g04870 --> 246955_at
1000
100
10
1
0.1
0.01
0.001
Pseudomonas
Phytophthora
elicitors

28

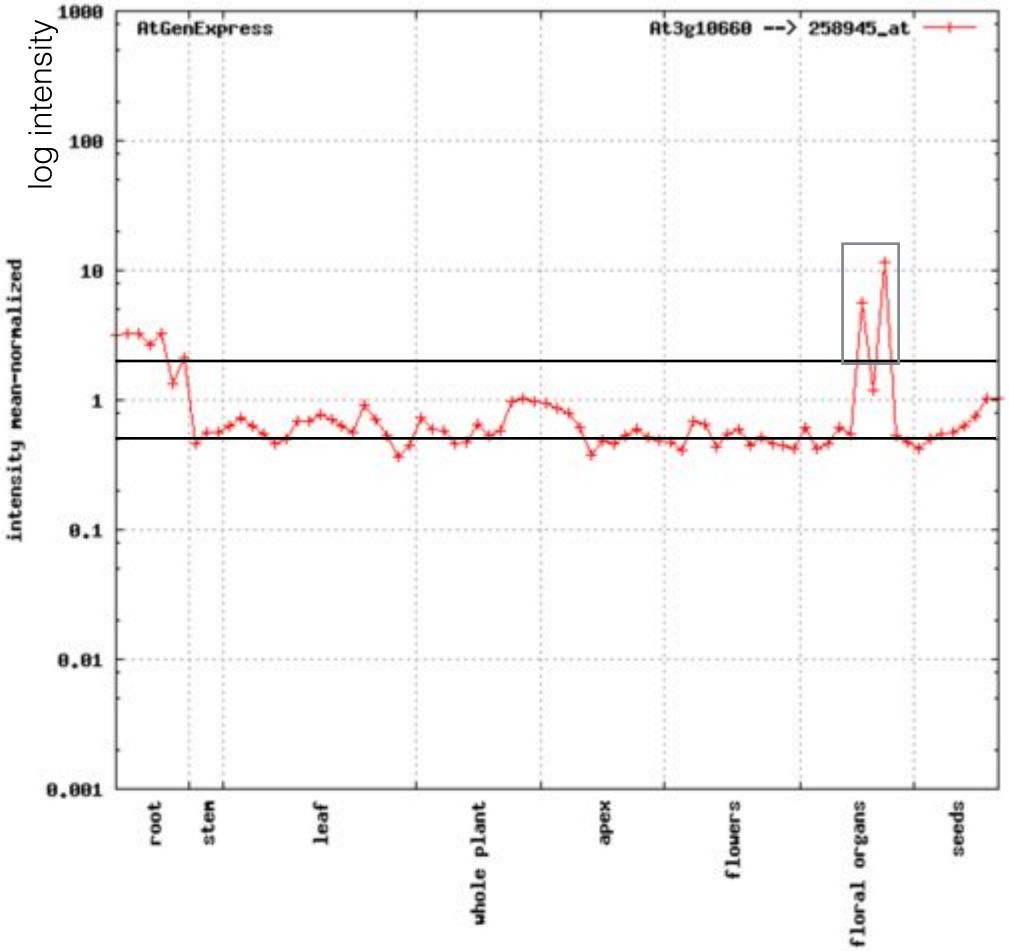
log intensity
intensity mean-normalized
1000
100
10
1
0.1
0.01
0.001
AtGenExpress
At3g10660 --> 258945_at
root
stem
leaf
whole plant
apex
flowers
floral organs
seeds

29

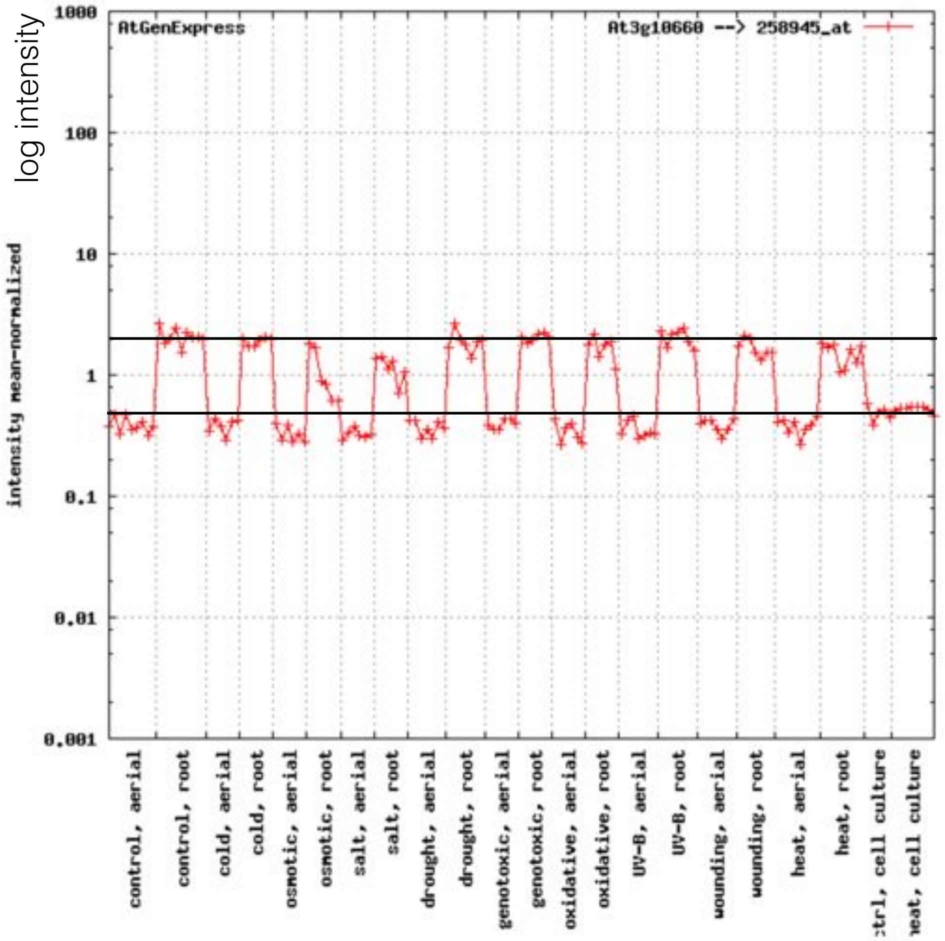
log intensity
intensity mean-normalized
1000
100
10
1
0.1
0.01
0.001
AtGenExpress
At3g10660 --> 258945_at
control, aerial
control, root
cold, aerial
cold, root
osmotic, aerial
osmotic, root
salt, aerial
salt, root
drought, aerial
drought, root
genotoxic, aerial
genotoxic, root
oxidative, aerial
oxidative, root
UV-B, aerial
UV-B, root
wounding, aerial
wounding, root
heat, aerial
heat, root

30

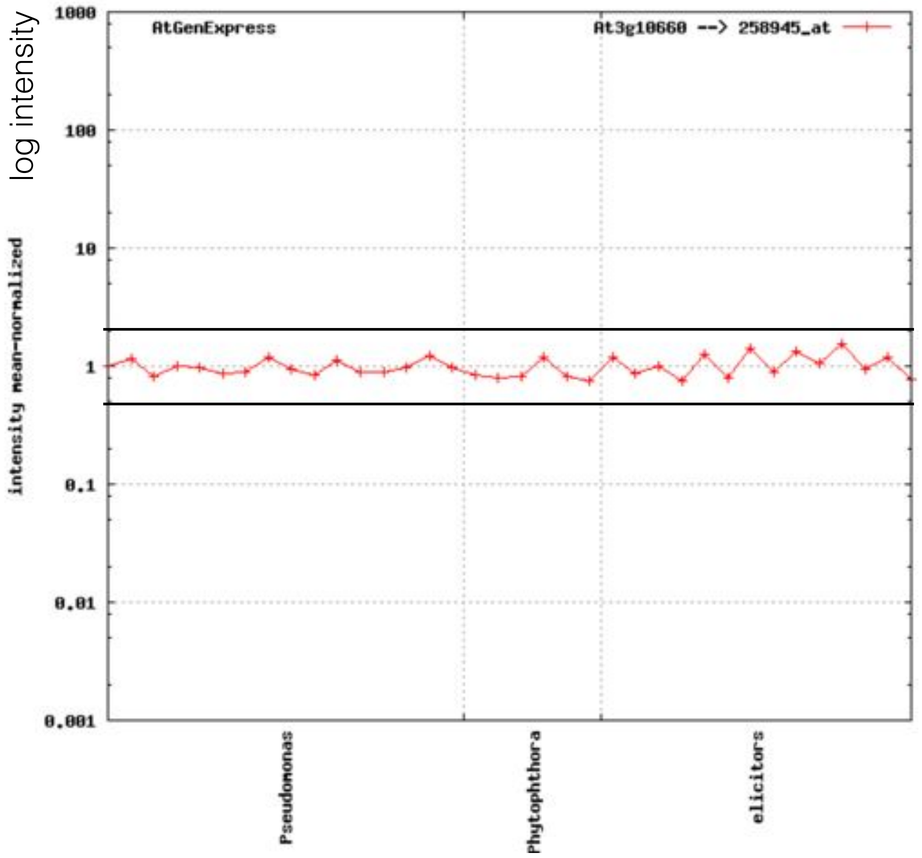
log intensity
intensity mean-normalized
1000
100
10
1
0.1
0.01
0.001
AtGenExpress
At3g10660 --> 258945_at
Pseudomonas
Phytophthora
elicitors

31

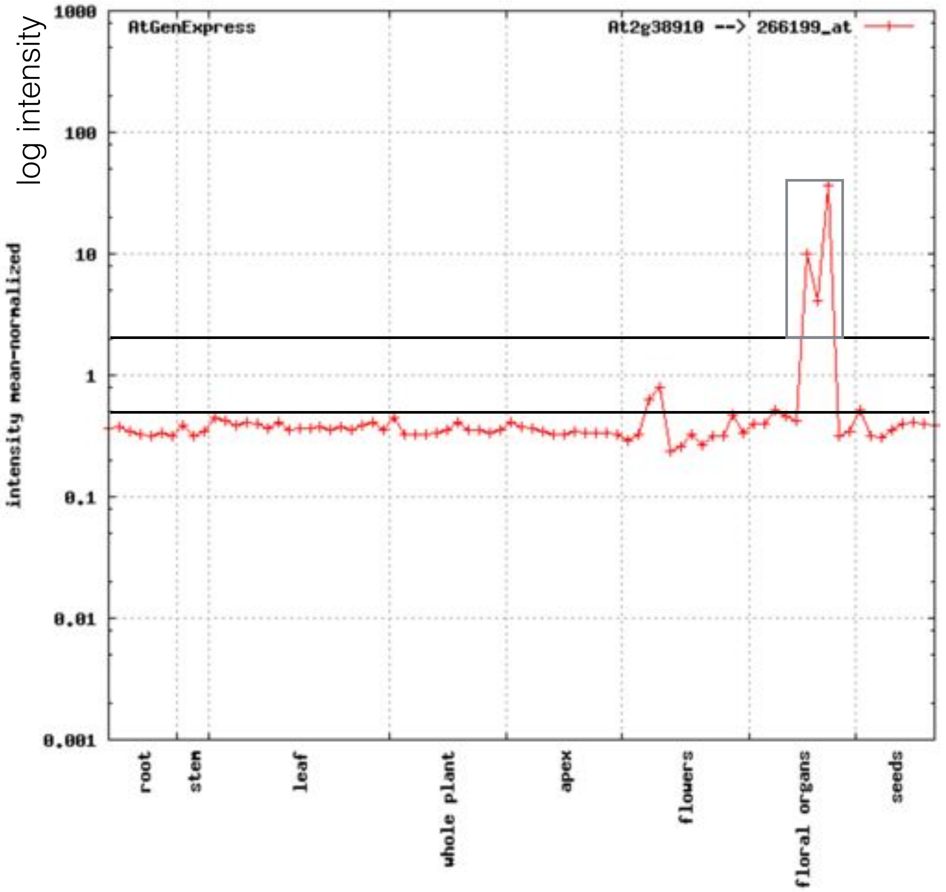
log intensity
intensity mean-normalized
AtGenExpress
At2g38910 --> 266199_at
1000
100
10
1
0.1
0.01
0.001
root
stem
leaf
whole plant
apex
flowers
floral organs
seeds

32

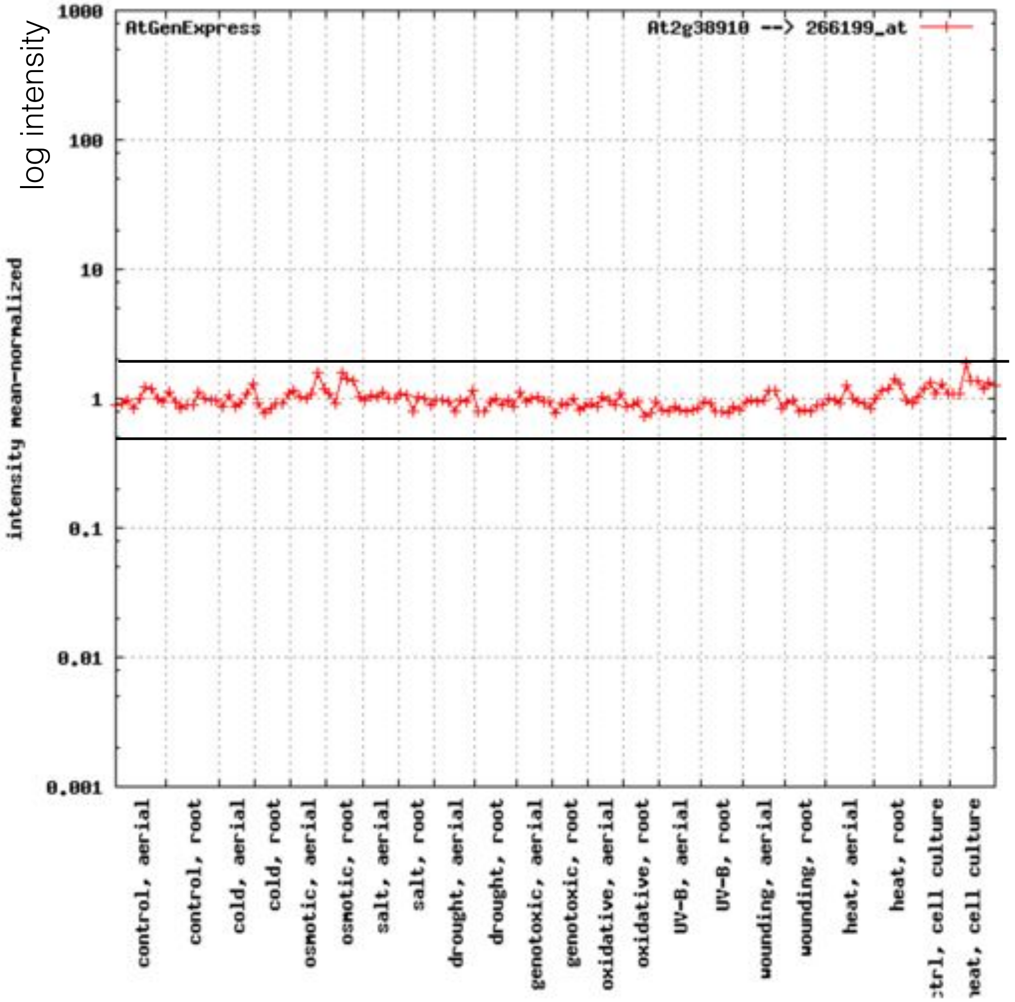
log intensity
intensity mean-normalized
AtGenExpress
At2g38910 --> 266199_at
1000
100
10
1
0.1
0.01
0.001
control, aerial
control, root
cold, aerial
cold, root
osmotic, aerial
osmotic, root
salt, aerial
salt, root
drought, aerial
drought, root
genotoxic, aerial
genotoxic, root
oxidative, aerial
oxidative, root
UV-B, aerial
UV-B, root
wounding, aerial
wounding, root
heat, aerial
heat, root
ctrl, cell culture
heat, cell culture

33

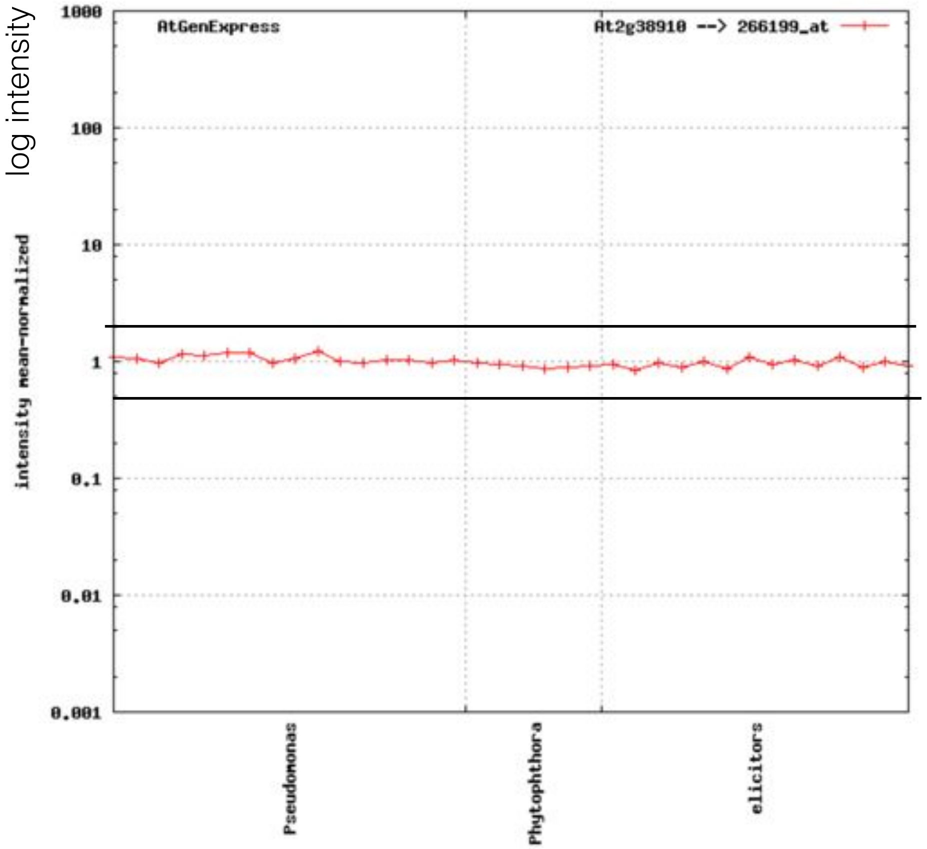
log intensity
intensity mean-normalized
AtGenExpress
At2g38910 --> 266199_at
1000
100
10
1
0.1
0.01
0.001
Pseudomonas
Phytophthora
elicitors

34

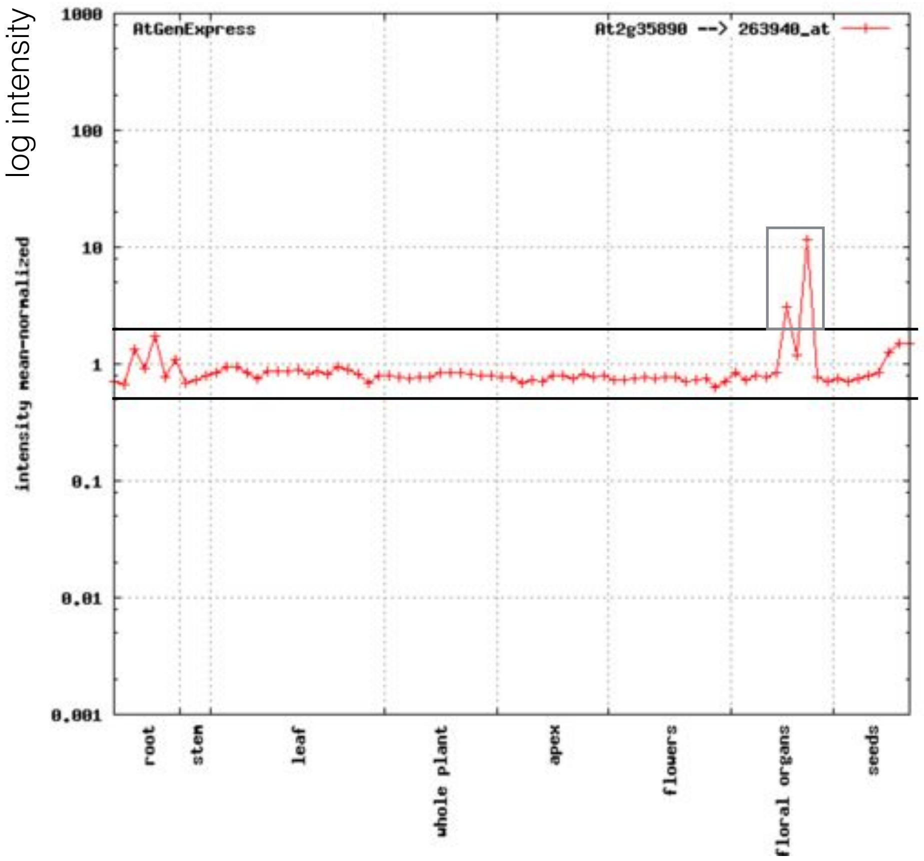
log intensity
intensity mean-normalized
AtGenExpress
At2g35890 --> 263940_at
1000
100
10
1
0.1
0.01
0.001
root
stem
leaf
whole plant
apex
flowers
floral organs
seeds

35

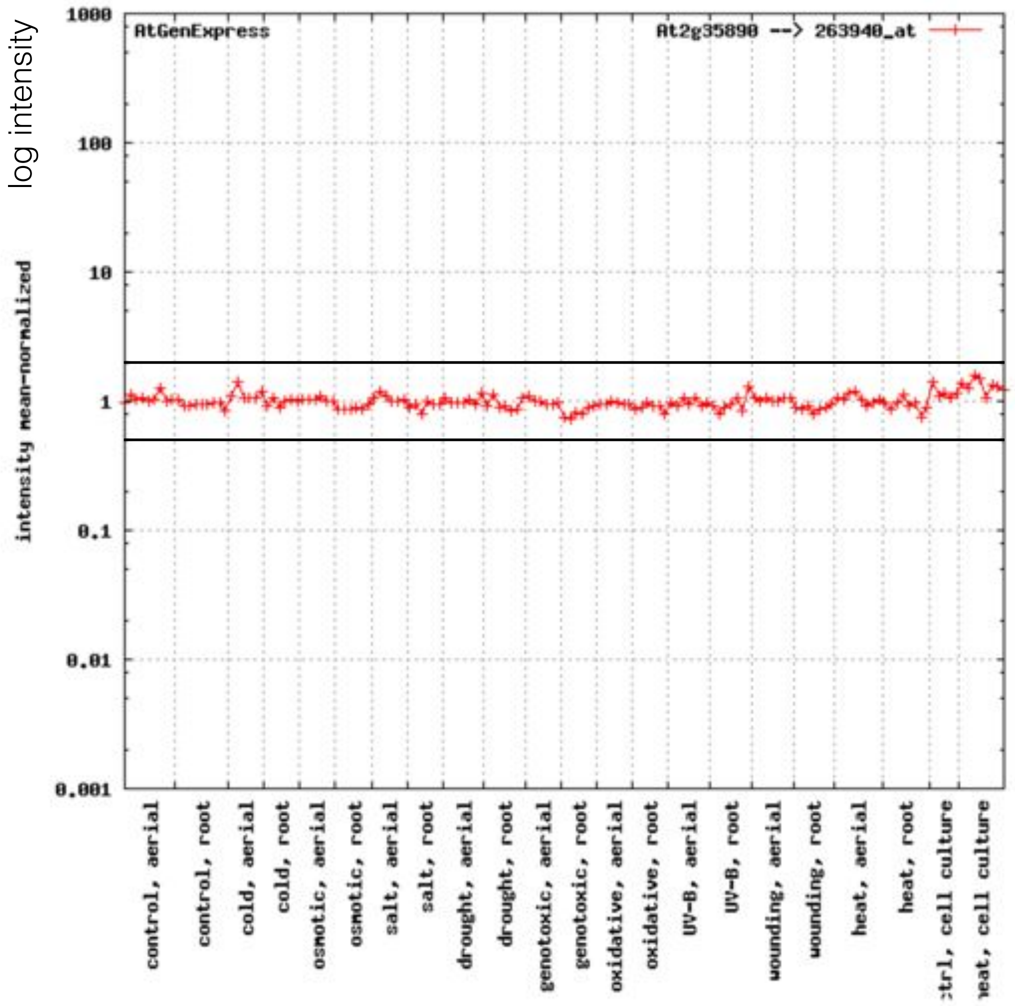
log intensity
intensity mean-normalized
AtGenExpress
At2g35890 --> 263940_at
1000
100
10
1
0.1
0.01
0.001
control, aerial
control, root
cold, aerial
cold, root
osmotic, aerial
osmotic, root
salt, aerial
salt, root
drought, aerial
drought, root
genotoxic, aerial
genotoxic, root
oxidative, aerial
oxidative, root
UV-B, aerial
UV-B, root
wounding, aerial
wounding, root
heat, aerial
heat, root
ctrl, cell culture
heat, cell culture

36

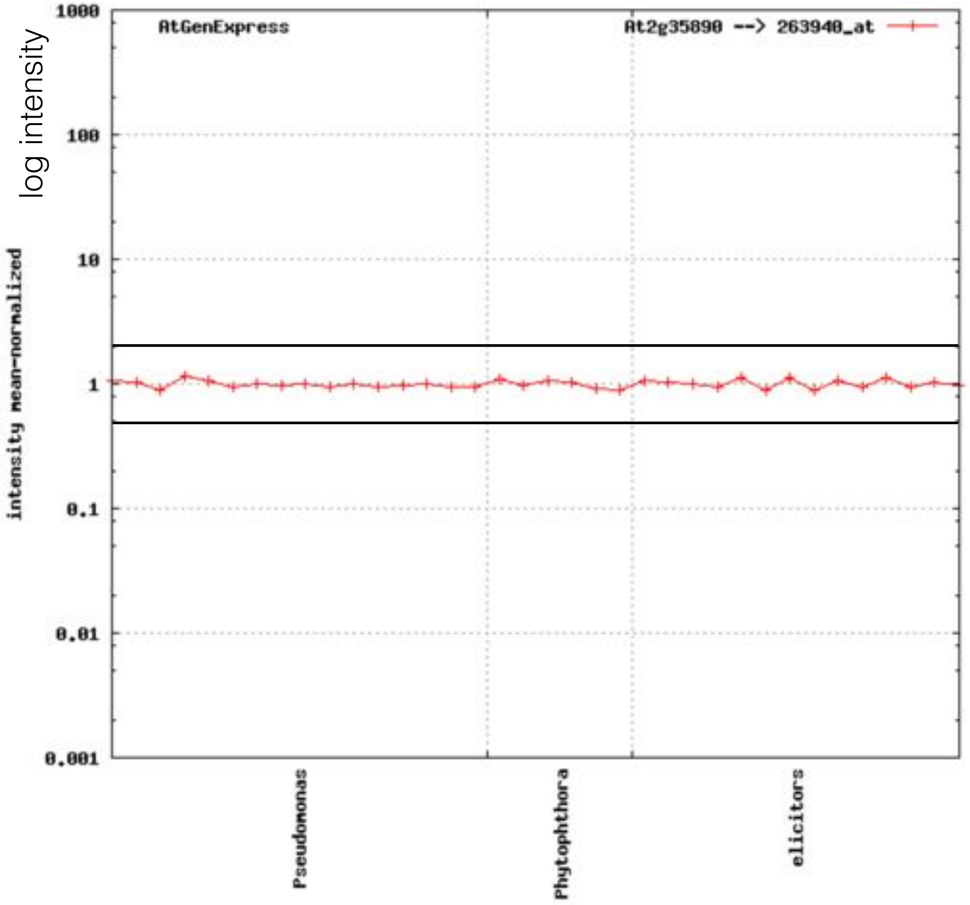
log intensity
intensity mean-normalized
AtGenExpress
At2g35890 --> 263940_at
1000
100
10
1
0.1
0.01
0.001
Pseudomonas
Phytophthora
elicitors

37

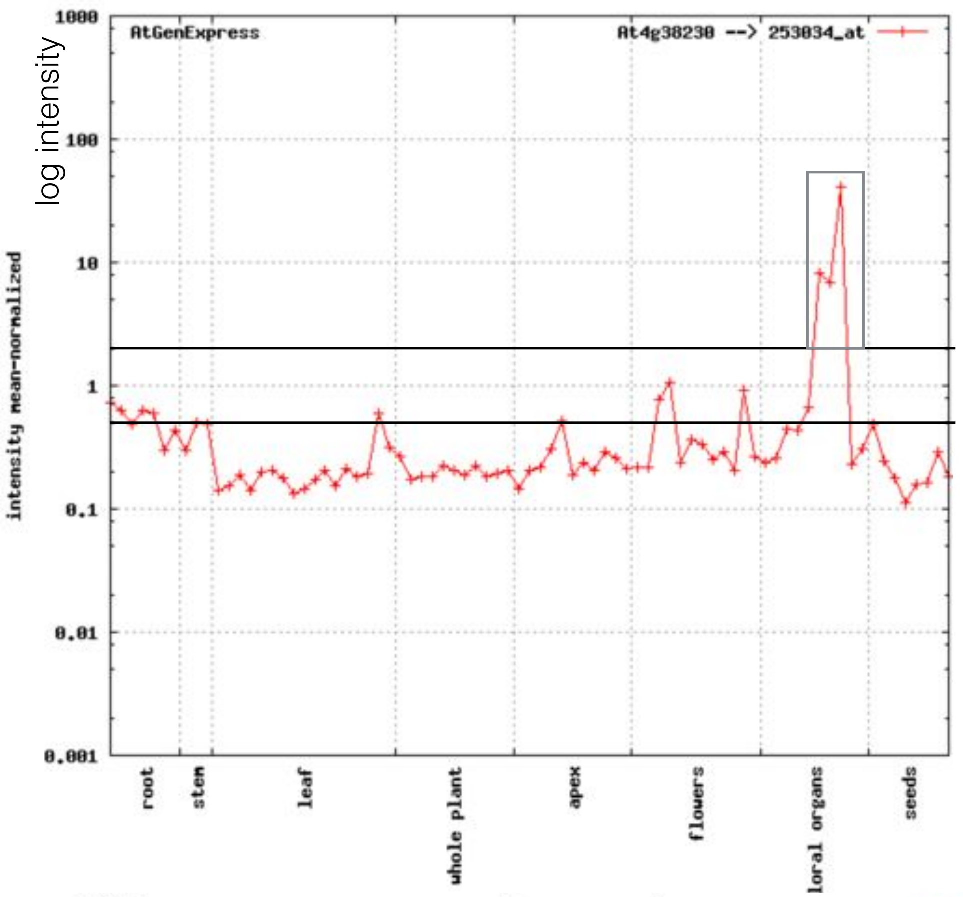
AtGenExpress
At4g38230 --> 253034_at
log intensity
intensity mean-normalized
1000
100
10
1
0.1
0.01
0.001
root
stem
leaf
whole plant
apex
flowers
loral organs
seeds

38

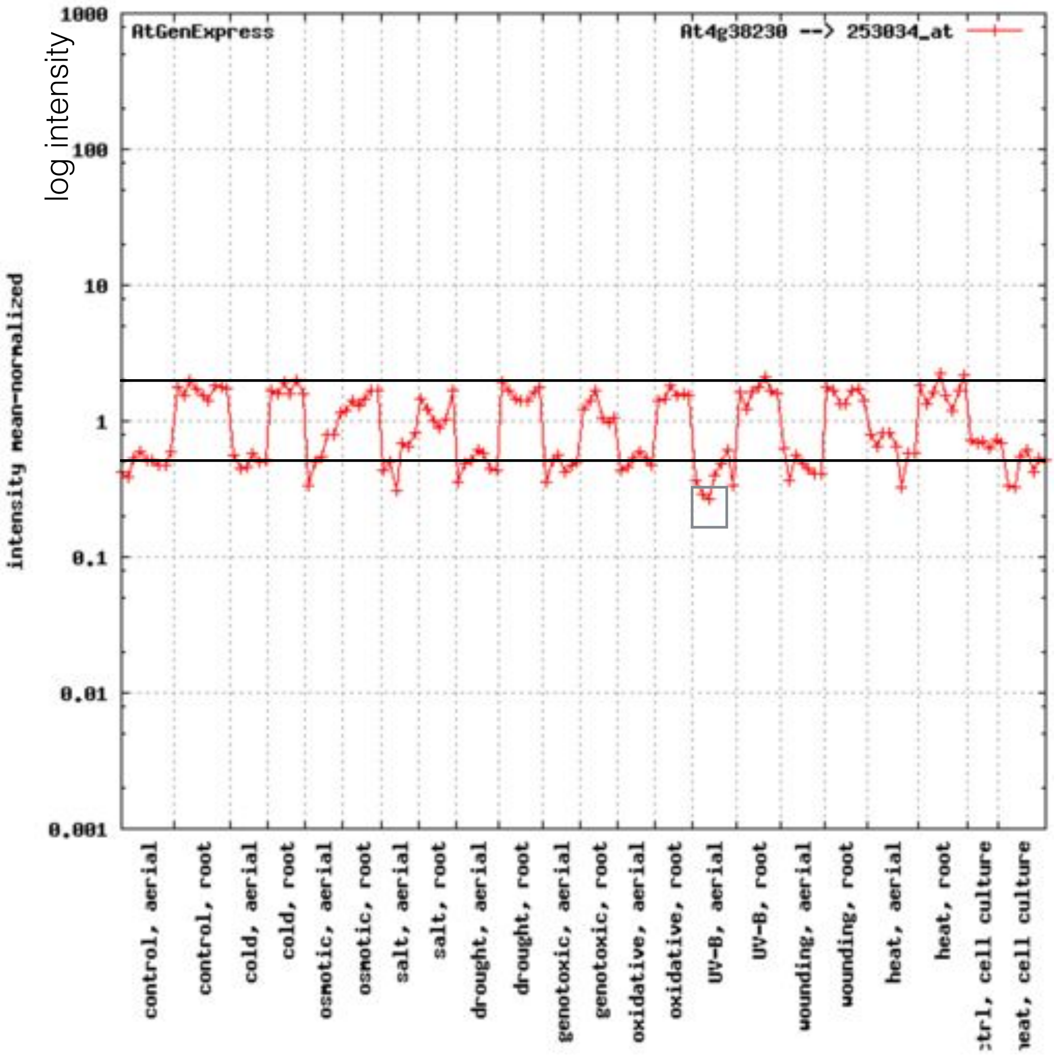
AtGenExpress
At4g38230 --> 253034_at
log intensity
intensity mean-normalized
1000
100
10
1
0.1
0.01
0.001
control, aerial
control, root
cold, aerial
cold, root
osmotic, aerial
osmotic, root
salt, aerial
salt, root
drought, aerial
drought, root
genotoxic, aerial
genotoxic, root
oxidative, aerial
oxidative, root
UV-B, aerial
UV-B, root
wounding, aerial
wounding, root
heat, aerial
heat, root
ctrl, cell culture
heat, cell culture

39

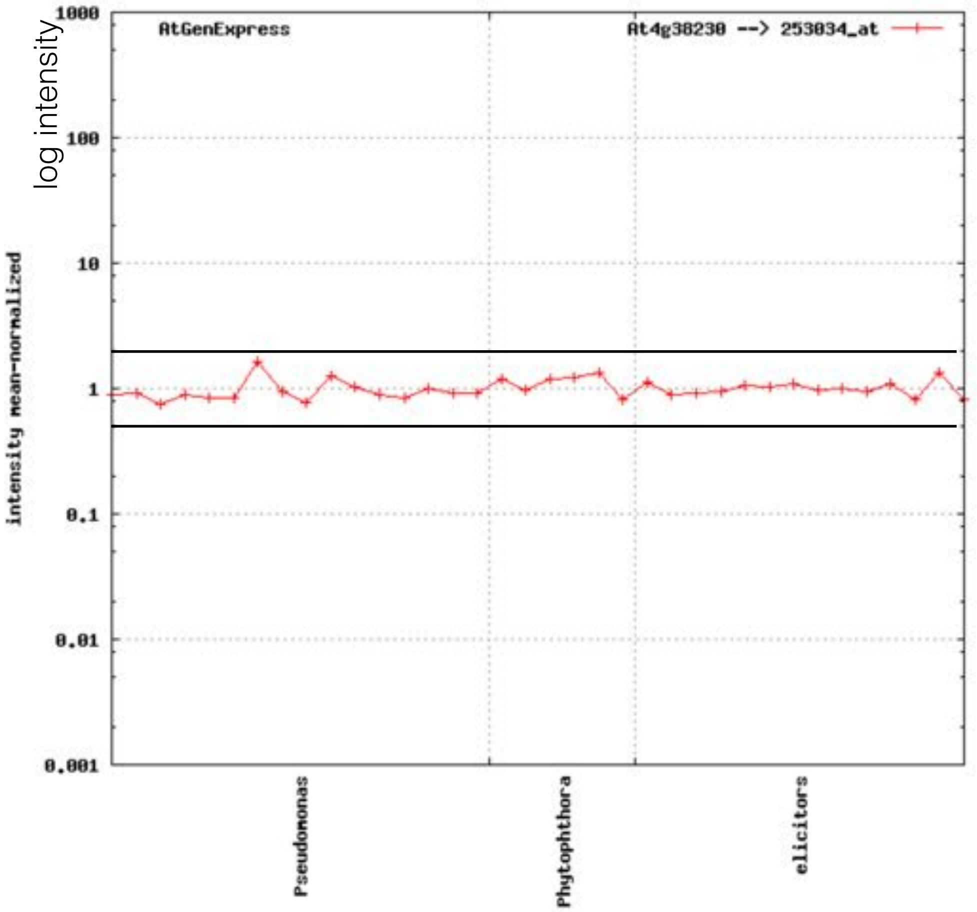
AtGenExpress
At4g38230 --> 253034_at
log intensity
intensity mean-normalized
1000
100
10
1
0.1
0.01
0.001
Pseudomonas
Phytophthora
elicitors

40

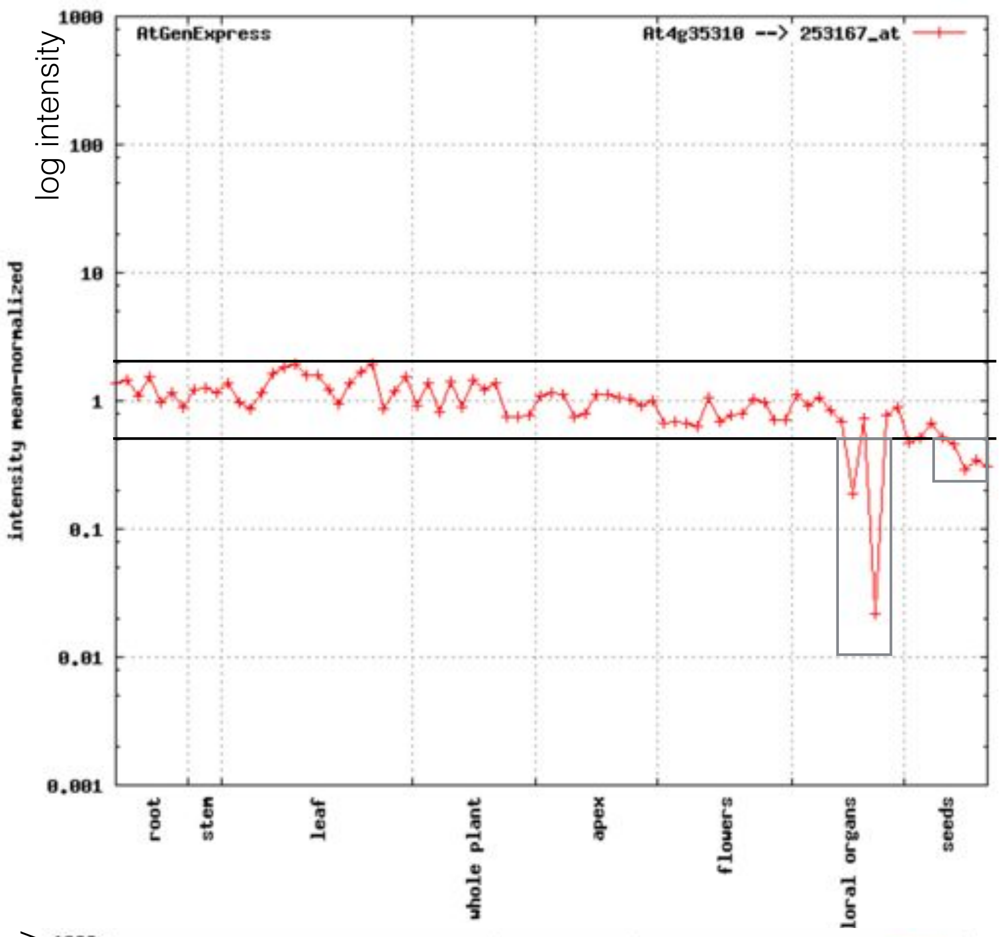
AtGenExpress
At4g35310 --> 253167_at
log intensity
intensity mean-normalized
1000
100
10
1
0.1
0.01
0.001
root
stem
leaf
whole plant
apex
flowers
loral organs
seeds

41

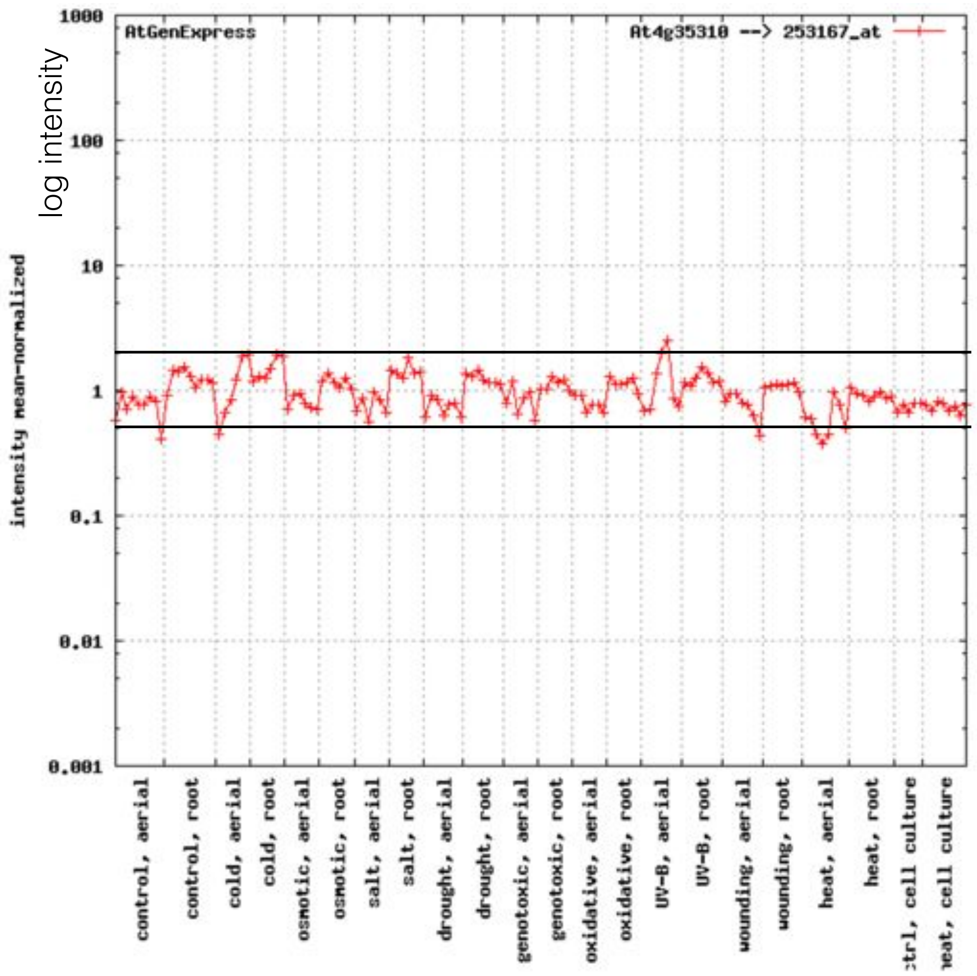
AtGenExpress
At4g35310 --> 253167_at
log intensity
intensity mean-normalized
1000
100
10
1
0.1
0.01
0.001
control, aerial
control, root
cold, aerial
cold, root
osmotic, aerial
osmotic, root
salt, aerial
salt, root
drought, aerial
drought, root
genotoxic, aerial
genotoxic, root
oxidative, aerial
oxidative, root
UV-B, aerial
UV-B, root
wounding, aerial
wounding, root
heat, aerial
heat, root
:trl, cell culture
neat, cell culture

42

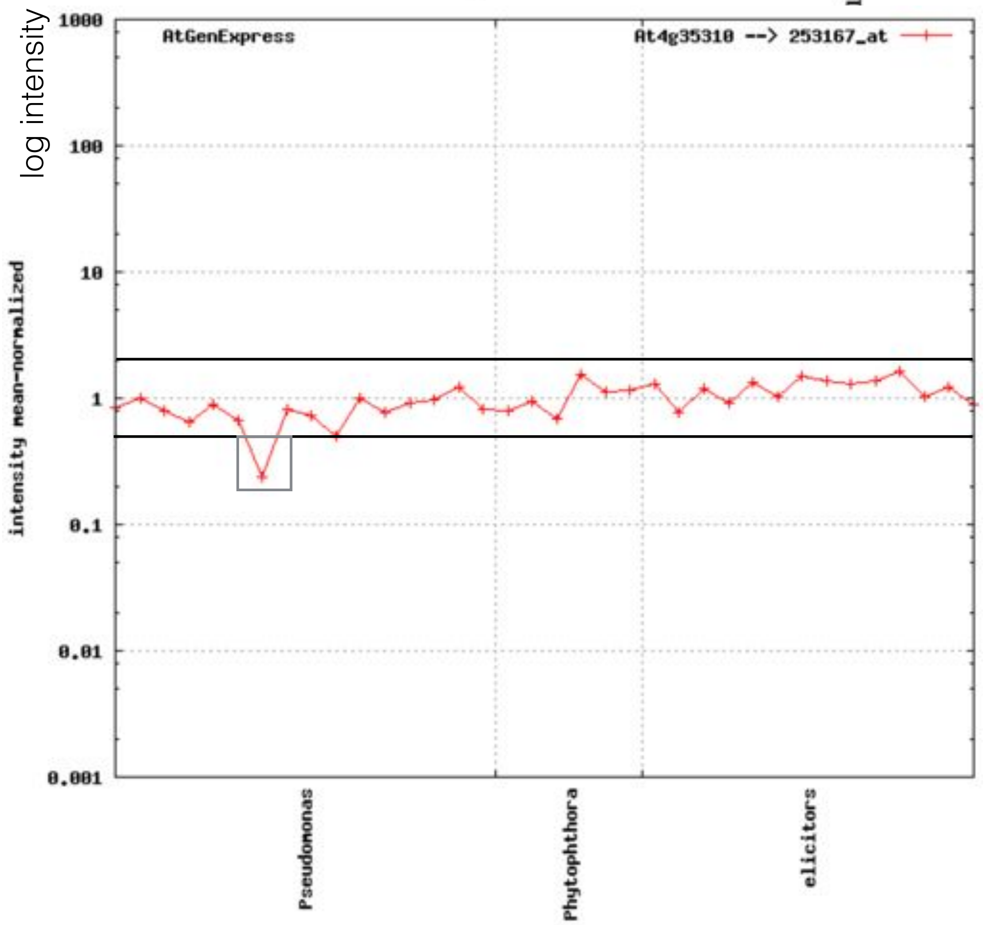
AtGenExpress
At4g35310 --> 253167_at
log intensity
intensity mean-normalized
1000
100
10
1
0.1
0.01
0.001
Pseudomonas
Phytophthora
elicitors

43

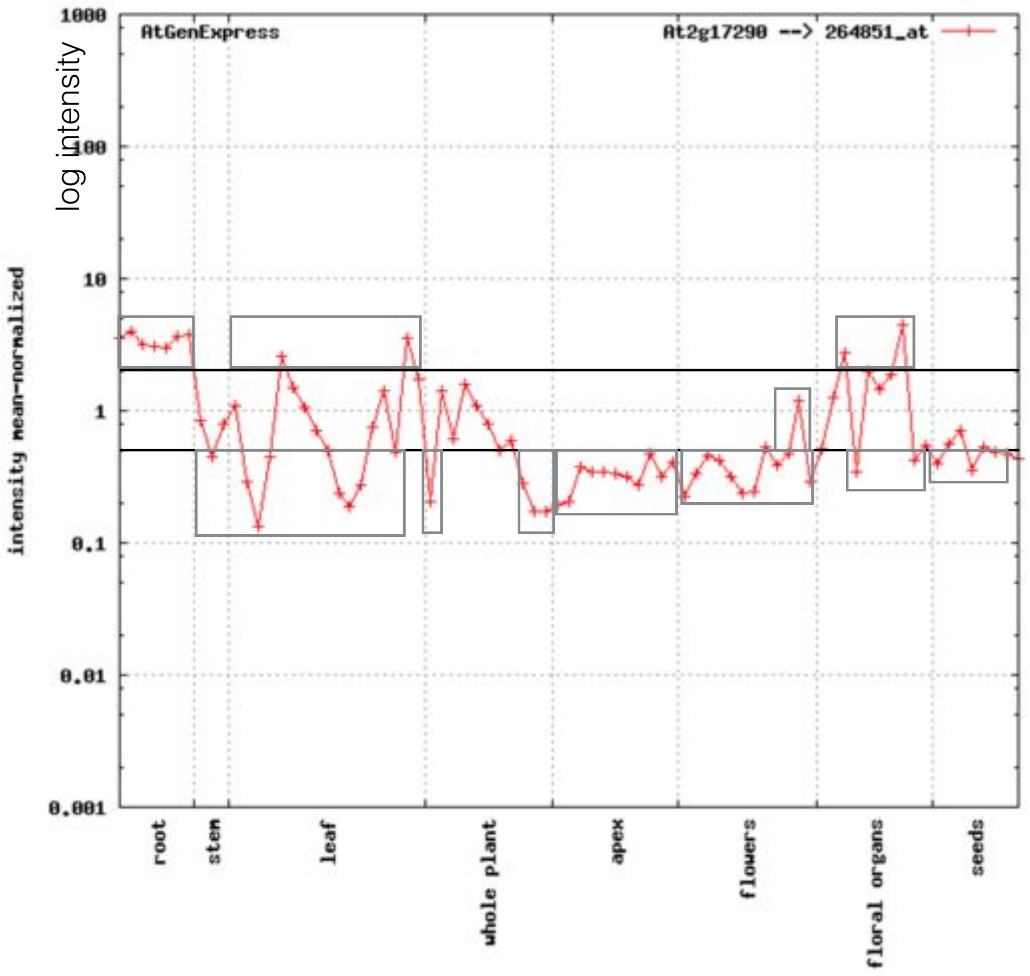
AtGenExpress
At2g17290 --> 264851_at
log intensity
intensity mean-normalized
1000
100
10
1
0.1
0.01
0.001
root
stem
leaf
whole plant
apex
flowers
floral organs
seeds

44

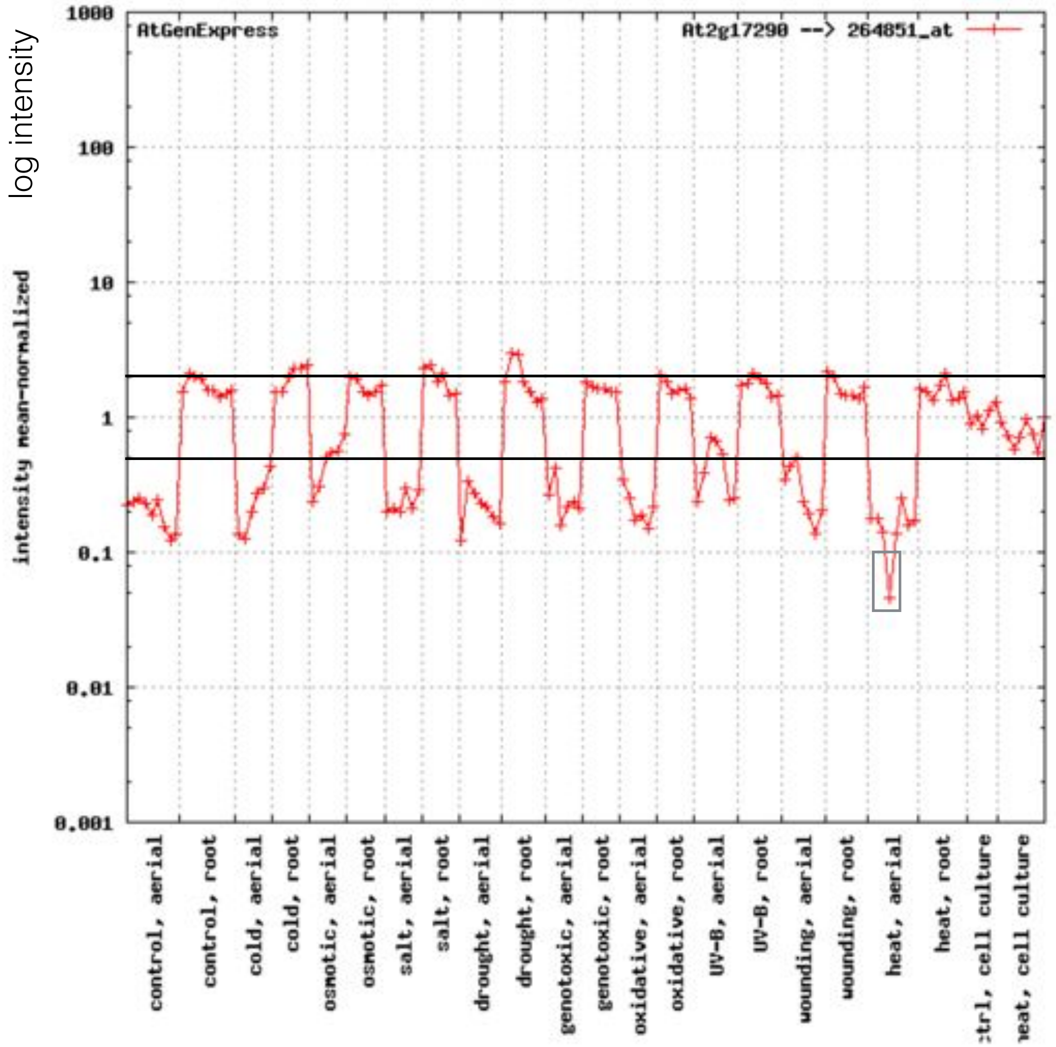
AtGenExpress
At2g17290 --> 264851_at
log intensity
intensity mean-normalized
1000
100
10
1
0.1
0.01
0.001
control, aerial
control, root
cold, aerial
cold, root
osmotic, aerial
osmotic, root
salt, aerial
salt, root
drought, aerial
drought, root
genotoxic, aerial
genotoxic, root
oxidative, aerial
oxidative, root
UV-B, aerial
UV-B, root
wounding, aerial
wounding, root
heat, aerial
heat, root

45

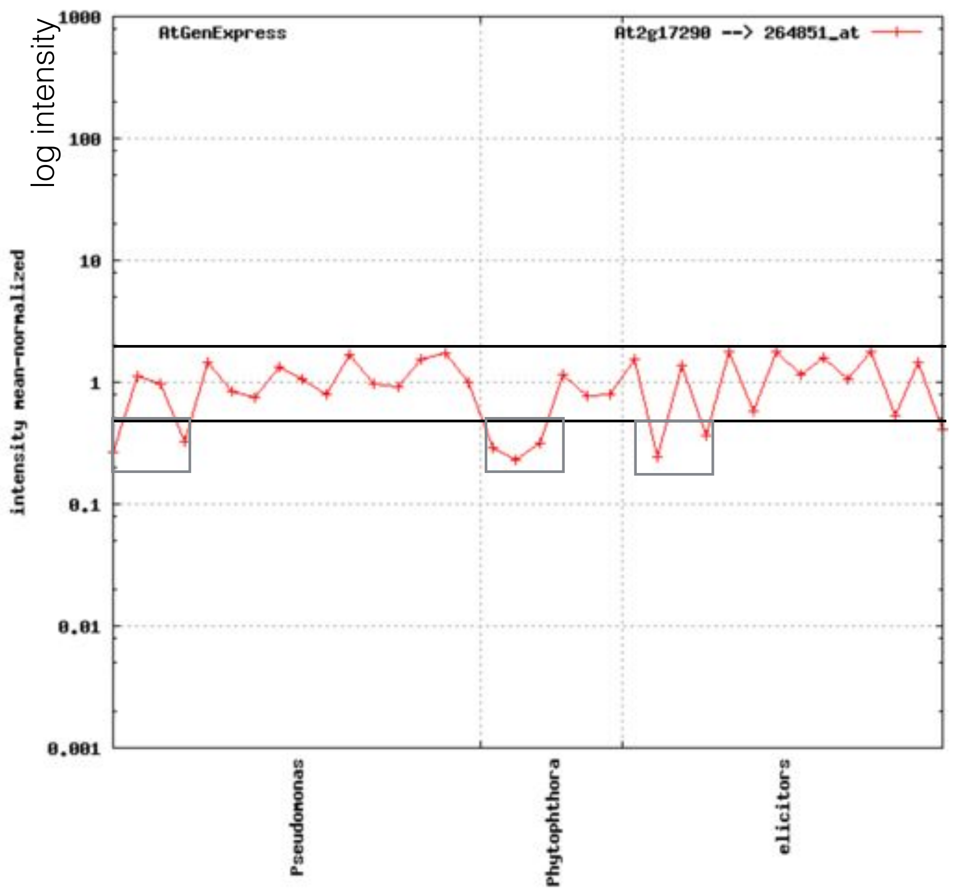
AtGenExpress
At2g17290 --> 264851_at
log intensity
intensity mean-normalized
1000
100
10
1
0.1
0.01
0.001
Pseudomonas
Phytophthora
elicitors

46

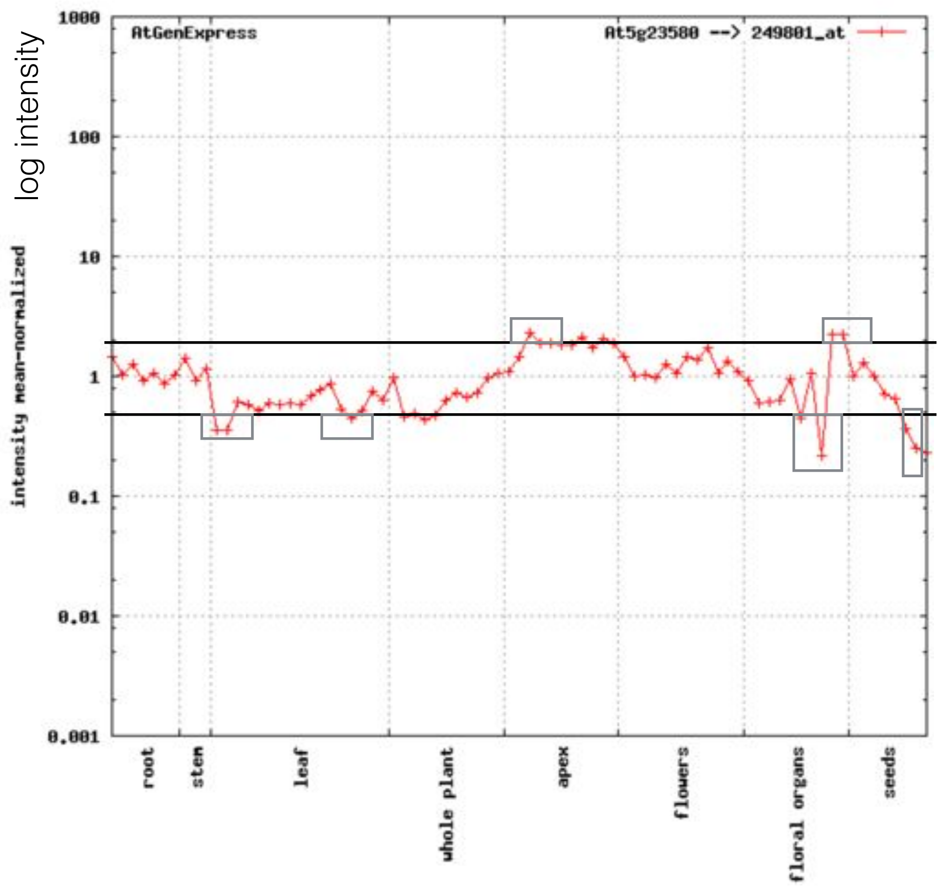
log intensity
intensity mean-normalized
1000
100
10
1
0.1
0.01
0.001
AtGenExpress
At5g23580 --> 249801_at
root
stem
leaf
whole plant
apex
flowers
floral organs
seeds

47

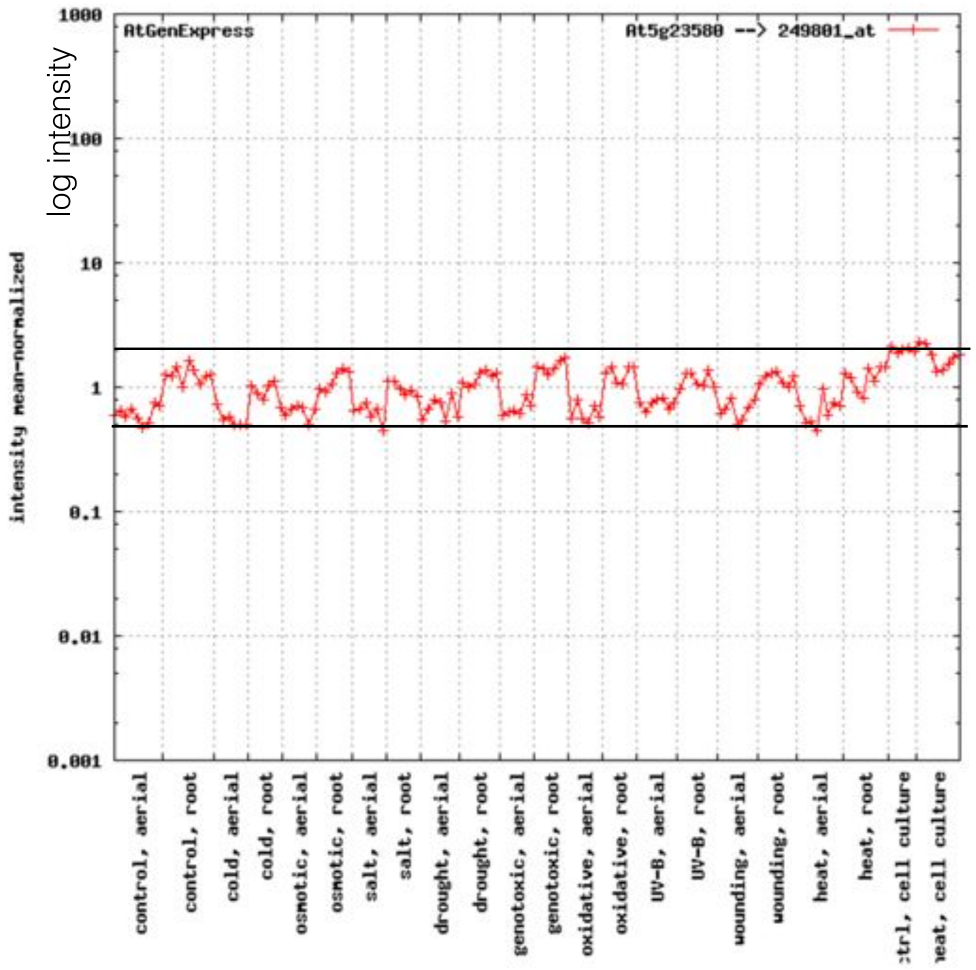
log intensity
intensity mean-normalized
1000
100
10
1
0.1
0.01
0.001
AtGenExpress
At5g23580 --> 249801_at
control, aerial
control, root
cold, aerial
cold, root
osmotic, aerial
osmotic, root
salt, aerial
salt, root
drought, aerial
drought, root
genotoxic, aerial
genotoxic, root
oxidative, aerial
oxidative, root
UV-B, aerial
UV-B, root
wounding, aerial
wounding, root
heat, aerial
heat, root
trl, cell culture
eat, cell culture

48

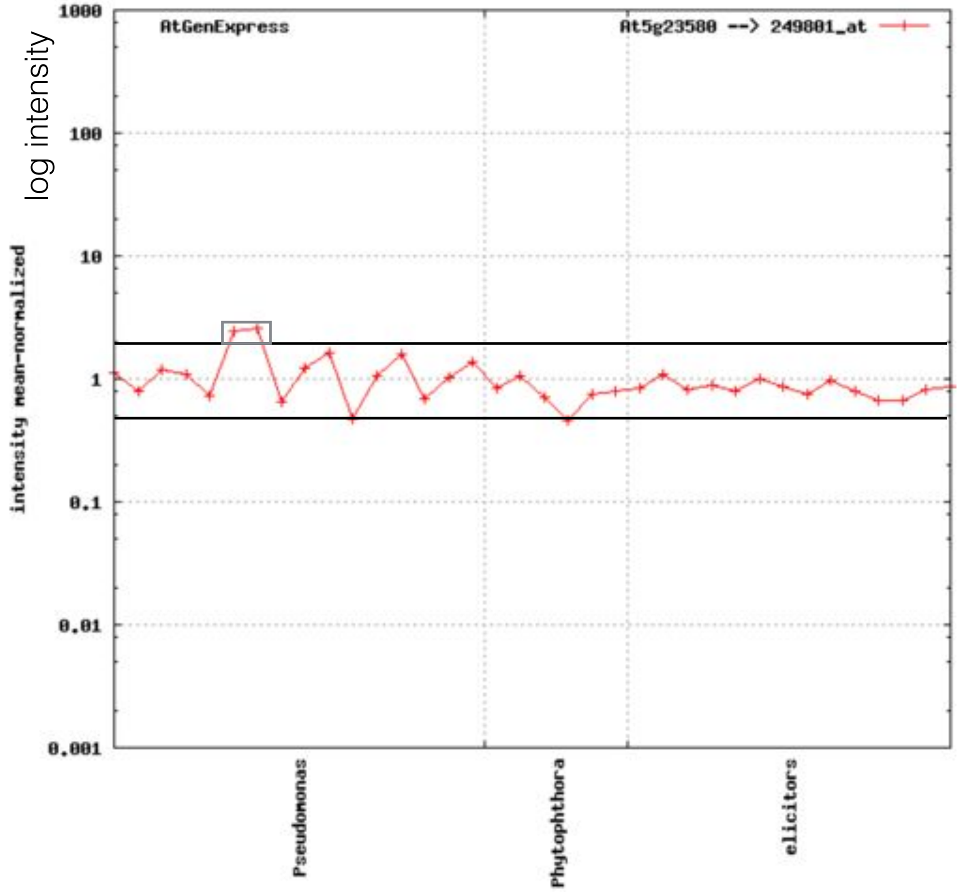
log intensity
intensity mean-normalized
1000
100
10
1
0.1
0.01
0.001
AtGenExpress
At5g23580 --> 249801_at
Pseudomonas
Phytophthora
elicitors

49

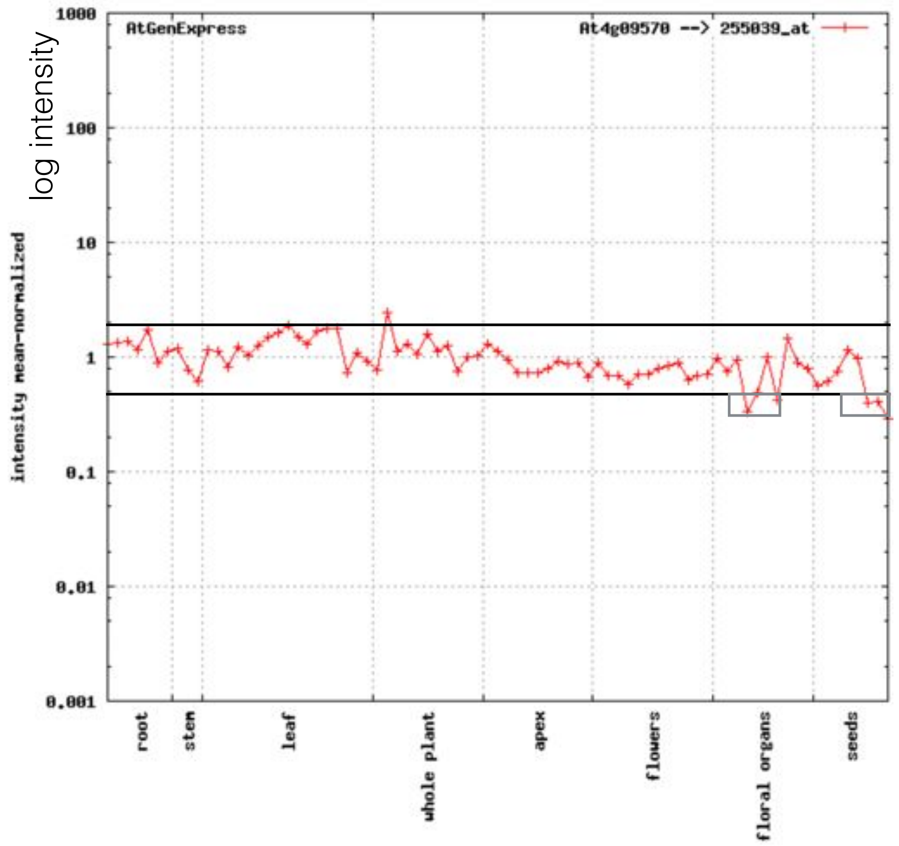
AtGenExpress
At4g09570 --> 255039_at
log intensity
intensity mean-normalized
1000
100
10
1
0.1
0.01
0.001
root
stem
leaf
whole plant
apex
flowers
floral organs
seeds

50

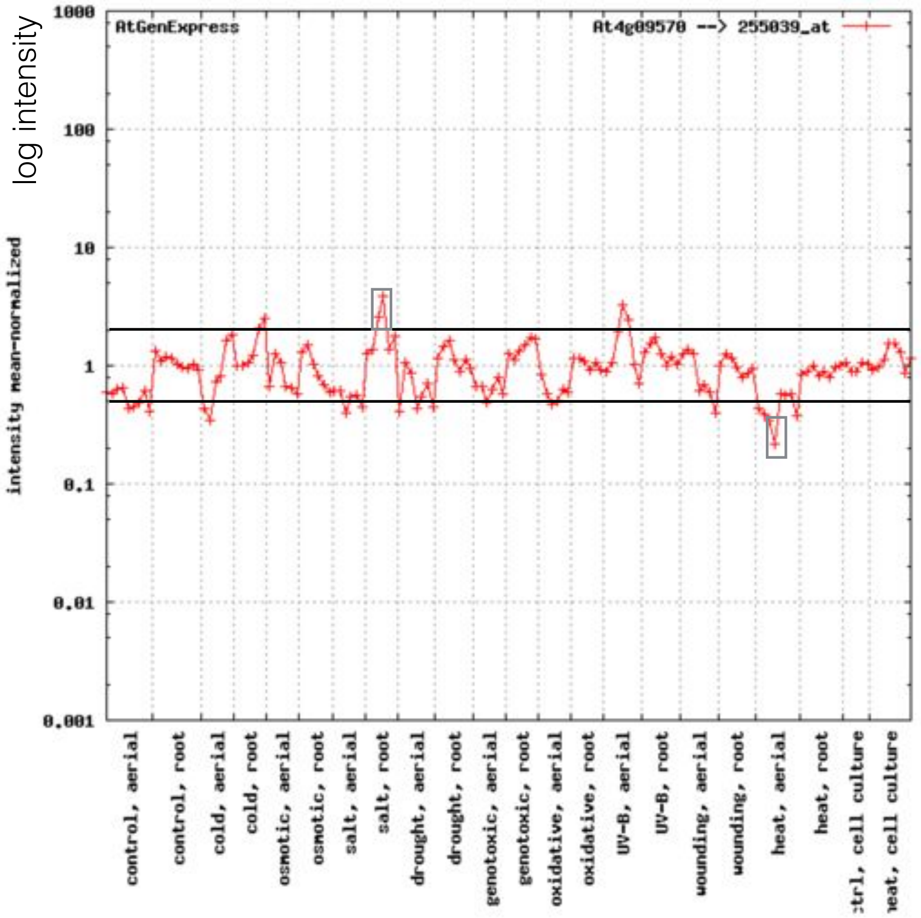
AtGenExpress
At4g09570 --> 255039_at
log intensity
intensity mean-normalized
1000
100
10
1
0.1
0.01
0.001
control, aerial
control, root
cold, aerial
cold, root
osmotic, aerial
osmotic, root
salt, aerial
salt, root
drought, aerial
drought, root
genotoxic, aerial
genotoxic, root
oxidative, aerial
oxidative, root
UV-B, aerial
UV-B, root
wounding, aerial
wounding, root
heat, aerial
heat, root

51

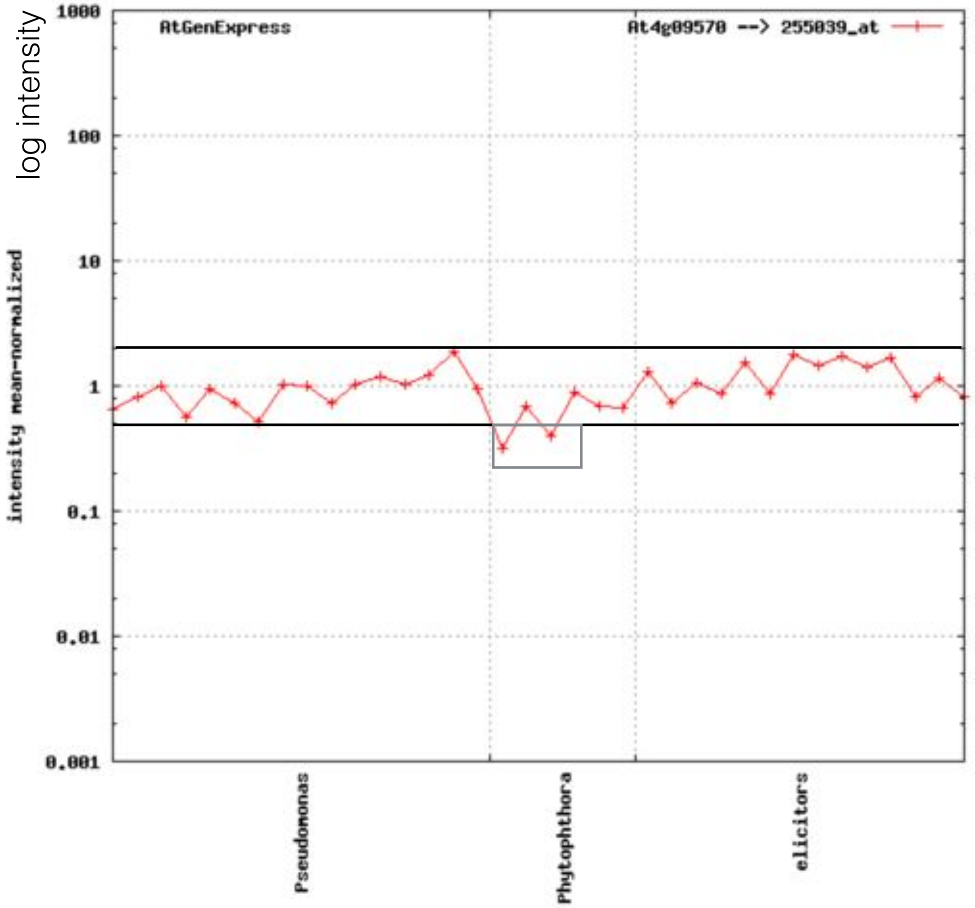
AtGenExpress
At4g09570 --> 255039_at
log intensity
intensity mean-normalized
1000
100
10
1
0.1
0.01
0.001
Pseudomonas
Phytophthora
elicitors

52

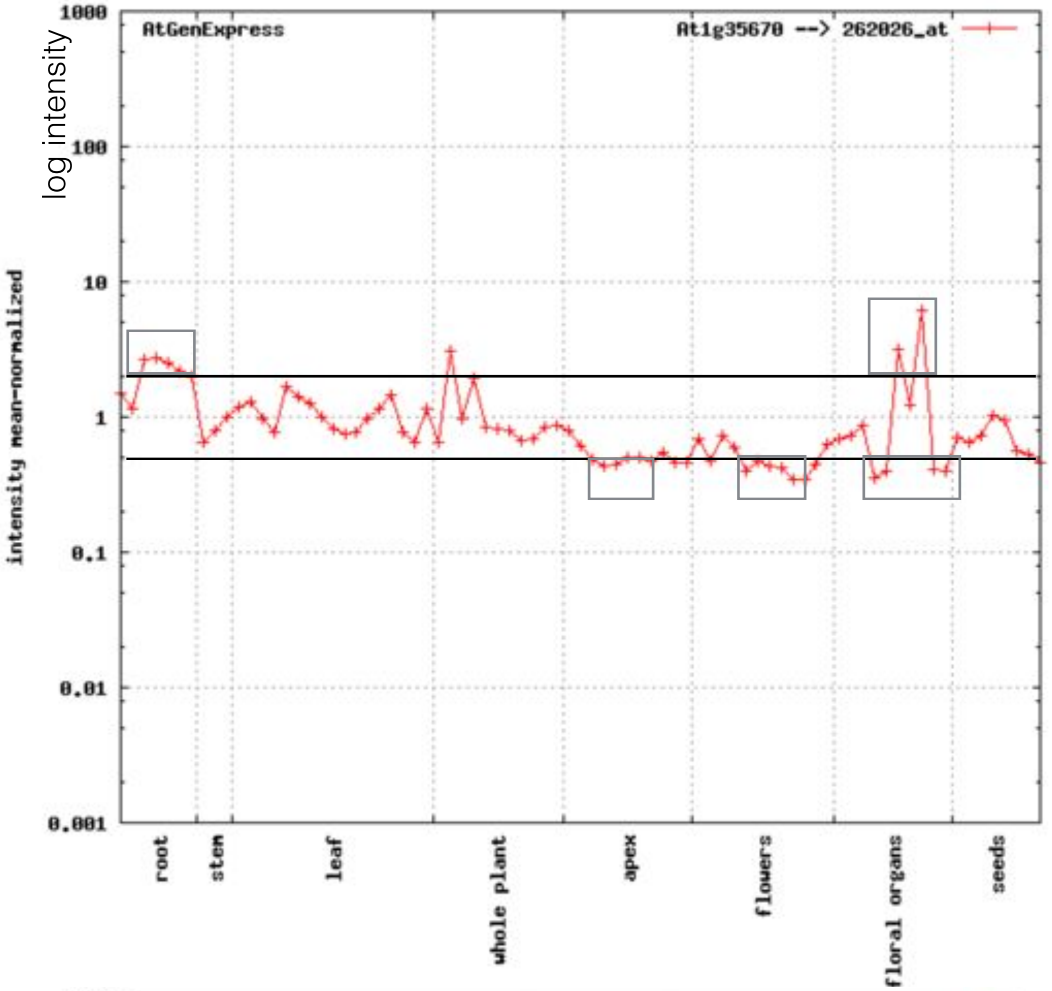
log intensity
intensity mean-normalized
AtGenExpress
At1g35670 --> 262026_at
1000
100
10
1
0.1
0.01
0.001
root
stem
leaf
whole plant
apex
flowers
floral organs
seeds

53

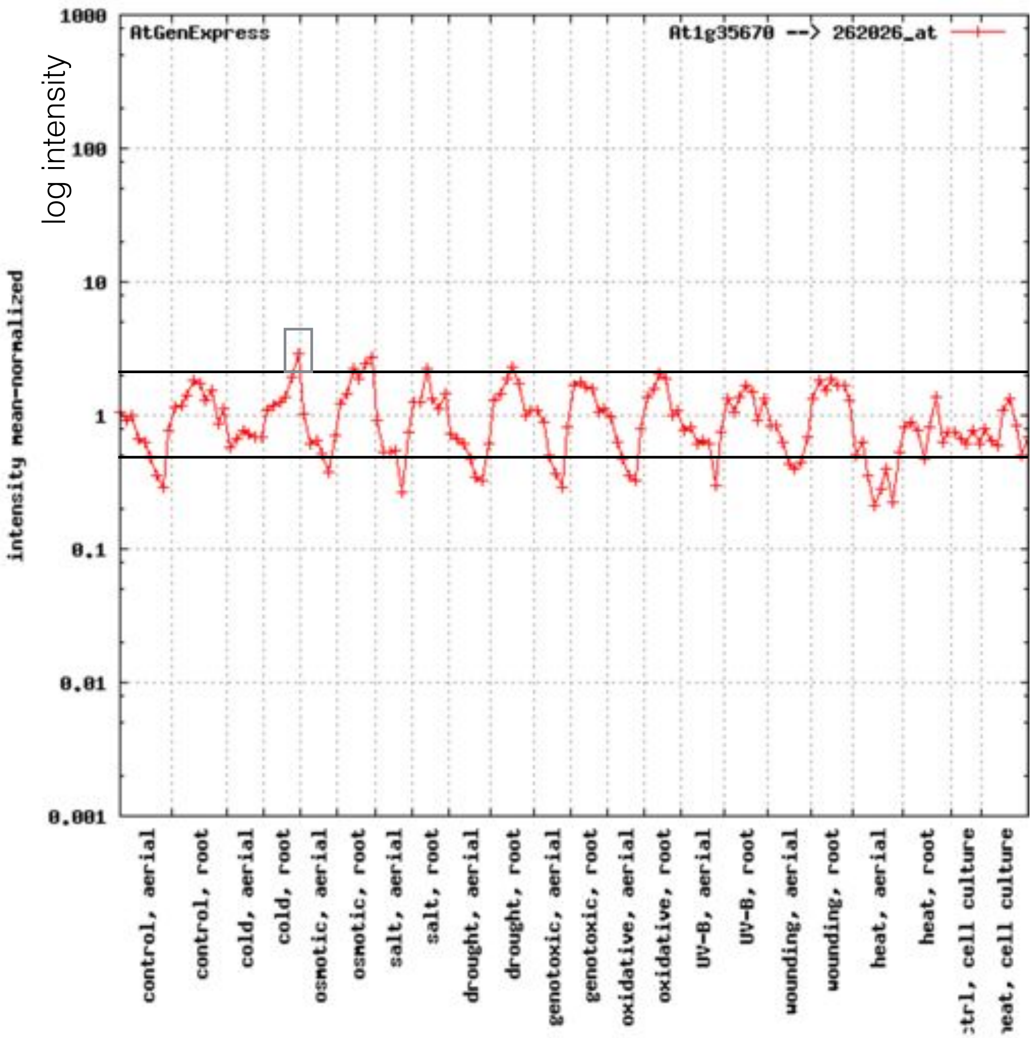
log intensity
intensity mean-normalized
AtGenExpress
At1g35670 --> 262026_at
1000
100
10
1
0.1
0.01
0.001
control, aerial
control, root
cold, aerial
cold, root
osmotic, aerial
osmotic, root
salt, aerial
salt, root
drought, aerial
drought, root
genotoxic, aerial
genotoxic, root
oxidative, aerial
oxidative, root
UV-B, aerial
UV-B, root
wounding, aerial
wounding, root
heat, aerial
heat, root

54

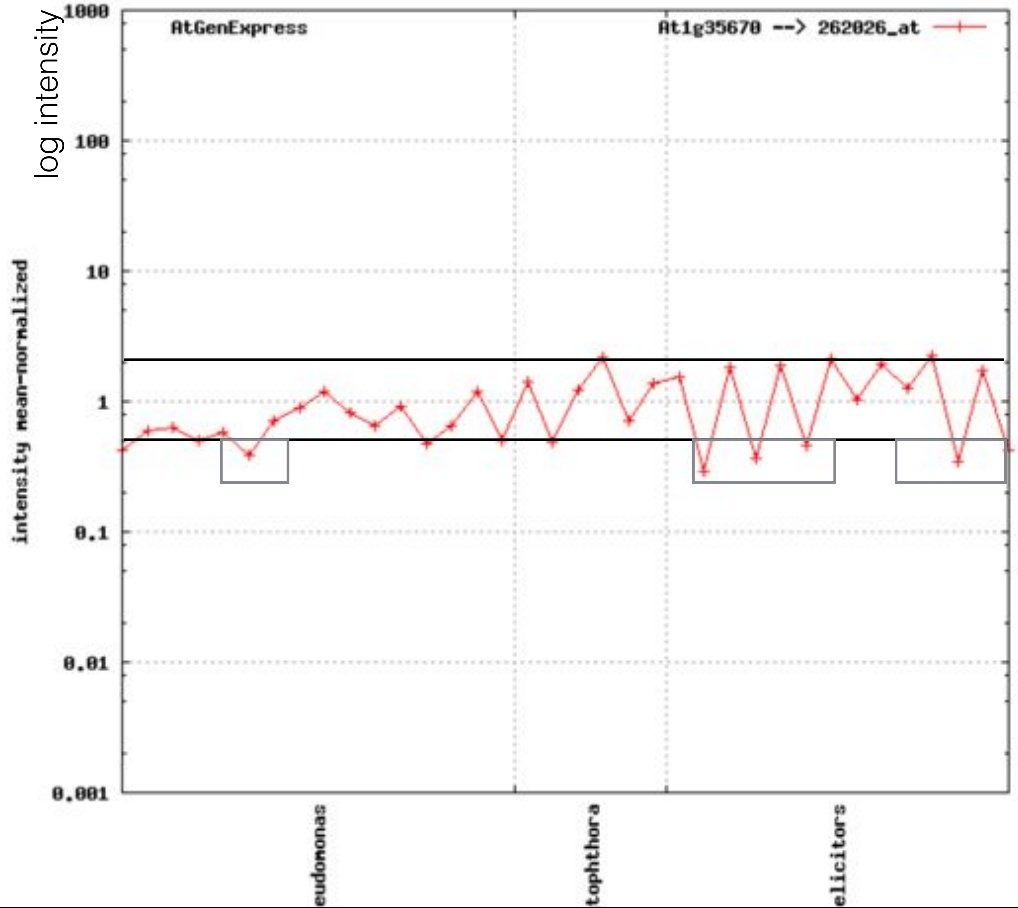
log intensity
intensity mean-normalized
AtGenExpress
At1g35670 --> 262026_at
1000
100
10
1
0.1
0.01
0.001

55

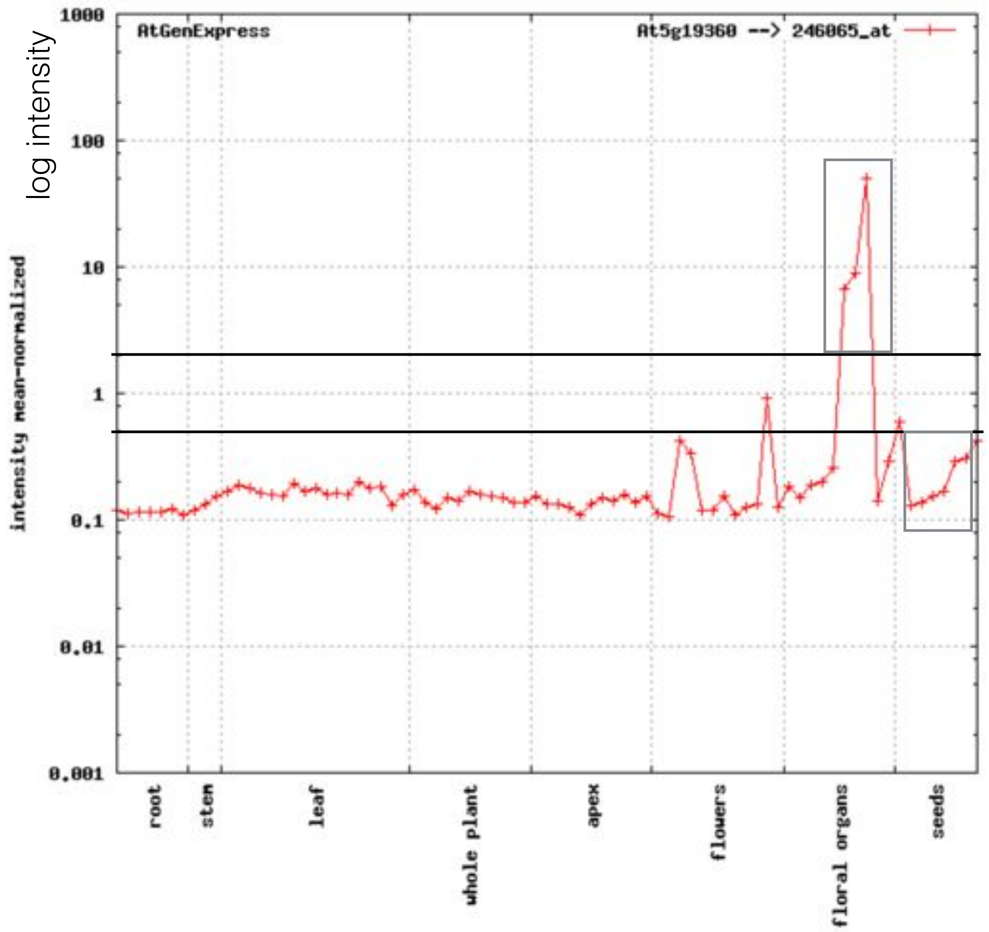
AtGenExpress
At5g19360 --> 246065_at
log intensity
intensity mean-normalized
1000
100
10
1
0.1
0.01
0.001
root
stem
leaf
whole plant
apex
flowers
floral organs
seeds

56

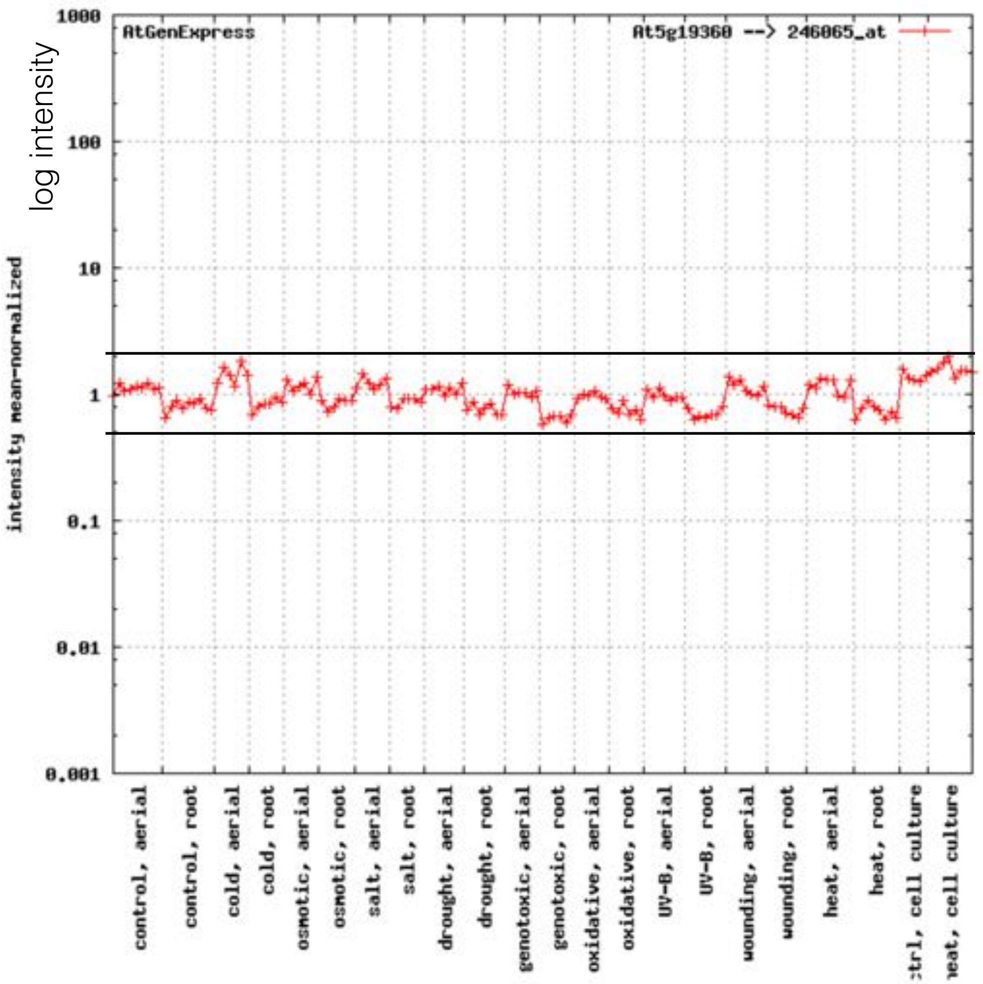
AtGenExpress
At5g19360 --> 246065_at
log intensity
intensity mean-normalized
1000
100
10
1
0.1
0.01
0.001
control, aerial
control, root
cold, aerial
cold, root
osmotic, aerial
osmotic, root
salt, aerial
salt, root
drought, aerial
drought, root
genotoxic, aerial
genotoxic, root
oxidative, aerial
oxidative, root
UV-B, aerial
UV-B, root
wounding, aerial
wounding, root
heat, aerial
heat, root

57

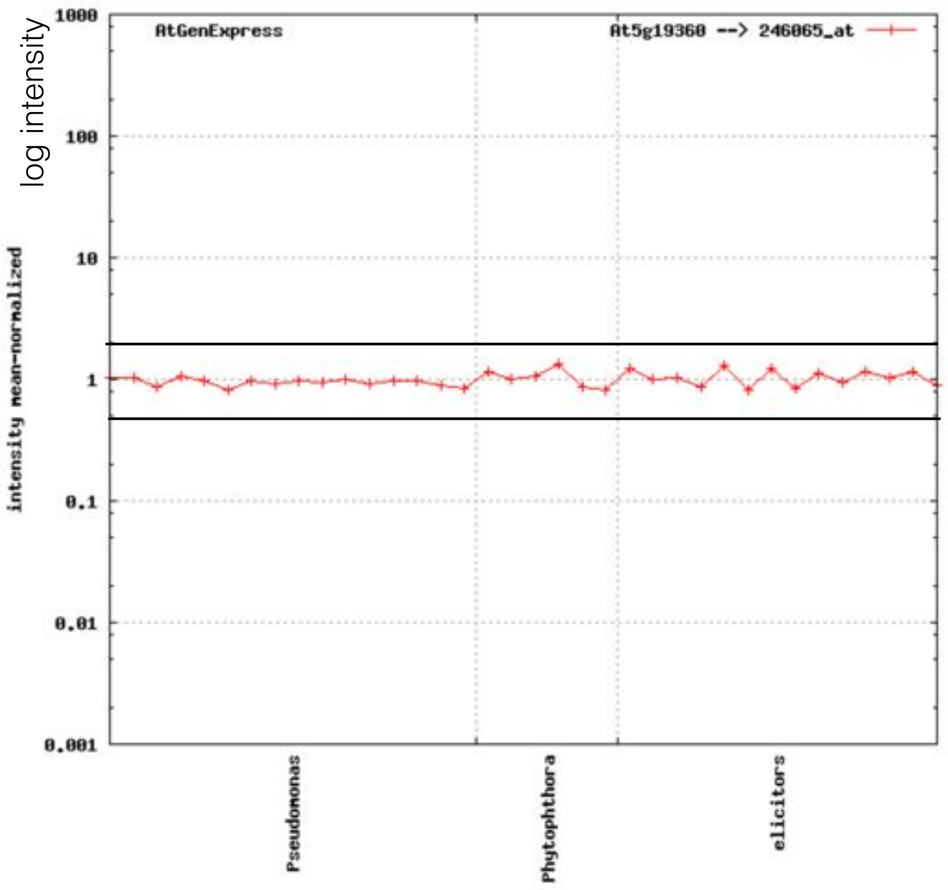
AtGenExpress
At5g19360 --> 246065_at
log intensity
intensity mean-normalized
1000
100
10
1
0.1
0.01
0.001
Pseudomonas
Phytophthora
elicitors

58

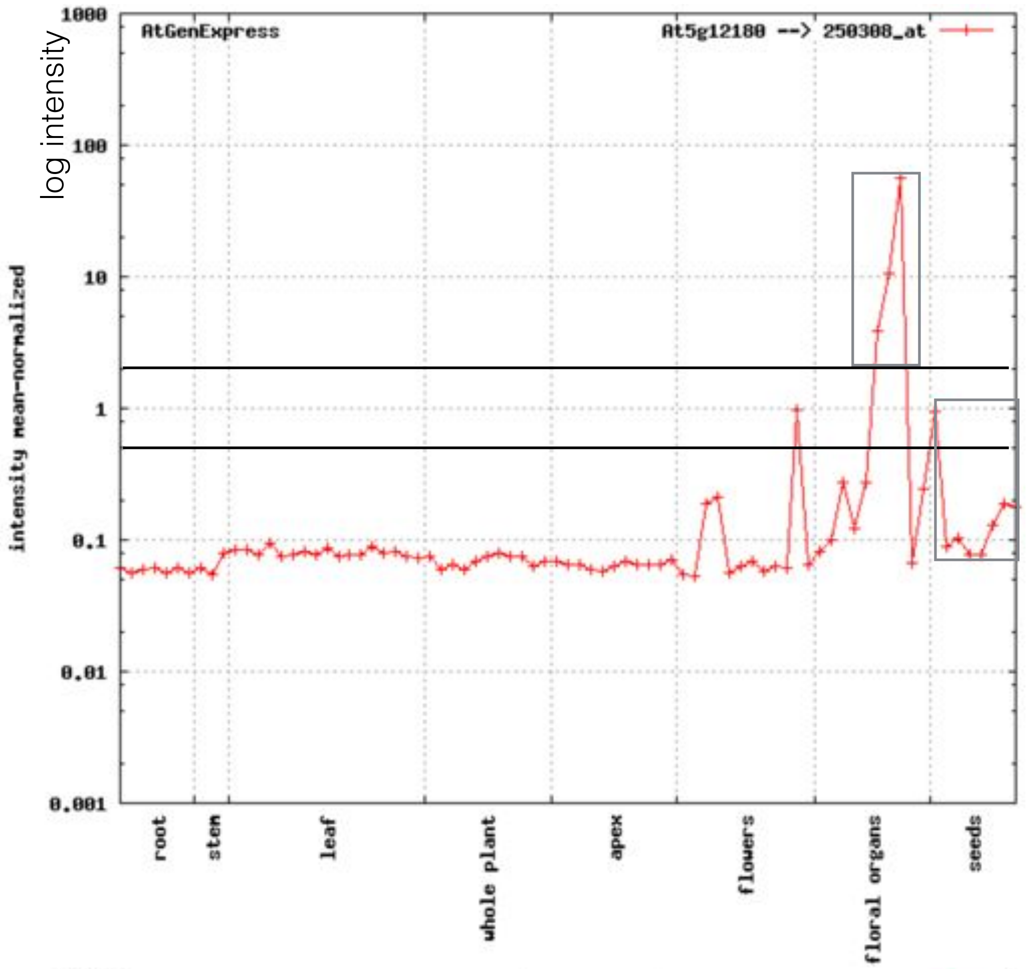

log intensity
intensity mean-normalized
AtGenExpress
At5g12180 --> 250308_at
1000
100
10
1
0.1
0.01
0.001
root
stem
leaf
whole plant
apex
flowers
floral organs
seeds

59

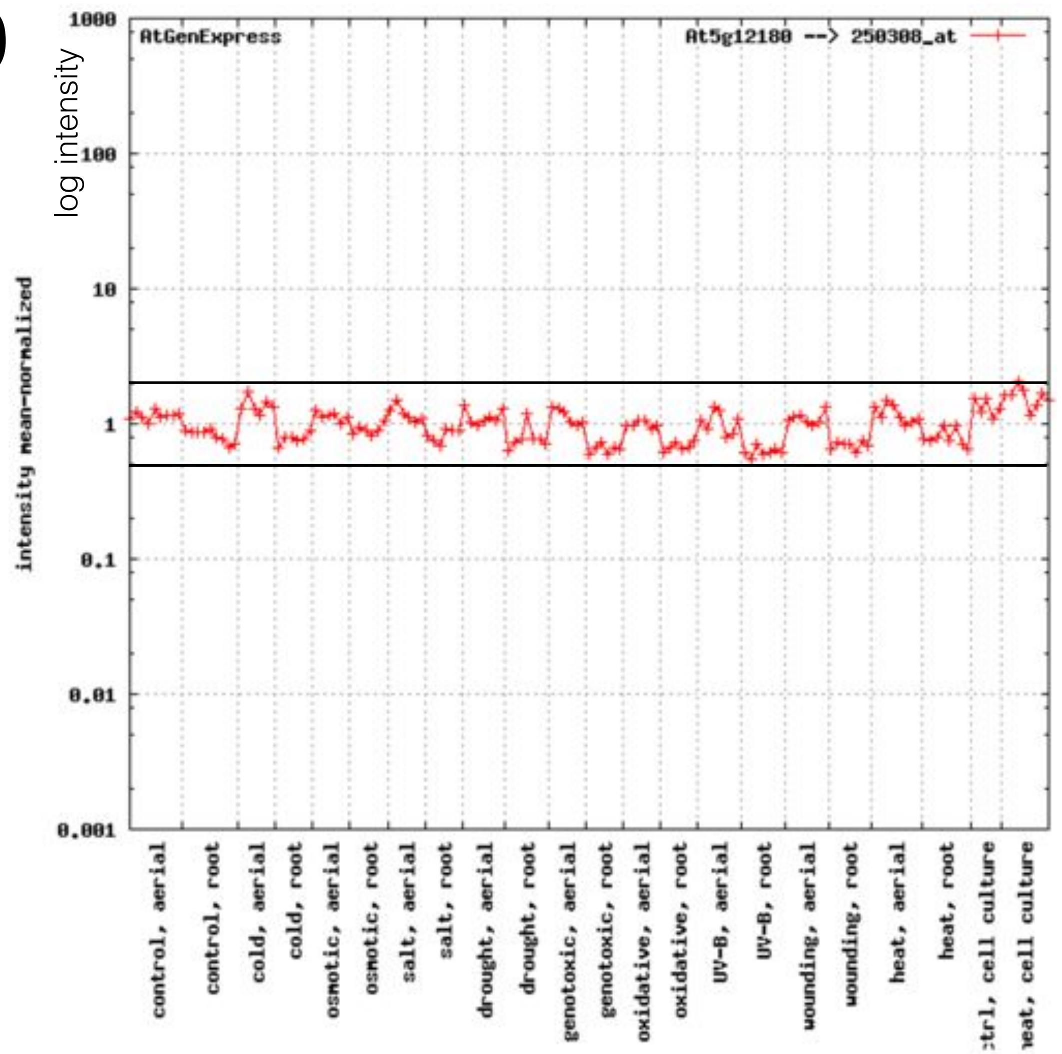

log intensity
intensity mean-normalized
AtGenExpress
At5g12180 --> 250308_at
1000
100
10
1
0.1
0.01
0.001
control, aerial
control, root
cold, aerial
cold, root
osmotic, aerial
osmotic, root
salt, aerial
salt, root
drought, aerial
drought, root
genotoxic, aerial
genotoxic, root
oxidative, aerial
oxidative, root
UV-B, aerial
UV-B, root
wounding, aerial
wounding, root
heat, aerial
heat, root
trl, cell culture
eat, cell culture

60

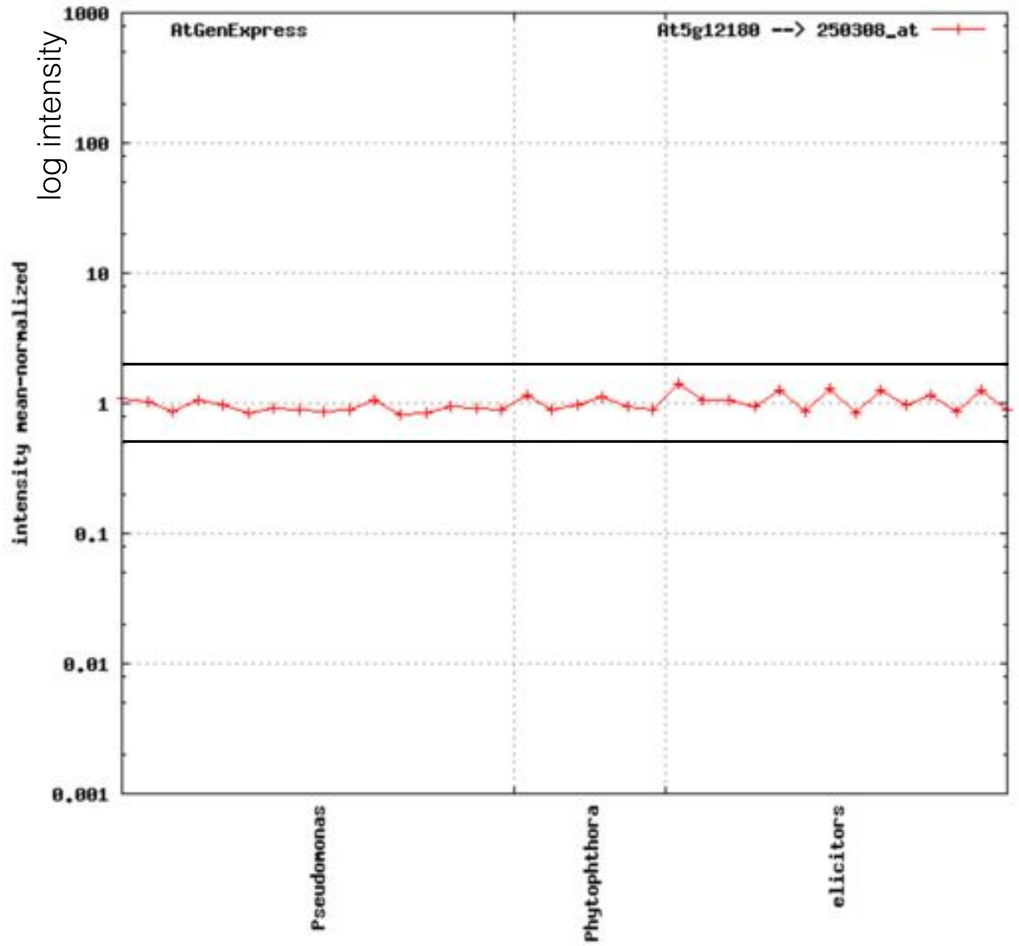

log intensity
intensity mean-normalized
AtGenExpress
At5g12180 --> 250308_at
1000
100
10
1
0.1
0.01
0.001
Pseudomonas
Phytophthora
elicitors

61

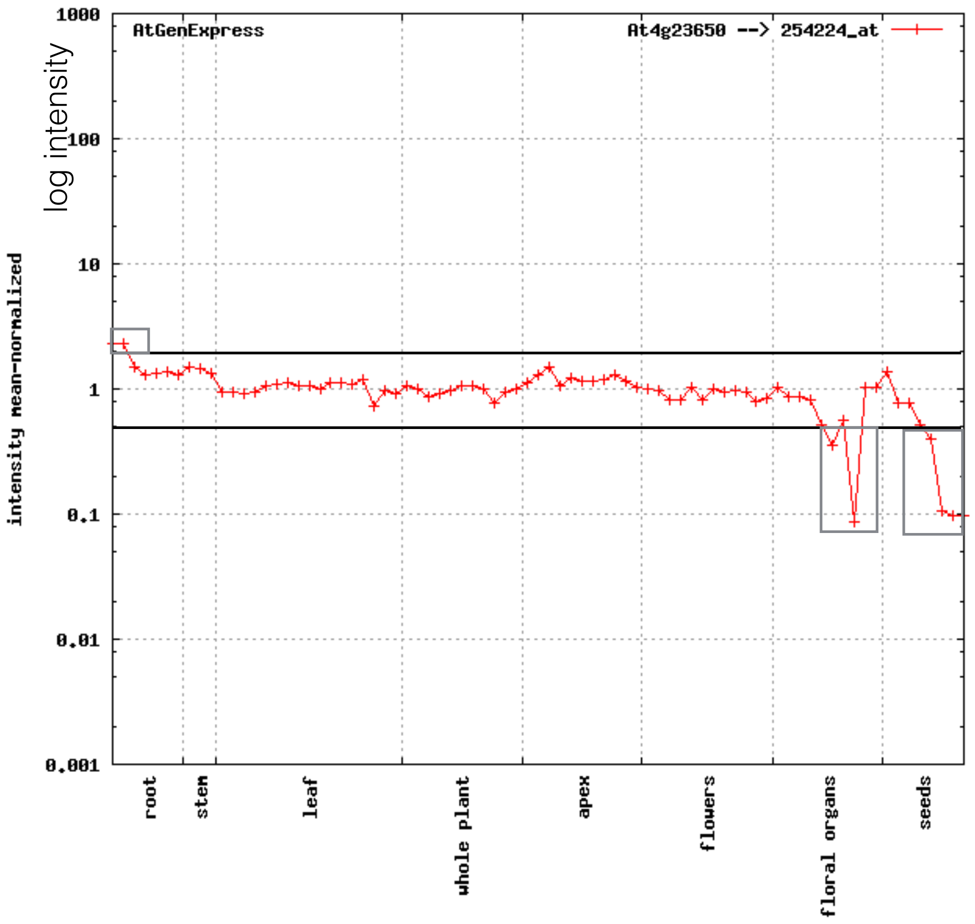
AtGenExpress
At4g23650 --> 254224_at
log intensity
intensity mean-normalized
1000
100
10
1
0.1
0.01
0.001
root
stem
leaf
whole plant
apex
flowers
floral organs
seeds

62

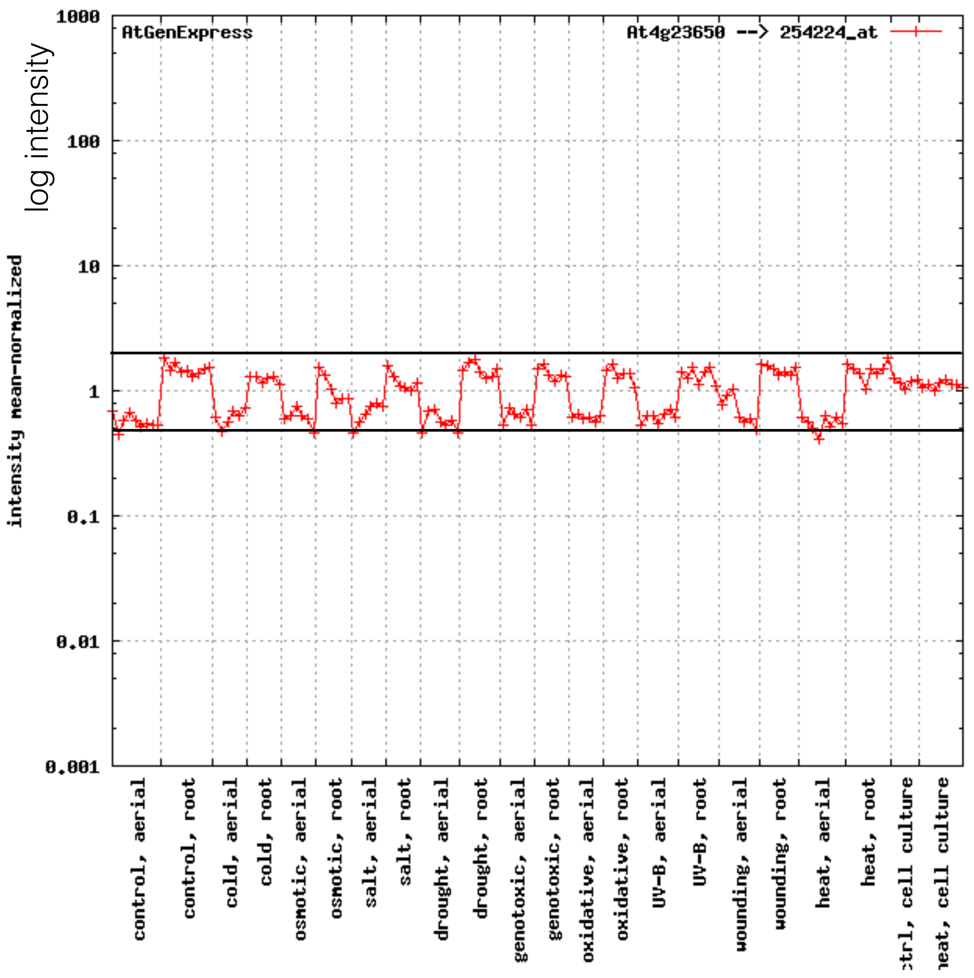
AtGenExpress
At4g23650 --> 254224_at
log intensity
intensity mean-normalized
1000
100
10
1
0.1
0.01
0.001
control, aerial
control, root
cold, aerial
cold, root
osmotic, aerial
osmotic, root
salt, aerial
salt, root
drought, aerial
drought, root
genotoxic, aerial
genotoxic, root
oxidative, aerial
oxidative, root
UV-B, aerial
UV-B, root
wounding, aerial
wounding, root
heat, aerial
heat, root
ctrl, cell culture
heat, cell culture

63

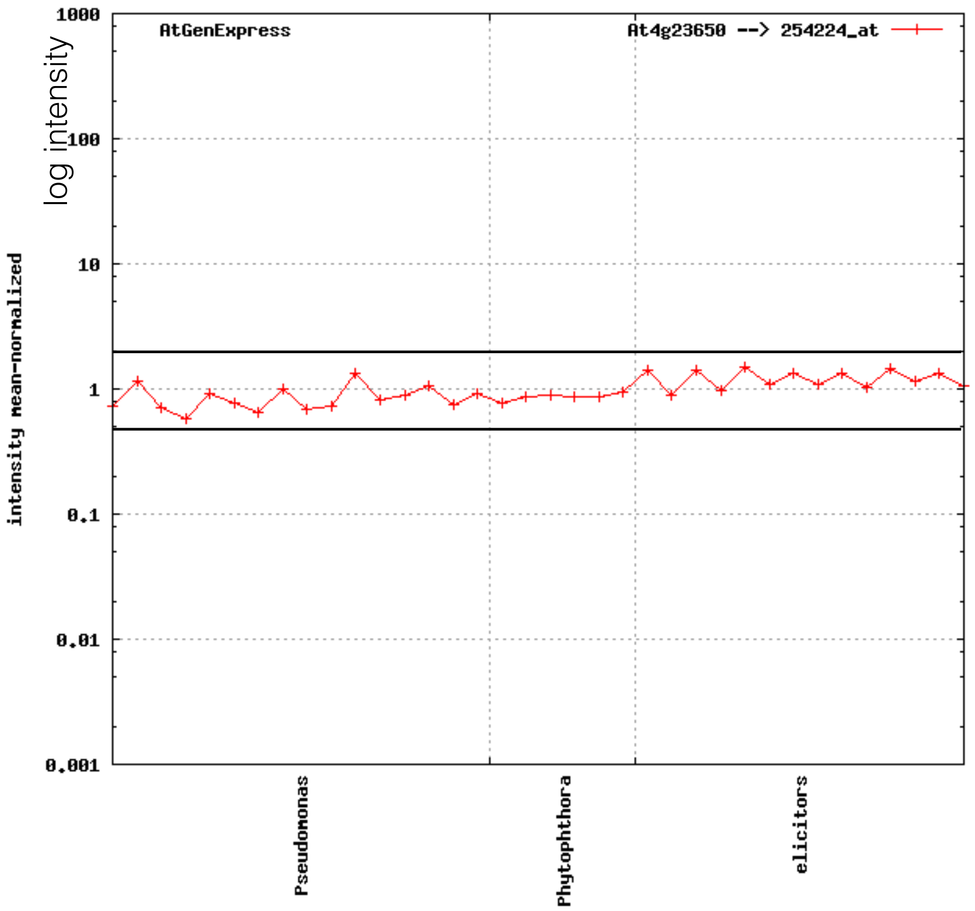
AtGenExpress
At4g23650 --> 254224_at
log intensity
intensity mean-normalized
1000
100
10
1
0.1
0.01
0.001
Pseudomonas
Phytophthora
elicitors

64

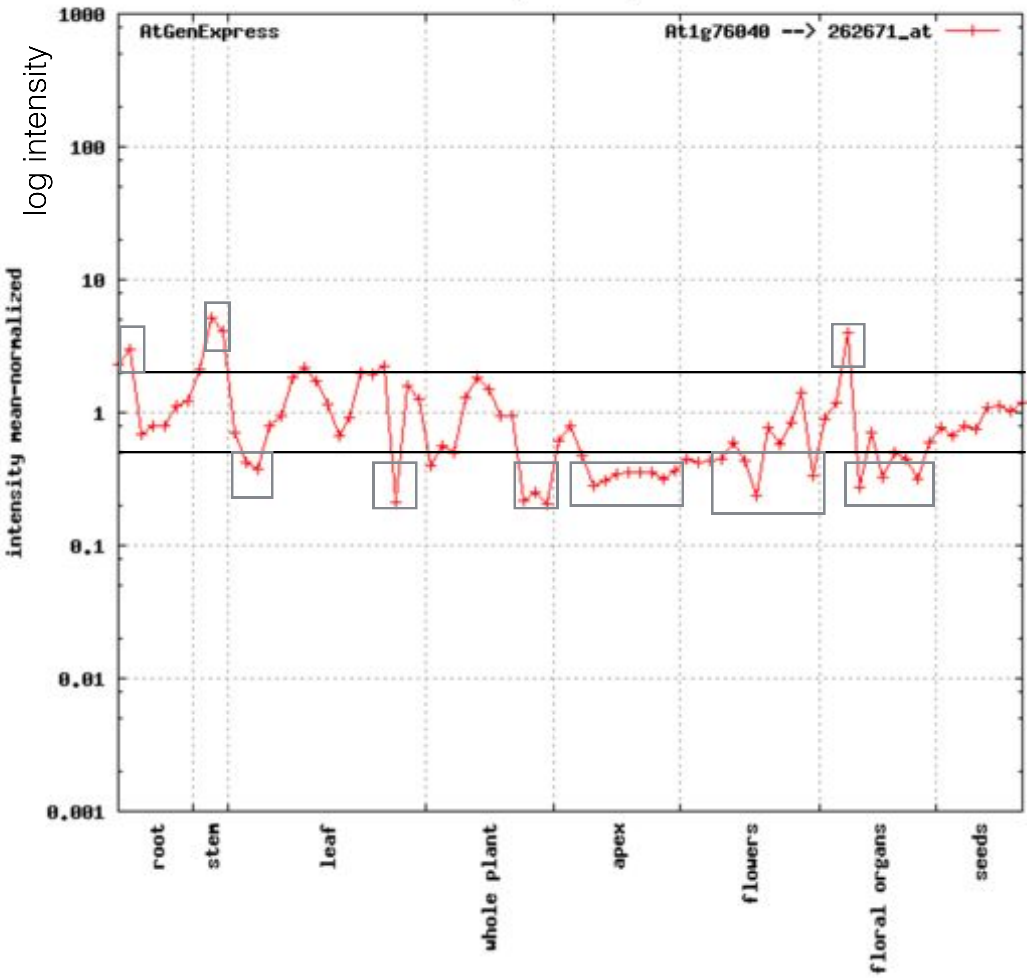
log intensity
AtGenExpress
At1g76040 --> 262671_at
intensity mean-normalized
1000
100
10
1
0.1
0.01
0.001
root
stem
leaf
whole plant
apex
flowers
floral organs
seeds

65

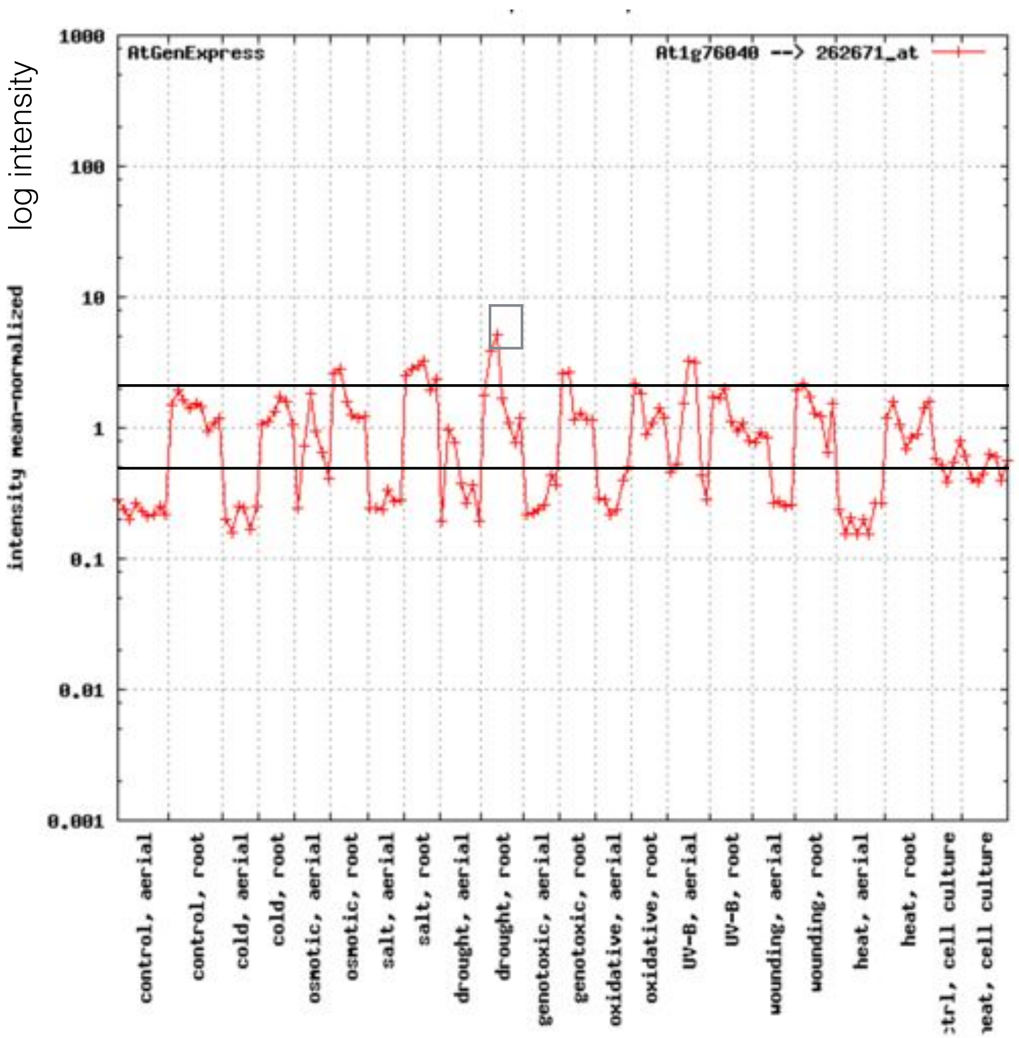
log intensity
AtGenExpress
At1g76040 --> 262671_at
intensity mean-normalized
1000
100
10
1
0.1
0.01
0.001
control, aerial
control, root
cold, aerial
cold, root
osmotic, aerial
osmotic, root
salt, aerial
salt, root
drought, aerial
drought, root
genotoxic, aerial
genotoxic, root
oxidative, aerial
oxidative, root
UV-B, aerial
UV-B, root
wounding, aerial
wounding, root
heat, aerial
heat, root

66

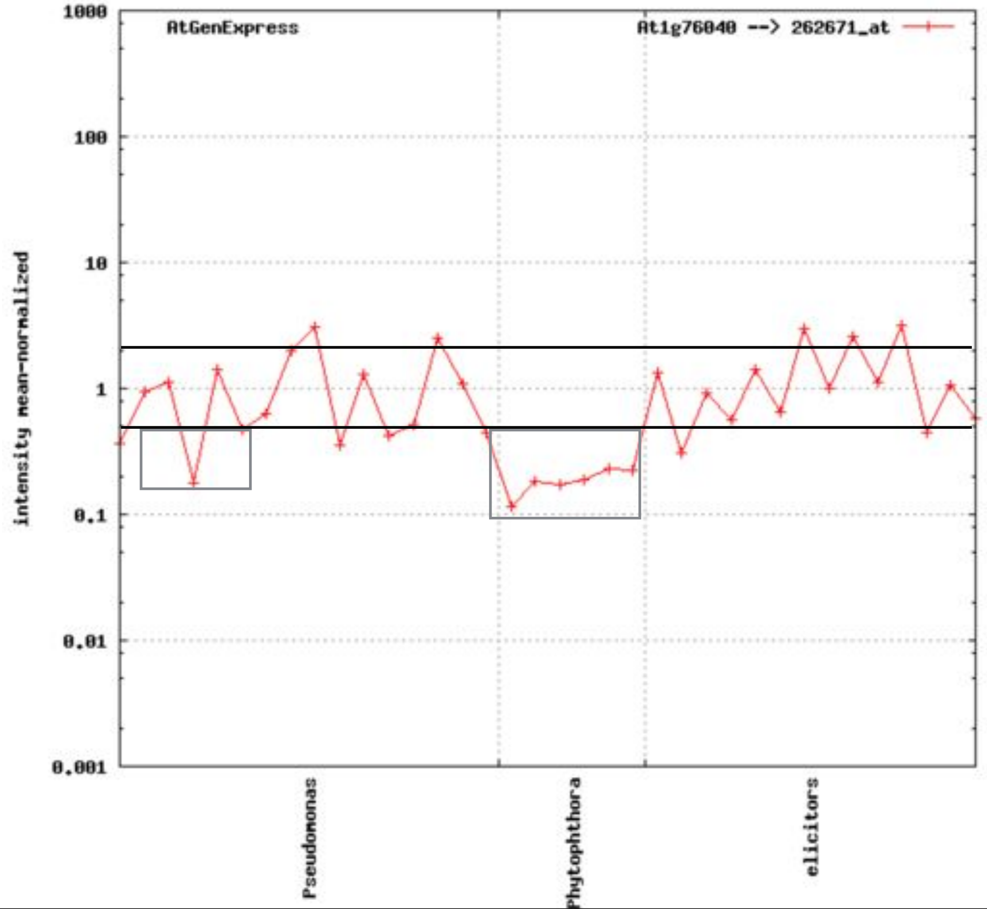
AtGenExpress
At1g76040 --> 262671_at
intensity mean-normalized
1000
100
10
1
0.1
0.01
0.001
Pseudomonas
Phytophthora
elicitors

67

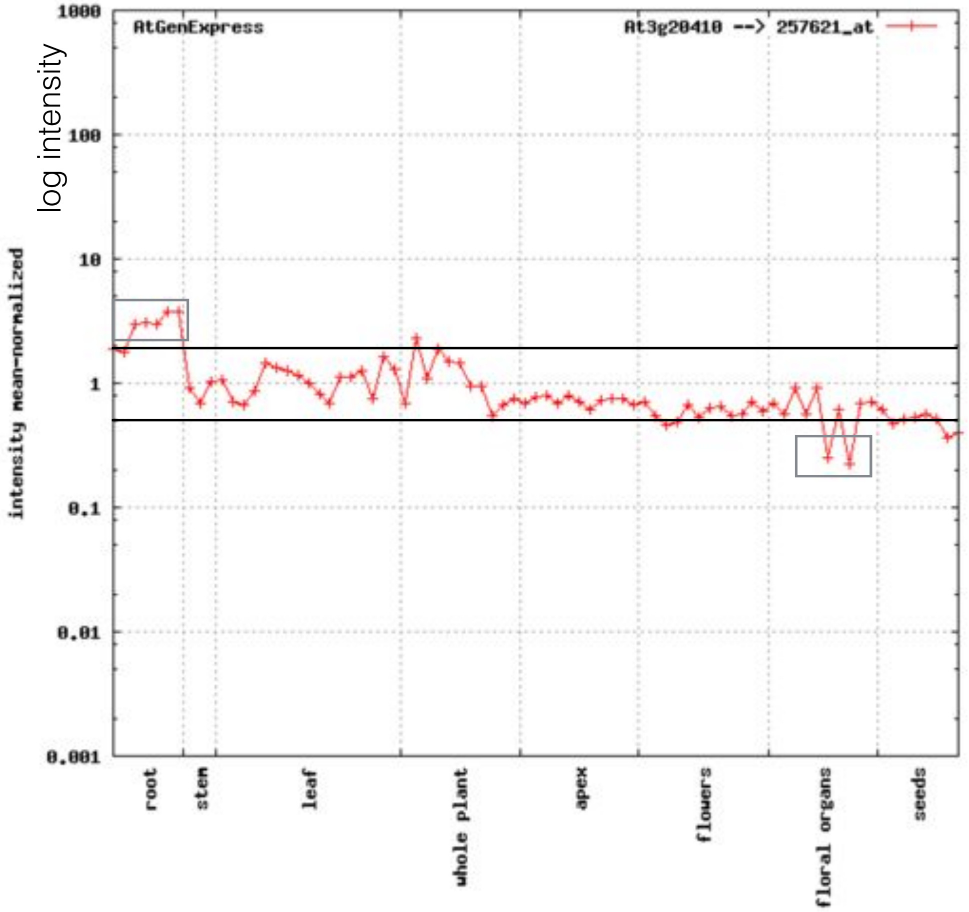
AtGenExpress
At3g20410 --> 257621_at
log intensity
intensity mean-normalized
1000
100
10
1
0.1
0.01
0.001
root
stem
leaf
whole plant
apex
flowers
floral organs
seeds

68

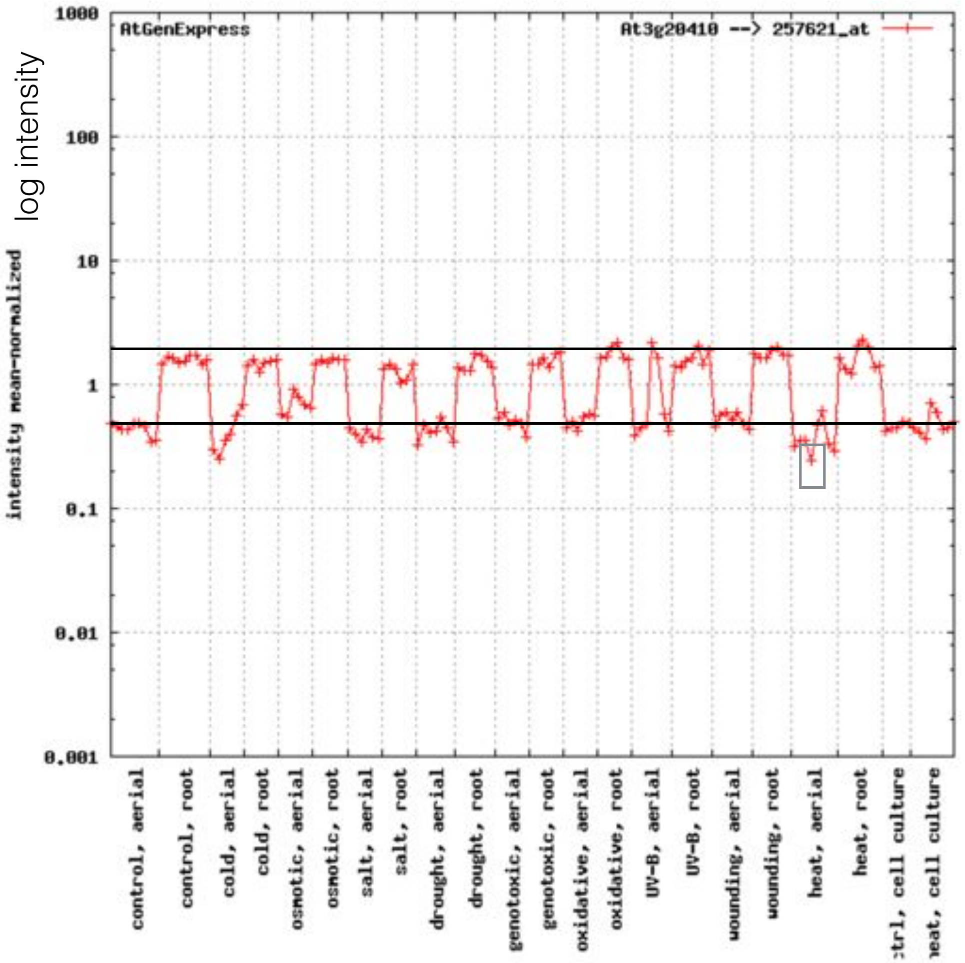
AtGenExpress
At3g20410 --> 257621_at
log intensity
intensity mean-normalized
1000
100
10
1
0.1
0.01
0.001
control, aerial
control, root
cold, aerial
cold, root
osmotic, aerial
osmotic, root
salt, aerial
salt, root
drought, aerial
drought, root
genotoxic, aerial
genotoxic, root
oxidative, aerial
oxidative, root
UV-B, aerial
UV-B, root
wounding, aerial
wounding, root
heat, aerial
heat, root

69

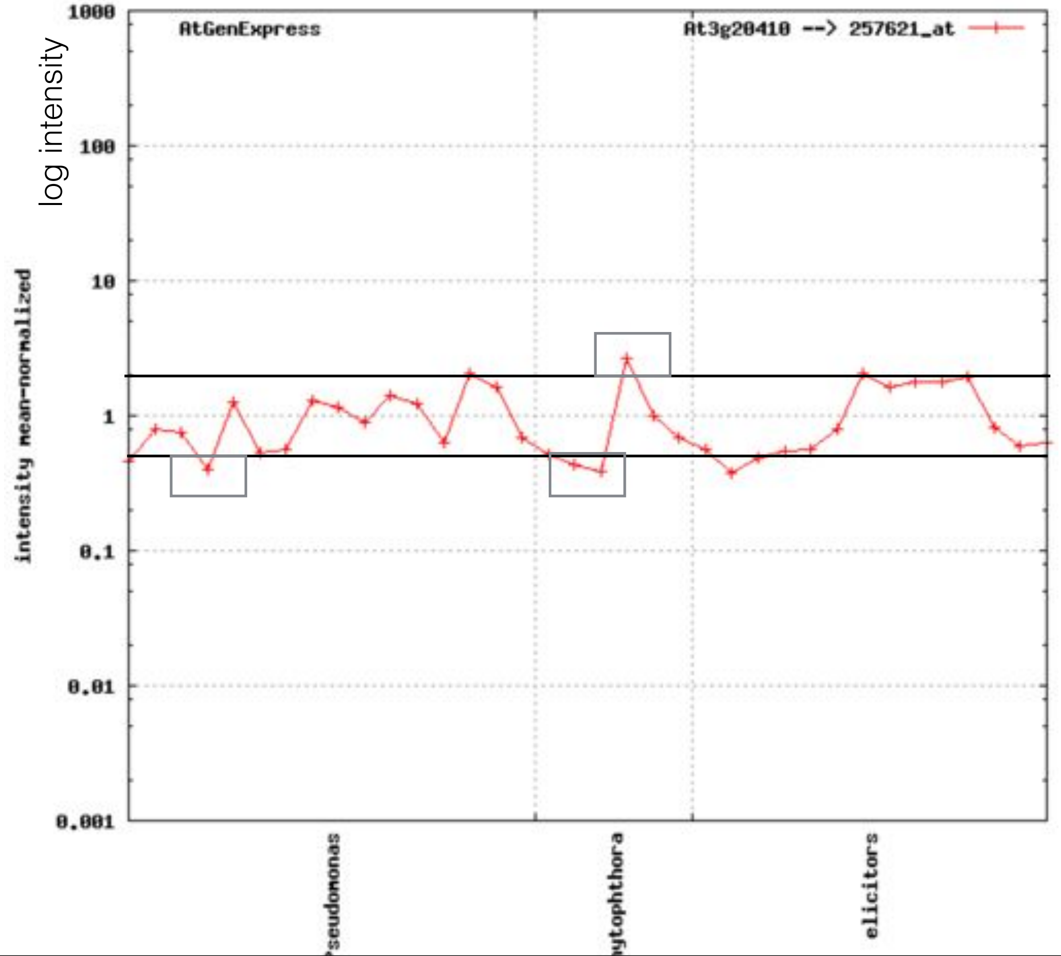
AtGenExpress
At3g20410 --> 257621_at
log intensity
intensity mean-normalized
1000
100
10
1
0.1
0.01
0.001
elicitors

70

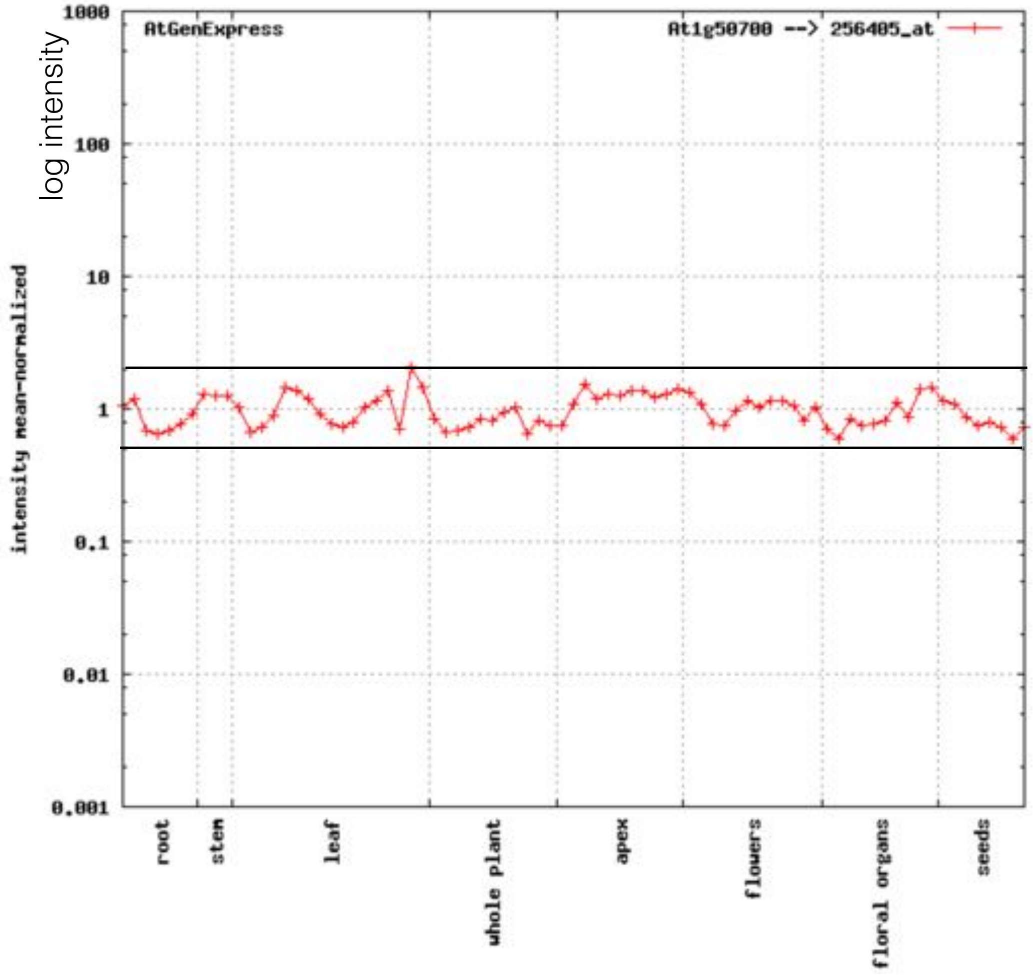
AtGenExpress
At1g50700 --> 256405_at
log intensity
intensity mean-normalized
1000
100
10
1
0.1
0.01
0.001
root
stem
leaf
whole plant
apex
flowers
floral organs
seeds

71

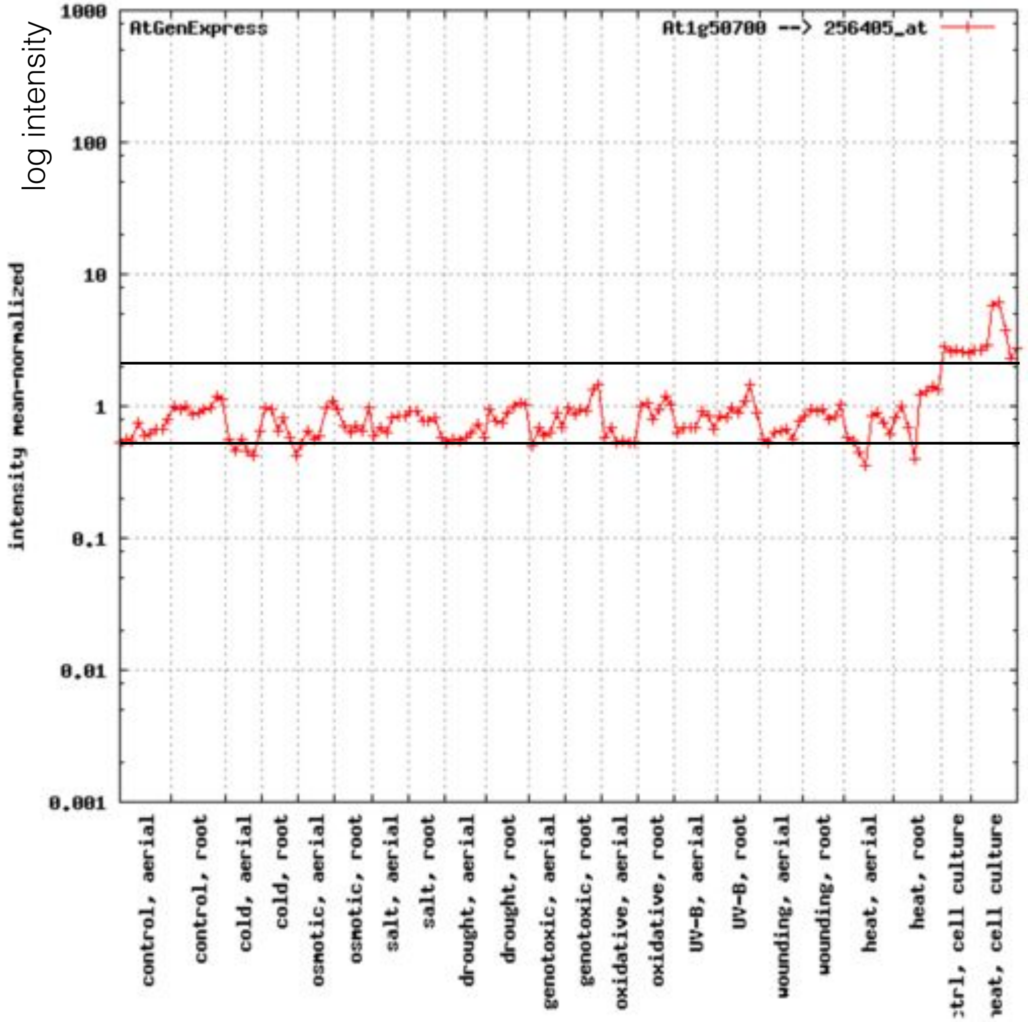
AtGenExpress
At1g50700 --> 256405_at
log intensity
intensity mean-normalized
1000
100
10
1
0.1
0.01
0.001
control, aerial
control, root
cold, aerial
cold, root
osmotic, aerial
osmotic, root
salt, aerial
salt, root
drought, aerial
drought, root
genotoxic, aerial
genotoxic, root
oxidative, aerial
oxidative, root
UV-B, aerial
UV-B, root
wounding, aerial
wounding, root
heat, aerial
heat, root
cell culture
cell culture

72

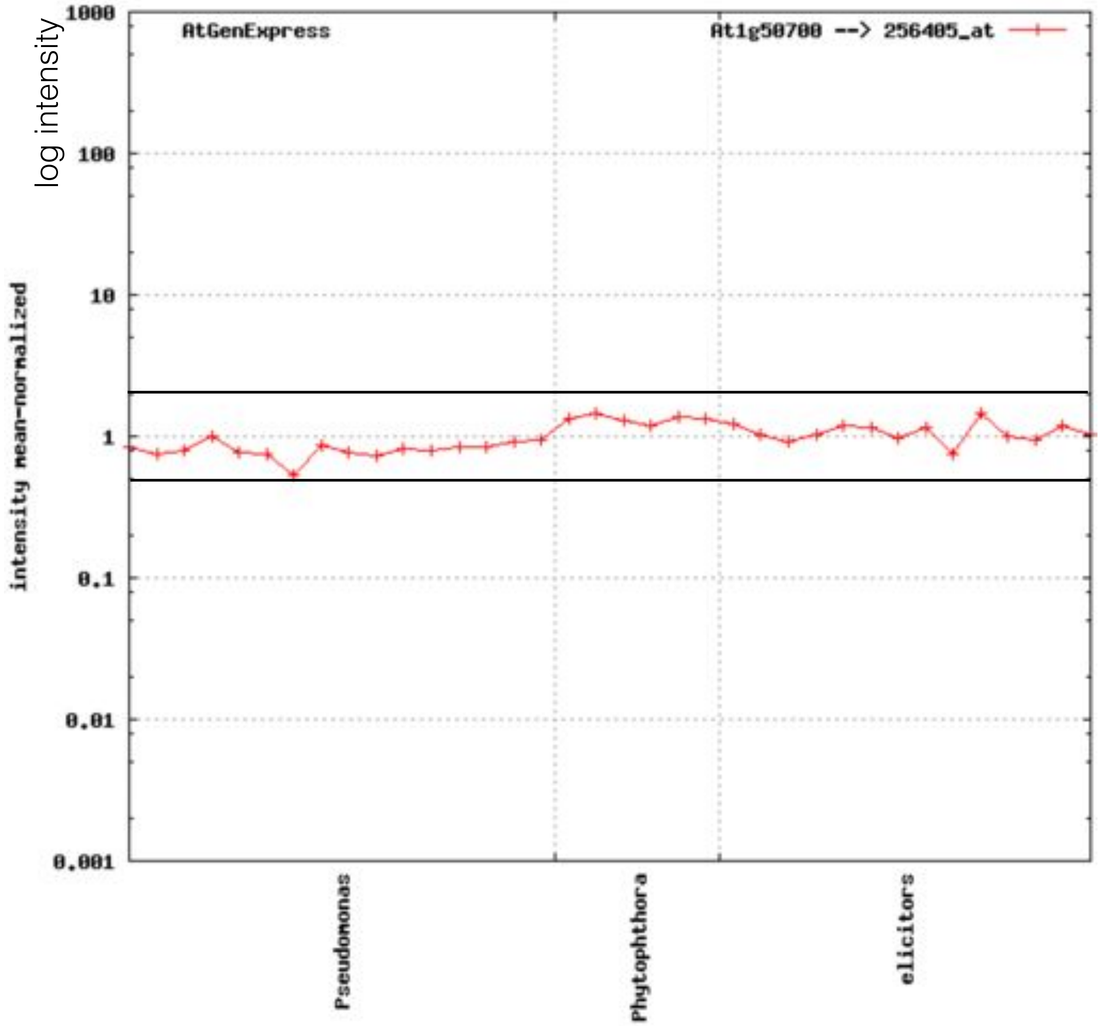
AtGenExpress
At1g50700 --> 256405_at
log intensity
intensity mean-normalized
1000
100
10
1
0.1
0.01
0.001
Pseudomonas
Phytophthora
elicitors

73

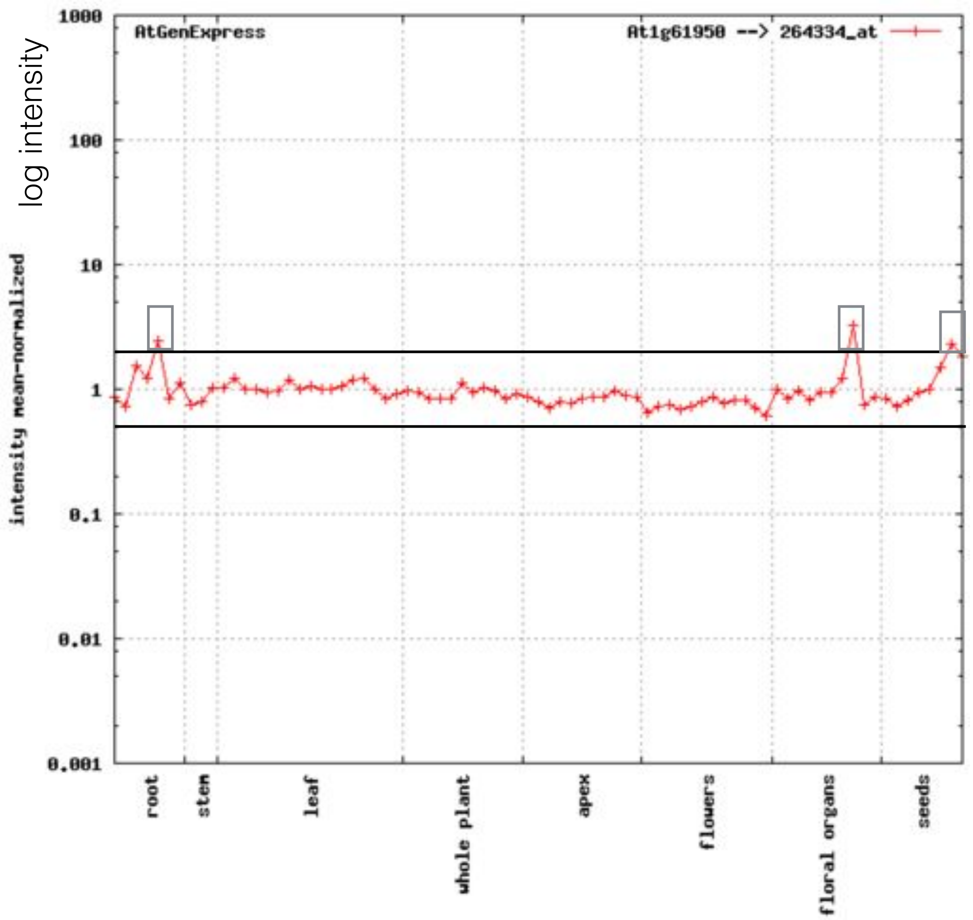
log intensity
intensity mean-normalized
AtGenExpress
At1g61950 --> 264334_at
1000
100
10
1
0.1
0.01
0.001
root
stem
leaf
whole plant
apex
flowers
floral organs
seeds

74

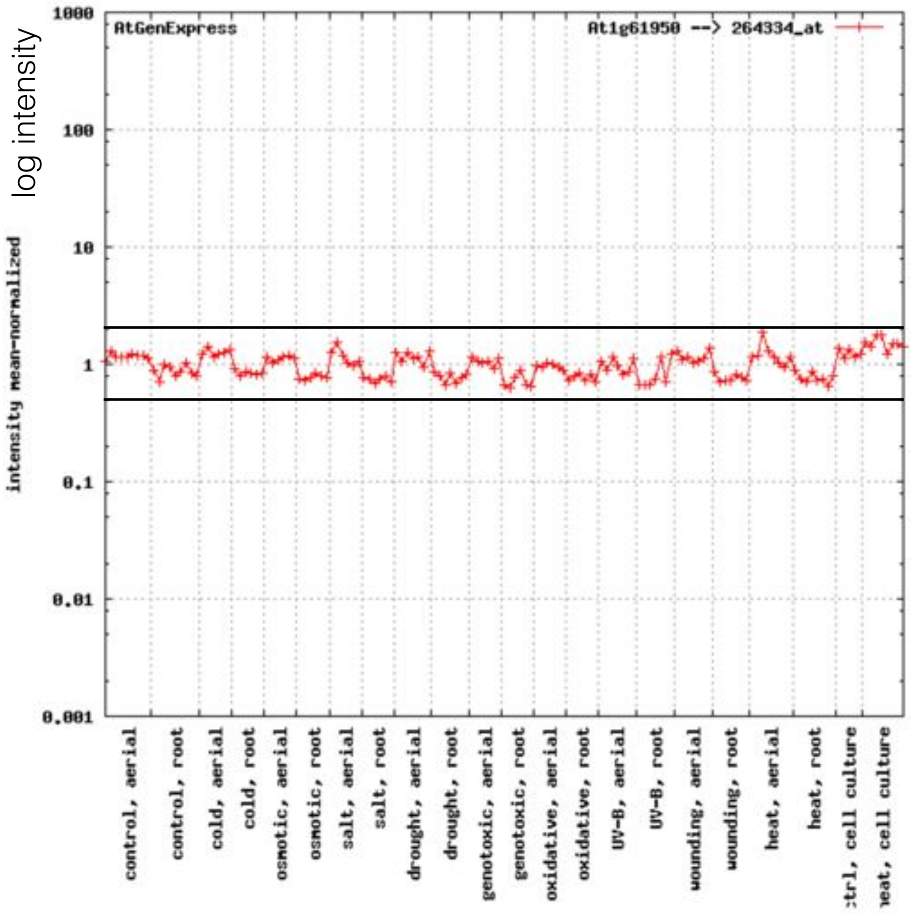
log intensity
intensity mean-normalized
AtGenExpress
At1g61950 --> 264334_at
1000
100
10
1
0.1
0.01
0.001
control, aerial
control, root
cold, aerial
cold, root
osmotic, aerial
osmotic, root
salt, aerial
salt, root
drought, aerial
drought, root
genotoxic, aerial
genotoxic, root
oxidative, aerial
oxidative, root
UV-B, aerial
UV-B, root
wounding, aerial
wounding, root
heat, aerial
heat, root

75

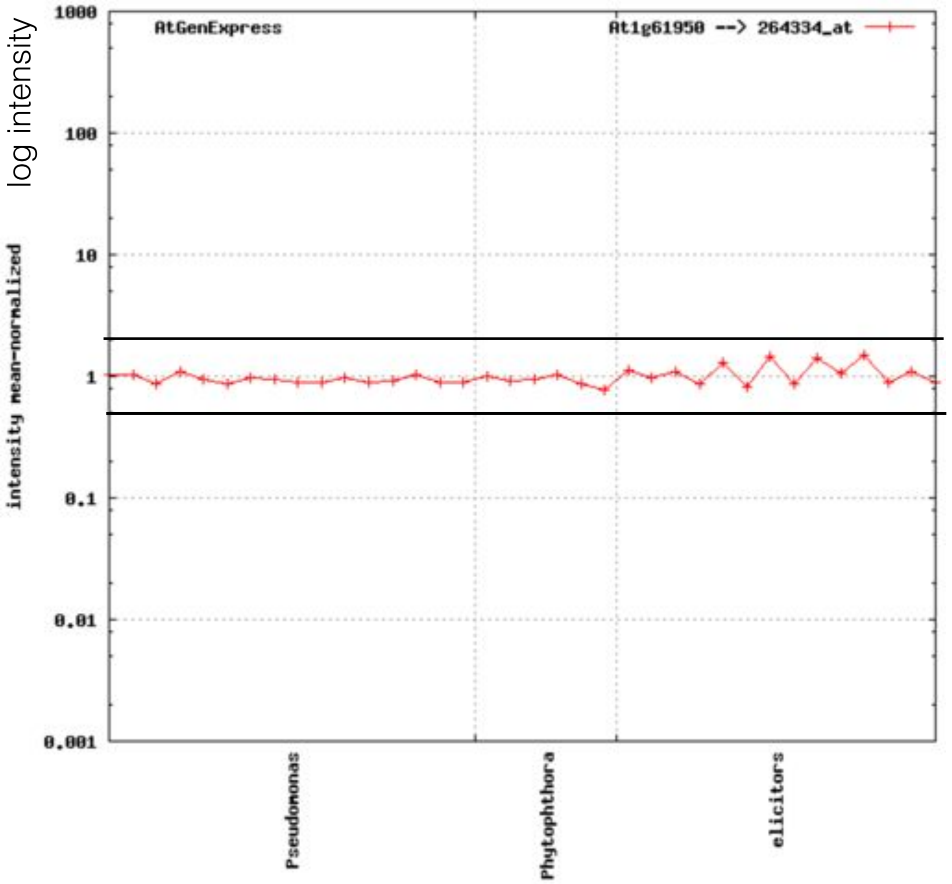
log intensity
intensity mean-normalized
AtGenExpress
At1g61950 --> 264334_at
1000
100
10
1
0.1
0.01
0.001
Pseudomonas
Phytophthora
elicitors

76

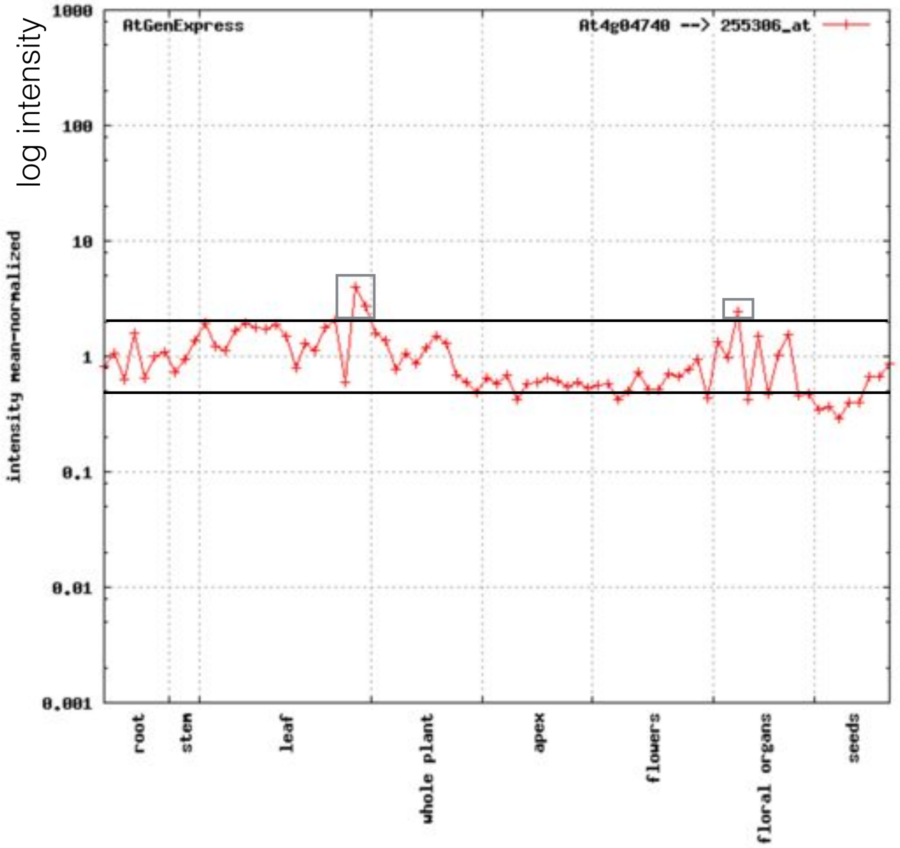
log intensity
intensity mean-normalized
1000
100
10
1
0.1
0.01
0.001
AtGenExpress
At4g04740 --> 255306_at
root
stem
leaf
whole plant
apex
flowers
floral organs
seeds

77

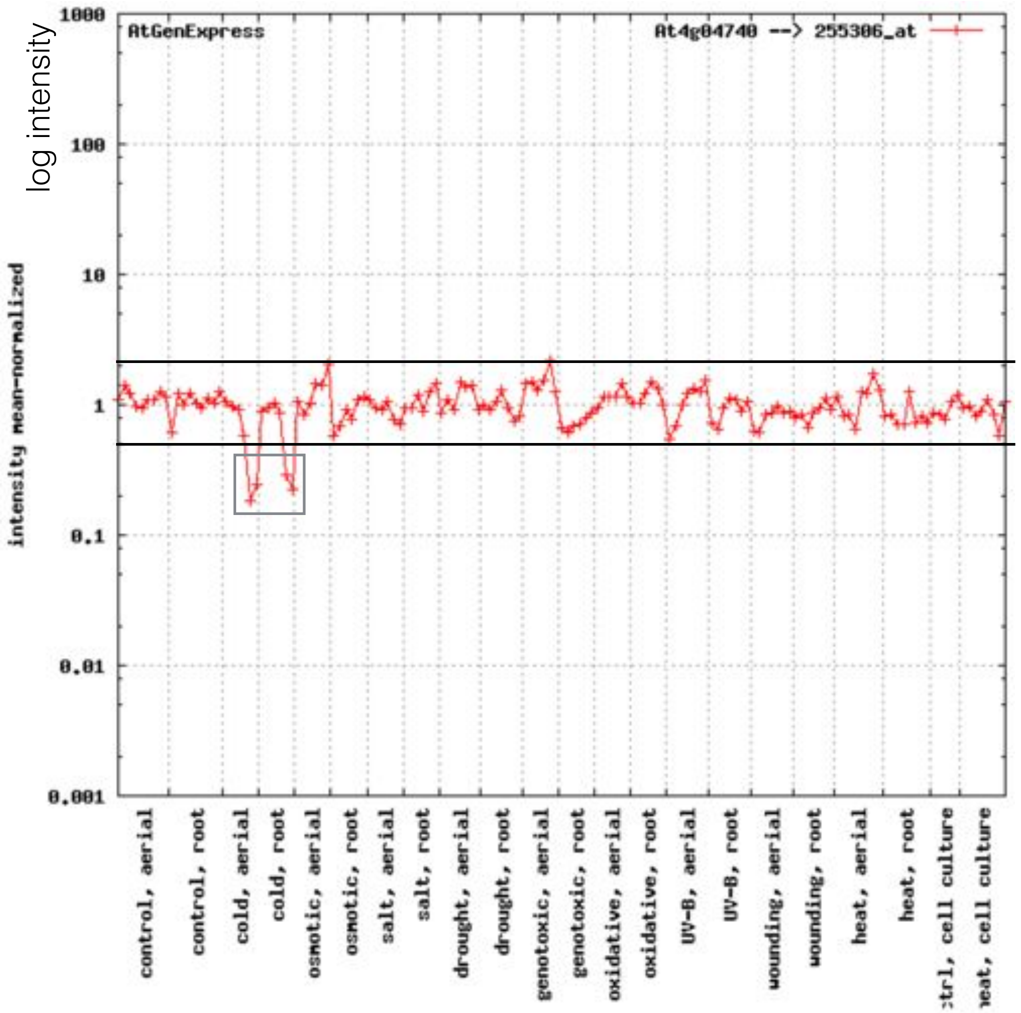
log intensity
intensity mean-normalized
1000
100
10
1
0.1
0.01
0.001
AtGenExpress
At4g04740 --> 255306_at
control, aerial
control, root
cold, aerial
cold, root
osmotic, aerial
osmotic, root
salt, aerial
salt, root
drought, aerial
drought, root
genotoxic, aerial
genotoxic, root
oxidative, aerial
oxidative, root
UV-B, aerial
UV-B, root
wounding, aerial
wounding, root
heat, aerial
heat, root

78

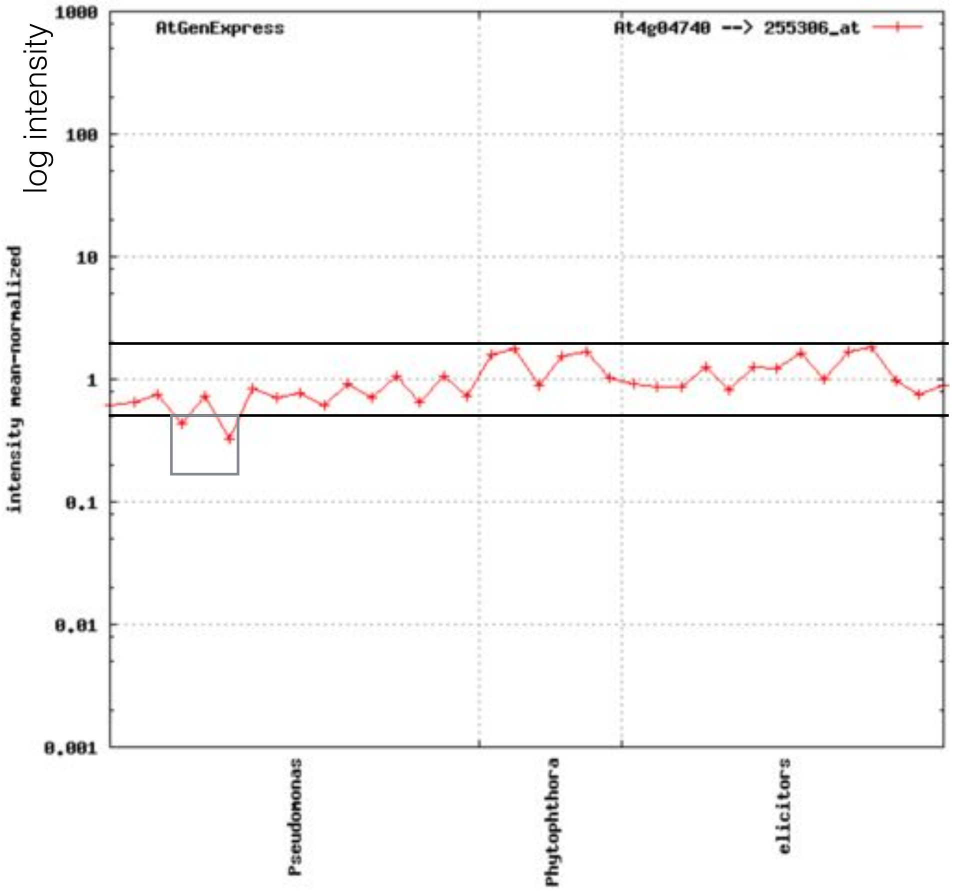
log intensity
intensity mean-normalized
1000
100
10
1
0.1
0.01
0.001
AtGenExpress
At4g04740 --> 255306_at
Pseudomonas
Phytophthora
elicitors

79

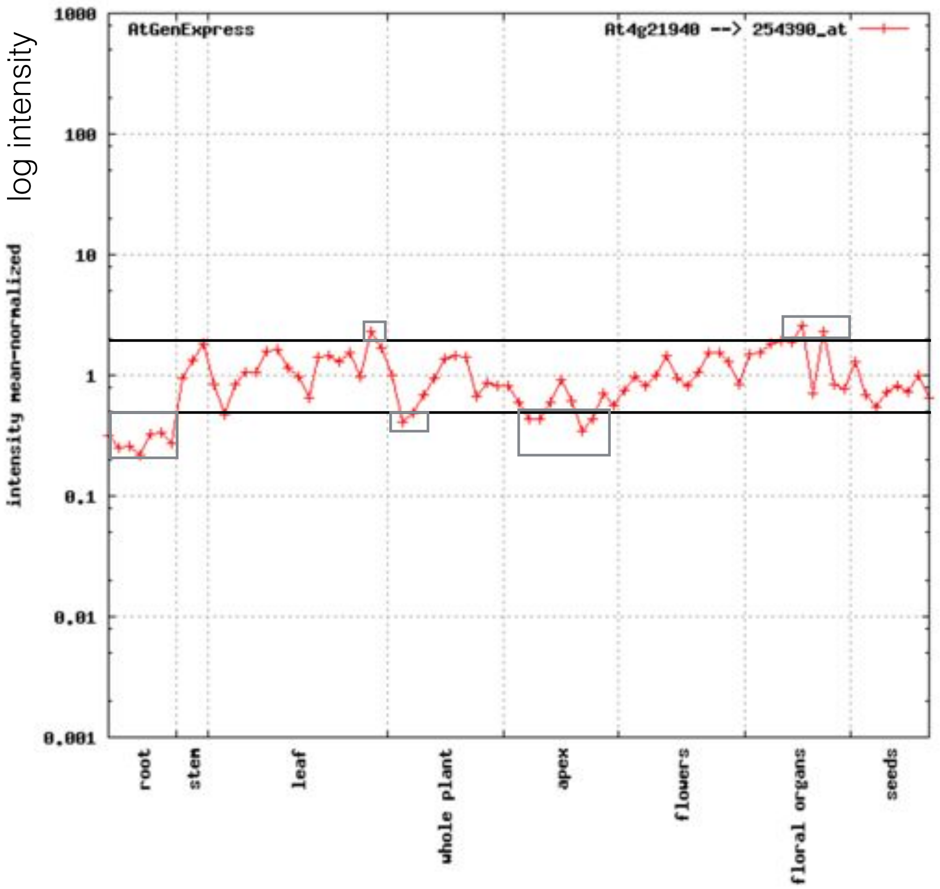

log intensity
intensity mean-normalized
AtGenExpress
At4g21940 --> 254390_at
1000
100
10
1
0.1
0.01
0.001
root
stem
leaf
whole plant
apex
flowers
floral organs
seeds

80

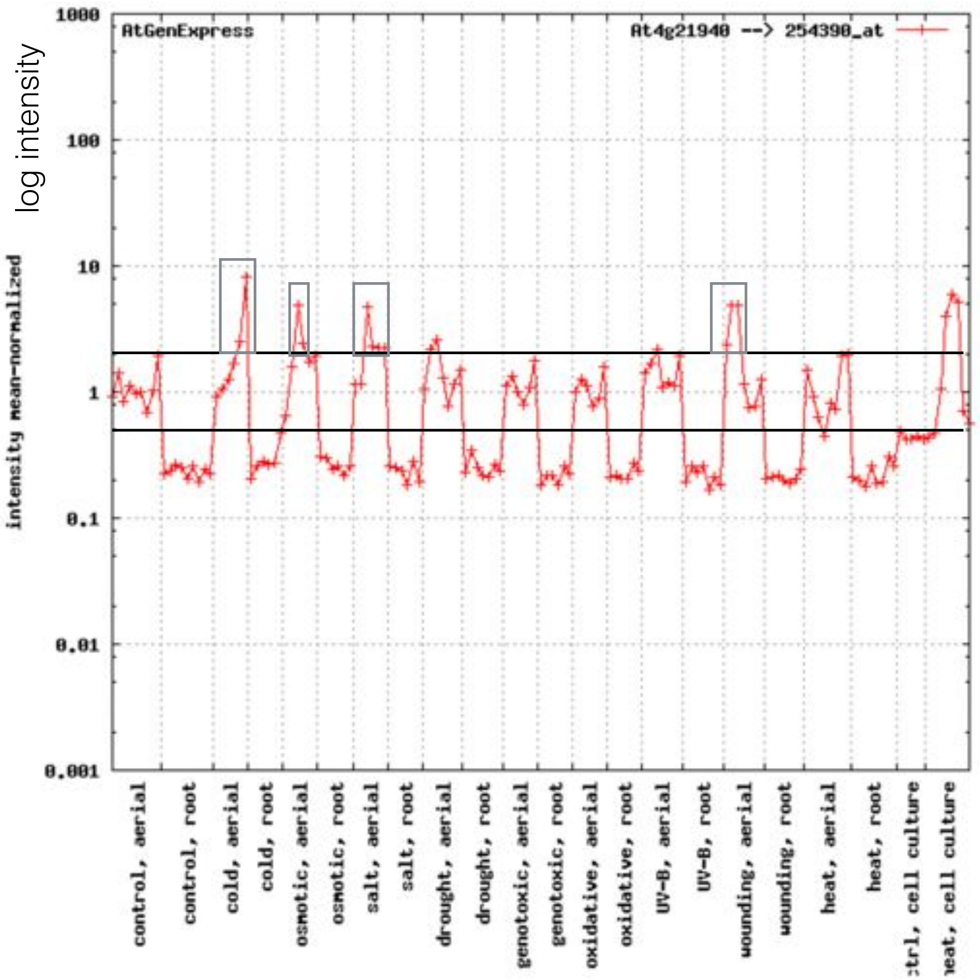

log intensity
intensity mean-normalized
AtGenExpress
At4g21940 --> 254390_at
1000
100
10
1
0.1
0.01
0.001
control, aerial
control, root
cold, aerial
cold, root
osmotic, aerial
osmotic, root
salt, aerial
salt, root
drought, aerial
drought, root
genotoxic, aerial
genotoxic, root
oxidative, aerial
oxidative, root
UV-B, aerial
UV-B, root
wounding, aerial
wounding, root
heat, aerial
heat, root

81

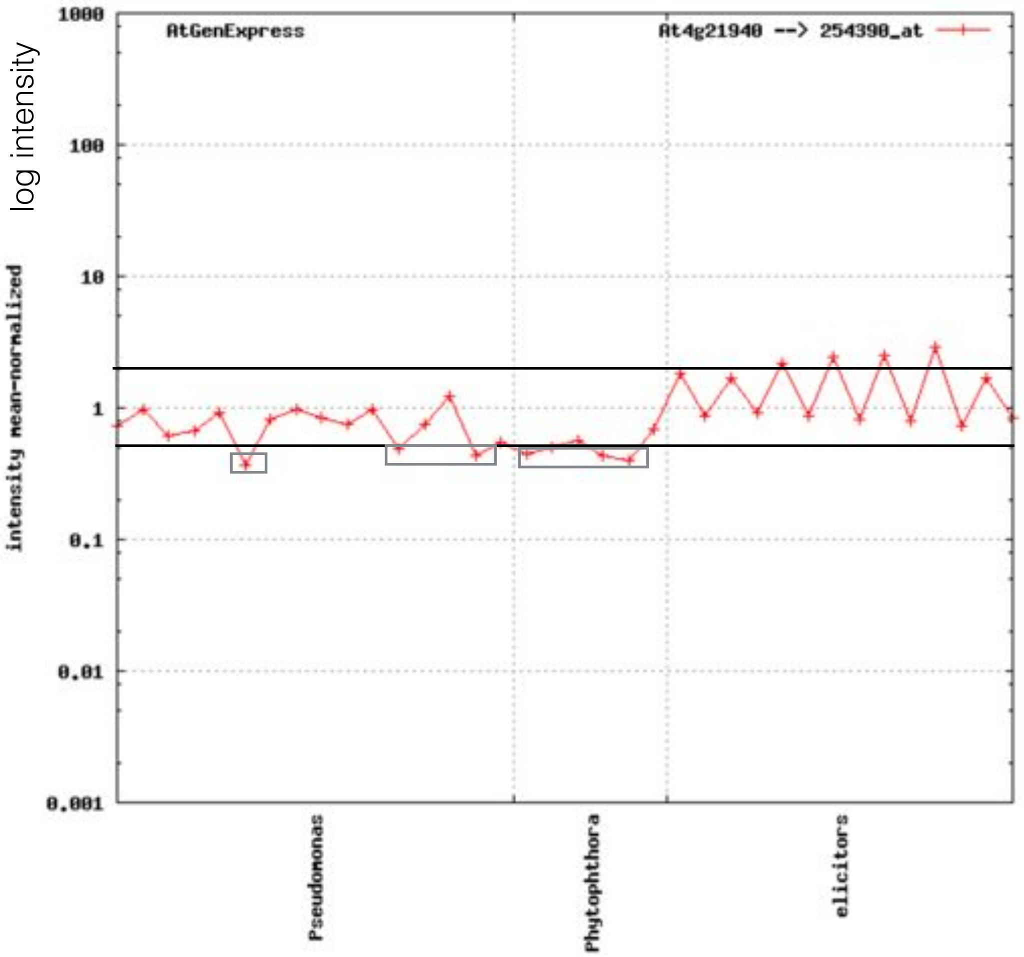

log intensity
intensity mean-normalized
AtGenExpress
At4g21940 --> 254390_at
1000
100
10
1
0.1
0.01
0.001
Pseudomonas
Phytophthora
elicitors

82

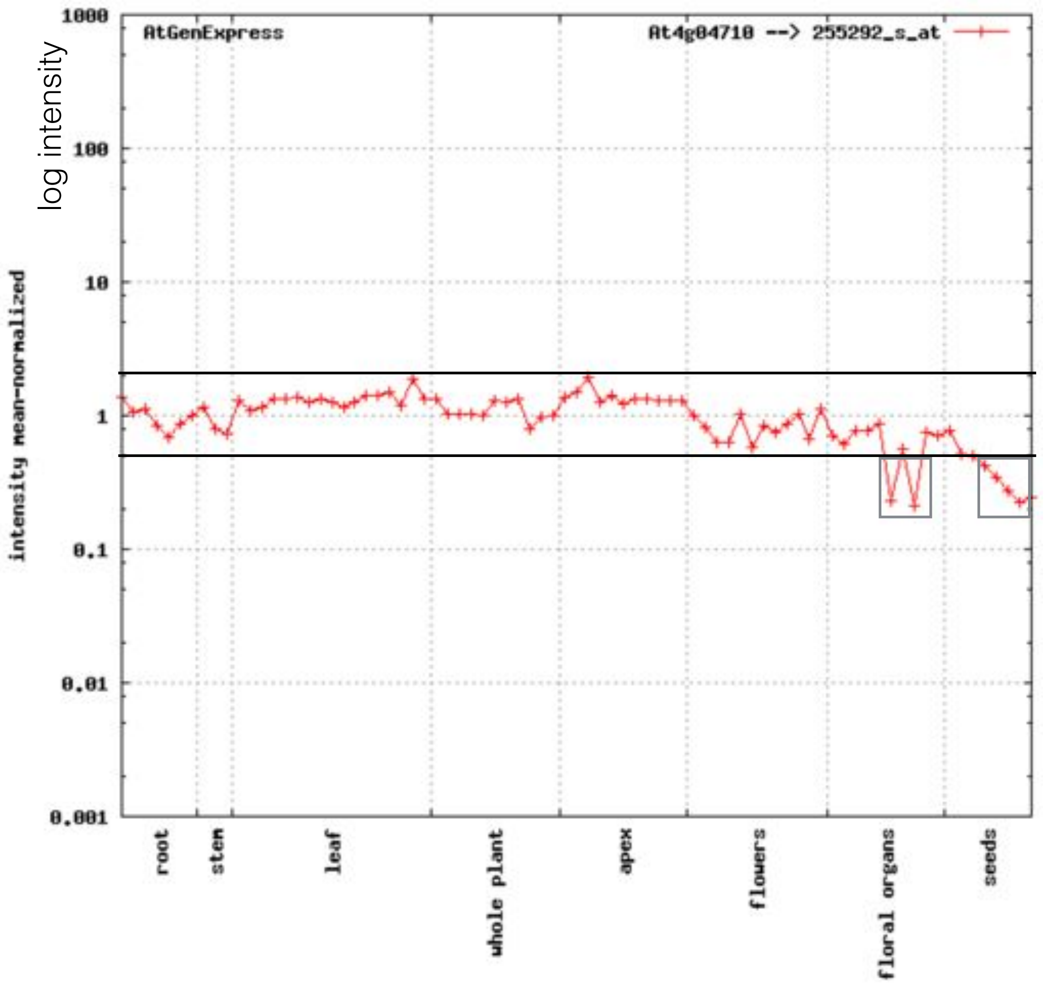
AtGenExpress
At4g04710 --> 255292_s_at
log intensity
intensity mean-normalized
1000
100
10
1
0,1
0,01
0,001
root
stem
leaf
whole plant
apex
flowers
floral organs
seeds

83

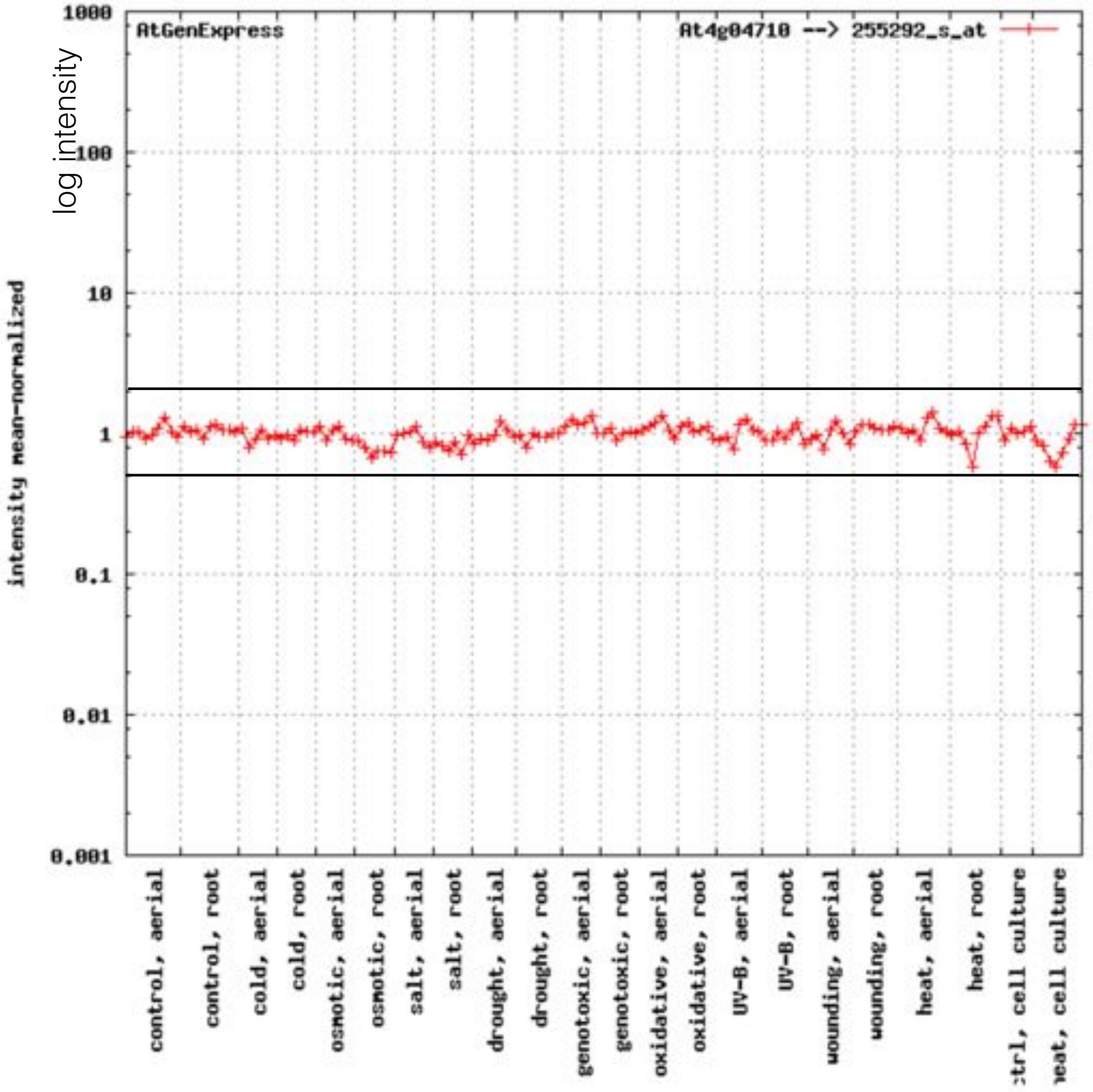
AtGenExpress
At4g04710 --> 255292_s_at
log intensity
intensity mean-normalized
1000
100
10
1
0,1
0,01
0,001
control, aerial
control, root
cold, aerial
cold, root
osmotic, aerial
osmotic, root
salt, aerial
salt, root
drought, aerial
drought, root
genotoxic, aerial
genotoxic, root
oxidative, aerial
oxidative, root
UV-B, aerial
UV-B, root
wounding, aerial
wounding, root
heat, aerial
heat, root

84

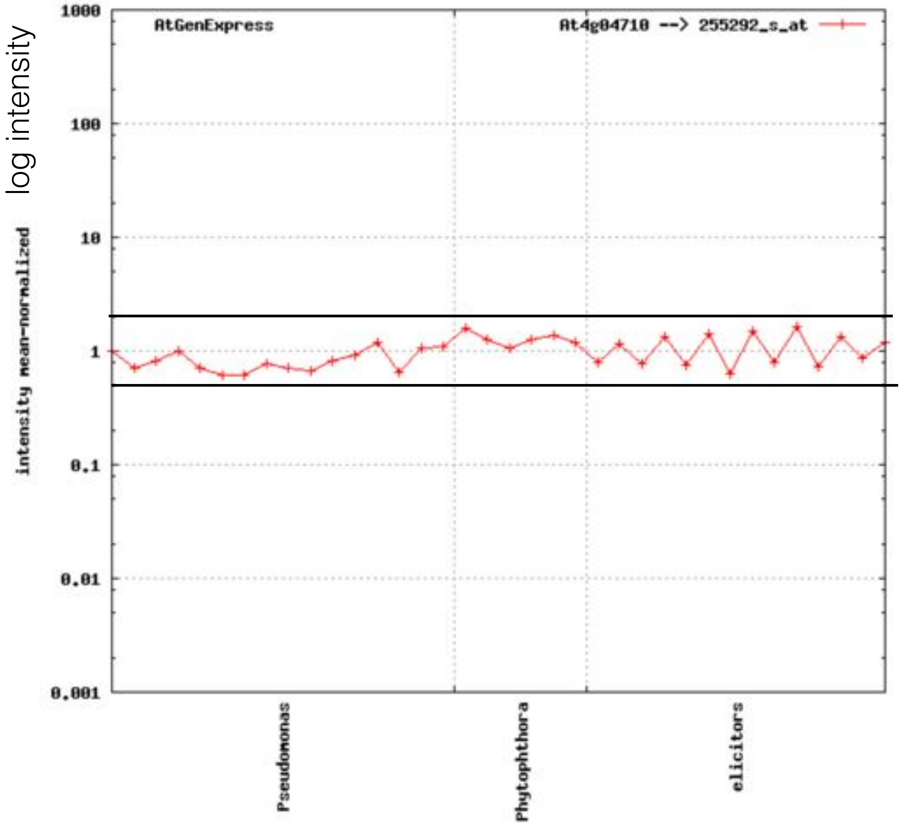
AtGenExpress
At4g04710 --> 255292_s_at
log intensity
intensity mean-normalized
1000
100
10
1
0,1
0,01
0,001
Pseudomonas
Phytophthora
elicitors

85

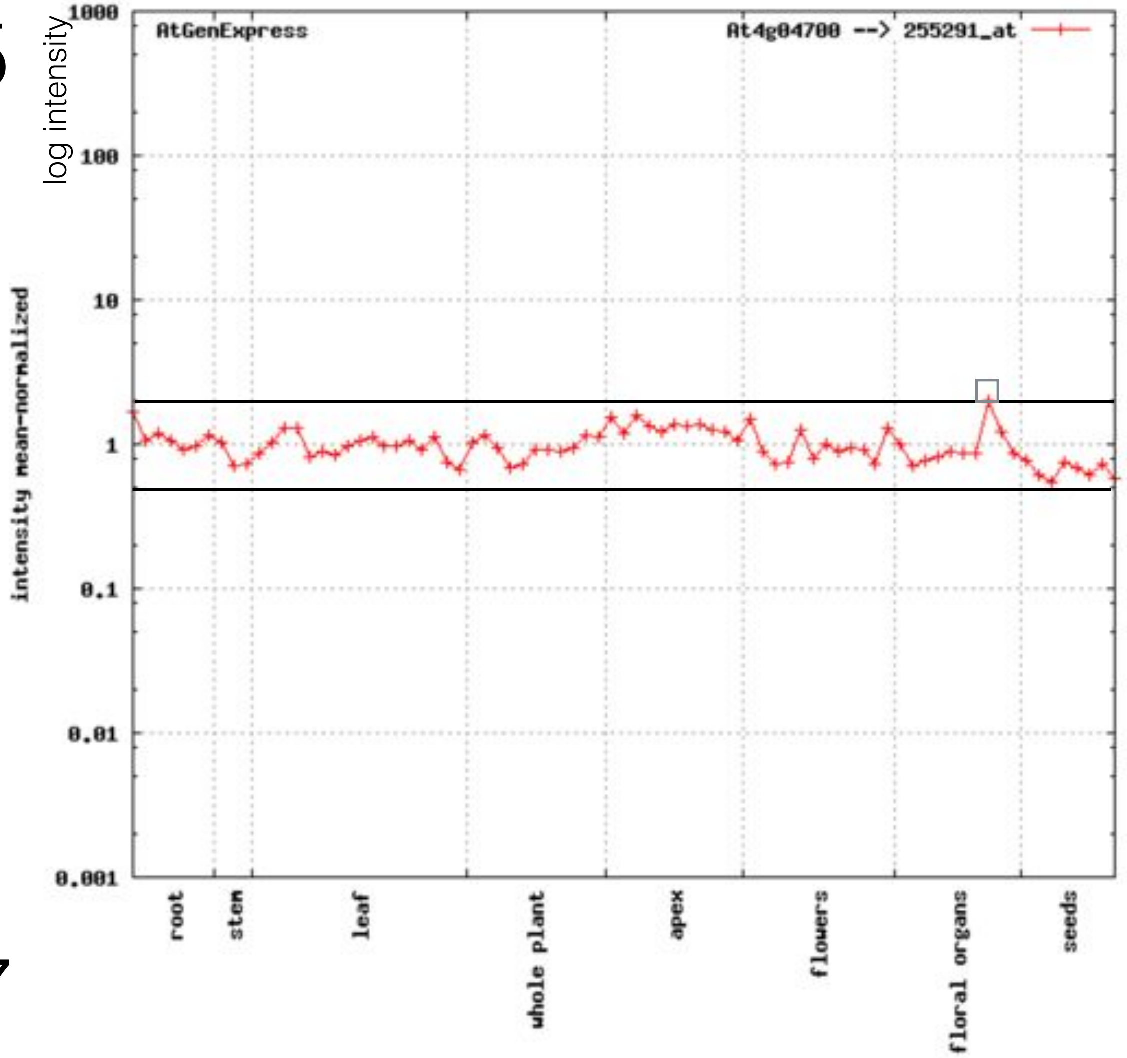
log intensity
intensity mean-normalized
AtGenExpress
At4g04700 --> 255291_at
1000
100
10
1
0.1
0.01
0.001
root
stem
leaf
whole plant
apex
flowers
floral organs
seeds

86

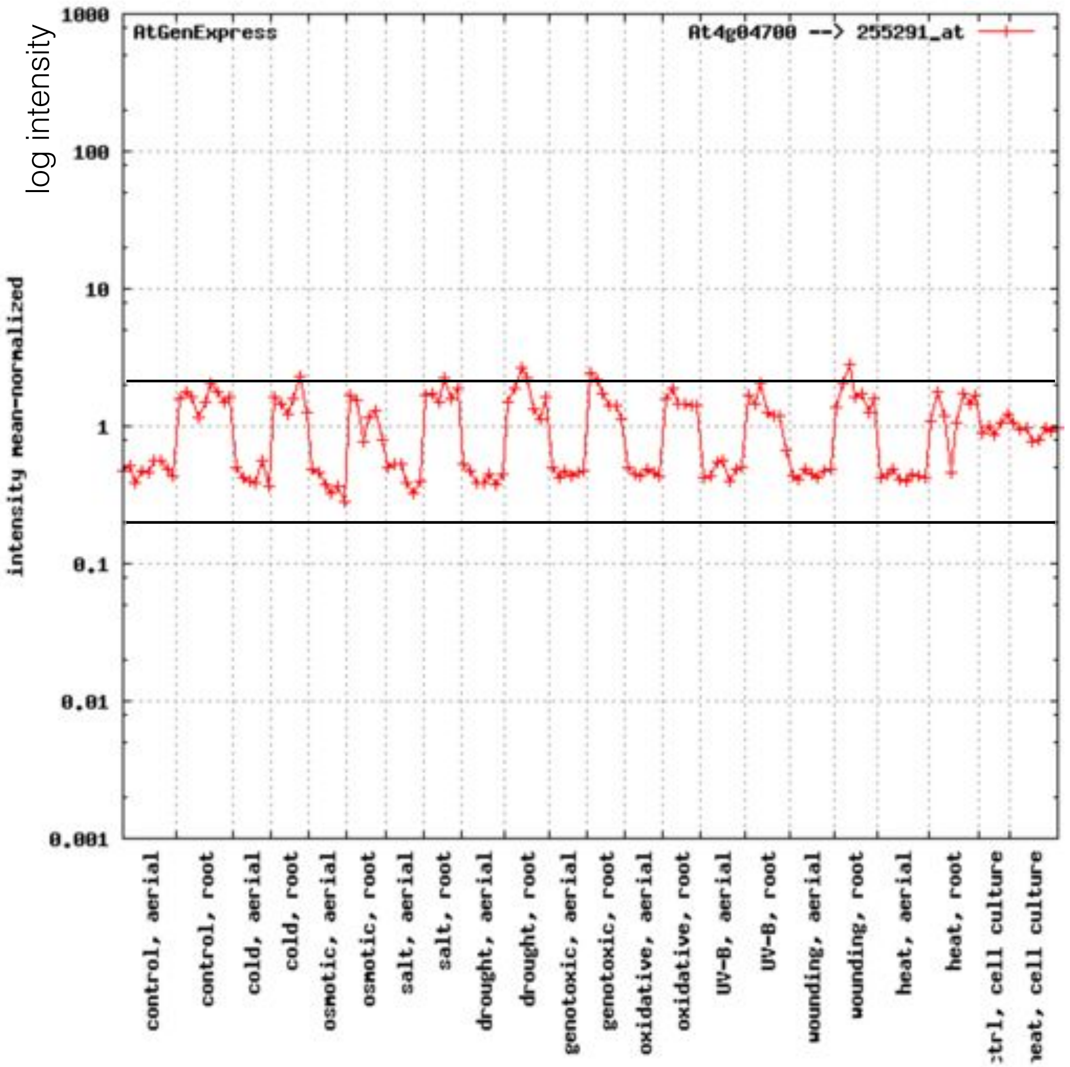
log intensity
intensity mean-normalized
AtGenExpress
At4g04700 --> 255291_at
1000
100
10
1
0.1
0.01
0.001
control, aerial
control, root
cold, aerial
cold, root
osmotic, aerial
osmotic, root
salt, aerial
salt, root
drought, aerial
drought, root
genotoxic, aerial
genotoxic, root
oxidative, aerial
oxidative, root
UV-B, aerial
UV-B, root
wounding, aerial
wounding, root
heat, aerial
heat, root
ctrl, cell culture
heat, cell culture

87

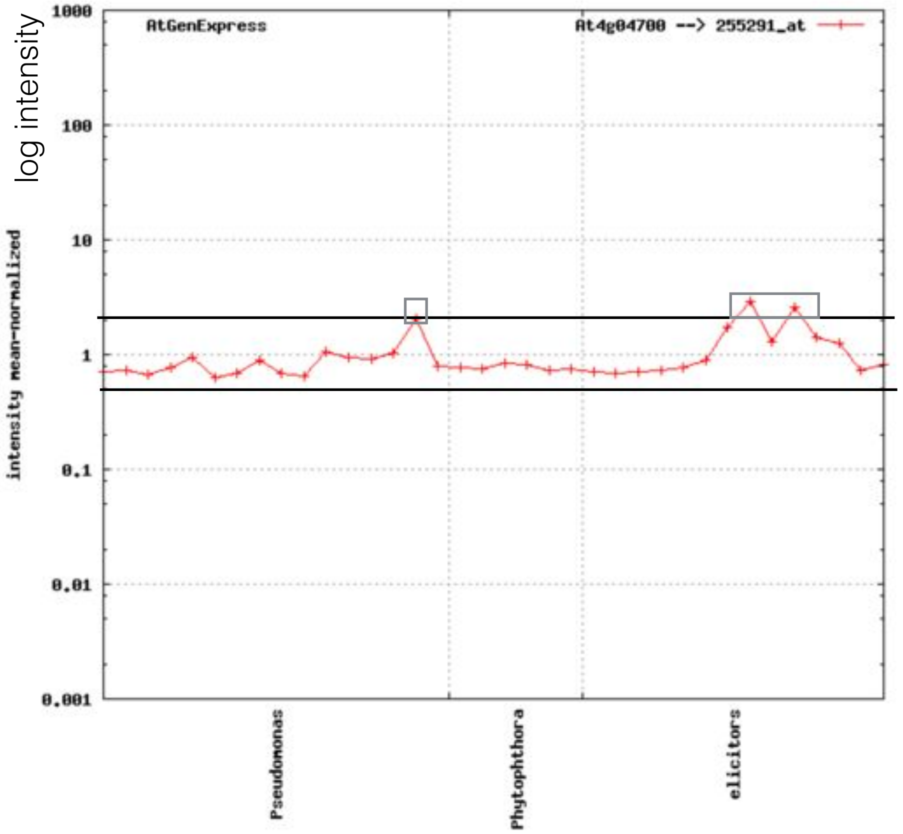
log intensity
intensity mean-normalized
AtGenExpress
At4g04700 --> 255291_at
1000
100
10
1
0.1
0.01
0.001
Pseudomonas
Phytophthora
elicitors

88

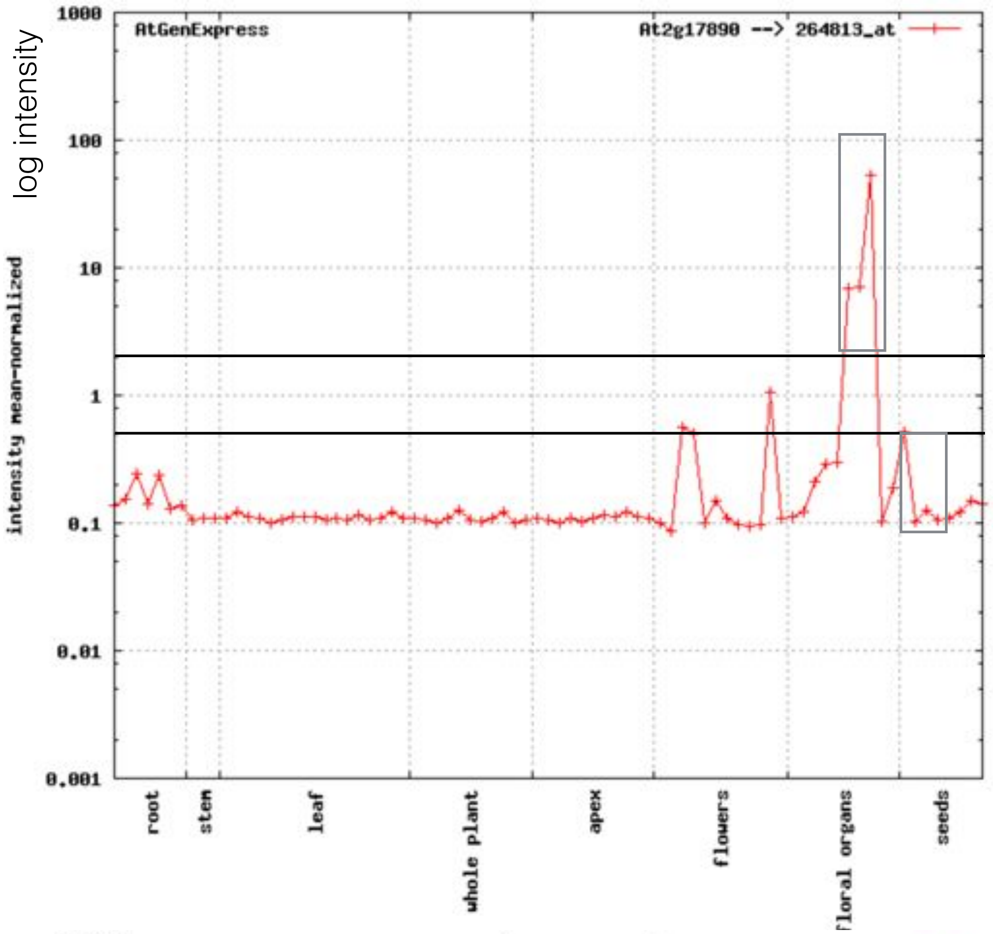

log intensity
intensity mean-normalized
AtGenExpress
At2g17890 --> 264813_at
1000
100
10
1
0.1
0.01
0.001
root
stem
leaf
whole plant
apex
flowers
floral organs
seeds

89

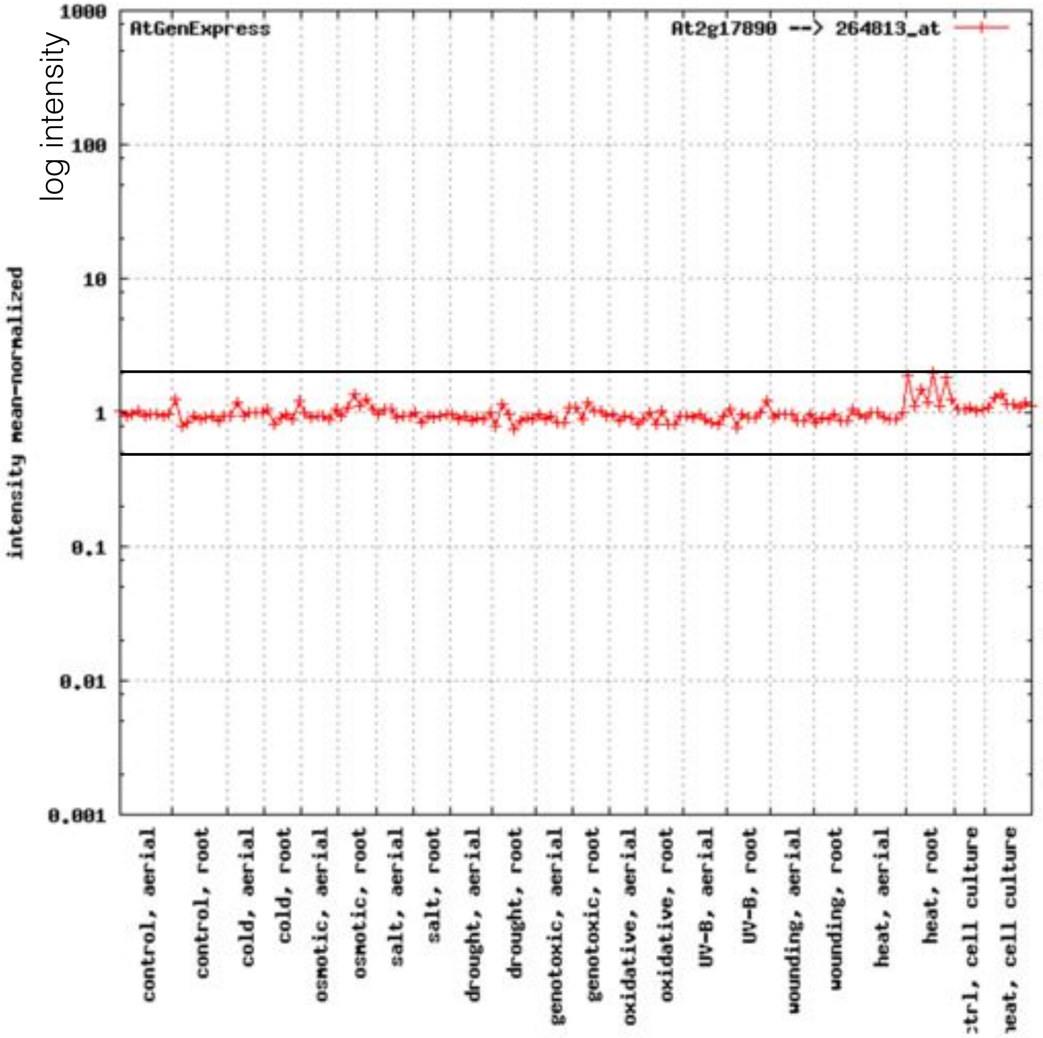

log intensity
intensity mean-normalized
AtGenExpress
At2g17890 --> 264813_at
1000
100
10
1
0.1
0.01
0.001
control, aerial
control, root
cold, aerial
cold, root
osmotic, aerial
osmotic, root
salt, aerial
salt, root
drought, aerial
drought, root
genotoxic, aerial
genotoxic, root
oxidative, aerial
oxidative, root
UV-B, aerial
UV-B, root
wounding, aerial
wounding, root
heat, aerial
heat, root

90

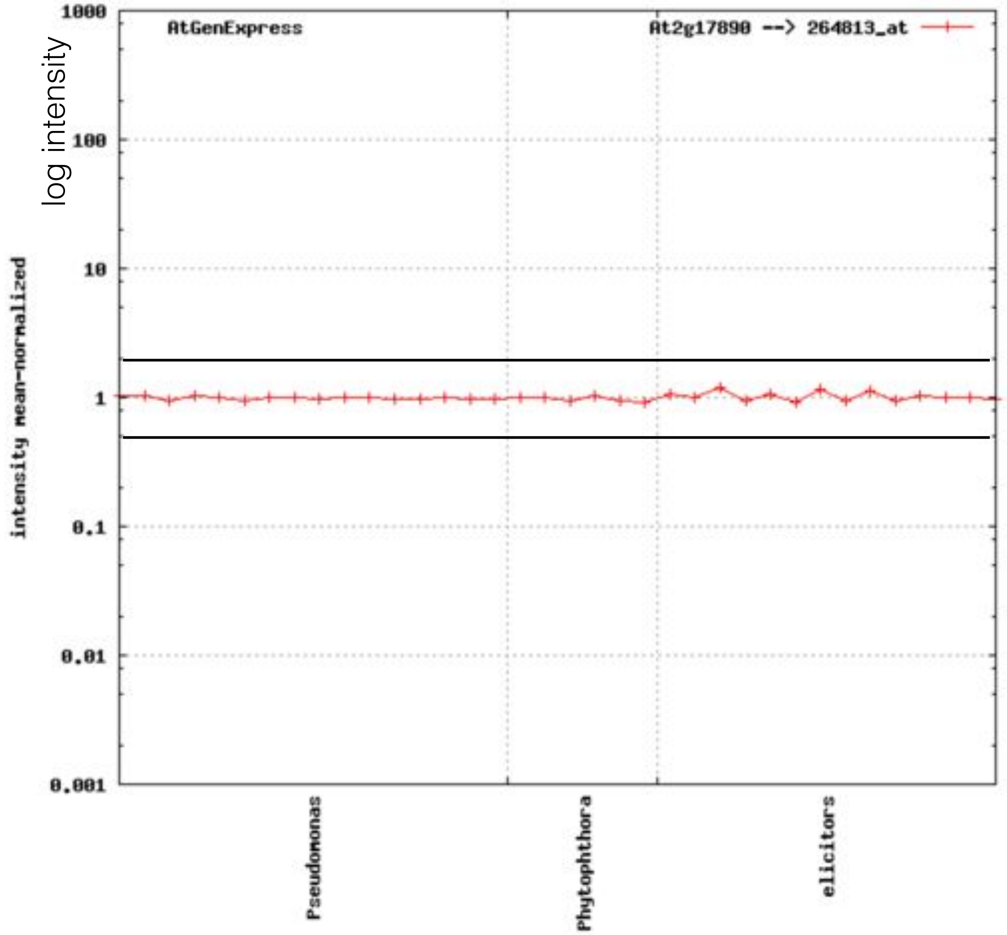

log intensity
intensity mean-normalized
AtGenExpress
At2g17890 --> 264813_at
1000
100
10
1
0.1
0.01
0.001
Pseudomonas
Phytophthora
elicitors

91

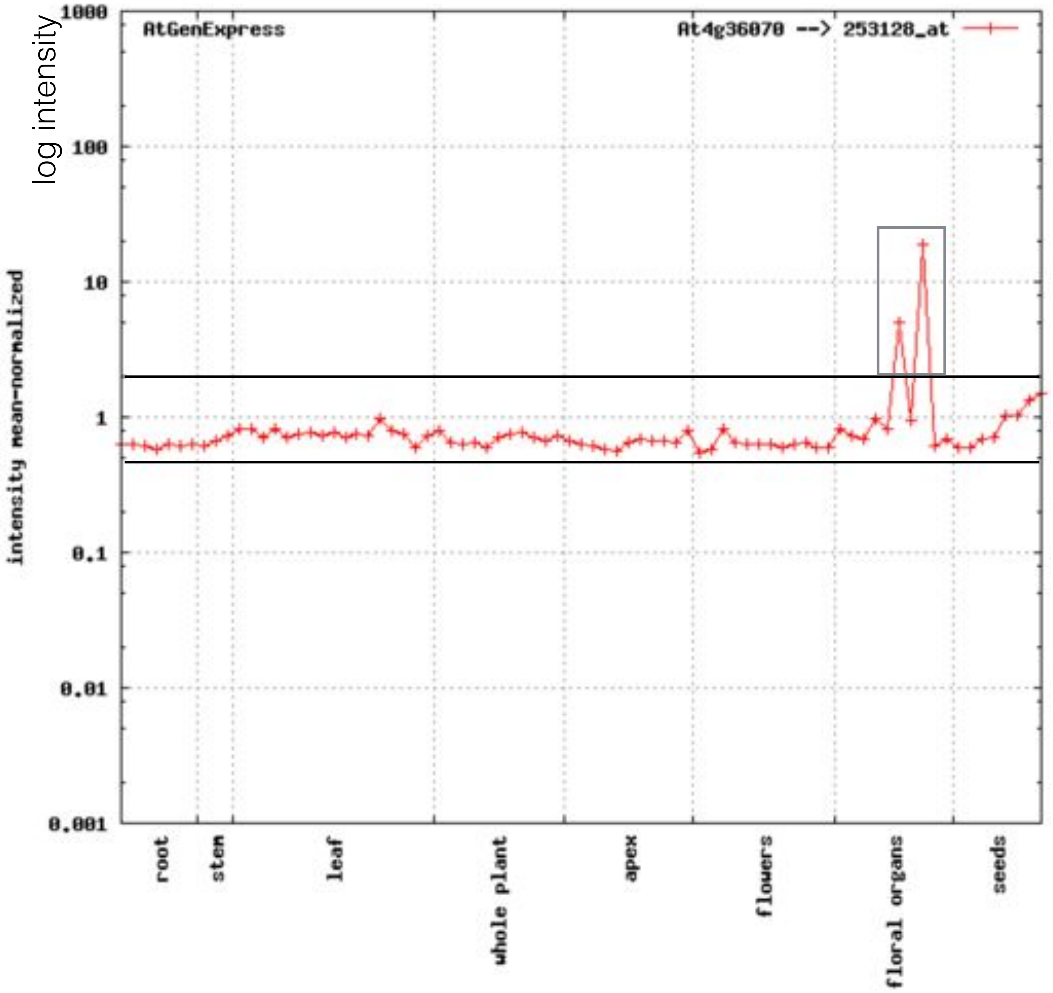
log intensity
intensity mean-normalized
AtGenExpress
At4g36070 --> 253128_at
1000
100
10
1
0.1
0.01
0.001
root
stem
leaf
whole plant
apex
flowers
floral organs
seeds

92

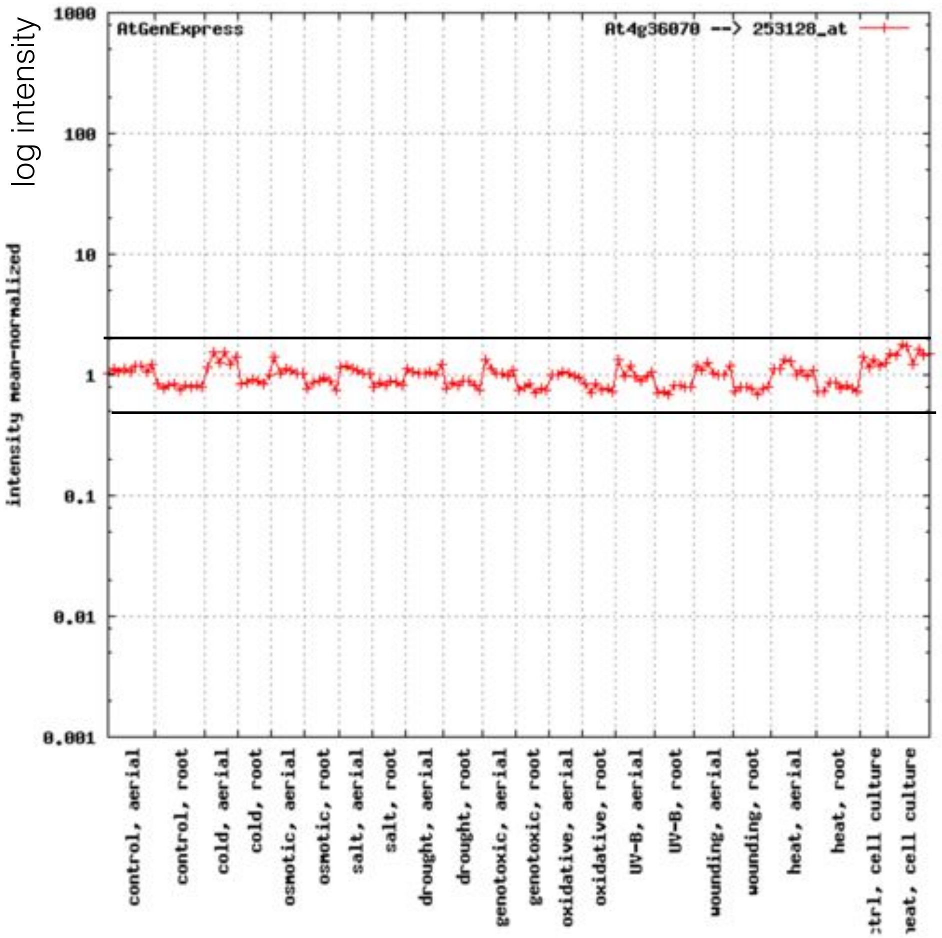
log intensity
intensity mean-normalized
AtGenExpress
At4g36070 --> 253128_at
1000
100
10
1
0.1
0.01
0.001
control, aerial
control, root
cold, aerial
cold, root
osmotic, aerial
osmotic, root
salt, aerial
salt, root
drought, aerial
drought, root
genotoxic, aerial
genotoxic, root
oxidative, aerial
oxidative, root
UV-B, aerial
UV-B, root
wounding, aerial
wounding, root
heat, aerial
heat, root
ctrl, cell culture
heat, cell culture

93

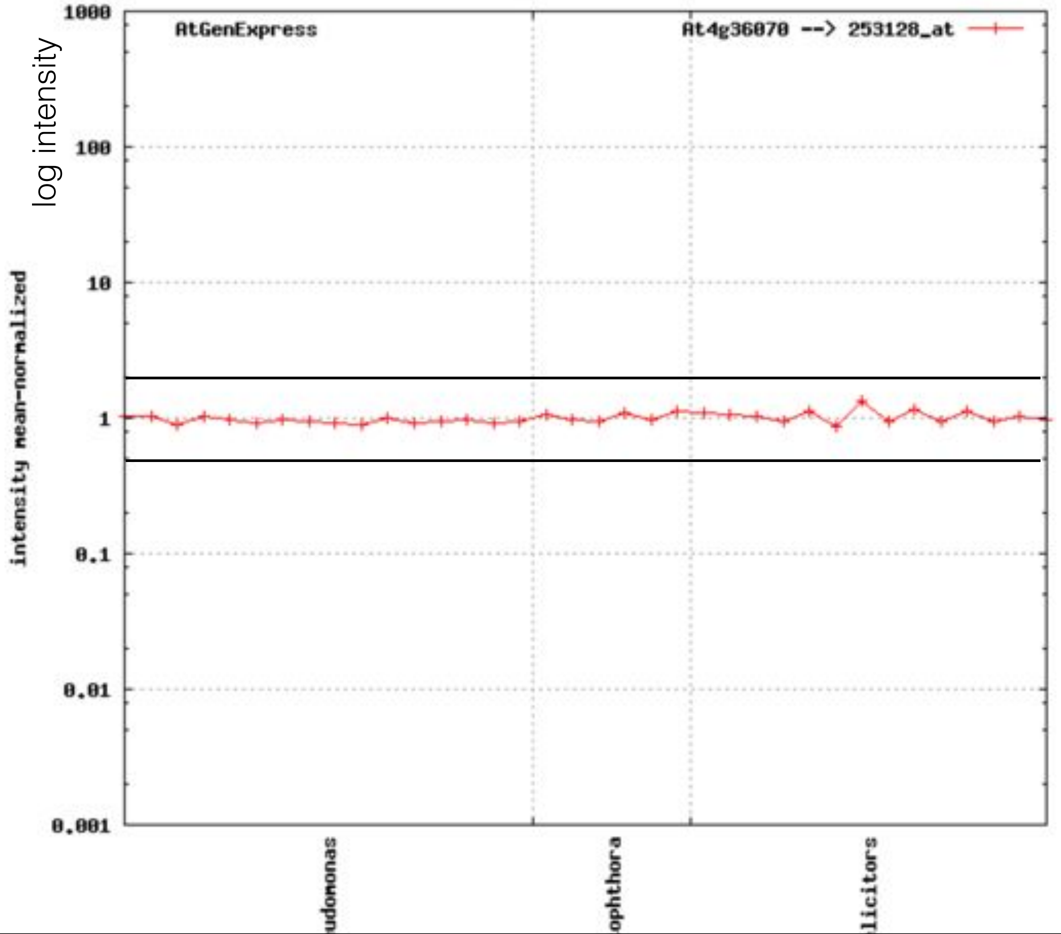
log intensity
intensity mean-normalized
AtGenExpress
At4g36070 --> 253128_at
1000
100
10
1
0.1
0.01
0.001
udomonas
ophthora
licitors

94

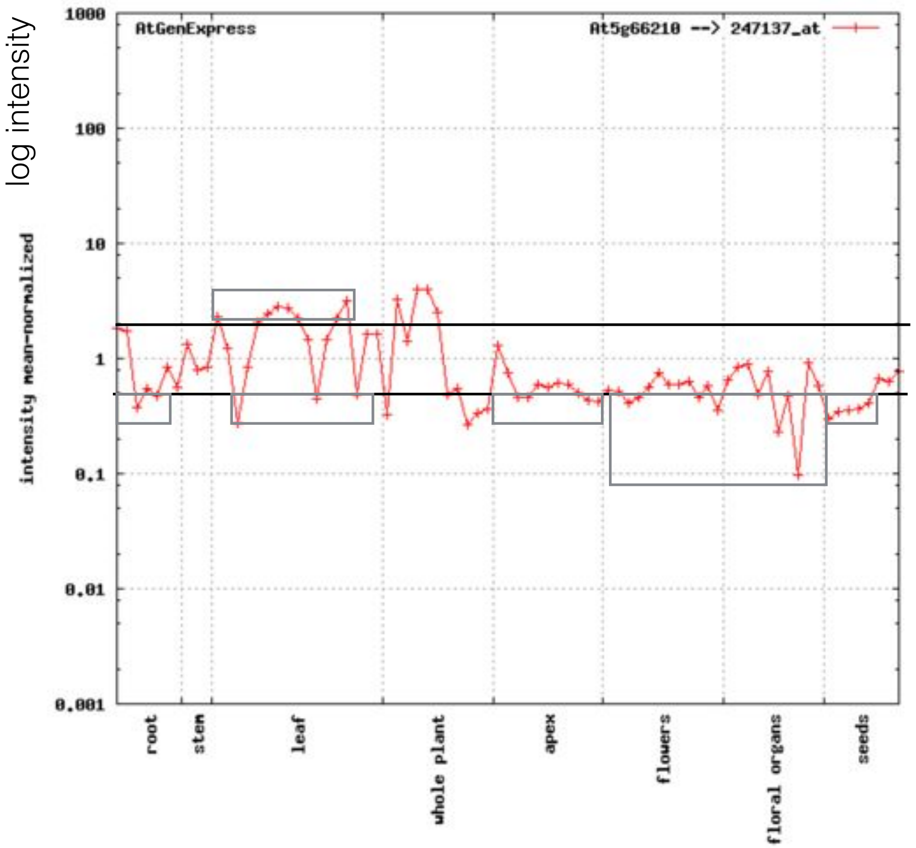
log intensity
AtGenExpress
At5g66210 --> 247137_at
intensity mean-normalized
1000
100
10
1
0.1
0.01
0.001
root
stem
leaf
whole plant
apex
flowers
floral organs
seeds

95

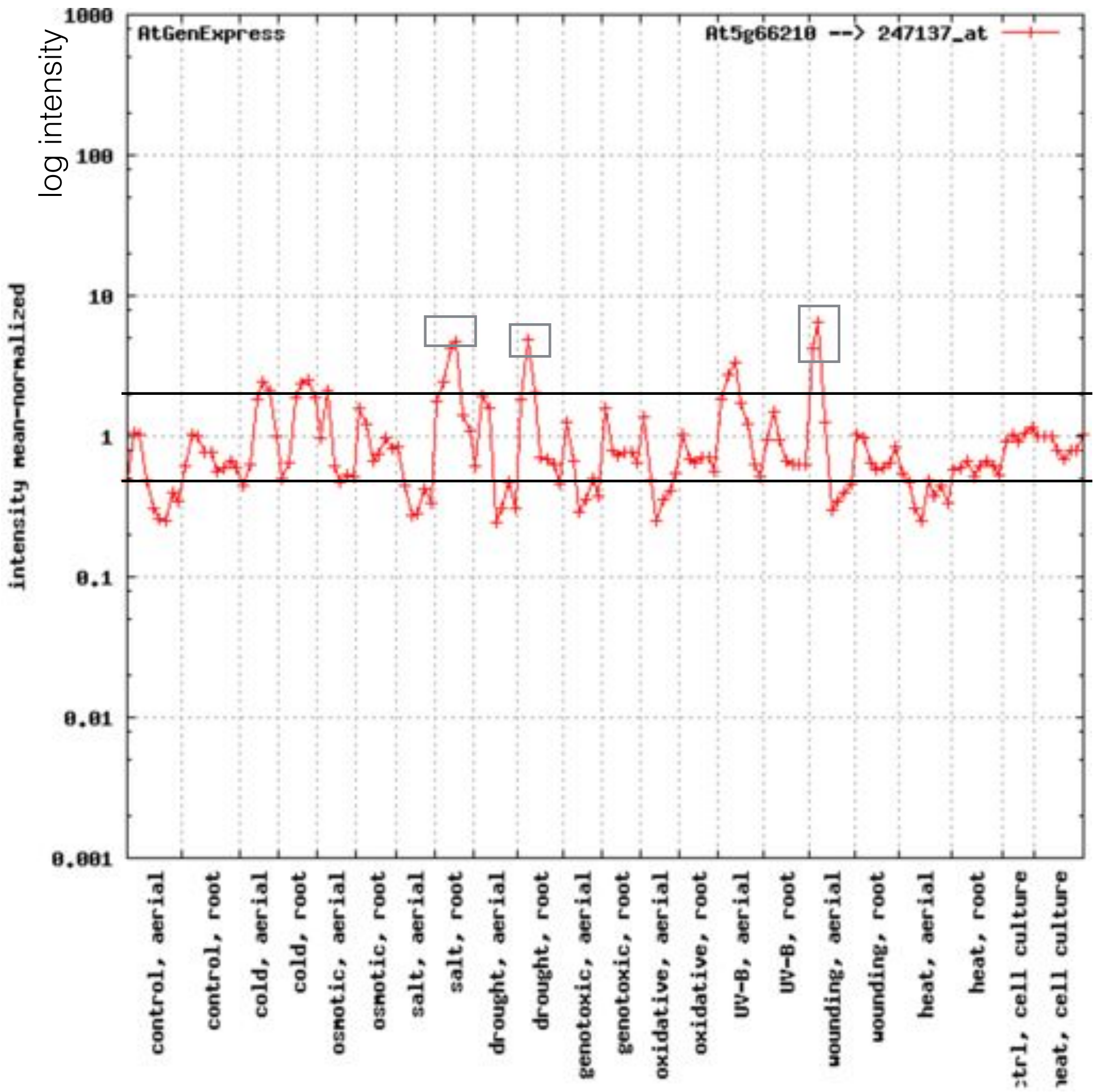
log intensity
AtGenExpress
At5g66210 --> 247137_at
intensity mean-normalized
1000
100
10
1
0.1
0.01
0.001
control, aerial
control, root
cold, aerial
cold, root
osmotic, aerial
osmotic, root
salt, aerial
salt, root
drought, aerial
drought, root
genotoxic, aerial
genotoxic, root
oxidative, aerial
oxidative, root
UV-B, aerial
UV-B, root
wounding, aerial
wounding, root
heat, aerial
heat, root

96

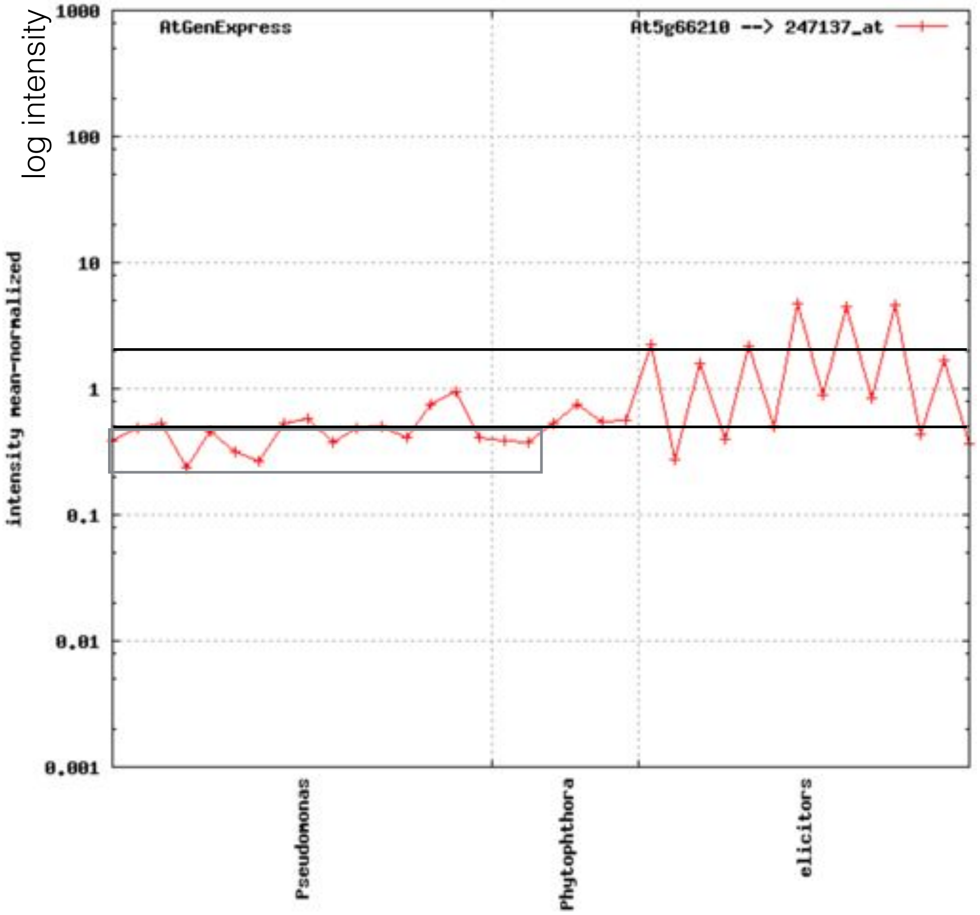
log intensity
AtGenExpress
At5g66210 --> 247137_at
intensity mean-normalized
1000
100
10
1
0.1
0.01
0.001
Pseudomonas
Phytophthora
elicitors

97

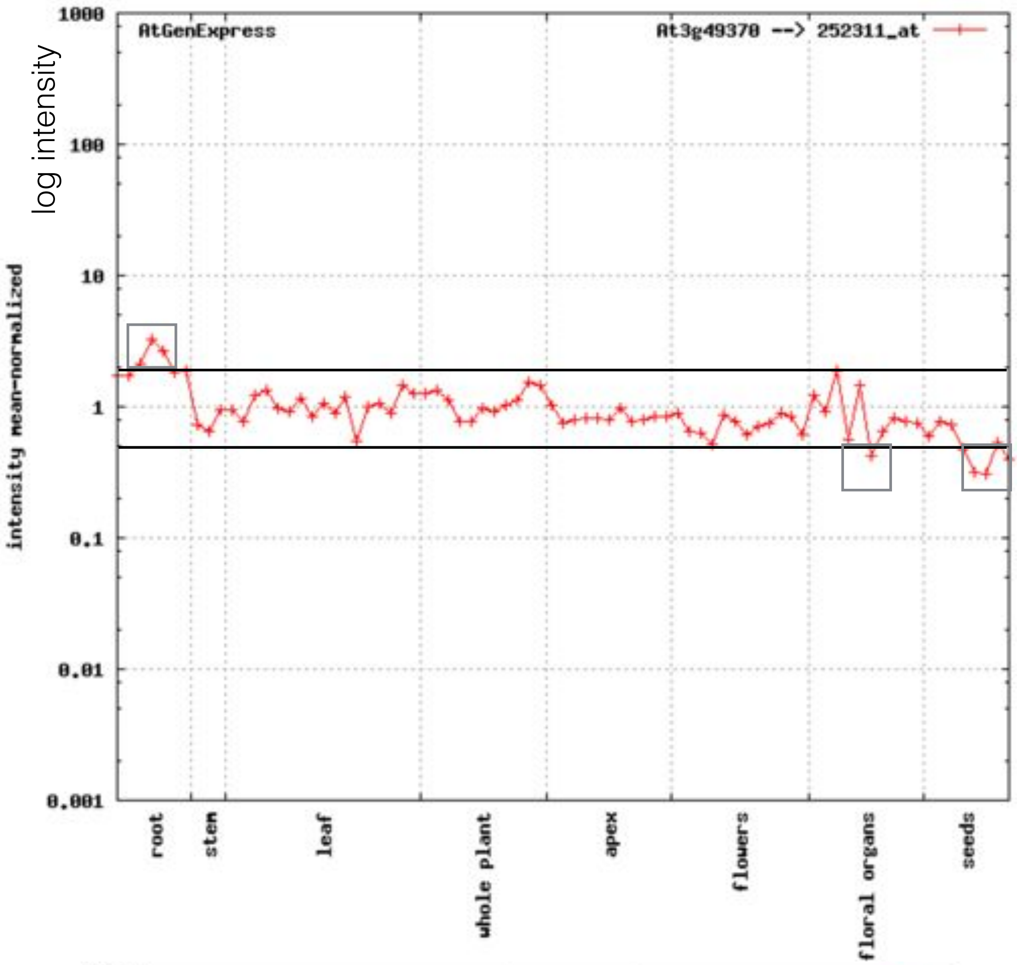

log intensity
intensity mean-normalized
AtGenExpress
At3g49370 --> 252311_at
1000
100
10
1
0.1
0.01
0.001
root
stem
leaf
whole plant
apex
flowers
floral organs
seeds

98

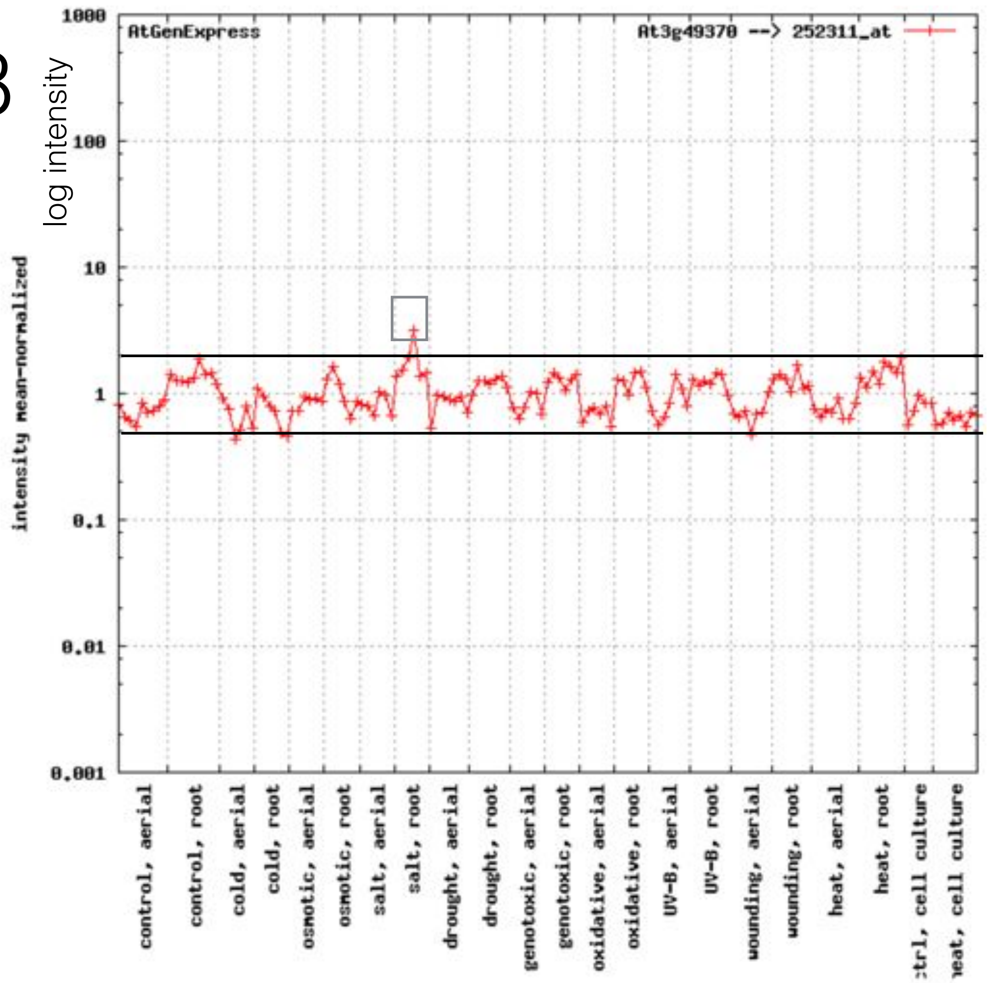

log intensity
intensity mean-normalized
AtGenExpress
At3g49370 --> 252311_at
1000
100
10
1
0.1
0.01
0.001
control, aerial
control, root
cold, aerial
cold, root
osmotic, aerial
osmotic, root
salt, aerial
salt, root
drought, aerial
drought, root
genotoxic, aerial
genotoxic, root
oxidative, aerial
oxidative, root
UV-B, aerial
UV-B, root
wounding, aerial
wounding, root
heat, aerial
heat, root

99

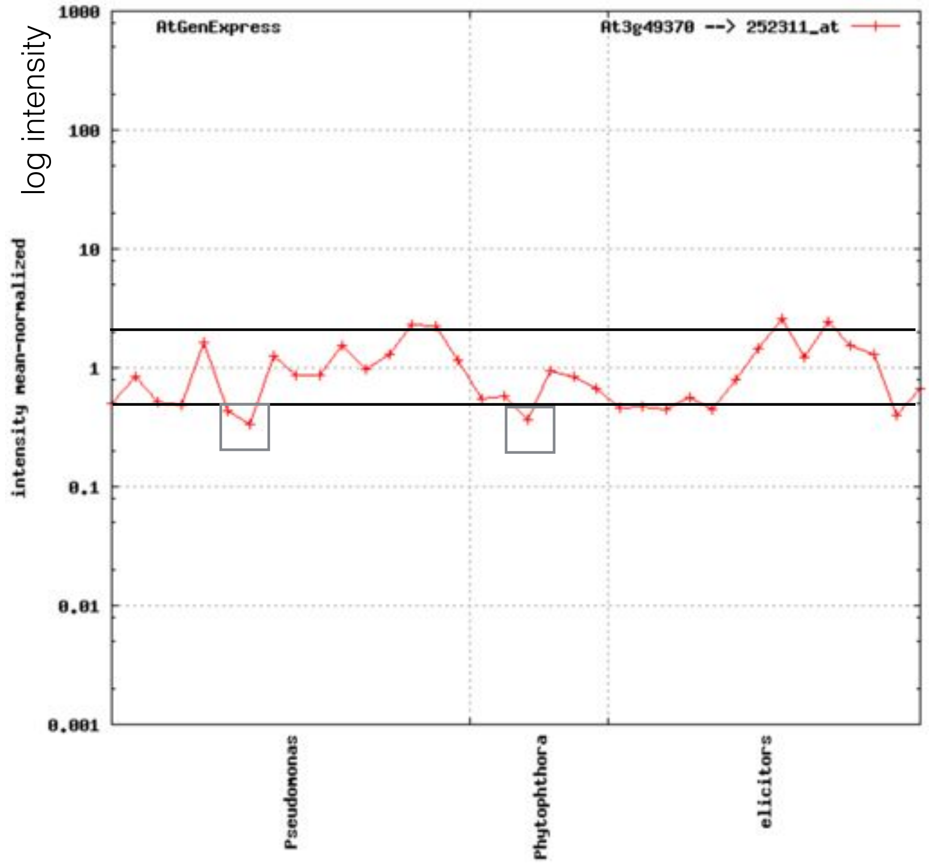

log intensity
intensity mean-normalized
AtGenExpress
At3g49370 --> 252311_at
1000
100
10
1
0.1
0.01
0.001
Pseudomonas
Phytophthora
elicitors

100

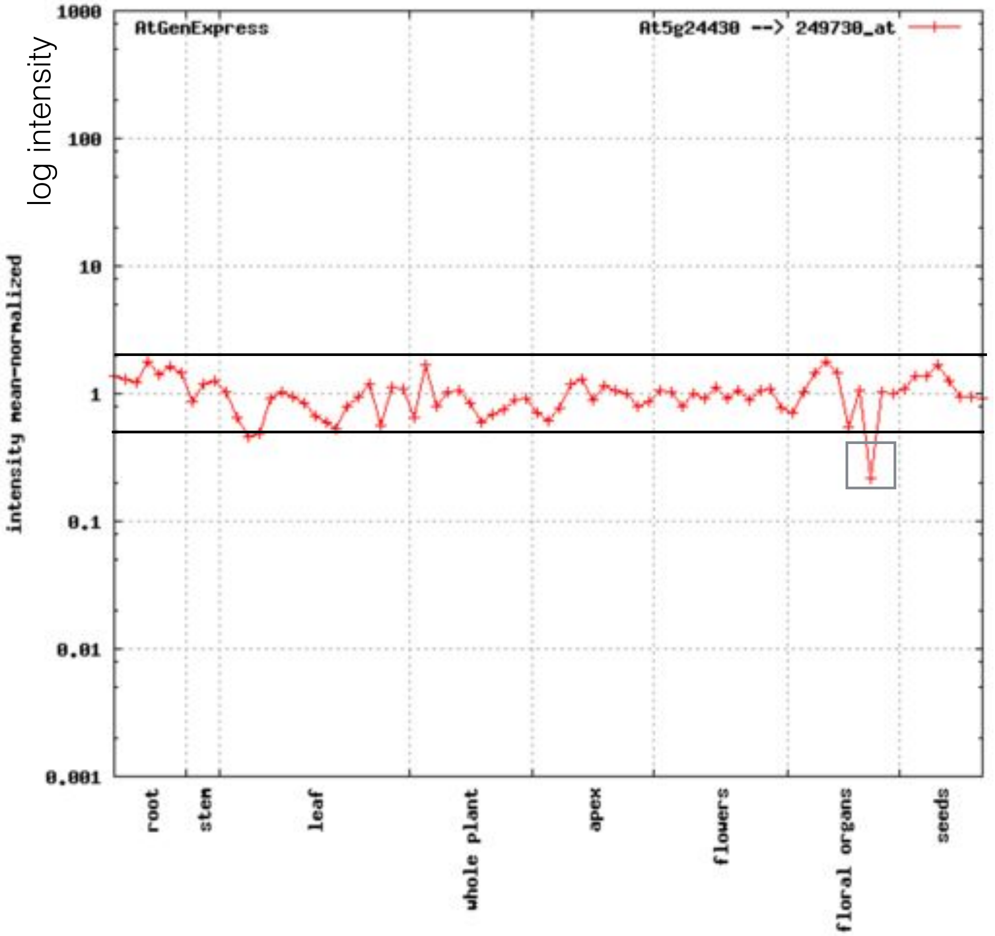
AtGenExpress
At5g24430 --> 249730_at
log intensity
intensity mean-normalized
1000
100
10
1
0.1
0.01
0.001
root
stem
leaf
whole plant
apex
flowers
floral organs
seeds

101

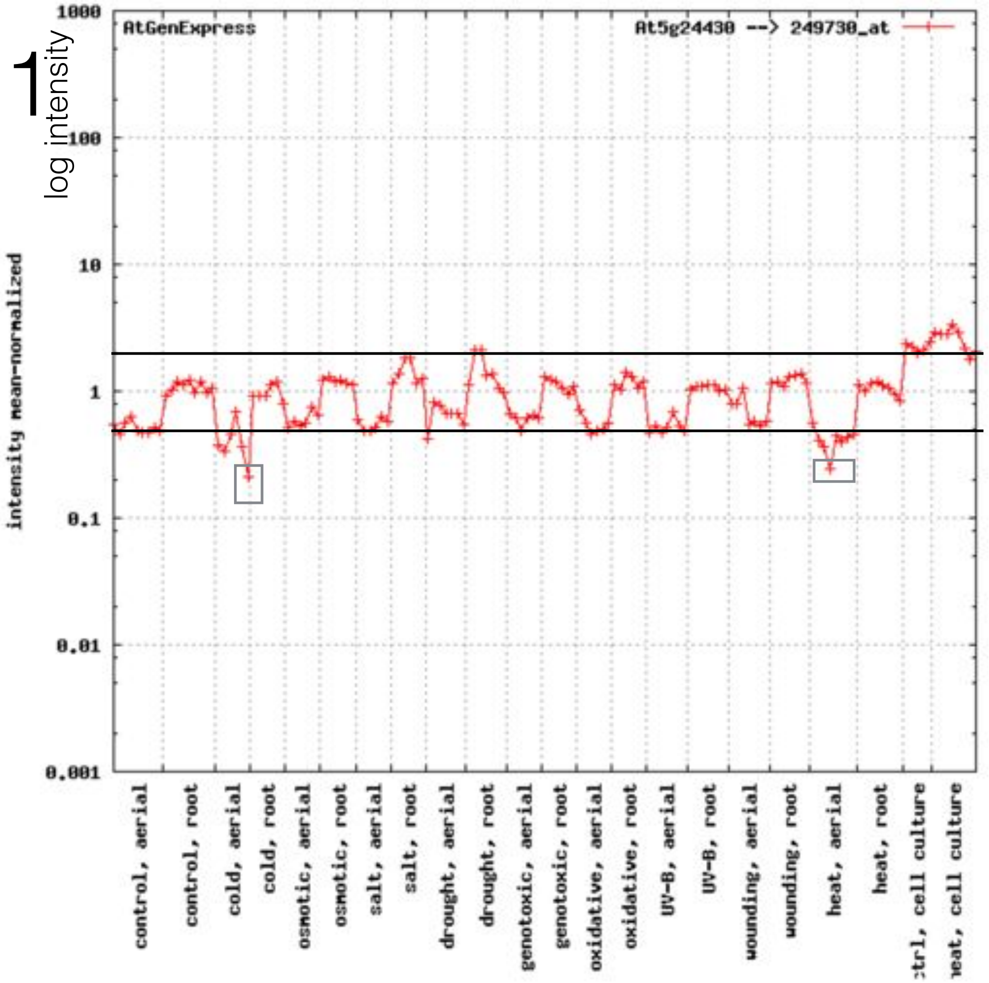
AtGenExpress
At5g24430 --> 249730_at
log intensity
intensity mean-normalized
1000
100
10
1
0.1
0.01
0.001
control, aerial
control, root
cold, aerial
cold, root
osmotic, aerial
osmotic, root
salt, aerial
salt, root
drought, aerial
drought, root
genotoxic, aerial
genotoxic, root
oxidative, aerial
oxidative, root
UV-B, aerial
UV-B, root
wounding, aerial
wounding, root
heat, aerial
heat, root

102

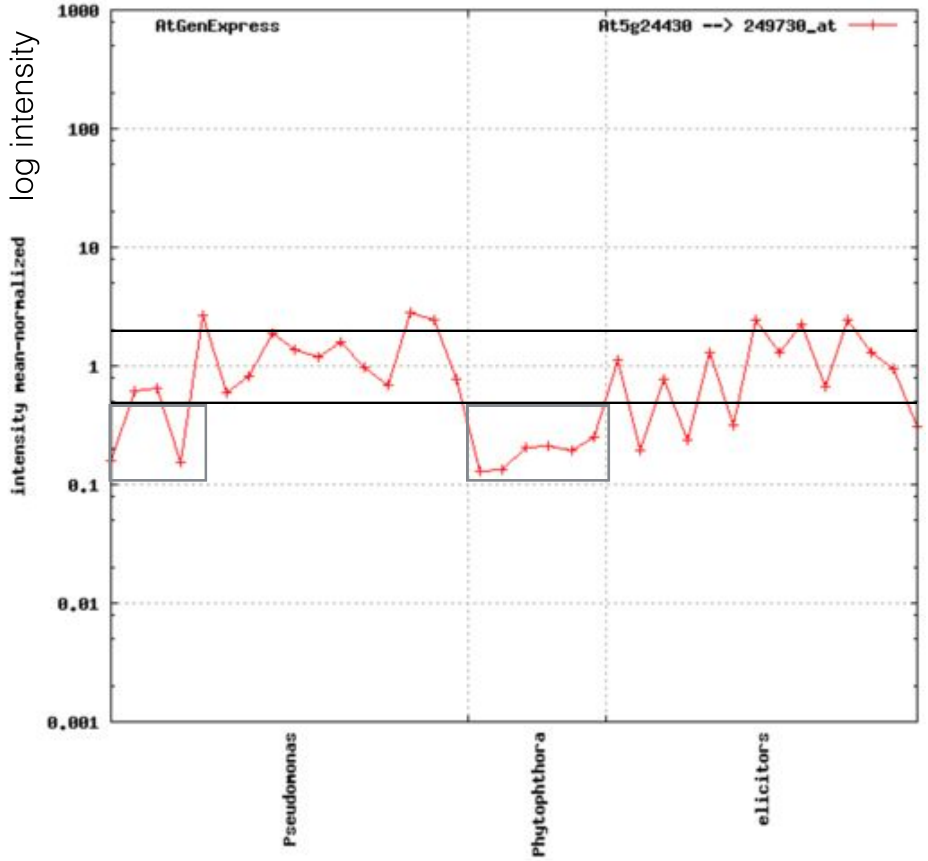
AtGenExpress
At5g24430 --> 249730_at
log intensity
intensity mean-normalized
1000
100
10
1
0.1
0.01
0.001
Pseudomonas
Phytophthora
elicitors

103

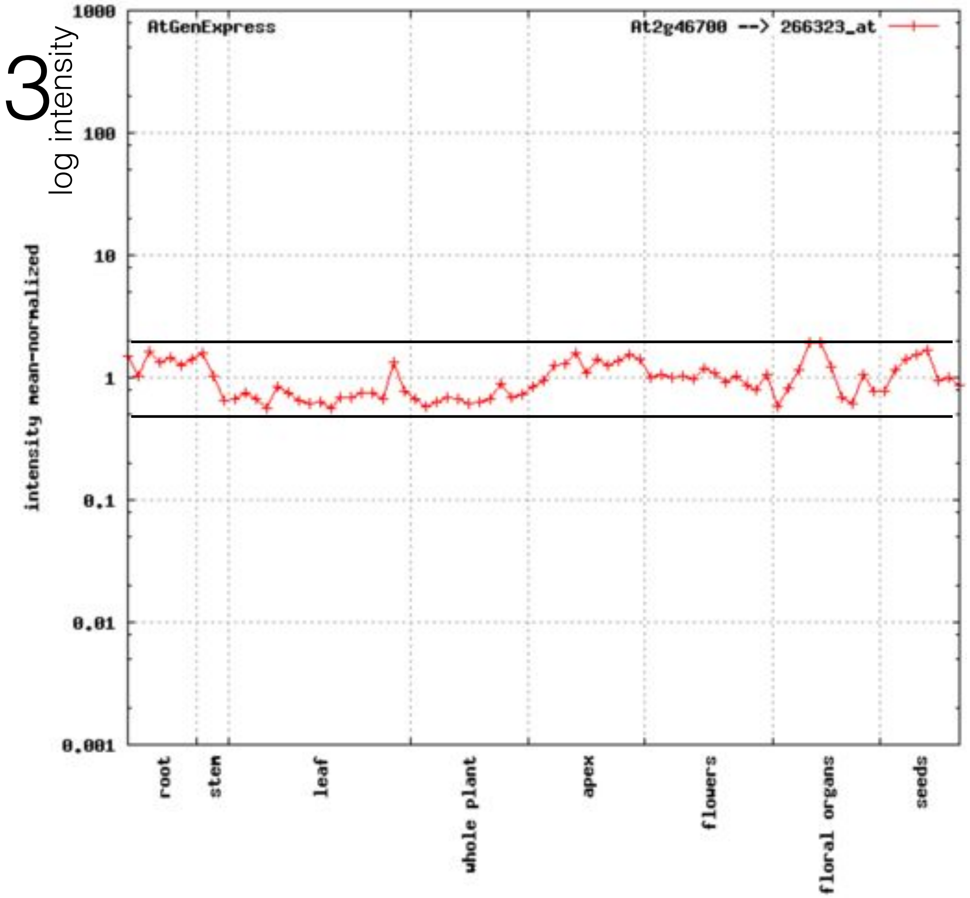
log intensity
intensity mean-normalized
AtGenExpress
At2g46700 --> 266323_at
1000
100
10
1
0.1
0.01
0.001
root
stem
leaf
whole plant
apex
flowers
floral organs
seeds

104

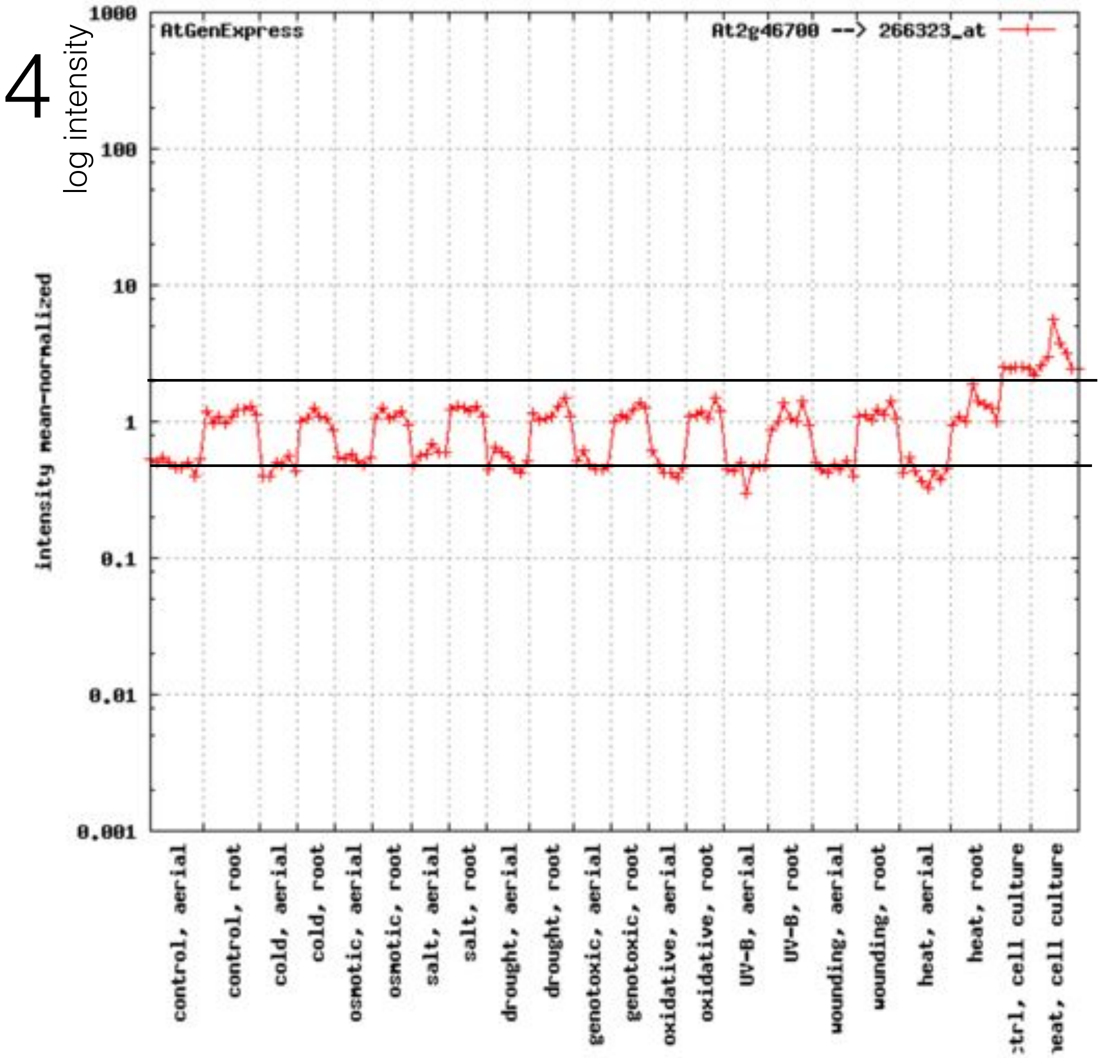
log intensity
intensity mean-normalized
AtGenExpress
At2g46700 --> 266323_at
1000
100
10
1
0.1
0.01
0.001
control, aerial
control, root
cold, aerial
cold, root
osmotic, aerial
osmotic, root
salt, aerial
salt, root
drought, aerial
drought, root
genotoxic, aerial
genotoxic, root
oxidative, aerial
oxidative, root
UV-B, aerial
UV-B, root
wounding, aerial
wounding, root
heat, aerial
heat, root
ctrl, cell culture
heat, cell culture

105

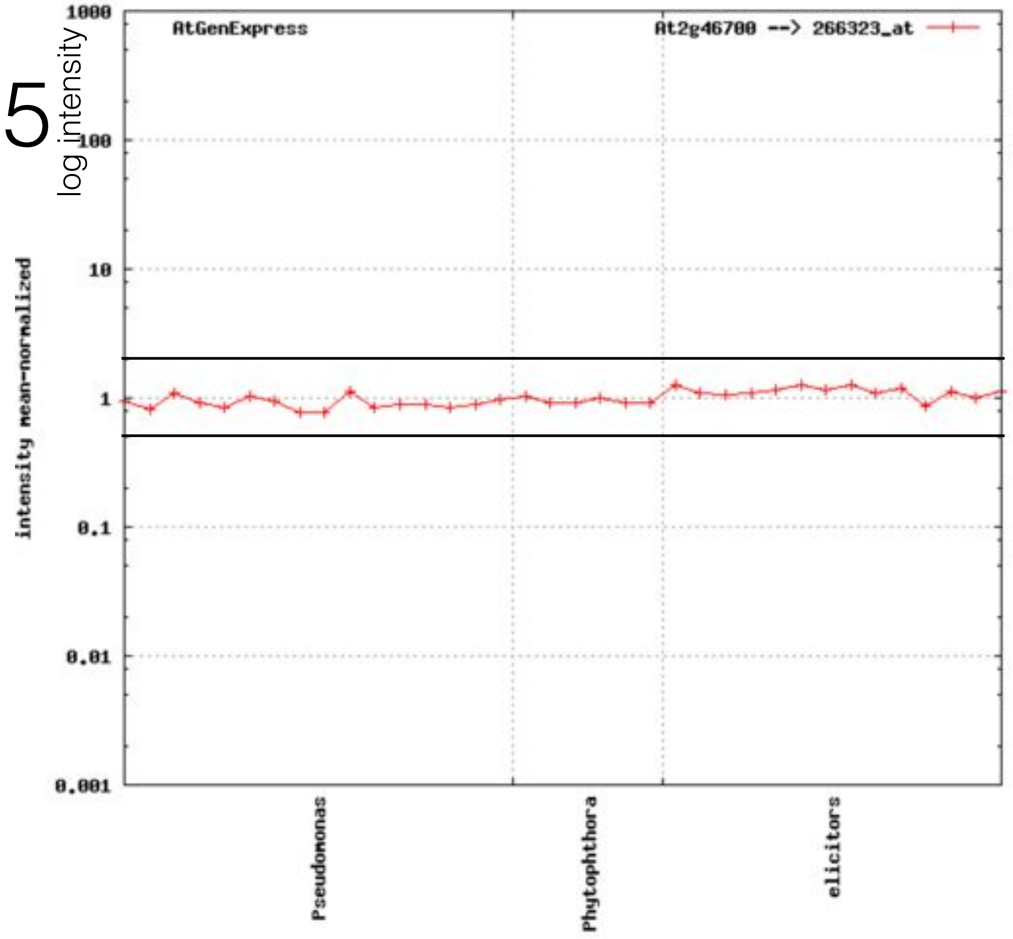
log intensity
intensity mean-normalized
AtGenExpress
At2g46700 --> 266323_at
1000
100
10
1
0.1
0.01
0.001
Pseudomonas
Phytophthora
elicitors

106

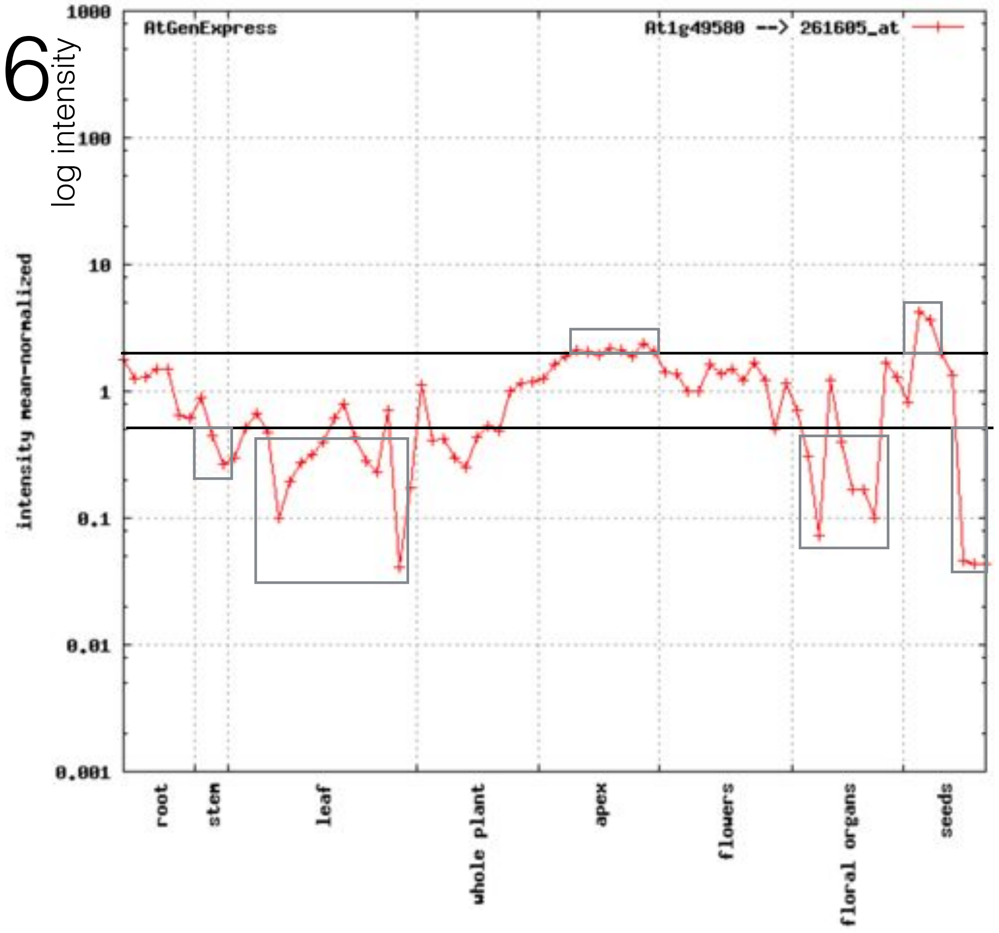
AtGenExpress
At1g49580 --> 261605_at
log intensity
intensity mean-normalized
1000
100
10
1
0.1
0.01
0.001
root
stem
leaf
whole plant
apex
flowers
floral organs
seeds

107

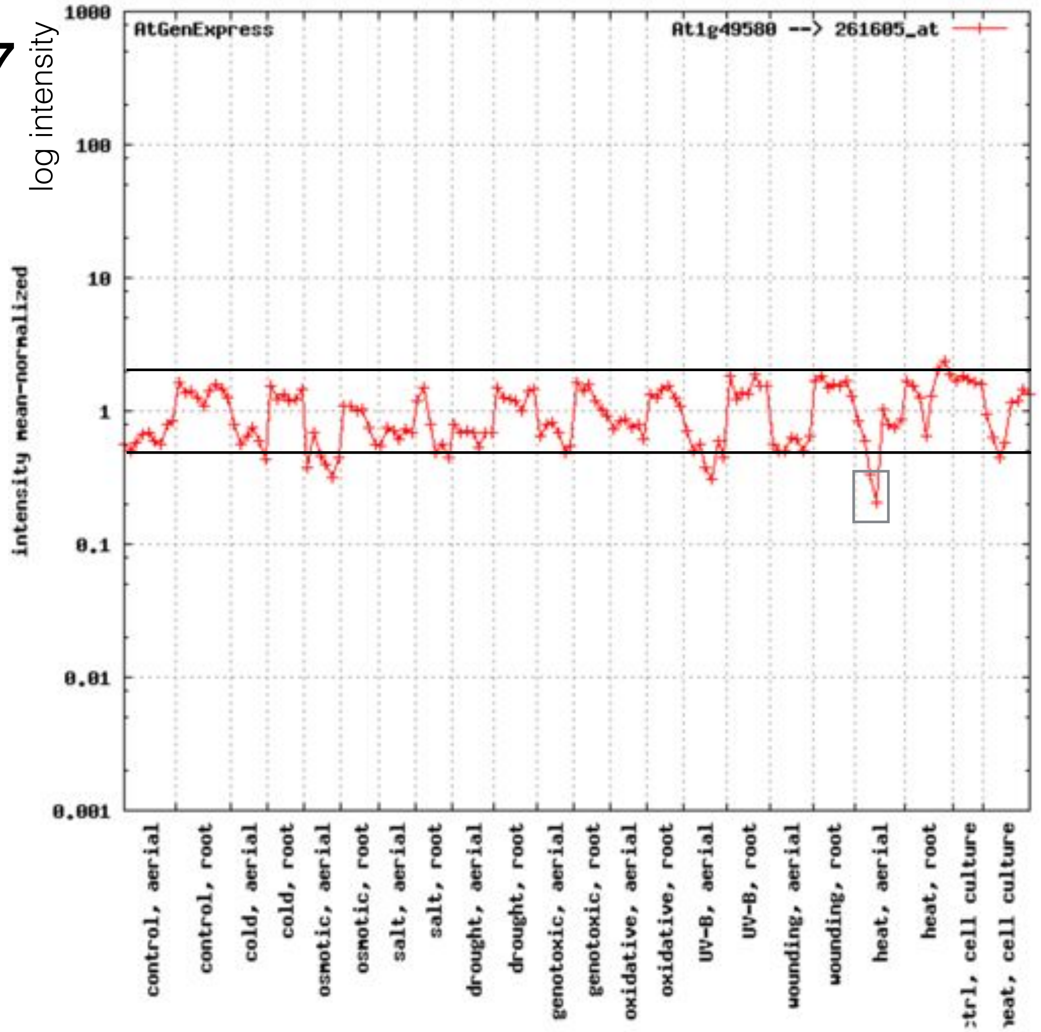
AtGenExpress
At1g49580 --> 261605_at
log intensity
intensity mean-normalized
1000
100
10
1
0.1
0.01
0.001
control, aerial
control, root
cold, aerial
cold, root
osmotic, aerial
osmotic, root
salt, aerial
salt, root
drought, aerial
drought, root
genotoxic, aerial
genotoxic, root
oxidative, aerial
oxidative, root
UV-B, aerial
UV-B, root
wounding, aerial
wounding, root
heat, aerial
heat, root
:trl, cell culture
ıeat, cell culture

108

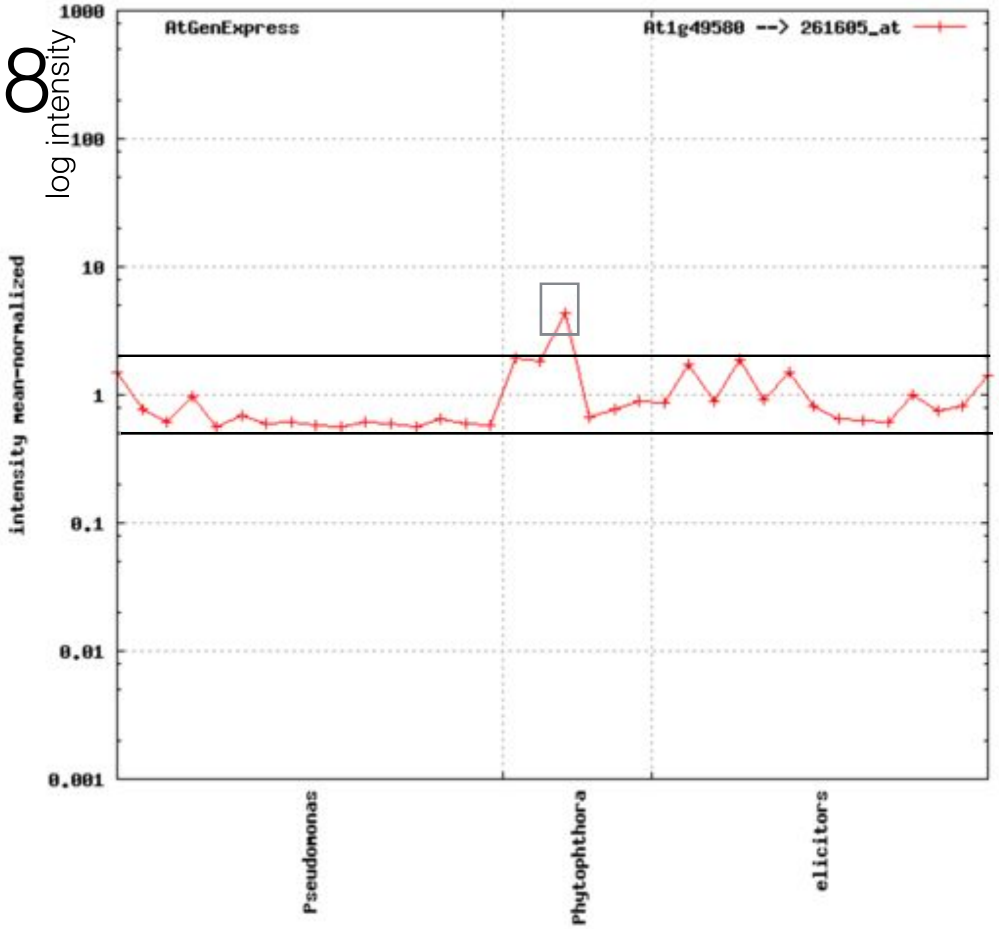
AtGenExpress
At1g49580 --> 261605_at
log intensity
intensity mean-normalized
1000
100
10
1
0.1
0.01
0.001
Pseudomonas
Phytophthora
elicitors

109

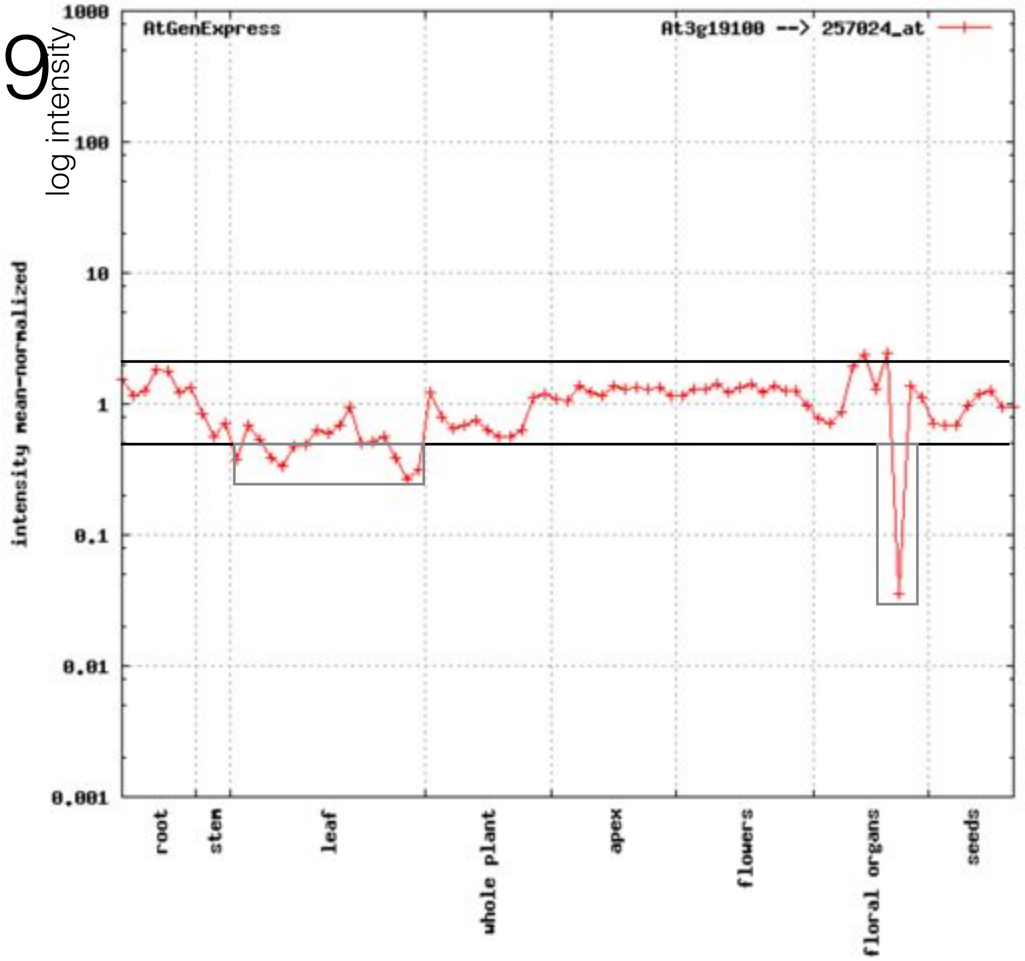
AtGenExpress
At3g19100 --> 257024_at
log intensity
intensity mean-normalized
1000
100
10
1
0.1
0.01
0.001
root
stem
leaf
whole plant
apex
flowers
floral organs
seeds

110

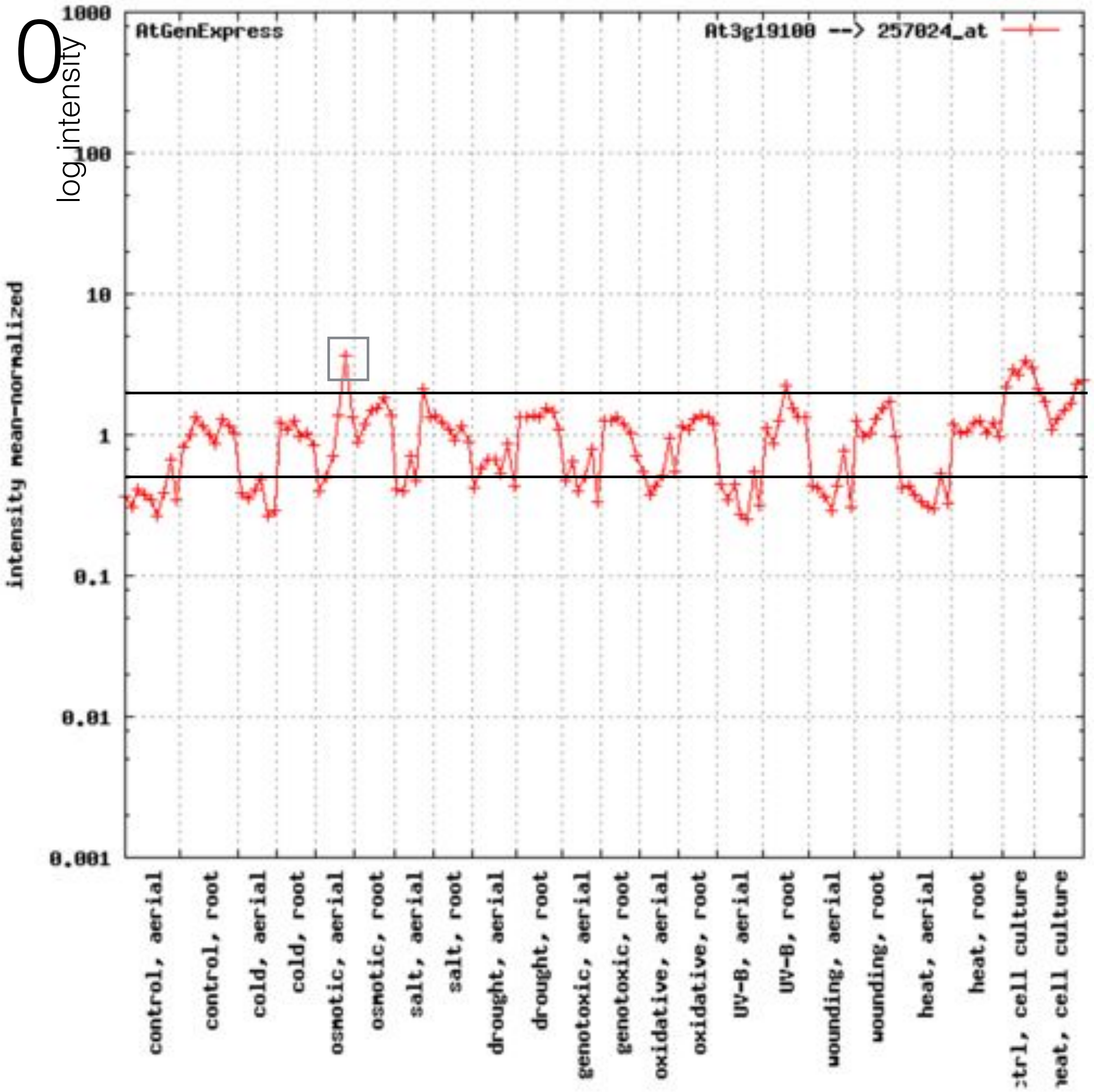
AtGenExpress
At3g19100 --> 257024_at
log intensity
intensity mean-normalized
1000
100
10
1
0.1
0.01
0.001
control, aerial
control, root
cold, aerial
cold, root
osmotic, aerial
osmotic, root
salt, aerial
salt, root
drought, aerial
drought, root
genotoxic, aerial
genotoxic, root
oxidative, aerial
oxidative, root
UV-B, aerial
UV-B, root
wounding, aerial
wounding, root
heat, aerial
heat, root
ctrl, cell culture
heat, cell culture

111

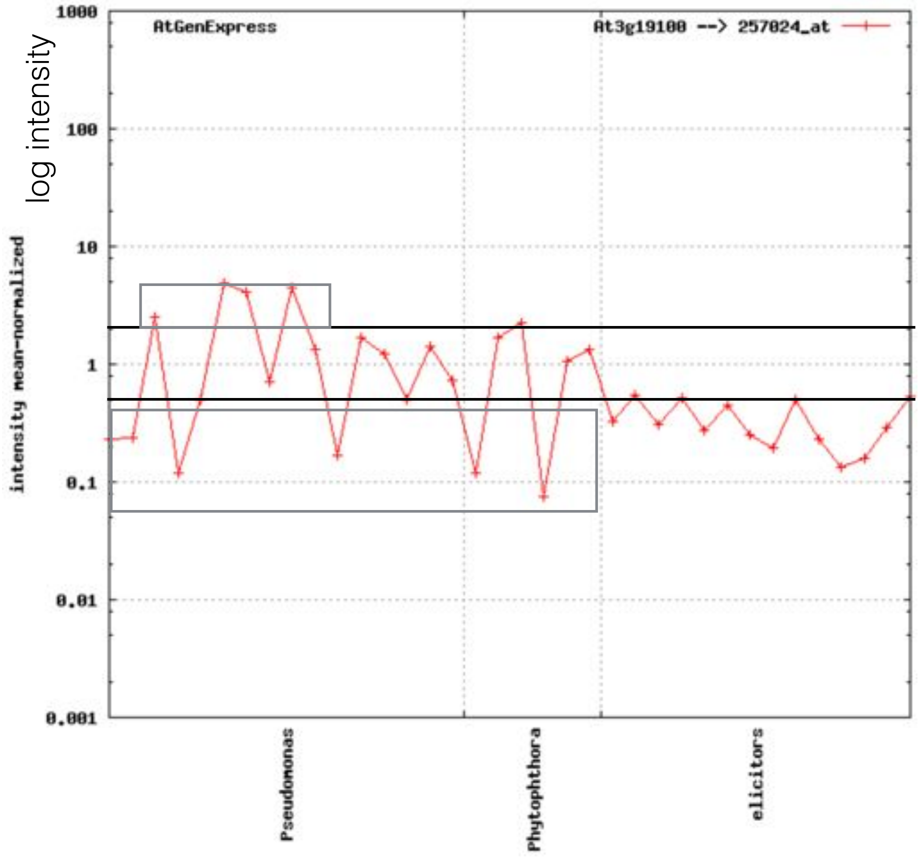
AtGenExpress
At3g19100 --> 257024_at
log intensity
intensity mean-normalized
1000
100
10
1
0.1
0.01
0.001
Pseudomonas
Phytophthora
elicitors

112

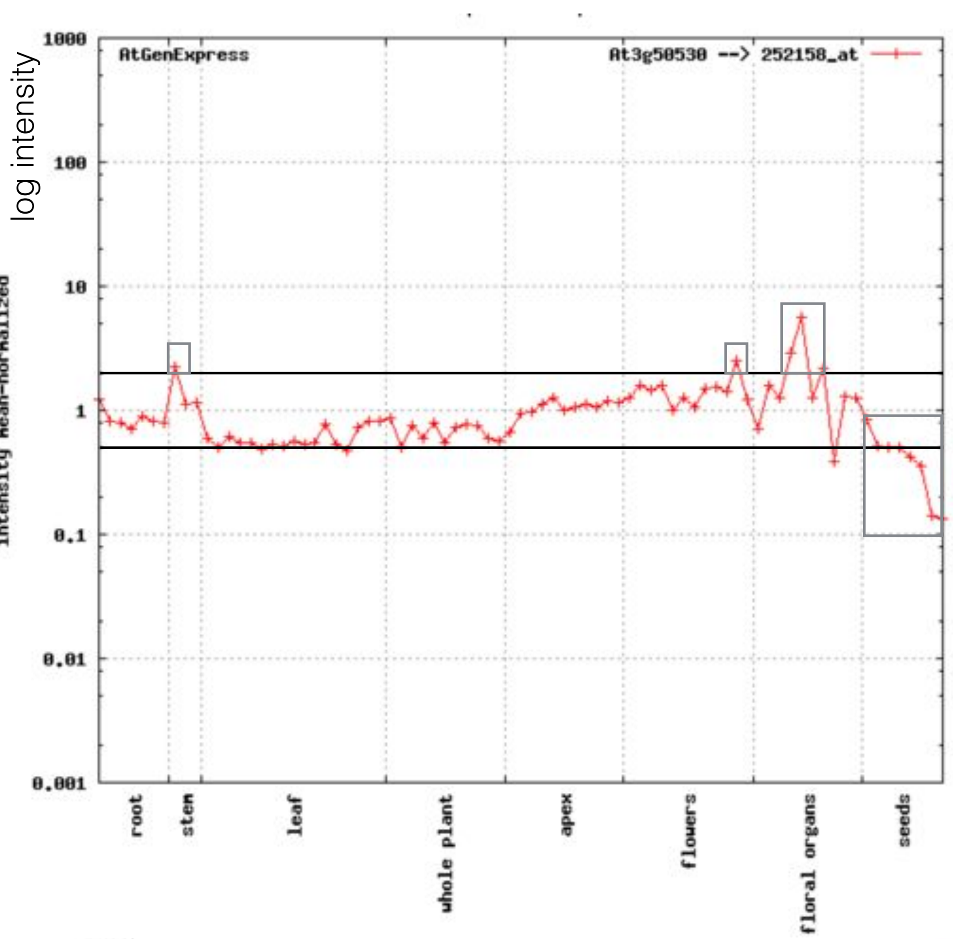
log intensity
intensity mean-normalized
AtGenExpress
At3g50530 --> 252158_at
1000
100
10
1
0.1
0.01
0.001
root
stem
leaf
whole plant
apex
flowers
floral organs
seeds

113

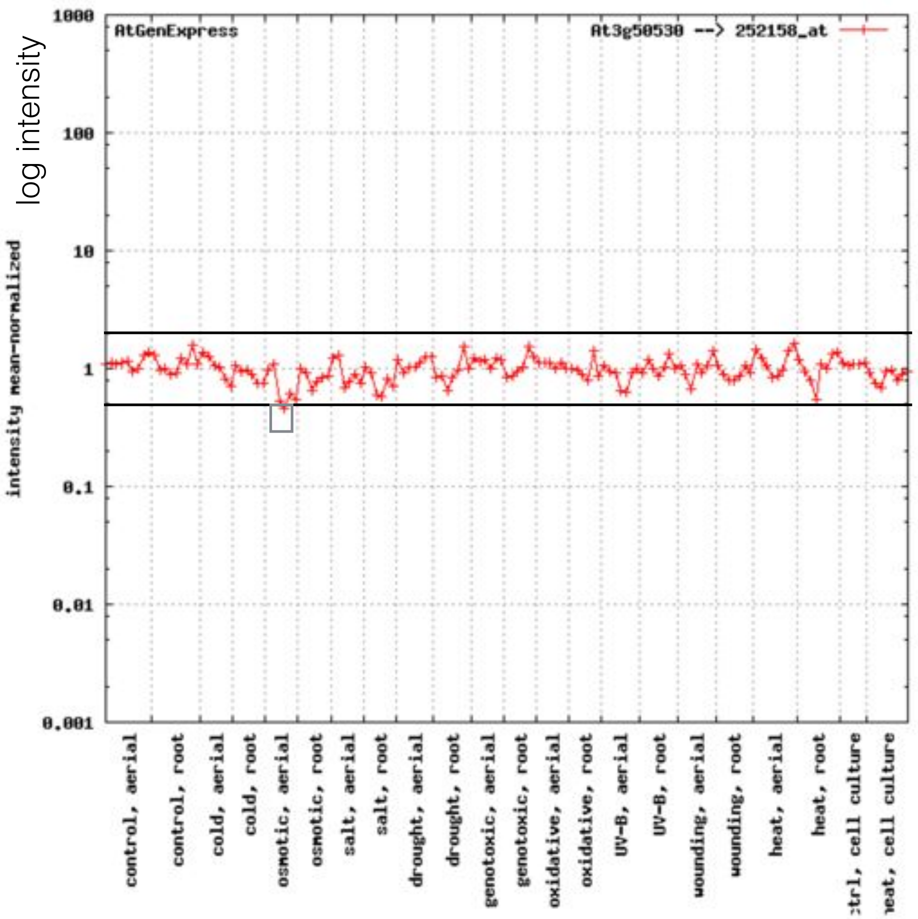
log intensity
intensity mean-normalized
AtGenExpress
At3g50530 --> 252158_at
1000
100
10
1
0.1
0.01
0.001
control, aerial
control, root
cold, aerial
cold, root
osmotic, aerial
osmotic, root
salt, aerial
salt, root
drought, aerial
drought, root
genotoxic, aerial
genotoxic, root
oxidative, aerial
oxidative, root
UV-B, aerial
UV-B, root
wounding, aerial
wounding, root
heat, aerial
heat, root
ctrl, cell culture
heat, cell culture

114

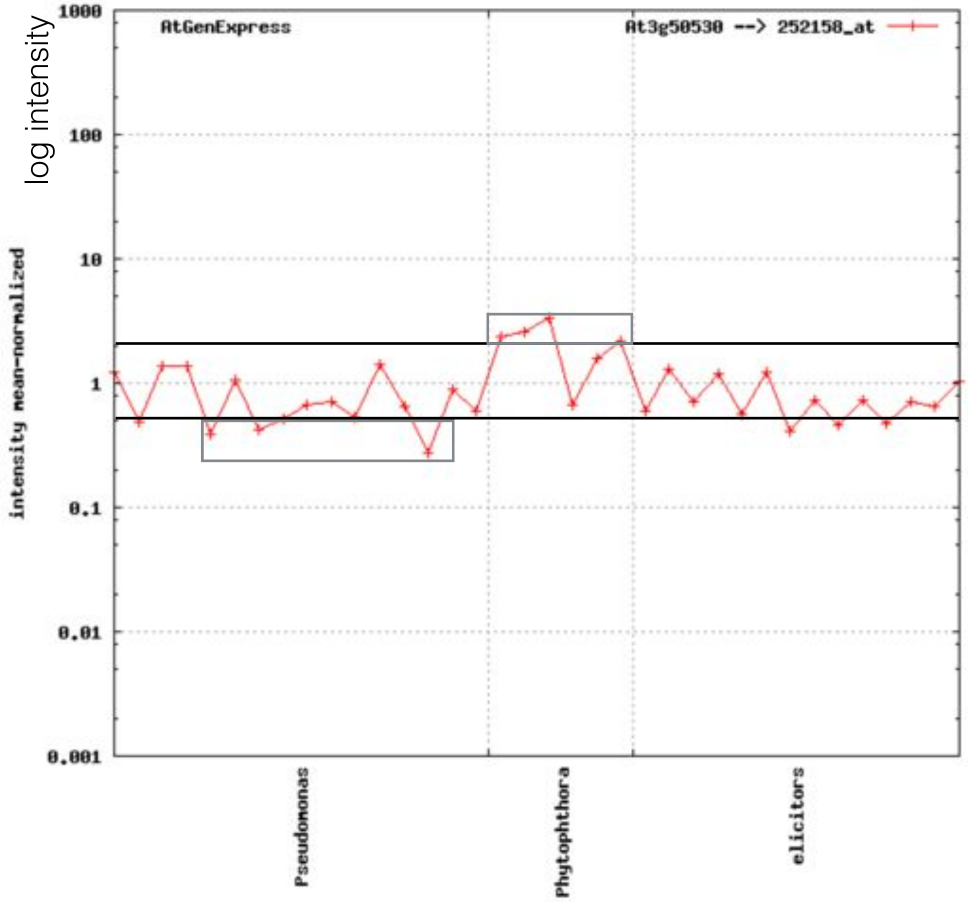
log intensity
intensity mean-normalized
AtGenExpress
At3g50530 --> 252158_at
1000
100
10
1
0.1
0.01
0.001
Pseudomonas
Phytophthora
elicitors

115

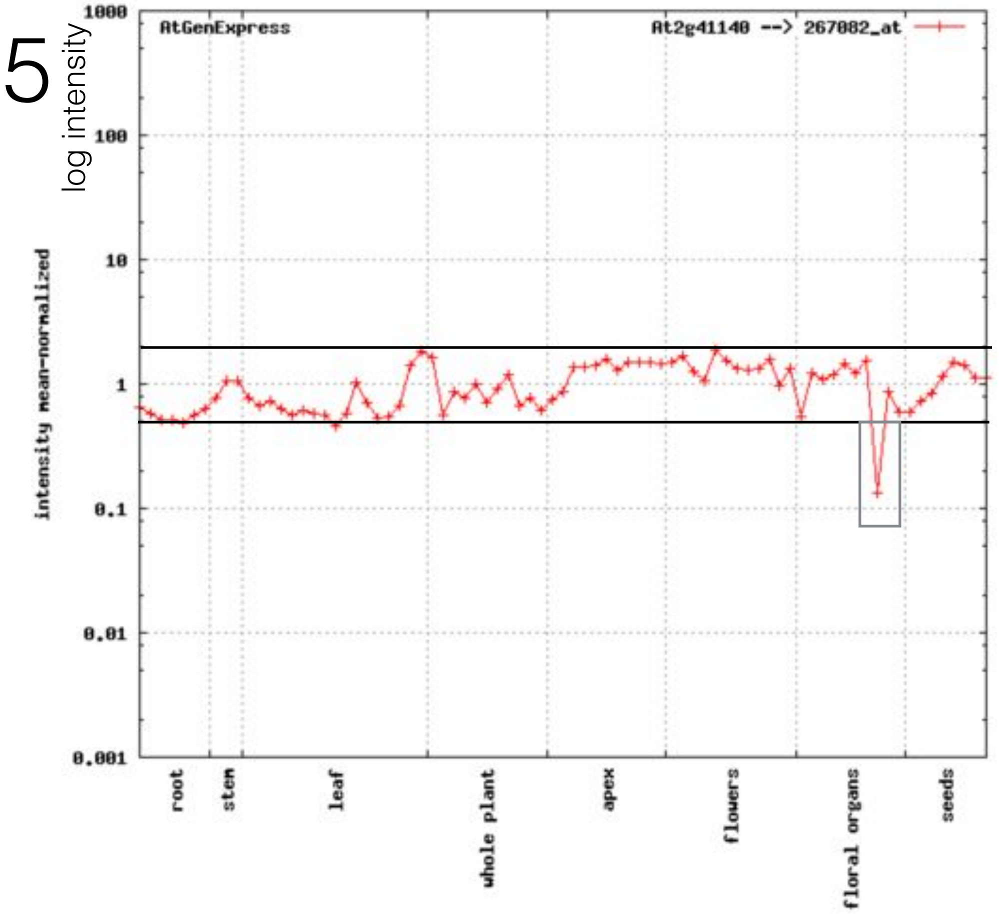
log intensity
intensity mean-normalized
AtGenExpress
At2g41140 --> 267082_at
1000
100
10
1
0.1
0.01
0.001
root
stem
leaf
whole plant
apex
flowers
floral organs
seeds

116

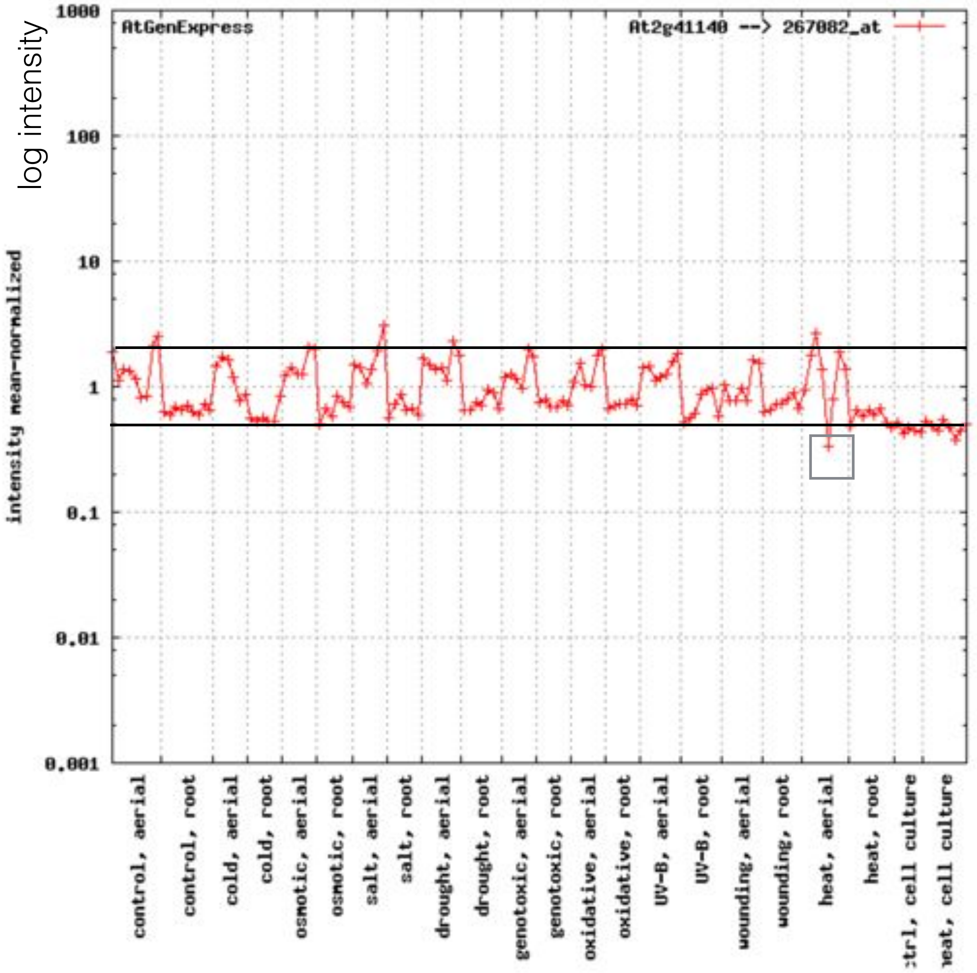
log intensity
intensity mean-normalized
AtGenExpress
At2g41140 --> 267082_at
1000
100
10
1
0.1
0.01
0.001
control, aerial
control, root
cold, aerial
cold, root
osmotic, aerial
osmotic, root
salt, aerial
salt, root
drought, aerial
drought, root
genotoxic, aerial
genotoxic, root
oxidative, aerial
oxidative, root
UV-B, aerial
UV-B, root
wounding, aerial
wounding, root
heat, aerial
heat, root

117

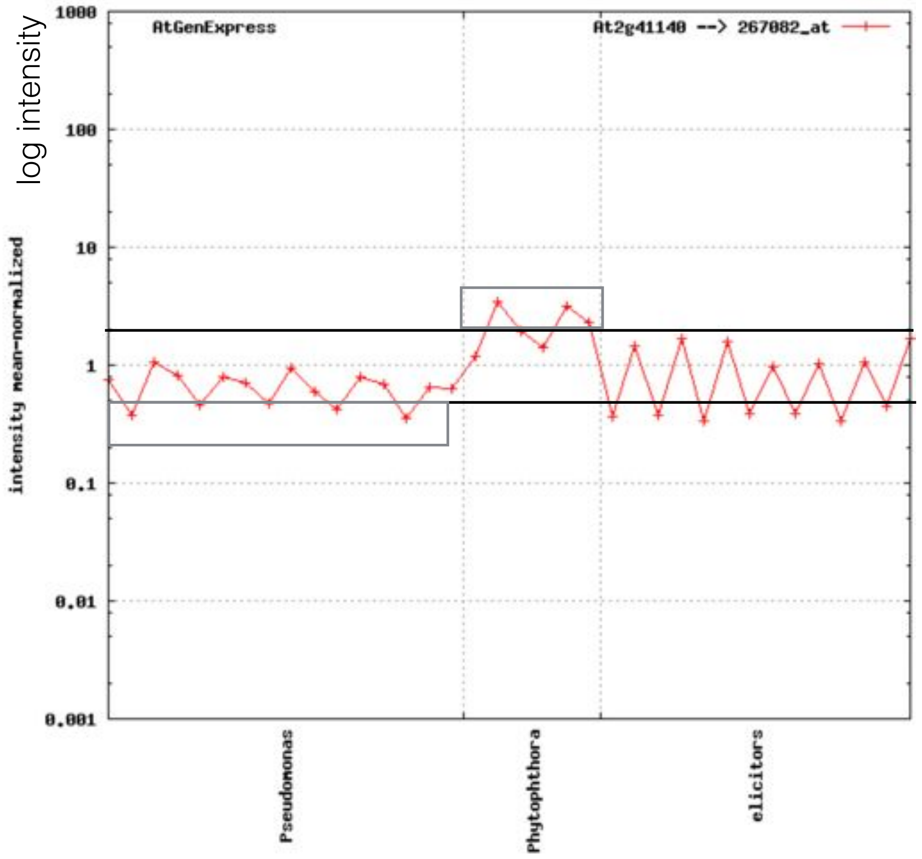
log intensity
intensity mean-normalized
AtGenExpress
At2g41140 --> 267082_at
1000
100
10
1
0.1
0.01
0.001
Pseudomonas
Phytophthora
elicitors

118

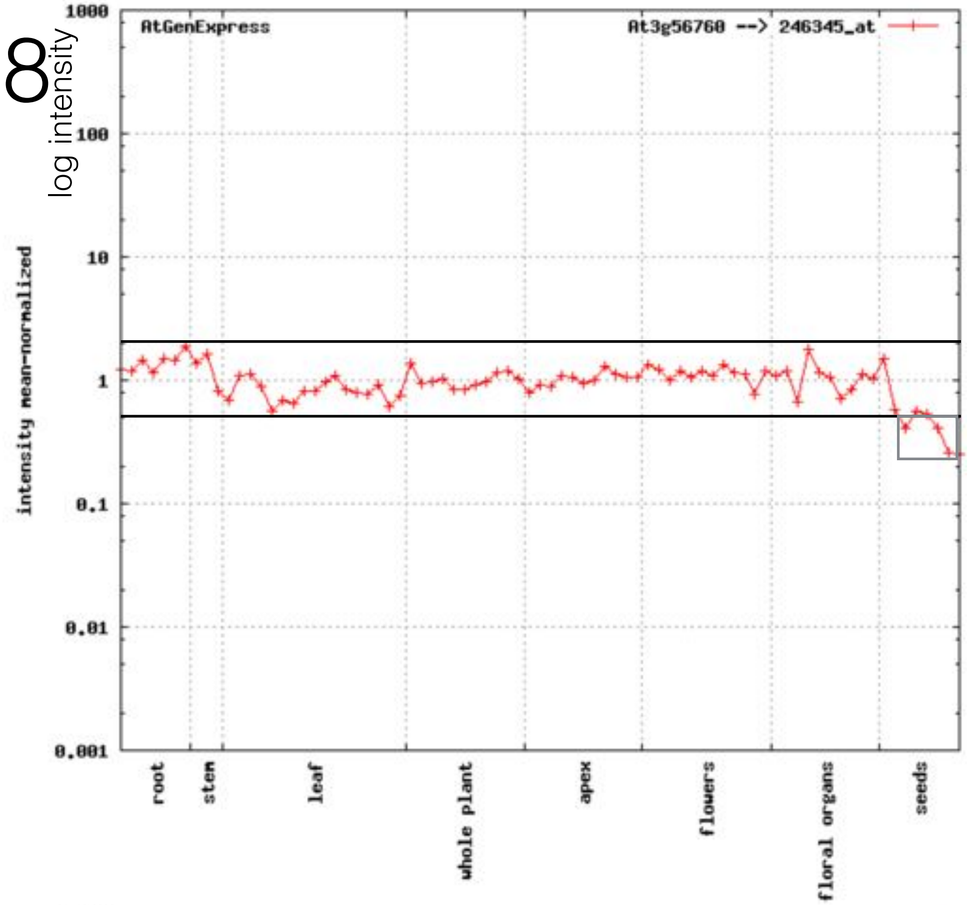
log intensity
AtGenExpress
At3g56760 --> 246345_at
intensity mean-normalized
1000
100
10
1
0.1
0.01
0.001
root
stem
leaf
whole plant
apex
flowers
floral organs
seeds

119

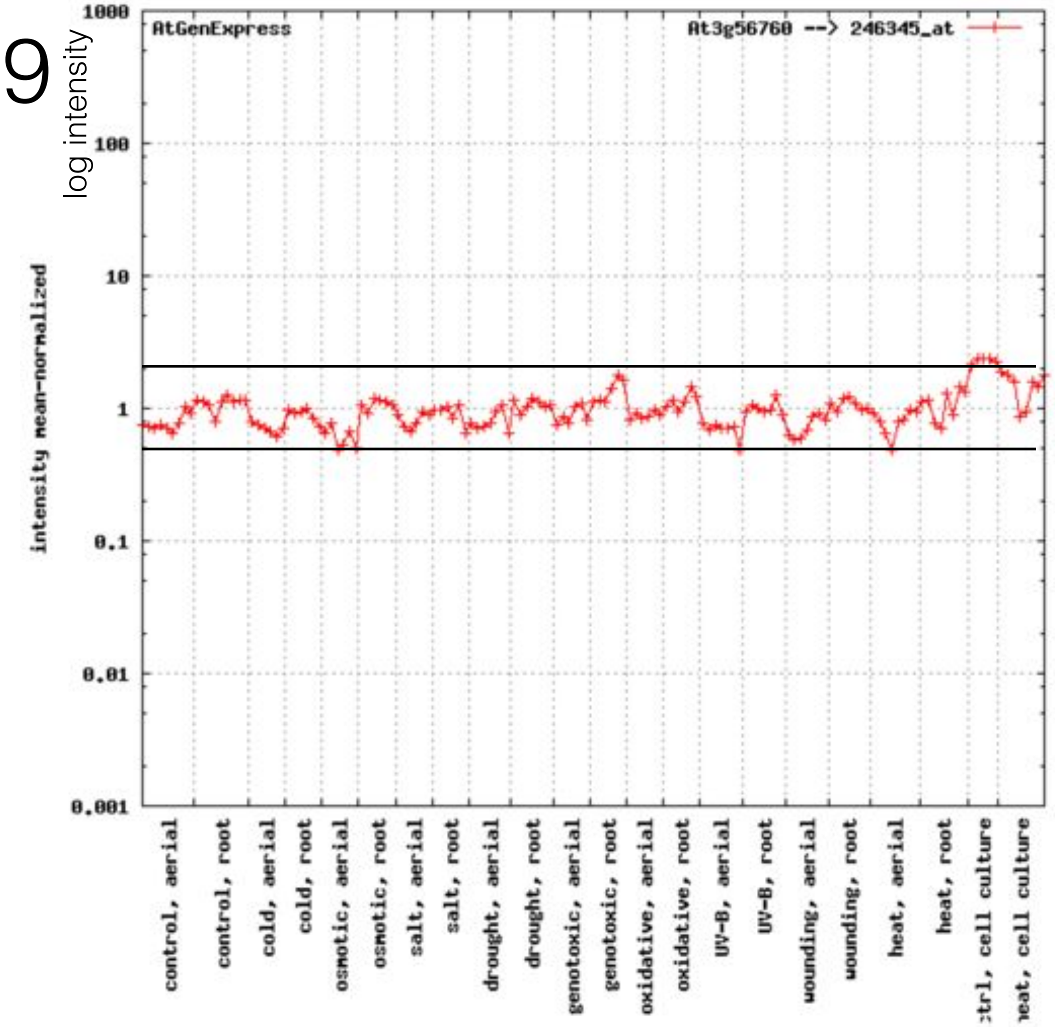
log intensity
AtGenExpress
At3g56760 --> 246345_at
intensity mean-normalized
1000
100
10
1
0.1
0.01
0.001
control, aerial
control, root
cold, aerial
cold, root
osmotic, aerial
osmotic, root
salt, aerial
salt, root
drought, aerial
drought, root
genotoxic, aerial
genotoxic, root
oxidative, aerial
oxidative, root
UV-B, aerial
UV-B, root
wounding, aerial
wounding, root
heat, aerial
heat, root

120

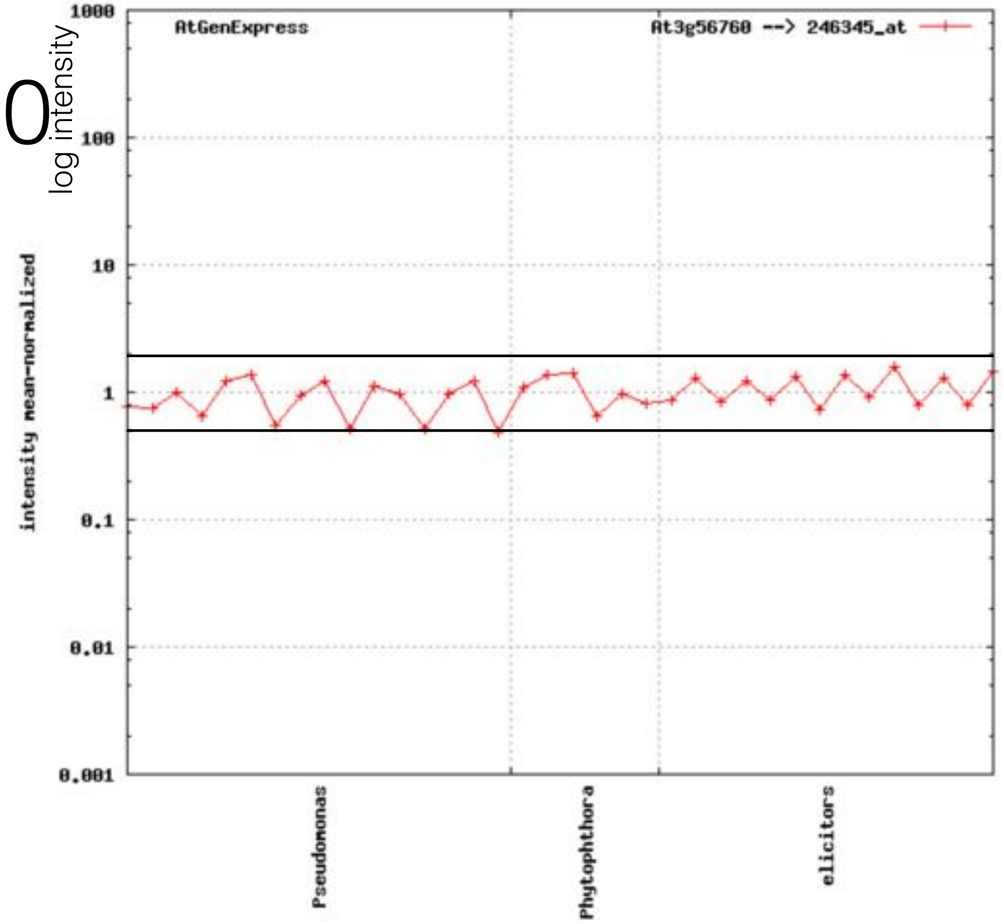
log intensity
AtGenExpress
At3g56760 --> 246345_at
intensity mean-normalized
1000
100
10
1
0.1
0.01
0.001
Pseudomonas
Phytophthora
elicitors

121

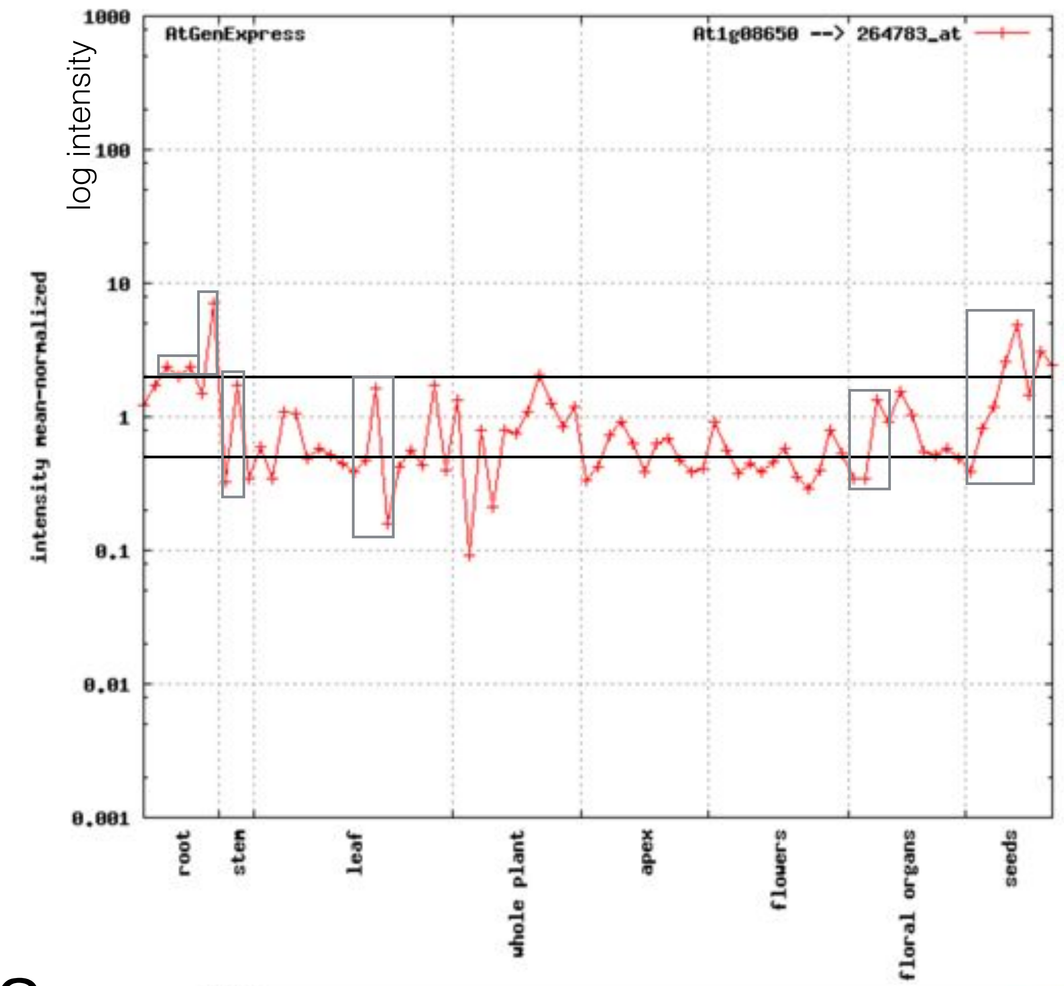
AtGenExpress
At1g08650 --> 264783_at
log intensity
intensity mean-normalized
1000
100
10
1
0.1
0.01
0.001
root
stem
leaf
whole plant
apex
flowers
floral organs
seeds

122

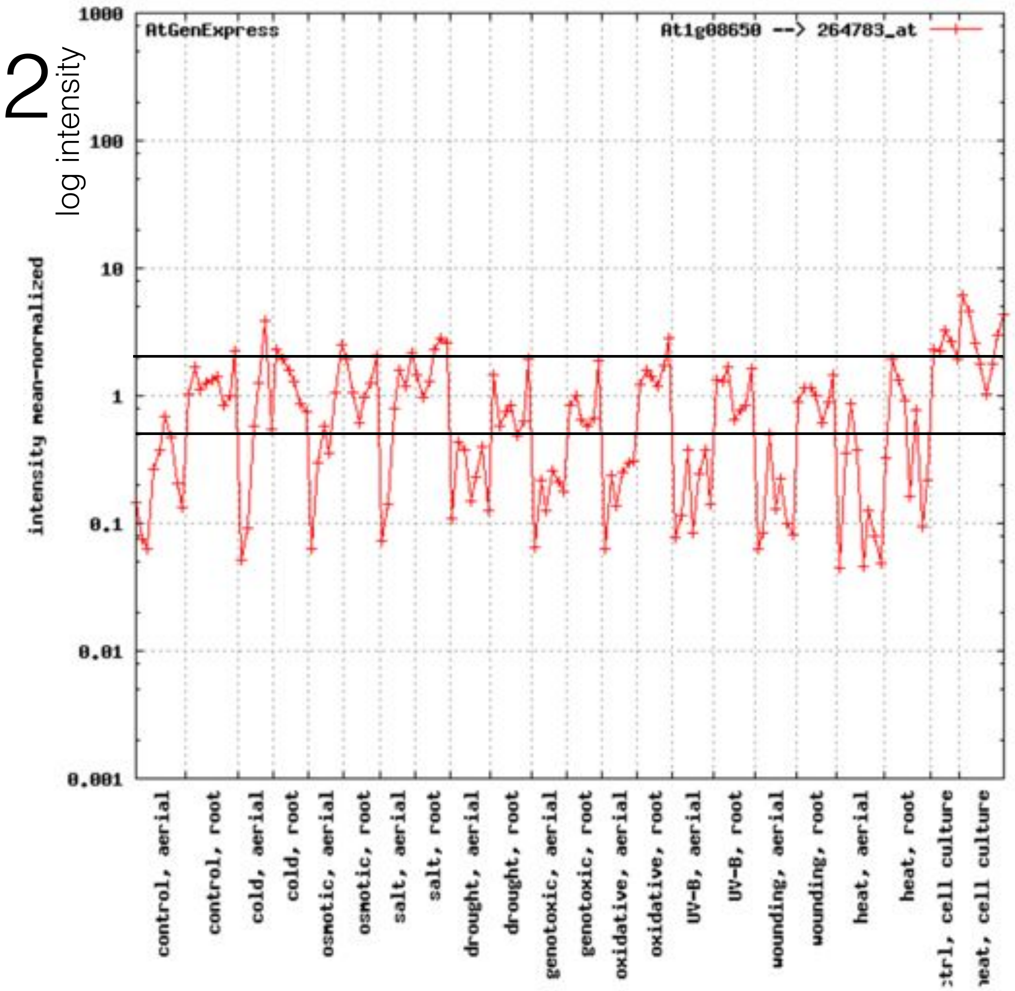
AtGenExpress
At1g08650 --> 264783_at
log intensity
intensity mean-normalized
1000
100
10
1
0.1
0.01
0.001
control, aerial
control, root
cold, aerial
cold, root
osmotic, aerial
osmotic, root
salt, aerial
salt, root
drought, aerial
drought, root
genotoxic, aerial
genotoxic, root
oxidative, aerial
oxidative, root
UV-B, aerial
UV-B, root
wounding, aerial
wounding, root
heat, aerial
heat, root
ctrl, cell culture
heat, cell culture

123

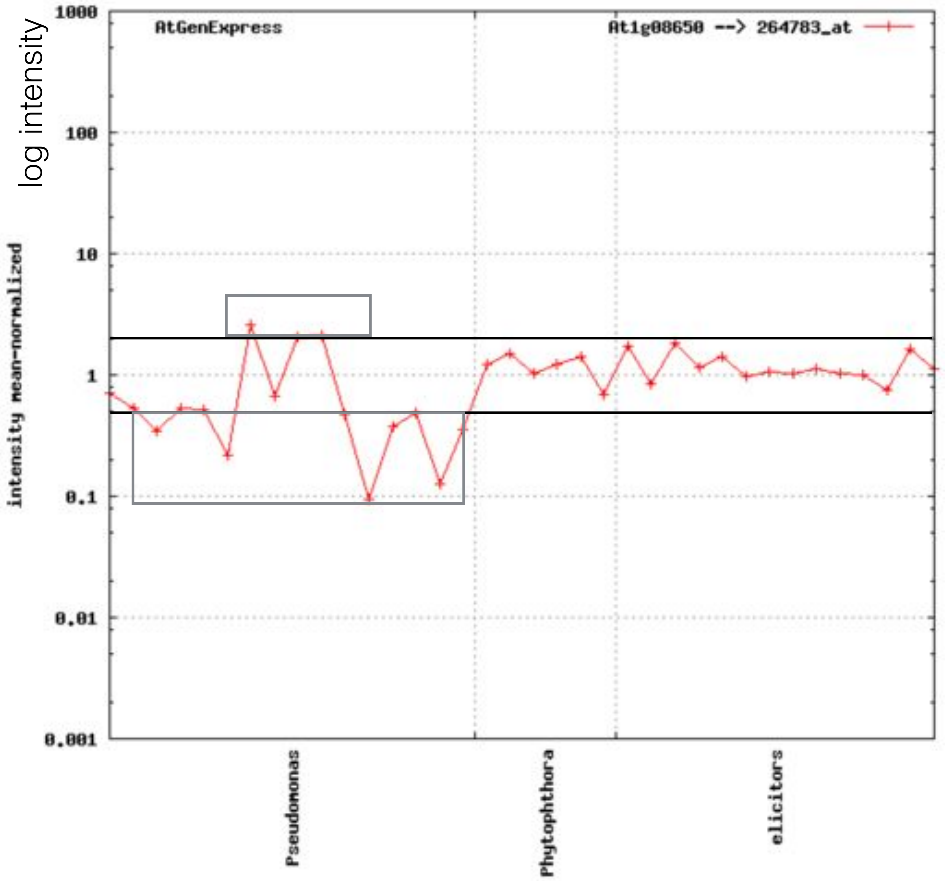
AtGenExpress
At1g08650 --> 264783_at
log intensity
intensity mean-normalized
1000
100
10
1
0.1
0.01
0.001
Pseudomonas
Phytophthora
elicitors

124

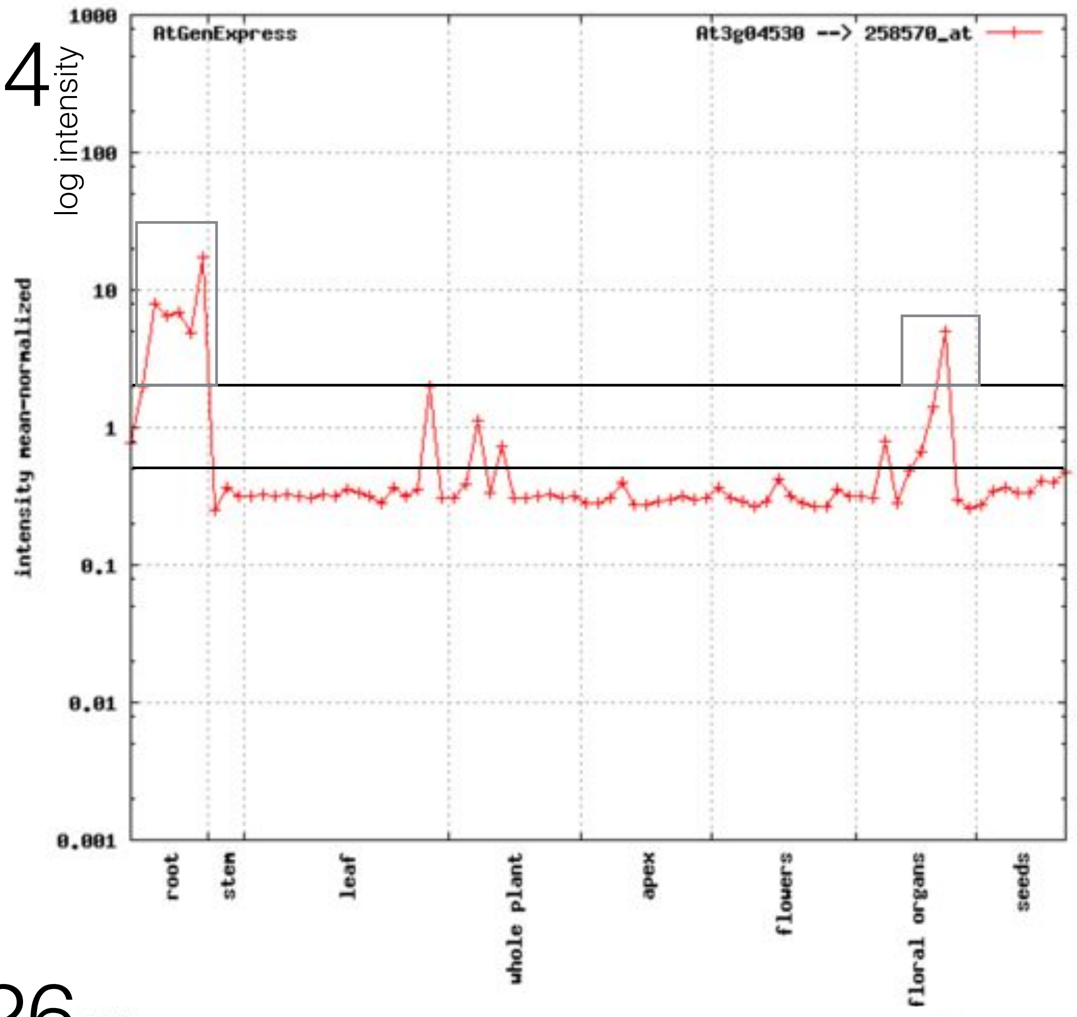
log intensity
intensity mean-normalized
AtGenExpress
At3g04530 --> 258570_at
1000
100
10
1
0.1
0.01
0.001
root
stem
leaf
whole plant
apex
flowers
floral organs
seeds

125

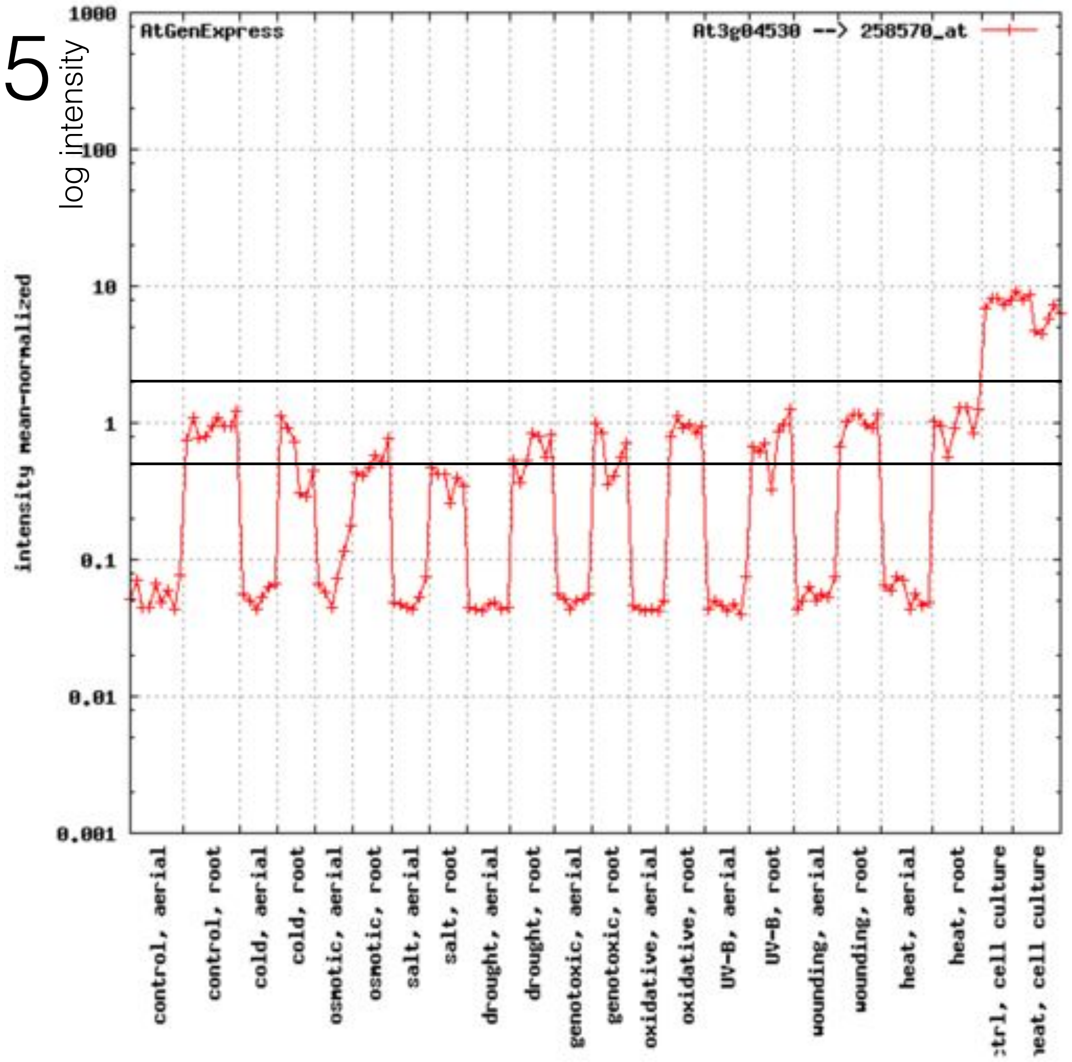
log intensity
intensity mean-normalized
AtGenExpress
At3g04530 --> 258570_at
1000
100
10
1
0.1
0.01
0.001
control, aerial
control, root
cold, aerial
cold, root
osmotic, aerial
osmotic, root
salt, aerial
salt, root
drought, aerial
drought, root
genotoxic, aerial
genotoxic, root
oxidative, aerial
oxidative, root
UV-B, aerial
UV-B, root
wounding, aerial
wounding, root
heat, aerial
heat, root

126

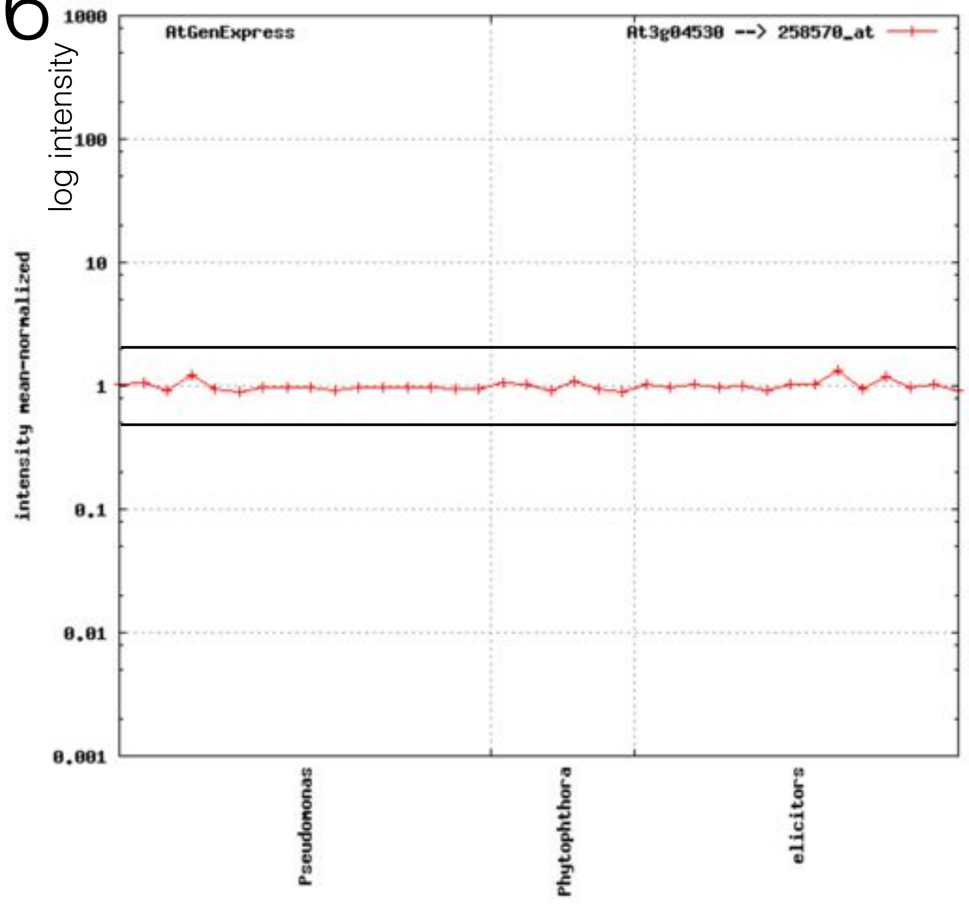
log intensity
intensity mean-normalized
AtGenExpress
At3g04530 --> 258570_at
1000
100
10
1
0.1
0.01
0.001
Pseudomonas
Phytophthora
elicitors

127

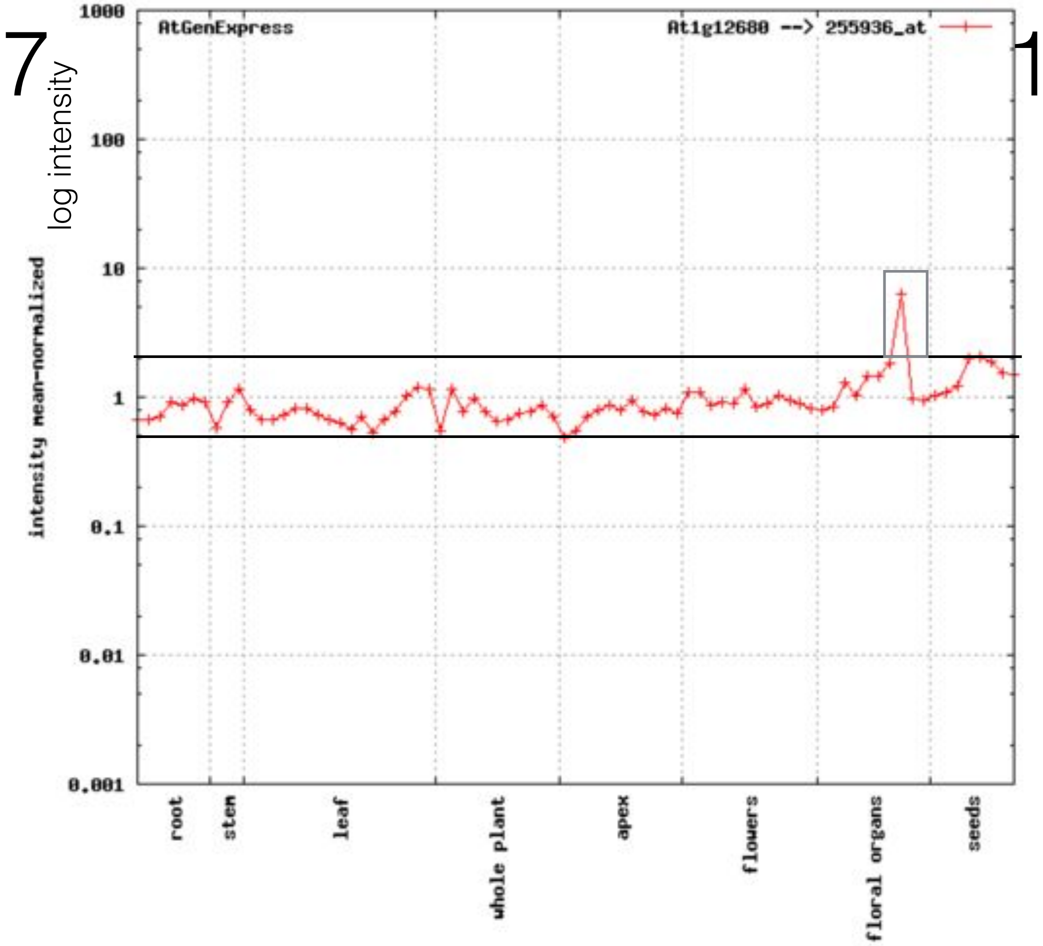
AtGenExpress
At1g12680 --> 255936_at
log intensity
intensity mean-normalized
1000
100
10
1
0.1
0.01
0.001
root
stem
leaf
whole plant
apex
flowers
floral organs
seeds

128

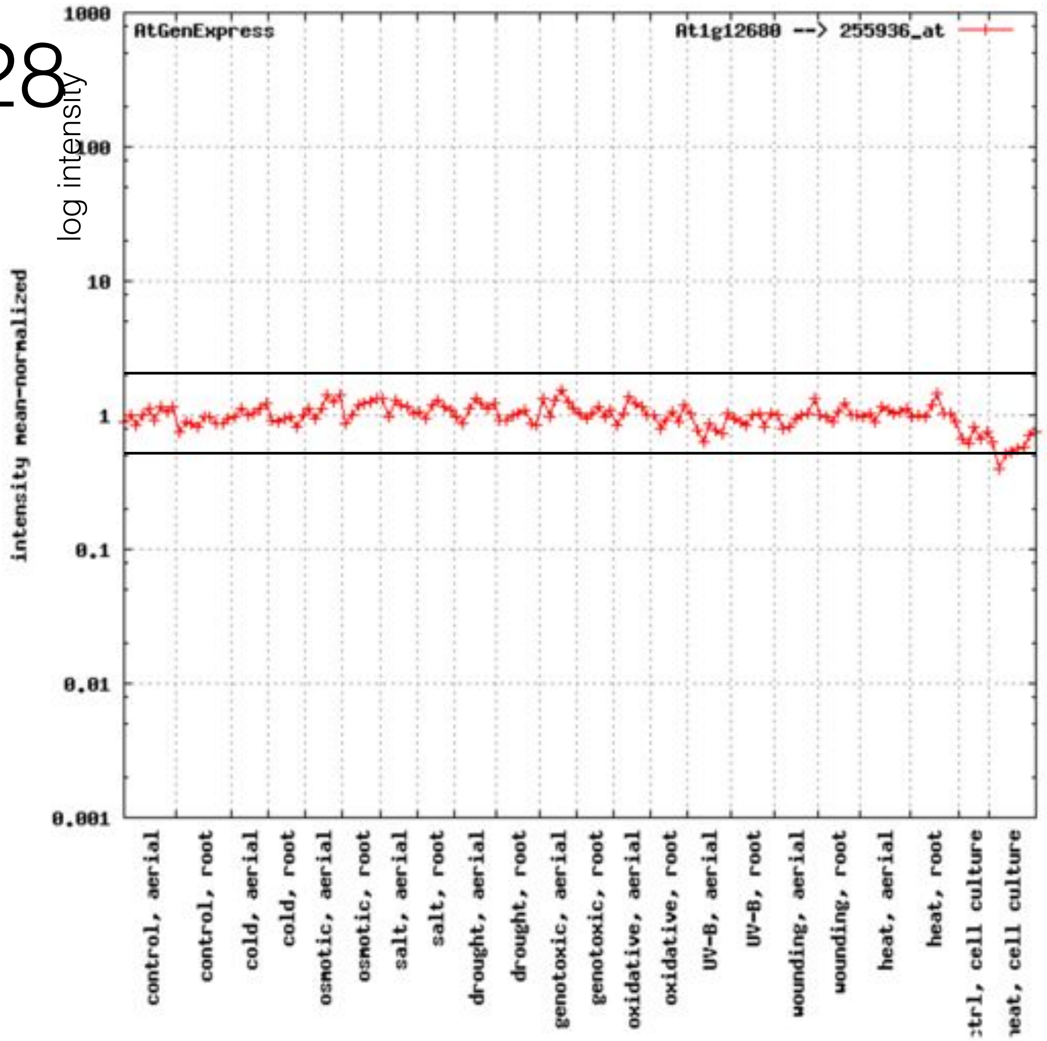
AtGenExpress
At1g12680 --> 255936_at
log intensity
intensity mean-normalized
1000
100
10
1
0.1
0.01
0.001
control, aerial
control, root
cold, aerial
cold, root
osmotic, aerial
osmotic, root
salt, aerial
salt, root
drought, aerial
drought, root
genotoxic, aerial
genotoxic, root
oxidative, aerial
oxidative, root
UV-B, aerial
UV-B, root
wounding, aerial
wounding, root
heat, aerial
heat, root

129

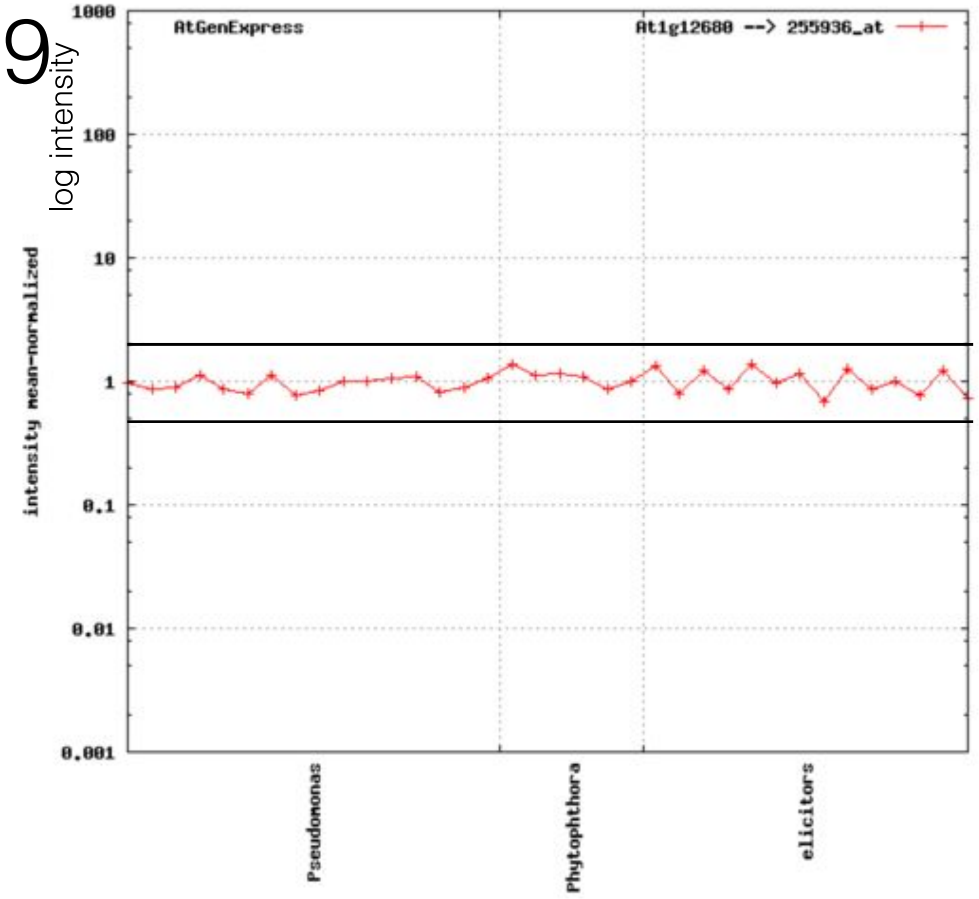
AtGenExpress
At1g12680 --> 255936_at
log intensity
intensity mean-normalized
1000
100
10
1
0.1
0.01
0.001
Pseudomonas
Phytophthora
elicitors

130

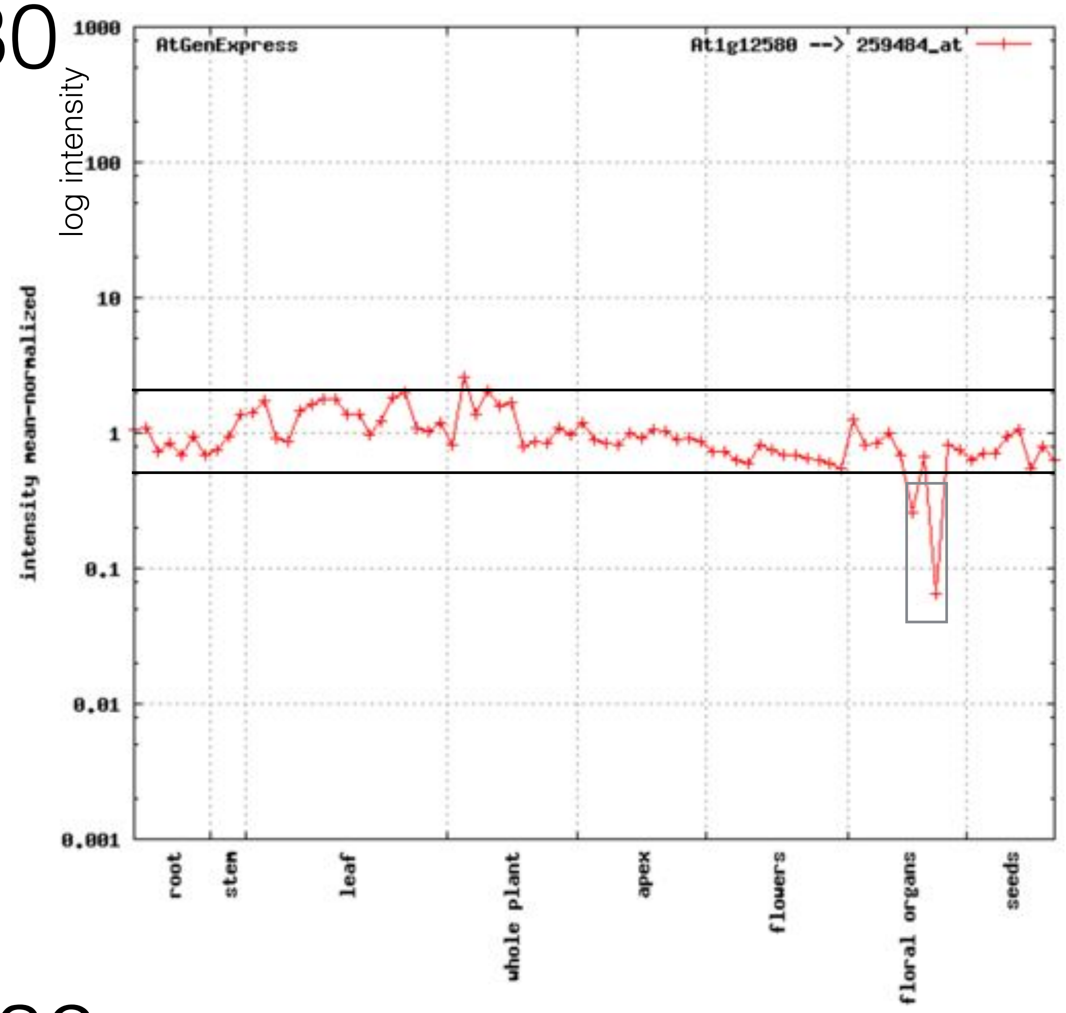
AtGenExpress
At1g12580 --> 259484_at
log intensity
intensity mean-normalized
1000
100
10
1
0.1
0.01
0.001
root
stem
leaf
whole plant
apex
flowers
floral organs
seeds

131

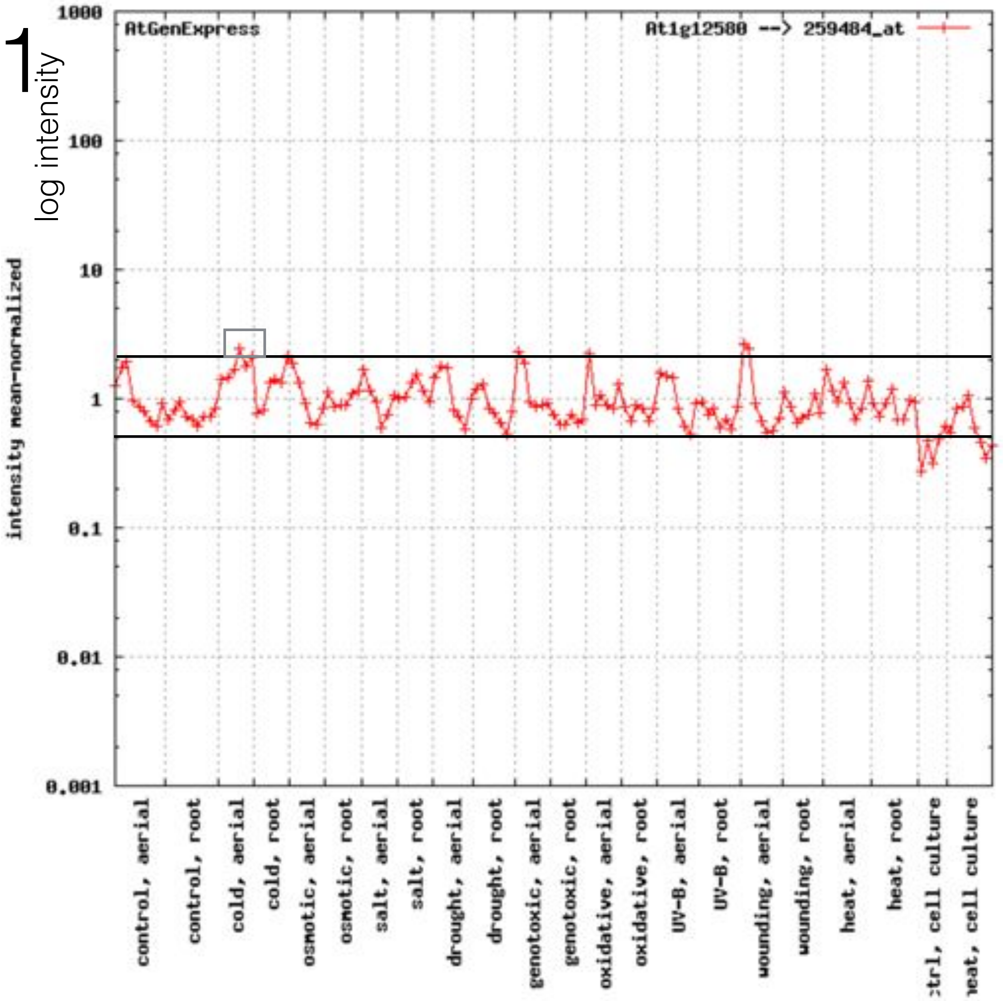
AtGenExpress
At1g12580 --> 259484_at
log intensity
intensity mean-normalized
1000
100
10
1
0.1
0.01
0.001
control, aerial
control, root
cold, aerial
cold, root
osmotic, aerial
osmotic, root
salt, aerial
salt, root
drought, aerial
drought, root
genotoxic, aerial
genotoxic, root
oxidative, aerial
oxidative, root
UV-B, aerial
UV-B, root
wounding, aerial
wounding, root
heat, aerial
heat, root

132

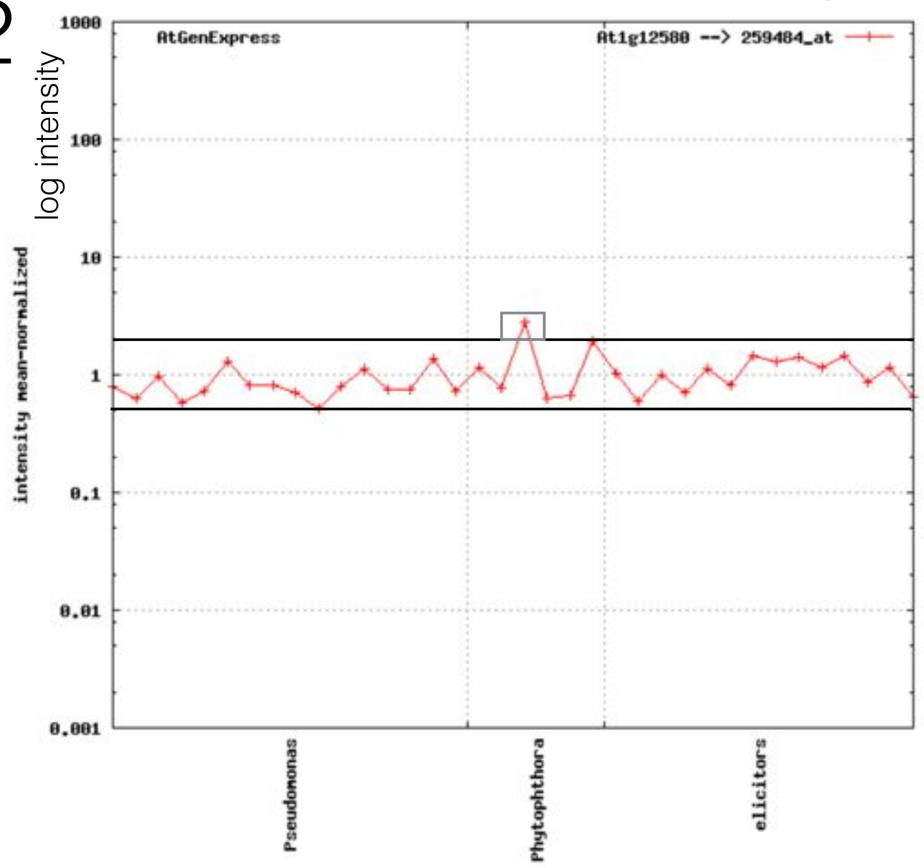
AtGenExpress
At1g12580 --> 259484_at
log intensity
intensity mean-normalized
1000
100
10
1
0.1
0.01
0.001
Pseudomonas
Phytophthora
elicitors

133

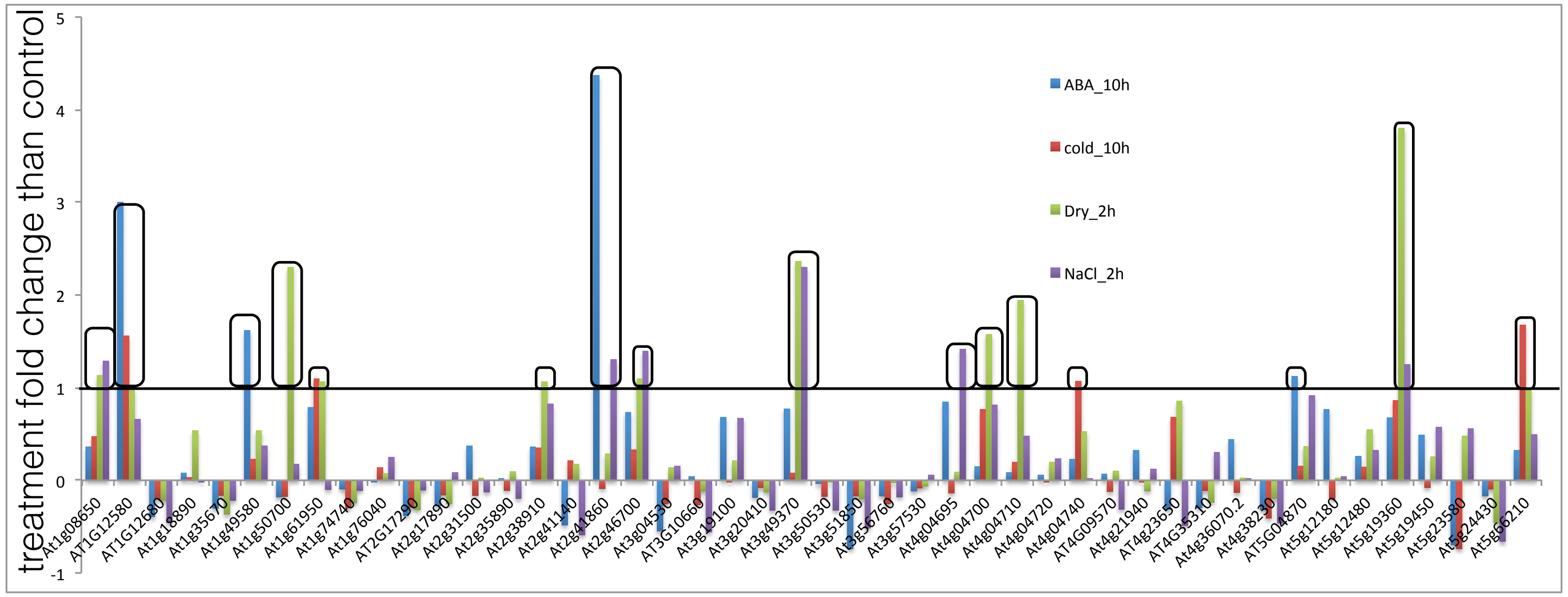
treatment fold change than control
ABA_10h
cold_10h
Dry_2h
NaCl_2h

134

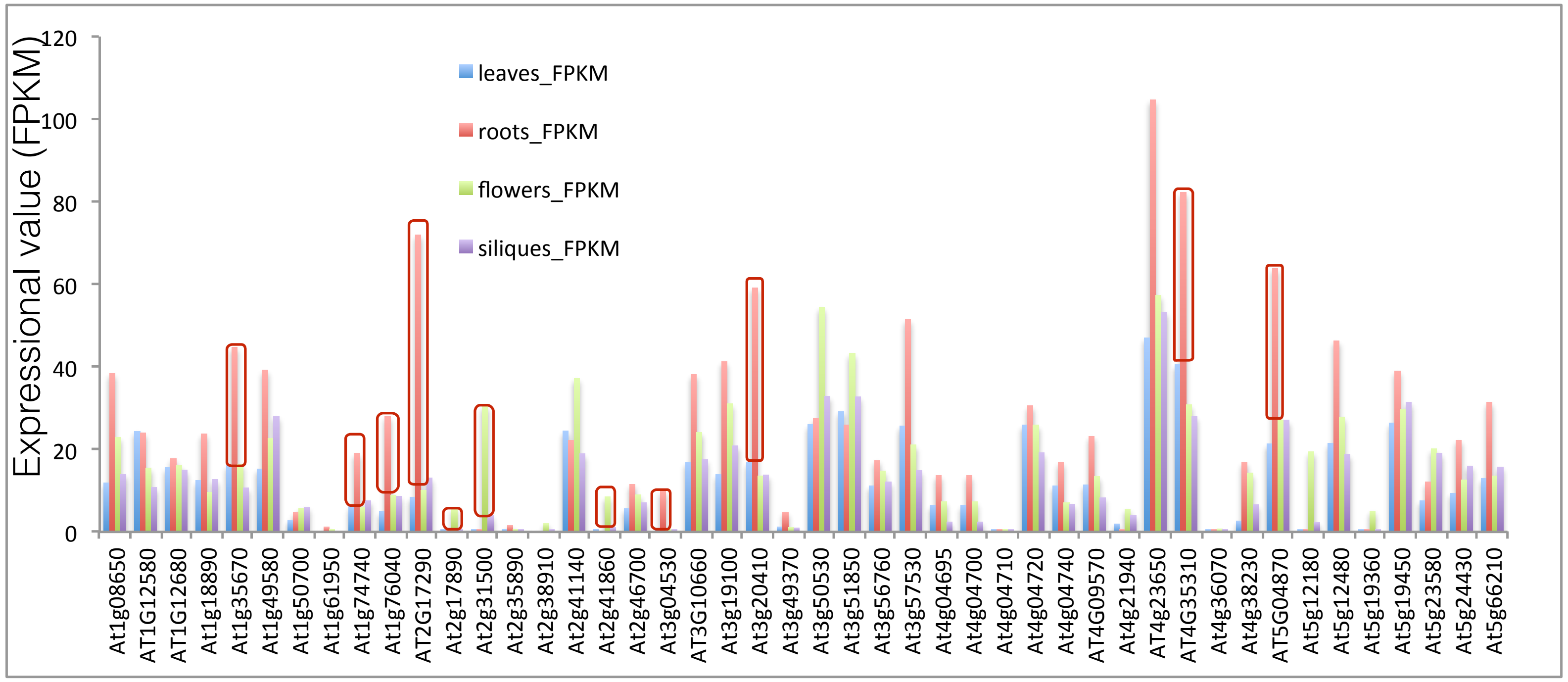
Expressional value (FPKM)
leaves_FPKM
roots_FPKM
flowers_FPKM
siliques_FPKM

135

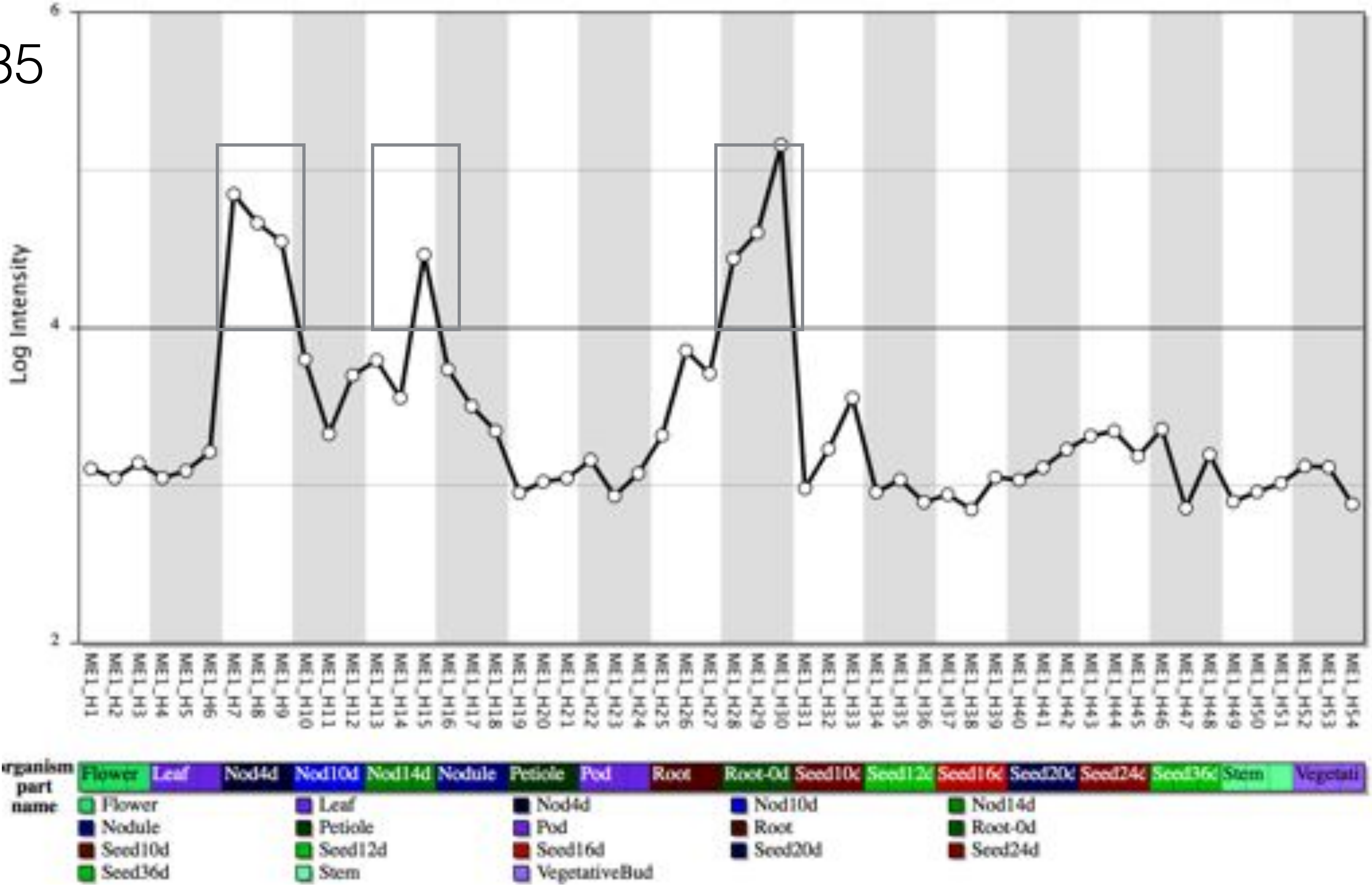

Log Intensity
6
4
2
ME1_H1
ME1_H2
ME1_H3
ME1_H4
ME1_H5
ME1_H6
ME1_H7
ME1_H8
ME1_H9
ME1_H10
ME1_H11
ME1_H12
ME1_H13
ME1_H14
ME1_H15
ME1_H16
ME1_H17
ME1_H18
ME1_H19
ME1_H20
ME1_H21
ME1_H22
ME1_H23
ME1_H24
ME1_H25
ME1_H26
ME1_H27
ME1_H28
ME1_H29
ME1_H30
ME1_H31
ME1_H32
ME1_H33
ME1_H34
ME1_H35
ME1_H36
ME1_H37
ME1_H38
ME1_H39
ME1_H40
ME1_H41
ME1_H42
ME1_H43
ME1_H44
ME1_H45
ME1_H46
ME1_H47
ME1_H48
ME1_H49
ME1_H50
ME1_H51
ME1_H52
ME1_H53
ME1_H54
organism part name
Flower
Leaf
Nod4d
Nod10d
Nod14d
Nodule
Petiole
Pod
Root
Root-0d
Seed10d
Seed12d
Seed16d
Seed20d
Seed24d
Seed36d
Stem
VegetativeBud

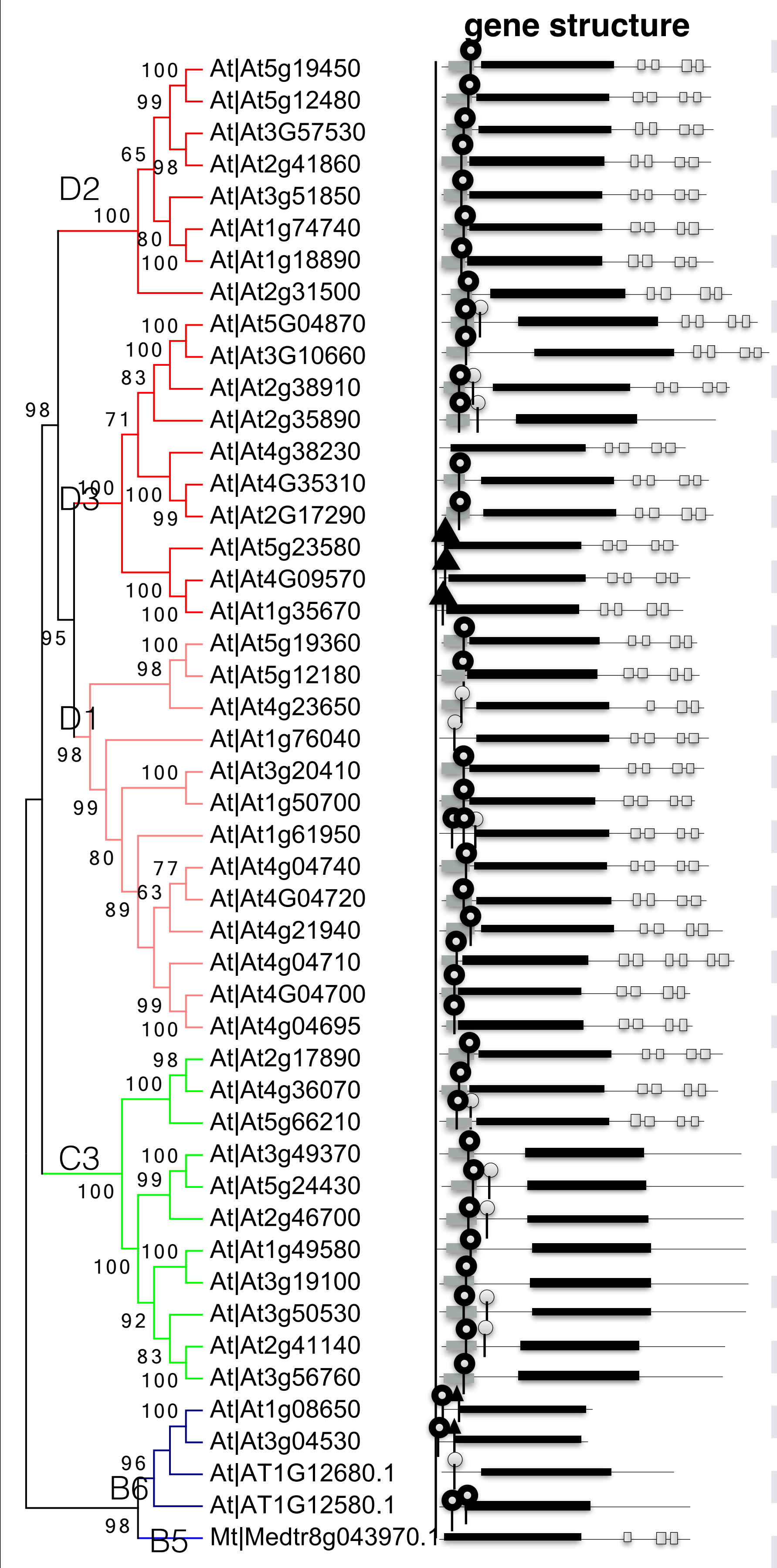

Gene_ID	Subcellular_Localization	development	Abiotic and biotic Stress	Plant-microbe communication
At5g19450	PM*	stomata*, mature pollen(Fig.S7_1)	salt(Fig. S7_2)	None(Fig.S7_3)
At5g12480	PM*	all tissues(Fig.S7_4), stomata*	None(Fig.S7_5)	pathogen(Fig. S7_6)
At3g57530	Membrane*,nucleus*	pollen, seed maturation (Fig. S7_7)	ABA*, stomata*, salt(Fig. S7_8)	pathogen(Fig.S7_9)
At2g41860	chlo: 5, cyto: 2, extr: 2, vacu: 2, plas: 1, E.R.: 1	pollen, seed maturation(Fig. S7_10)	ABA,NaCl (Fig. S7_133),none(Fig.S4_11)	None(Fig.S7_12)
At3g51850	Membrane*,PM*	pollen, seed maturation(Fig.S7_13)	Herbivore*,none(Fig.S7_14)	None(Fig.S7_15)
At1g74740	membrane*	whole plant except apex(Fig. S7_16)	ABA*, abiotic stress*,cold(Fig.S7_17)	pathogen(Fig. S7_18)
At1g18890	PM*	whole plant(Fig.S7_19)	ABA*, drought*, stomata*,heat(Fig.S7_20)	pathogen(Fig. S7_21)
At2g31500	chlo: 4, mito: 4, cyto: 3, plas: 2	flowers,seed maturation(Fig.S7_22)	None(Fig.S7_133,S7_23)	None(Fig.S7_24)
AT5G04870	Peroxisomes*,oil bodies*	pollen(Fig.S7_25)	SA&defense&cold&ABA(Fig.S7_26,133)&NaCl&oxidative stress&secondary metabolism*	pathogen(Fig.S7_27)
AT3G10660	ER and other membranes*	pollen(Fig.S7_28)	none(Fig.S7_29)	None(Fig.S7_30)
At2g38910	chlo: 4, cyto: 3, mito: 2.5, nucl: 2, pero: 2, mito_plas: 2	pollen(Fig.S7_31)	Drought(Fig.S7_133),none(Fig.S7_32)	None(Fig.S7_33)
At2g35890	membrane*	pollen(Fig.S7_34)	none(Fig.S7_35)	none(Fig.S7_36)
At4g38230	Peroxisomes,chlo,mito	pollen(Fig.S7_37)	UV-B(Fig.S7_38)	none(Fig.S7_39)
AT4G35310	Membrane*, cytosol*, nucleus*	pollen,seed maturation(Fig.S7_40)	MAMP*,UV-B(Fig.S7_41)	pathogen(Fig. S7_42)
AT2G17290	Membrane*, cytosol*, nucleus*	root,stem,leaf,apex,flowers,seed maturation (Fig. S7_43)	MAMP*,ABA*,drought*,salinity*,stomata*, heat(Fig.S7_44)	pathogen(Fig. S7_45)
At5g23580	Cytosol*, nucleus*	whole plant(Fig.S7_46)	ABA*,none(Fig.S7_47)	pathogen(Fig. S7_48)
AT4G09570	Cytosol*, nucleus*	flower,seed maturation(Fig.S7_49)	MAMP*,ABA*,drought*,salinity*,stomata*,salt,heat(Fig.S7_50)	pathogen(Fig. S7_51)
At1g35670	Cytosol*, nucleus*	whole plant(Fig.S7_52)	MAMP*,ABA*,drought*, salinity*,stomata*,cold(Fig.S7_53)	pathogen(Fig. S7_54)
At5g19360	PM*	pollen*, seed maturation(Fig.S7_55)	ABA*,cold*,NaCl*&Drought*(Fig.S5),none(Fig.S7_56)	none(Fig.S7_57)
At5g12180	PM*	pollen, seed maturation*(Fig.S7_58)	ABA*,Drought*, none(Fig.S7_59)	none(Fig.S7_60)
AT4G23650	Soluble*,PM*,tonoplast*	root,pollen,seed maturation(Fig.S7_61)	Herbivore*,salinity*,stomata*,none(Fig.S7_62)	none(Fig.S7_63)
At1g76040	cyto,nucl,chlo	root,leaf,stem,apex,flower(Fig.S7_64)	ABA*,Cold*,Drought*(Fig.S7_65)	pathogen(Fig S7_66)
At3g20410	chlo: 5, cyto_nucl: 4, nucl: 3.5, cyto: 3.5, mito	root,pollen(Fig.S7_67)	Drought*,NaCl*,heat(Fig.S7_68)	pathogen(Fig.S7_69)
At1g50700	cyto: 8.5, cyto_nucl: 6.5, nucl: 3.5	whole plant(Fig. S7_70)	none(Fig.S7_71),dry(Fig.S7_133)	none(Fig.S7_72)
At1g61950	membrane*	root,pollen,seed maturation(Fig.S7_73)	cold,dry(Fig.S7_133),none(Fig.S7_74)	none(Fig.S7_75)
At4g04740	PM*	leaf,flower(Fig.S7_76)	Drought*,salinity*,stomata*,cold(Fig.S7_77,S7_133)	pathogen(Fig.S7_78)
At4g04720	PM*	stomata*	Osmotic stress*	unknown
At4g21940	cyto: 5.5, cyto_nucl: 4, plas: 3, chlo: 2, nucl: 1.5, mito: 1	root,leaf,apex,flower(Fig.S7_79)	cold,osmotic stress,salt(Fig.S7_80)	pathogen(Fig.S7_81)
At4g04710	cyto: 6, E.R.: 3, nucl: 2, mito: 1, pero: 1	flower,seed maturation(Fig.S7_82)	dry(Fig.S7_133),none(FigS7_83)	none(Fig.S7_84)
At4g04700	cyto: 12, cysk: 1	pollen(Fig.S7_85)	none(Fig.S7_86),dry(Fig.S7_133)	pathogen(Fig.S7_87)
At4g04695	chlo: 5, cyto: 4, nucl: 2, mito: 2	unknown	NaCl(Fig.S5)	unknown
At2g17890	PM*	pollen, seed(Fig.S7_88)	none(Fig.S7_89)	none(Fig.S7_90)
At4g36070	cyto: 6, nucl: 4, mito: 3	pollen(Fig.S7_91)	none(Fig.S7_92)	none(Fig.S7_93)
At5g66210	PM*	root,leaf,apex,pollen,seed maturation(Fig.S7_94)	salt,drought,wounding(Fig.S4_95),cold(Fig.S7_133)	pathogen(Fig. S7_96)
At3g49370	chlo: 4, nucl: 4, cyto: 4, mito: 1	root,flower,seed maturation(Fig.S7_97)	salt(Fig.S7_98,S7_133), dry(Fig.S7_133)	pathogen(Fig. S7_99)
At5g24430	chlo: 6, nucl: 5, cyto: 1, mito: 1	pollen(Fig.S7_100)	cold,heat(Fig.S7_101)	pathogen(Fig. S7_102)
At2g46700	cyto: 5, E.R.: 5, chlo: 1, nucl: 1, mito: 1	whole plant(Fig. S7_103)	none(Fig.S7_104),dry&NaCl(Fig.S7_133)	none(Fig.S7_105)
At1g49580	chlo: 5, cyto: 4, nucl: 3, mito: 1	stem,leaf,apex,flower,seed maturation(Fig.S7_106)	heat(Fig.S7_107),ABA(Fig.S7_133)	pathogen(Fig. S7_108)
At3g19100	cyto: 7, nucl: 3, chlo: 2, cysk: 2	leaf,pollen(Fig.S7_109)	osmotic stress(Fig.S7_110)	pathogen(Fig. S7_111)
At3g50530	chlo: 7, nucl: 4, cyto: 1, mito: 1	stem, flower, seed maturation(Fig.S7_112)	osmotic stress(Fig.S7_113)	pathogen(Fig. S7_114)
At2g41140	cyto: 5, nucl: 4, mito: 2, chlo: 1, vacu: 1	pollen(Fig.S7_115)	heat(Fig.S7_116)	pathogen(Fig. S7_117)
At3g56760	cyto: 7, nucl: 4, chlo: 1, mito: 1	seed maturation(Fig.S7_118)	none(Fig.S7_119)	none(Fig.S7_120)
At1g08650	cyto: 5, nucl: 4, chlo: 2, mito: 2	whole plant(Fig.S7_121),C4&CAM photosynthesis#$	none(Fig.S7_122),dry&NaCl(Fig.S7_133)	pathogen(Fig. S7_123)
At3g04530	cyto: 8, chlo: 2, nucl: 2, cysk: 2	root,pollen(Fig.S7_124),C4&CAM photosynthesis#$	none(Fig.S7_125)	none(Fig.S7_126)
At1g12680	nucl: 10, cyto: 2, chlo: 1	pollen(Fig.S7_127)	none(Fig.S7_128)	none(Fig.S7_129)
At1g12580	nucl: 10, cyto: 1, extr: 1, vacu: 1	pollen (Fig.S7_130)	cold(Fig.S7_131,S7_133),ABA(Fig.S7_133)	pathogen(Fig. S7_132)
Medtr8g043970	nucl: 6, cyto: 6, chlo: 2	none(Fig.S7_135)	none(Fig.S7_135)	root symbiosis(*,Fig.S7_135)

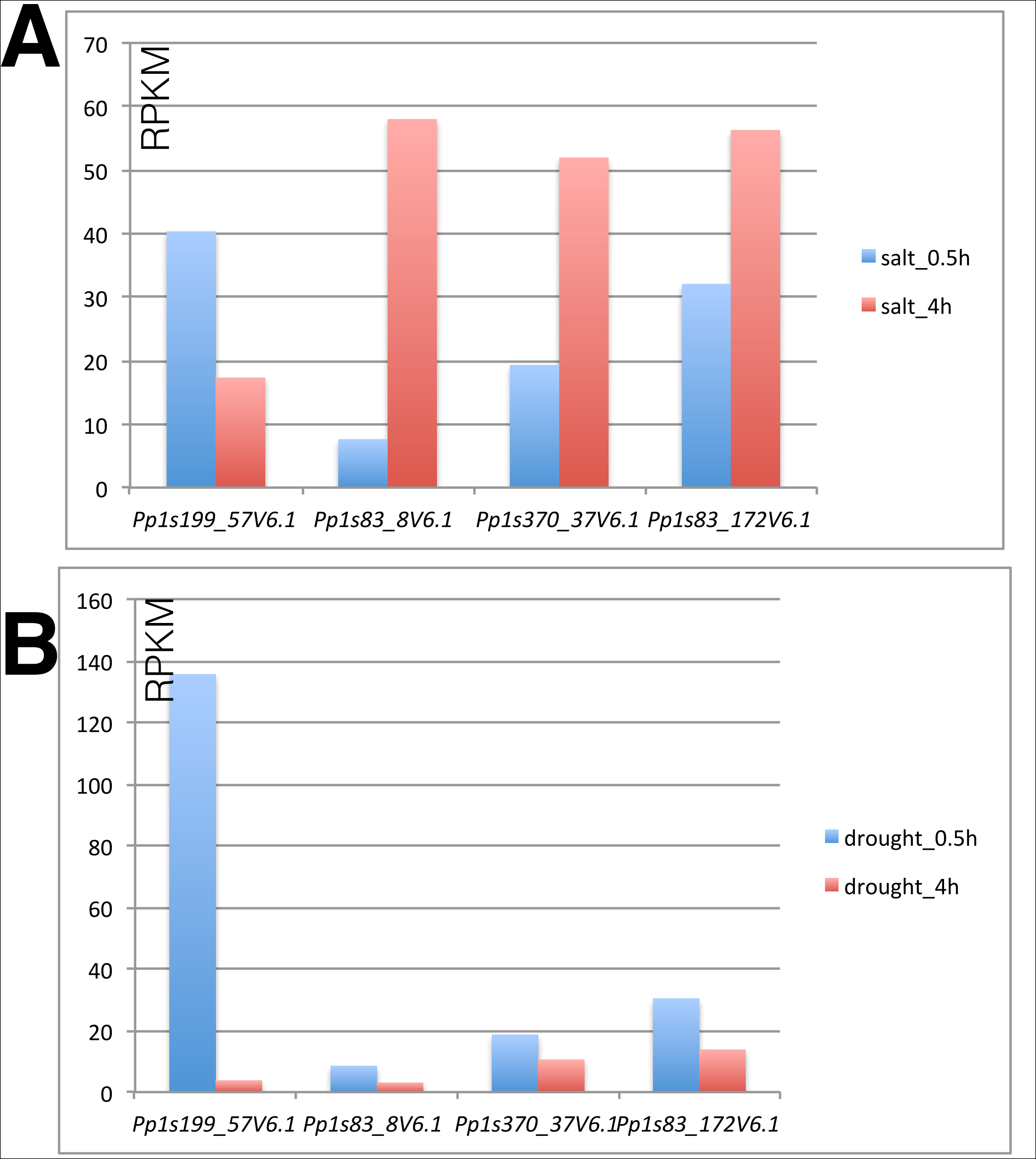
A
RPKM
70
60
50
40
30
20
10
0
salt_0.5h
salt_4h
Pp1s199_57V6.1
Pp1s83_8V6.1
Pp1s370_37V6.1
Pp1s83_172V6.1
B
RPKM
160
140
120
100
80
60
40
20
0
drought_0.5h
drought_4h
Pp1s199_57V6.1
Pp1s83_8V6.1
Pp1s370_37V6.1
Pp1s83_172V6.1

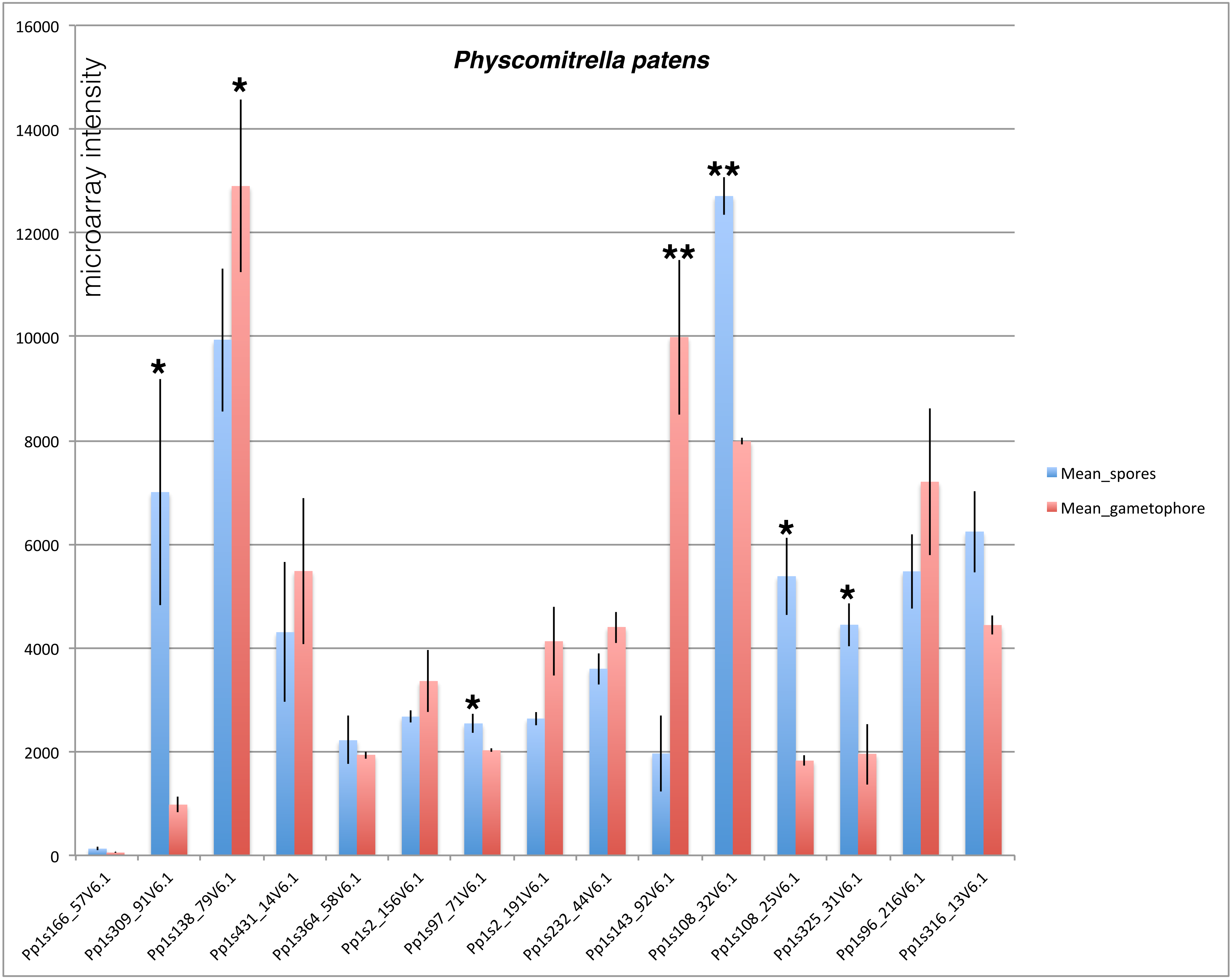
Physcomitrella patens
microarray intensity
16000
14000
12000
10000
8000
6000
4000
2000
0
Mean_spores
Mean_gametophore
Pp1s166_57V6.1
Pp1s309_91V6.1
Pp1s138_79V6.1
Pp1s431_14V6.1
Pp1s364_58V6.1
Pp1s2_156V6.1
Pp1s97_71V6.1
Pp1s2_191V6.1
Pp1s232_44V6.1
Pp1s143_92V6.1
Pp1s108_32V6.1
Pp1s108_25V6.1
Pp1s325_31V6.1
Pp1s96_216V6.1
Pp1s316_13V6.1

##########

This text file includes the Supplemental File S1, followed by the references to the supplemental figures.

##########

Supplemental File S1. Representative sequences that have innovative gene structure compared to the typical structure composed of a KD and a CaM-LD.

(1) degenerated kinase domain, distributed in red algae to flowering plants
Pcr|HS694783.1
Vca|Vocar20001731m
Olu|2798
Vitis_vinifera|VIT_00s0577g00010
(2) myristoylation at the Nterminal, distributed in Charophytes to flowering plants
Mpu|175307|126004
Cre|Cre13.g564500.t1.3
Kfl|kfl00234_0080
At|At2G17290
At|At2g41860
At|At5g12180
(3) palmytoylation at the NUterminal, distributed in seawater algae to flowering plants
Mpu|175307|126004
Cre|Cre13.g564500.t1.3
Kfl|kfl00036_0390
(4) C2 domain at the NUterminal, distributed only in freshwater algae
Cre|Cre17.g705000.t1.2
Vca|Vocar20010397m
(5) PEST motif in the NUterminal, distributed in freshwater algae to flowering plants

Cre|Cre02.g114750.t1.2
Kfl|kfl00013_0520
Atr|scaffold00018.149
Vv|VIT_16s0098g01140
(6) Acylation at the NUterminal, distributed in freshwater algae to flowering plants
Cre|Cre02.g114750.t1.2
Kfl|kfl00013_0520
Atr|scaffold00018.149
Vv|VIT_16s0098g01140
(7) DnaJ domain in the NUterminal, distributed in flowering plants
Br|Bra009697
Fv|mrna09567.1
(8) AAA domain in the NUterminal, distributed in flowering plants
Br|Bra001373
Br|Bra029859
(9) Two EF hands in the CUterminal, distributed in red algae to flowering plants
Pcr|HS694783.1
Cva|35323
Cre|Cre06.g265550.t1.2
Cva|20239
Atr|scaffold00073.66
(10) 4 EF hands at the CUterminal, the typical calmodulinUlike domain, distributed in seawater algae to flowering plants
Mpu|175307|126004
Vca|Vocar20000362m
At|At2G17290
At|At2g41860 (11) three EF hands at the CUterminal, distributed in seawater algae to flowering plants
Olu|2798
Csu|20203
Atr|scaffold00101.38
At|At4g23650 (12) one EF hands at the CUterminal, distributed in freshwater

algae to flowering plants
Csu|11155
Csu|2460
Ppa|Pp1s159_42V6.1
Sm|Sm173444|PACid:15414880

Fv|Fvmrna24077.1
Fv|Fvmrna18654.1
(13) Insertion in the AID, and degeneration of first EF hand, distributed in all streptophytes
Mv|GBSK01009566.1
Pla|ADV78070.1
Atr|scaffold00101.38
Os|LOC_Os05g41090.1
(14) CUterminal without domain detected
Sm|Sm99940|PACid:15420380
Atr|scaffold00069.118
Vv|VIT_16s0100g00340
Os|LOC_Os06g03682.1
(15) no AID nor CaMULD, found in Lycophytes to flowering plants
Sm|Sm99940|PACid:15420380
Atr|scaffold00148.66
At|At3g04530
(16) FBG domain at the C-terminal
Gm|Glyma07g18310.1
Gm|Glyma18g43160.1
Rc|28308.m000065
(17) Transmembrane domain at the C-terminal, found only in flowering plants
Pm|Pm007589
Pm|Pm023987
(18) S4 domain found in the C-terminal, found only in flowering plants
Al|339776
Lu|Lus10005619

Gr|Gorai.009G191500.1

Supplemental References

Aldous S. 2014. Evolution of the phosphoenolpyruvate carboxylase protein kinase family in C3 and C4 Flaveria spp. Plant Physiol. 165: 1076–1091.

Boudsocq M, Sheen J. 2013. CDPKs in immune and stress signaling. Trends Plant Sci 18: 30–40.

Khraiwesh B. 2015. Genome-wide expression analysis offers new insights into the origin and
evolution of Physcomitrella patens stress response. Sci. Rep. 5: 17434.

Yang X. 2015. A roadmap for research on crassulacean acid metabolism (CAM) to enhance sustainable food and bioenergy production in a hotter, drier world. New Phytol. 207: 491–504.
